# Selective Editing and Functionalization of the Mammalian Lipidome

**DOI:** 10.64898/2026.04.24.720406

**Published:** 2026-04-27

**Authors:** Binyou Wang, Lukas Lüthy, Logan Tenney, Leo Qi, Takeshi Harayama, Kim Ekroos, Johannes Morstein

**Affiliations:** a Division of Chemistry and Chemical Engineering, California Institute of Technology, Pasadena, California 91125, USA; b Institut de Pharmacologie Moléculaire et Cellulaire, Université Côte d’Azur - CNRS UMR7275 - Inserm U1323, Valbonne, France; c Lipidomics Consulting Ltd., Esbo, Finland

## Abstract

Lipids exhibit extraordinary molecular diversity, yet tools to selectively manipulate defined lipid classes in living cells are lacking. Here we show that lipid tail structure biases metabolic fate, enabling the design of synthetic lipid analogs with programmable metabolic selectivity. This approach enables selective cellular production of distinct lipid species or subclasses, including types of neutral lipids, phospholipids, sphingolipids, and ether lipids, without genetic or enzymatic perturbation. We further couple metabolic selectivity to chemical functionalization using bifunctional lipids, in which one modification directs metabolic flux and a second enables bioorthogonal tagging. Using this strategy, we achieve selective *in situ* labeling of different lipid pools in living cells. Together, our work establishes a chemical biology strategy that enables unprecedented precision in modulating, functionalizing, and rewiring the mammalian lipidome.

## Introduction

Lipids are central to cellular organization, metabolism, and signaling, yet they remain one of the least tractable classes of biomolecules for systematic functional interrogation.^1–3^ Lipid databases such as SwissLipids^4^ and LIPID MAPS^5^ now catalog over 10^6^ distinct lipid species, underscoring the vast chemical diversity of the lipidome. Despite this complexity, tools to selectively manipulate defined lipid classes or species in living cells are limited, restricting our ability to establish causal links between lipid identity and cellular function.^6–8^ Current approaches to perturb lipid biology rely largely on genetic or pharmacological modulation of lipid-metabolizing enzymes, which often produce broad and pleiotropic effects.

Recent advances in lipid chemical biology have significantly expanded our ability to interrogate lipid function in living systems.^9^ Enzyme-based membrane editors enable targeted remodeling of lipid headgroups at defined cellular locations with temporal control.^10,11^ Bifunctional phospholipids can be directly inserted into cellular membranes and subsequently fixed and labeled, providing a powerful approach to visualize lipid trafficking and distribution in living cells.^12,13^ Broad metabolic labeling strategies combined with organelle- or leaflet-specific reporters further enable spatially resolved analysis of lipid incorporation and turnover.^14,15^ Despite these advances, existing approaches primarily enable the observation or localized modification of lipids and do not provide a general strategy to control how lipids are routed through endogenous metabolic networks. Emerging evidence indicates that lipid tail structure is a key determinant of metabolic fate.^16^ We therefore reasoned that systematic chemical modification of lipid substrates could be used to bias their routing through metabolic networks and produce selective outcomes. In contrast to classical analog-sensitive chemical genetics,^17^ which relies on protein engineering to create orthogonal enzyme-substrate pairs, we hypothesized that native lipid metabolic pathways can discriminate neosubstrates through differential shape sensing in lipid binding pockets.

Here, we systematically define the structural dependence of lipid metabolism and test whether lipid tails can be engineered to control metabolic routing. Across diverse lipid classes, we show that lipid tail modifications enable selective metabolic routing and functionalization, establishing a general chemical biology strategy to edit and functionalize the mammalian lipidome. This approach creates new opportunities to interrogate lipid biology and to reprogram lipid flux in metabolic, neurological, and other diseases linked to dysregulated lipid metabolism.^18^

## Results

**Figure 1 |:**
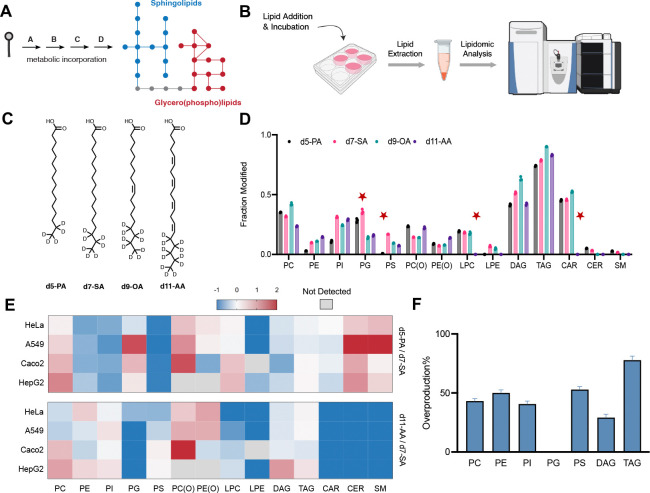
Lipid tail structure directs metabolic fate across the mammalian lipidome. **(A)** Schematic illustrating incorporation of exogenous lipid into cellular lipidome. **(B)** Schematic of chemical lipidomics workflow. Cells were treated with defined lipid species, followed by incubation, lipid extraction, and quantitative lipidomics analysis. **(C)** Chemical structures of isotopically labeled fatty acids: d5-palmitic acid (d5-PA), d7-stearic acid (d7-SA), d9-oleic acid (d9-OA), and d11-arachidonic acid (d11-AA). **(D)** Lipidomics analysis showing incorporation of labeled fatty acids (50 μM for 4 h in HeLa cells) across major lipid classes, shown as the fraction of modified lipids relative to endogenous levels (n = 3, mean ± s.d.). **(E)** Heatmap comparing relative incorporation efficiencies, expressed as ratios of d5-PA to d7-SA and d11-AA to d7-SA, across multiple cell lines (HeLa, A549, Caco-2, and HepG2). **(F)** Relative overproduction of lipid classes upon treatment with d7-SA compared to untreated controls (n = 6, mean ± s.d.).

Fatty acids are central lipid metabolites and are incorporated into diverse lipid classes, including phospholipids, neutral lipids, lysolipids, and sphingolipids through the action of various lipid trafficking and metabolizing enzymes (Figure 1A). In humans, some fatty acids are exclusively sourced exogenously and dietary interventions with certain fatty acids have shown benefits in several clinical trials.^19,20^ To systematically map the structural dependence of lipid metabolism, we established an untargeted chemical lipidomics platform in which we treat cells with labeled lipids for a defined time, perform lipid extraction with MTBE,^21^ and conduct lipidomic analysis^22^ capturing both endogenous and labeled lipid metabolites (Figure 1B). Comparing four common fatty acids with distinct chemical structures (Figure 1C), we observed pronounced structural dependence in their metabolic incorporation across lipid classes (Figure 1D). Palmitic acid (d5-PA) showed reduced incorporation into phosphatidylethanolamine (PE), phosphatidylinositol (PI), and phosphatidylserine (PS), whereas arachidonic acid (d11-AA) was poorly incorporated into lysolipids and acylcarnitine (CAR). Saturated fatty acids were incorporated more effectively into phosphatidylglycerol (PG) and oleic acid showed the highest incorporation into triacyclglycerols (TAG) consistent with its role as a potent lipid droplet inducer.^23,24^ To assess cell-type dependence, we extended this analysis across HeLa, A549, Caco-2, and HepG2 cells and found that relative incorporation trends were conserved (Figure 1E). Different lipid classes exhibited distinct incorporation kinetics (Figure S1A–F) and the overall incorporation selectivity patterns were robust and not effected by variables such as cellular confluence (Figure S1G). MS/MS analysis of d7-SA revealed minimal elongation or truncation after 4 h, with limited desaturation primarily funneled into phosphatidylcholine (PC) (Figure S1H). All detected metabolites, including elongated, truncated, and desaturated species, were included in the analysis to capture overall metabolic selectivity. To determine whether fatty acid supplementation alters total lipid output, we quantified lipid overproduction by comparing the sum of endogenous and labeled lipids to untreated controls (Figure 1F). PC, PE, PI, PS, DAG, and TAG showed clear increases, whereas total PG levels remained constant, suggesting tighter homeostatic regulation of PG and compensation via reduction of endogenous species within this class.

**Figure 2 |:**
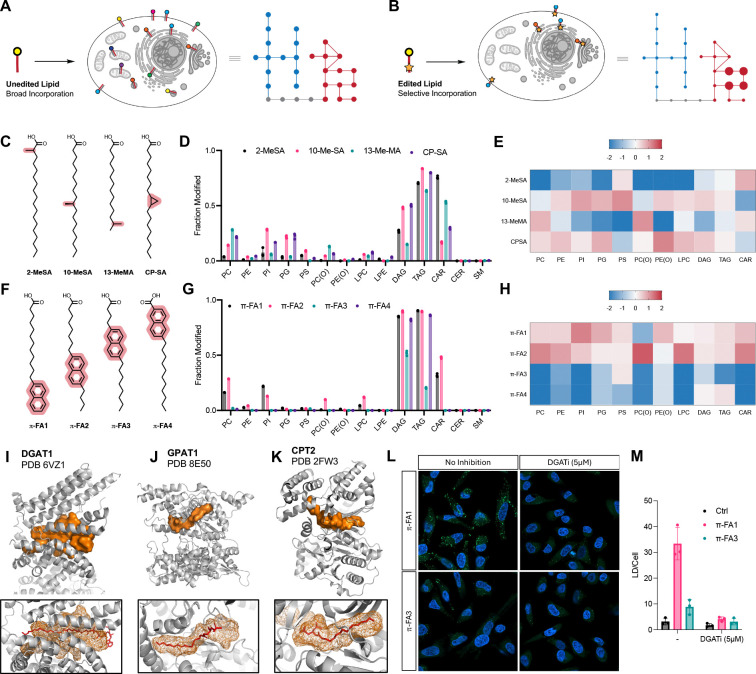
Chemical Editing of the Glycero(phospho)lipidome. **(A)** Schematic of broad lipidome-wide incorporation of endogenous fatty acids. **(B)** Schematic of selective incorporation of a chemically edited fatty acid. **(C)** Chemical structures of branched fatty acids. **(D)** Lipidomics analysis showing incorporation of branched fatty acids (50 μM for 4 h in HeLa cells) across major lipid classes, shown as the fraction of modified lipids relative to endogenous levels (n = 3, mean ± s.d.). **(E)** Heat map showing the relative change in branched fatty acid incorporation (log_2_-fold). **(F)** Chemical structures of aromatic fatty acids. **(G)** Lipidomics analysis showing incorporation of aromatic fatty acids (50 μM for 4 h in HeLa cells) across major lipid classes, shown as the fraction of modified lipids relative to endogenous levels (n = 3, mean ± s.d.). **(H)** Heat map showing the relative change in aromatic fatty acid incorporation (log_2_-fold). **(I–K)** Map of lipid binding pocket in lipid metabolic enzymes DGAT1 (PDB 6VZ1^25^), GPAT1 (PDB 8E50^26^), and CPT2 (PDB 2FW3^27^) generated using PyVOL.^28^ **(L)** Lipid Droplet Imaging in HeLa cells treated with 50 μM aromatic fatty acid for 4 h in the presence and absence of DGAT1 (15 μM A-922500) inhibitors. Lipid Droplets were stained with BODIPY 493/503 and nuclei with Hoechst 33342. **(M)** Quantification of lipid droplet content per cell (n = 3, mean ± s.d.).

While our data demonstrates dependence of metabolic fate on the structure of a fatty acid tail, overall incorporation of endogenous lipids remains relatively broad (Figure 2A). We reasoned that further chemical diversification could generate novel and more selective incorporation patterns, enabling targeted modulation of the lipidome (Figure 2B). To explore the feasibility of this hypothesis, we profiled branched fatty acids bearing methyl groups at defined positions, as well as a cyclopropyl analog (Figure 2C). These non-native lipids exhibit minimal endogenous background and are readily distinguishable in mass and retention time, enabling direct tracking without incorporation of isotopic labels. Although such branched fatty acids are not produced in mammalian cells, they occur in microorganisms and may influence host metabolism through the gut microbiome.^29^ Notably, we found striking differences in the metabolism and more selective incorporation patterns compared to the previously profiled dietary fatty acids (Figure 2D,E). **2-MeSA** was selectively routed into neutral lipids and acylcarnitine, with minimal phospholipid incorporation. In contrast, **10-MeSA** efficiently entered phospholipids with enrichment in PI and PG, whereas **13-MeMA** showed the opposite preference, favoring PC and (O)-PC over PI and PG. **CP-SA** exhibited a profile similar to **10-MeSA**, consistent with the shared position of modification. We next examined aromatic fatty acids with bulkier substitutions along the tail (Figure 2F). Similar to **2-MeSA**, an modification near the headgroup in **π-FA4** afforded selective incorporation into neutral lipids, with no detectable phospholipids or acylcarnitines (Figure 2G,H). In contrast, distal modifications (**π-FA1** and **π-FA2**) exhibited incorporation into both neutral lipids and phospholipids, albeit with distinct class distribution. Together, these results demonstrate that lipid tail modifications can program metabolic selectivity, enabling targeted enrichment of neutral lipids or defined phospholipid subsets. These patterns likely reflect structural constraints within lipid metabolic enzymes that encode differential substrate recognition. To test this hypothesis, we mapped the lipid binding pockets in three key metabolic enzymes, DGAT1, GPAT1, and CPT2 using PyVOL^28^, and found substantial variation in pocket size and shape. DGAT1 exhibits the largest and most diffuse lipid binding pocket, consistent with its ability to accommodate bulky modifications and route substrates into neutral lipid metabolism (Figure 2I). In contrast, GPAT1 contains a smaller, more restrictive pocket (Figure 2J), while CPT2 displays an intermediate architecture (Figure 2K), consistent with selective conversion of certain fatty acids into acylcarnitines and exclusion of others. Finally, we asked whether selective lipid metabolism translates into lipid-dependent cellular phenotypes by examining lipid droplet formation. **π-FA1** was efficiently converted into TAGs, whereas **π-FA3** showed minimal TAG formation. Consistent with these metabolic differences, **π-FA1** robustly induced lipid droplet formation, while **π-FA3** had little effect (Figure 2L,M).

**Figure 3 |:**
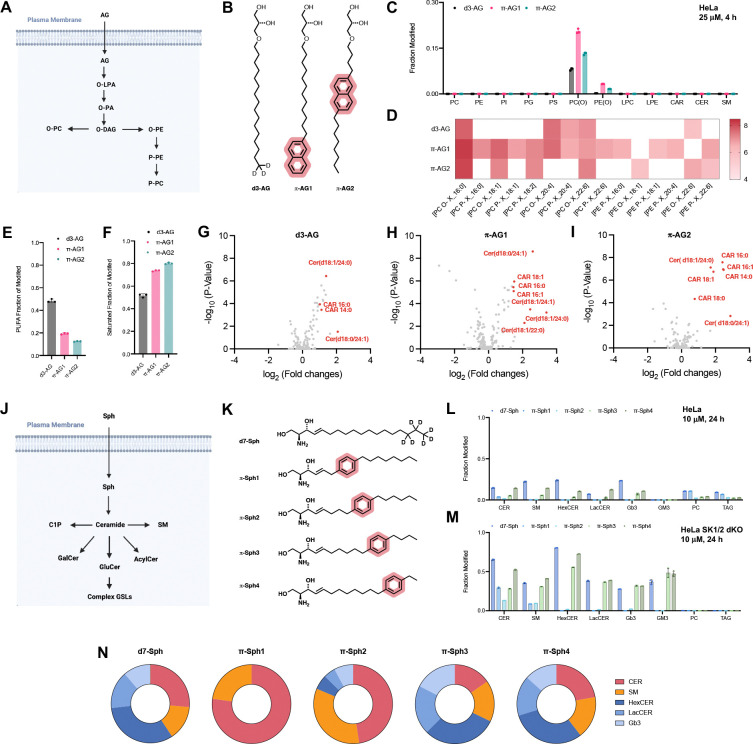
Chemical editing of the ether- and sphingolipidome. **(A)** Schematic of alkylglycerol (AG) metabolism. **(B)** Chemical structures of modified alkylglycerols. **(C)** Lipidomics analysis showing incorporation of alkylglycerols (50 μM for 4 h in HeLa cells) across major lipid classes, shown as the fraction of modified lipids relative to endogenous levels (n = 3, mean ± s.d.). **(D)** Heat map showing the relative levels (log_10_) of ether lipids formed from modified alkylglycerols (50 μM for 4 h). **(E)** Fraction of polyunsaturated lipid tails (PUFAs) in modified ether lipids. **(F)** Fraction of saturated lipid tails in modified ether lipids. **(G–I)** Volcano plots showing changes in endogenous lipids upon alkylglycerol treatments. **(J)** Schematic of sphingosine (Sph) metabolism. **(K)** Chemical structures of aromatic sphingosine analogues. **(L)** Lipidomics analysis showing incorporation of sphingosines (10 μM for 24 h in HeLa cells) across different lipid classes, shown as the fraction of modified lipids relative to endogenous levels (n = 3, mean ± s.d.). **(M)** Lipidomics analysis showing incorporation of sphingosines (10 μM for 24 h in HeLa SK1/2 dKO cells) across different lipid classes, shown as the fraction of modified lipids relative to endogenous levels (n = 3, mean ± s.d.). **(N)** Distribution of modified lipid fractions of **d7-Sph** and **π-Sph1–4** after 24 treatment of HeLa SK1/2 dKO cells in **(M)**.

We next asked whether combining chemical modifications with distinct metabolic entry points could further expand control over lipid incorporation. Alkylglycerols are thought to be selectively routed into ether lipids (Figure 3A).^30^ To test this, we synthesized deuterated and aromatic analogs (Figure 3B) and observed highly selective incorporation into ether lipids (Figure 3C; Figure S2A–H). Beyond class-level selectivity, these modifications enabled tuning of specific ether lipid species within a class, affording a level of control not readily achievable with genetic or pharmacological approaches (Figure 3D–F; Figure S2I–N). Supplementation with ether lipids further induced secondary changes to endogenous lipid pools (Figure 3G–I), including remodeled ceramides, consistent with reported crosstalk between ether lipids and sphingolipids.^31^ Notably, acylcarnitines were strongly upregulated following alkylglycerol treatment, suggesting a previously unrecognized link between ether lipid metabolism and acylcarnitine-associated energy metabolism.

To extend this approach to sphingolipids, we focused on sphingosine as a central metabolic precursor. Ceramides are the central branch point of sphingolipid metabolism, but their poor cell permeability limits their direct use.^32,33^ In contrast, sphingosine is readily taken up and converted into ceramides by ceramide synthases^34^, followed by metabolism into major sphingolipid classes, including sphingomyelin and complex glycosphingolipids (Figure 3J).^35,36^ We synthesized four aromatic sphingosine derivatives (Figure 3K) and found distinct selectivity patterns: **π-Sph1** and **π-Sph2** were preferentially converted into sphingomyelin, whereas **π-Sph3** and **π-Sph4** were incorporated into sphingomyelin and complex glycosphingolipids (Figure 3L; Figure S3A,B To prevent conversion of sphingosine (Sph) into fatty acids via S1P and thereby enhance flux into complex sphingolipids, we employed a double sphingosine kinase knockout HeLa cell line, which resulted in increased sphingolipid production (Figure 3M,N; Figure S3C–F). These results demonstrate that sphingolipid class distribution can be selectively tuned through substrate design, with potential relevance for diseases characterized by aberrant glycosphingolipid accumulation, such as Gb3 buildup in Fabry’s disease.^37^

**Figure 4 |:**
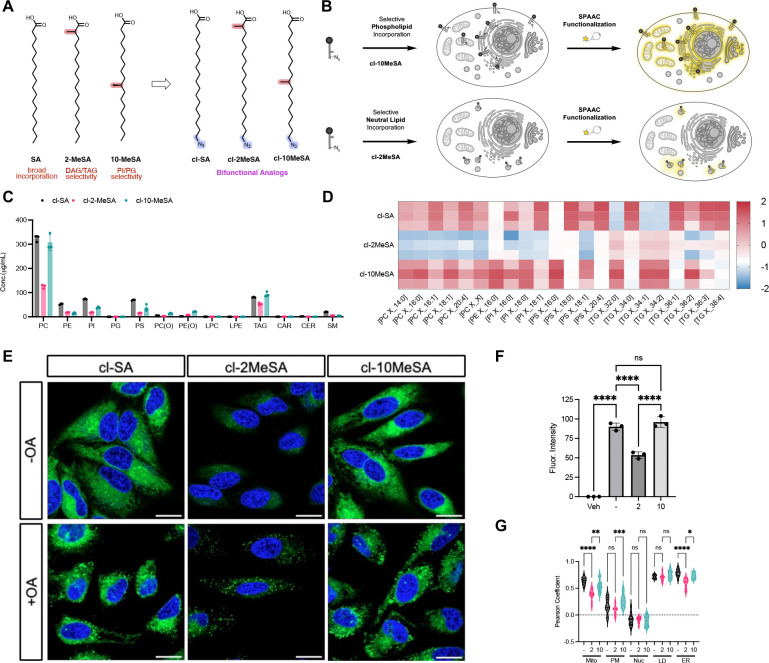
Selective *in situ* lipid functionalization. **(A)** Design and chemical structures of clickable branched fatty acid analogues. **(B)** Schematic illustrating the selective metabolic incorporation of bifunctional fatty acid analogues and functionalization using click chemistry. **(C)** Lipidomics analysis of functionalized probes (50 μM for 4 h in HeLa) showing incorporation across major lipid classes. Absolute concentrations (μg/mL) were quantified using class-specific deuterated internal standards (n = 3; mean ± s.d.). **(D)** Heat map comparing the relative incorporation of each probe into lipid species, normalized by z-score across treatments. **(E)** Confocal imaging of bifunctional fatty acid analogues in live cells after 4 hour incubation and subsequent SPAAC labeling with BODIPY-BCN (CO-1^38^). Images were taken with and without co-treatment of oleic acid to induce lipid droplet formation. Scale bar represents 20 μm. **(F)** Fluorescence intensities after labeling of bifunctional lipids (n = 3; mean ± s.d.). **(G)** Pearson correlation coefficients between the fluorescent signal of each probe and markers for different cellular organelles (n = 12; mean ± s.d.; *p<0.05, **p<0.01, ***p<0.001, ****p<0.0001).

Chemically modified lipids with selective metabolic routing enable targeted overproduction of defined lipid classes, providing a new level of control over lipid metabolism. We reasoned that this selectivity could be extended to enable class-specific functionalization. To test this, we designed bifunctional fatty acid analogs comprising a chemical modification that directs metabolic routing and an azide handle for bioorthogonal functionalization via strain-promoted azide-alkyne cycloaddition (SPAAC^39^; Figure 4A). We hypothesized that **cl-2MeSA** would preferentially incorporate into neutral lipids, while **cl-10MeSA** would target phospholipids with a distinct incorporation profile relative to **cl-SA** (Figure 4B). Lipidomic analysis demonstrated preferential neutral lipid incorporation of **cl-2MeSA** and increased phospholipid labeling with **cl-SA** and **cl-10MeSA** (Figure 4C,D). While bifunctional analogs retained selectivity, it was less pronounced than for **2-MeSA** and **10-MeSA** (Figure 2), indicating that the azide modification influences metabolic incorporation. Consistent with this, all three clickable analogs showed increased β-oxidation, likely due to the extended tail length introduced by the azide, which reduces selective incorporation patterns (Figure S4A–H). Further structural optimization is therefore likely to enhance selectivity. Despite this, we observed pronounced differences in the pools of functionalized lipids by live-cell imaging following SPAAC labeling with a low-background strained-alkyne fluorophore (CO-1^38^; Figure 4E). **cl-SA** and **cl-10MeSA** preferentially labeled phospholipids, resulting in strong fluorescence at the endoplasmic reticulum and associated membranes, consistent with sites of phospholipid synthesis and remodeling (Figure 4F). In contrast, under conditions that promote lipid droplet formation, **cl-2MeSA** selectively labeled neutral lipid pools and lipid droplets, whereas **cl-SA** and **cl-10MeSA** remained enriched in phospholipids (Figure 4F,G; Figure S4I,J). Together, these results establish a strategy for *in situ* functionalization of distinct lipid pools, enabling selective visualization of newly synthesized lipids and, more broadly, chemical targeting of defined lipid classes.

## Conclusion

In this work, we show that lipid tail structure is a programmable determinant of metabolic fate and that this principle can be leveraged to selectively route substrates through endogenous lipid metabolic networks. By systematically modifying central lipid metabolites and quantifying their downstream incorporation, we demonstrate that chemical variation of fatty acids, alkylglycerols, and sphingosines modulates metabolic flux and generates distinct lipid profiles. Fatty acid tail architecture strongly influenced incorporation into phospholipids, neutral lipids, lysolipids, and acylcarnitines, and modified branched and aromatic fatty acids produced strikingly selective routing patterns, enabling preferential enrichment of neutral lipids or defined phospholipid subsets. Modified alkylglycerols enabled selective routing into ether lipids and chemical modifications lead to distinct species distributions. Chemically modified sphingosines provided selective access to distinct sphingolipid classes, with some analogs preferentially routed into sphingomyelin and others into both sphingomyelin and complex glycosphingolipids.

We further couple metabolic selectivity to chemical functionalization. Bifunctional lipid analogs bearing a routing element and a bioorthogonal handle enable delivery of chemical functionality to defined lipid pools in living cells, allowing selective installation of probes, such as fluorophores or other functional groups, into newly synthesized cellular lipids. Together, our findings establish substrate-directed metabolic control as a broad strategy for selectively modulating the mammalian lipidome. Precision control of the lipidome expands our ability to interrogate lipid function and may enable selective lipidome reprogramming in disease.

## Synthetic Chemistry

### General Practice

Unless otherwise noted, all non-aqueous reactions were carried out under Nitrogen atmosphere, in oven dried glassware. Reagents were purchased from commercial suppliers (ABCR, ACROS, Sigma Aldrich, Ambeed, TCI, Strem, Alfa, Combi-Blocks or Fluorochem) and used without further purification. Anhydrous solvents over molecular sieves were purchased from Acros and used as received. Analytical thin layer chromatography (TLC) was performed on Merck silica gel 60 F254 TLC glass plates and visualized with 254 nm light and potassium permanganate (1.50 g KMnO_4_, 10.0 g K_2_CO_3_, 1.25 mL 10% NaOH, 200 mL water), or ceric ammonium molybdate (10.0 g Cerium(IV)sulfate, 25.0 g phosphomolybdic acid, 940 mL water 60.0 mL conc. sulfuric acid) staining solutions followed by heating. Organic solutions were concentrated by rotary evaporation at 40 °C. Chromatographic purification of reaction products was carried out by flash chromatography using Millipore Silica gel 60 (0.063–0.200 mm), under 0.3–0.5 bar overpressure.

### NMR Spectroscopy

^1^H NMR spectra were recorded on a Bruker AVIII 400 MHz spectrometer with a prodigy cryoprobe and are reported in ppm with the solvent resonance as the reference (CDCl_3_ at 7.26 ppm, MeOH-d_4_ at 3.31 ppm, DMSO-d_6_ at 2.50). Peaks and their apparent multiplicities are reported as (s = singlet, d = doublet, t = triplet, q = quartet, m = multiplet, br = broad signal, coupling constant(s) in Hz, integration). ^13^C NMR spectra were recorded with 1H-decoupling on Bruker AVIII 101 MHz spectrometer prodigy cryoprobe and are reported in ppm with the solvent resonance as the reference unless noted otherwise (CDCl_3_ at 77.16 ppm, MeOH-d_4_ at 49.00 ppm DMSO-d_6_ at 39.52 ppm).

### HRMS

High resolution mass spectrometric data were obtained on an Agilent 6230 LC-TOF and are reported as (*m/z*).

### Cell culture.

All cell lines, including HeLa, A549, Caco-2, and HepG2 cells, were cultured in T75 flasks (Fisher Scientific, FB012937) in Dulbecco’s Modified Eagle Medium (DMEM; high glucose, sodium pyruvate, and L-glutamine; GenClone, 25–500) supplemented with 10% (v/v) fetal bovine serum (FBS; GenClone, 25–550) and 1% (v/v) penicillin–streptomycin (P/S). HeLa SK1/2 KO were previously described.^40^

## Lipidomic Analysis

### Materials

Materials used for LC–MS were water, Optima^™^ LC/MS Grade (Fisher Scientific, W6–4), acetonitrile, Optima^™^ LC/MS Grade (Fisher Scientific, A955–4), Isopropanol, Optima^™^ LC/MS Grade (Fisher Scientific, A461-A), ammonium formate (Sigma-Aldrich, 70221–25G-F) and formic acid Optima^™^ LC/MS Grade (Fisher Scientific, A117–50). Solvents for lipid extraction were tert-butyl methyl ether (Sigma-Aldrich, 34875) and methanol (Fisher Scientific, A456). The lipid internal standard mixture was Deuterated Lipidomics MaxSpec ^®^ Mixture (Cayman Chemical, 40974).

### Lipid extraction

Lipid extraction was performed using a tert-butyl methyl ether (MTBE)-based protocol.^41^ Cells were washed twice with ice-cold PBS, harvested from 6-well plates, and pelleted at 800 × g for 5 min at 4 °C. Pellets were resuspended in 70 μL water, followed by addition of 232 μL methanol and 20 μL (10× diluted) internal standard mixture. Subsequently, 840 μL MTBE was added and samples were incubated for 1 h at room temperature with shaking. Phase separation was induced by adding 150 μL water, followed by incubation for 5 min at room temperature and centrifugation at 1,000 × g for 10 min. The upper organic phase (750 μL) was collected and transferred to glass autosampler vials (Thermo, 6PSV9–1PG) and dried overnight under vacuum.

### Liquid Chromatography

Dried lipid extracts were reconstituted in 500 μL of Solvent B (isopropanol/acetonitrile/water, 88:10:2, vol/vol) containing 10 mM ammonium formate and 0.1% (vol/vol) formic acid. Lipids were separated by reversed-phase LC on a Vanquish Core system (Thermo Fisher Scientific) equipped with an Accucore C30 column (150 × 2.1 mm, 2.6 mm, 150 Å, Thermo Fisher Scientific). Mobile phase A from LC system was acetonitrile/water, 60:40, vol/vol with 10 mM ammonium formate and 0.1% (vol/vol) formic acid and Mobile phase B from LC system was isopropanol/acetonitrile/water, 88:10:2, vol/vol both containing 10 mM ammonium formate and 0.1% (vol/vol) formic acid. Separation was performed at 45 °C at a flow rate of 0.26 mL/min using the following gradient: 0–2 min, 30–43% B (curve 5); 2.1–12 min, 43–55% B (curve 5); 12–18 min, 65–85% B (curve 5); 18–20 min, 85–100% B (curve 5); 20–25 min, 100% B isocratic; 25.1–28 min, 100%–30% B (curve 5) followed by 7 min re-equilibration at 30% B.

### Mass spectrometry

LC was coupled to an Orbitrap Exploris^™^ 240 mass spectrometer (Thermo Fisher Scientific) with a heated electrospray ionization (HESI) source. Mass spectra were acquired in positive and negative modes with the following ESI parameters: sheath gas, 40 (Arb); auxiliary gas, 10 (Arb); sweep gas, 0 (Arb); spray voltage, (+)3.25 kV (positive ion mode); (−)3 kV (negative ion mode); Ion transfer tube temperature, 300 °C; S-lens RF level, 70; Vaporizer temperature, 275 °C. Data acquisition for lipid identification was performed in data-dependent acquisition mode (DDA) full scan with/without MS/MS. The full scan has the resolution of 120,000, AGC target set to standard mode, maximum injection time set to auto mode in a scan range of m/z 100–1,200. Data-dependent MS/MS scans were acquired with a resolution of 15,000, AGC target set to standard mode, maximum injection time set to auto mode, isolation window of 1 m/z and stepped normalized collision energies of 20, 30 and 40. A data-dependent MS2 was triggered (Cycle time of 1.2 s) when an AGC target of 2.5e3 was reached followed by a dynamic exclusion for 10 s. All isotopes and charge states >1 were excluded. Full scan spectra were acquired in profile type and MS/MS scans were acquired in centroid type. Data for lipid quantification in individual samples were acquired in full scan MS mode with the following parameters: resolution of 120,000, AGC target set to standard mode, maximum injection time set to auto mode in a scan range of m/z 100–1200.

### Lipid identification and quantification

Raw data were processed using Compound Discoverer v3.4 (Thermo Scientific) with a modified MetID Mass List Search workflow using the following parameters: m/z tolerance for precursor mass selection set to 5 ppm; minimum peak intensity threshold 30,000; S/N threshold set to 1.5; Peak rating threshold set to 0.4; Gap fill enabled with a 1.5 S/N threshold, 3 minimum scans per peak. A mass list having exact masses of both endogenous lipids and chemically modified lipids was imported for analysis. For lipid identification, triacylgylcerols, diacylglycerols were identified as [M + NH_4_]^+^ adducts. Lysophosphatidylcholines, and acyl-, ether- and vinyl ether-PC, acylcarnitines, ceramides and sphingomyelins were analyzed as [M + H]^+^ adducts. Lyso-phosphatidylethanolamines, and acyl-, ether- and vinyl ether-PE, phosphatidylserines, phosphatidylinositols and phosphatidylglycerol were analyzed as [M−H]^−^ adducts. Acyl-, ether- and vinyl ethers were identified as [M−H]^−^ adducts.^42^ Ether lipids and plasmalogens were identified based on MS/MS fragmentation, where diagnostic sn2 acyl chain fragments were used to determined fatty acyl composition. The lipid classes BMP and PG are isomeric and could not be distinguished with high confidence under the conditions used. Quantification was performed by peak integration of the extracted ion chromatograms of most common ion adducts. Peak integration was first performed by CD then manually curated and adjusted. Identified lipids were normalized to peak areas of added internal standards to decrease analytical variation. Raw lipidomics data were analyzed using Compound Discoverer and exported as .csv files and plotted in GraphPad Prism 11.0.0 (GraphPad Software).

### Lipid Droplet Imaging and Quantification

HeLa cells were seeded in 18-well Cellvis 18 Chambered Coverglass System (C18–1.5H), coated with poly-L-lysine, at the density of 15k cells/well for 12 hours prior to treatment in DMEM (10% FBS, 1% P/S). Cells were incubated with DMEM (10% FBS, 1% P/S) containing 50 μM of π-FA1 and π-FA3 respectively for 6 hours. After incubation, the cells were washed with warm PBS and fixed with 4% (w/v) PFA for 8 minutes. Lipid droplets were stained with 1 μM BODIPY 493/503 at 37°C for 20 minutes and the nuclei were stained with 1 μg/mL Hoechst 33342 at 37°C for 15 minutes. Prior to imaging, cells were washed with PBS. The fixed cells were imaged using Zeiss LSM800 with a 40X water objective. Acquired images were saved as .Tif files for analysis. All images were processed with Fiji^43^ (ImageJ v2.16.0/1.54p). For each channel in the representative fluorescence microscopy image, the images are first converted to 8-bit format and adjusted the brightness (min:32, max:255). The merged images were generated by merging the figure using processed images obtained from the DAPI and BODIPY channel. For nuclei quantification, the original image from the DAPI channel were converted into 8-bit format and set with adjusted threshold (min:46, max:255). The function, find edge, was applied prior to using analyze particle function (size: 500-infinity pixel) to quantify the number of nuclei. For LD quantification, the original image from BODFI channel were converted into 8-bit format and set with adjusted threshold (min: 70, max: 255). The image is converted to mask and quantified using the analyze particle function (size: 0-Infinity, circularity: 0.20–1.00 pixel). The LDs/cell was calculated by dividing the number of LDs in each image by the number of nuclei in each image. The data was plotted using GraphPad Prism. Unpaired t-test with Welch’s correction was used to determine if the difference between treatments were significant.

### SPAAC Imaging

HeLa cells were seeded in 8-well chamber with glass imaging slide (Cellvis #C8–1.5H-N) 24h before imaging experiment in DMEM (10% FBS, 1% P/S), such that cells grow to approximately 70% confluency at the time of imaging. The fatty acid (clSA, cl-2MeSA, cl-10MeSA) as a DMSO stock was diluted to a final concentration of 10 μM in warm DMEM (10% FBS, 1% P/S) with vehicle (DMSO) or oleic acid (40 μM) and a final concentration of DMSO of 0.5%, 200 μL of this solution was added to cells and cells incubated for 4 h at 37 °C and 5% CO_2_ (v/v). At this time, media is removed and replaced with 200 μL of 5 μM CO-1 in full media.^36^ The cells were incubated for 1h at 37°C and 5% CO2 (v/v). Media is then removed and replaced with 200 μL of 1 mg/mL Hoechst-33342 in phenol red-free DMEM media (10% FBS, 1% P/S) for 20 min or, for co-staining experiments, the organelle tracking dyes were used at the following concentration diluted in phenol red-free DMEM media (without FBS or P/S) and incubated for 10 min, BioTracker 405 Blue Mitochondria Dye (Millipore Sigma #SCT135, 100 nM) and CellMask^™^ Plasma Membrane Deep Red (Thermo Fisher C10046, 2 μg/mL) or ER-Tracker red (MCE #HY-D1431, 2 μM) and LipidSpotTM 610 (Biotium #70069-T, 1/1000 dilution). Cells were washed once with phenol red free DMEM media (10% FBS, 1% P/S) and replaced with fresh phenol red free DMEM media (10% FBS, + 1% P/S) for imaging. Cells were imaged with a Zeiss LSM 800, AxioObserver Z1 Inverted with Definite focus confocal microscope equipped with an incubation chamber, 405 nm, 488 nm, 561 nm and 633 nm excitation laser lines, GaAsP detectors, and a 63x oil immersion objective. In the image processing, FIJI was used to calculate fluorescence intensity for images of each probe. Cell regions to be quantified were selected by setting a threshold based after a Gaussian blur filter (sigma=1) was applied. Then “Create Mask” and “Create Selection” functions were used to select the area to be quantified based on the determined threshold. The mean fluorescence was then quantified for an image, which represented n=1, an ordinary one-way ANOVA was the statistical method used to compare the means of the treatment groups. For colocalization, the coloc2 plugin with FIJI was used to calculate Pearson’s correlation coefficient, where correlation is calculated for individual cells by drawing masks around individual cells, n=12 cells quantified per treatment condition, an ordinary one-way ANOVA was the statistical method used to compare the means of the colocalization coefficients.

## Supplementary Material

Supplement 1

Associated Content

Supporting information include synthetic procedures and NMR characterization data.
