# Supplementary material for: Selective Editing and Functionalization of the Mammalian Lipidome
Source: bioRxiv. 2026 Apr 27:2026.04.24.720406. Preprint. [Version 1] doi: 10.64898/2026.04.24.720406 (PMC13142499; doi:10.64898/2026.04.24.720406)
Supplement: Supplement 1 [file NIHPP2026.04.24.720406v1-supplement-1.pdf]

bioRxiv preprint doi: https://doi.org/10.64898/2026.04.24.720406; this version posted April 27, 2026. The copyright holder for this preprint (which was not certified by peer review) is the author/funder, who has granted bioRxiv a license to display the preprint in perpetuity. It is made available under aCC-BY 4.0 International license.

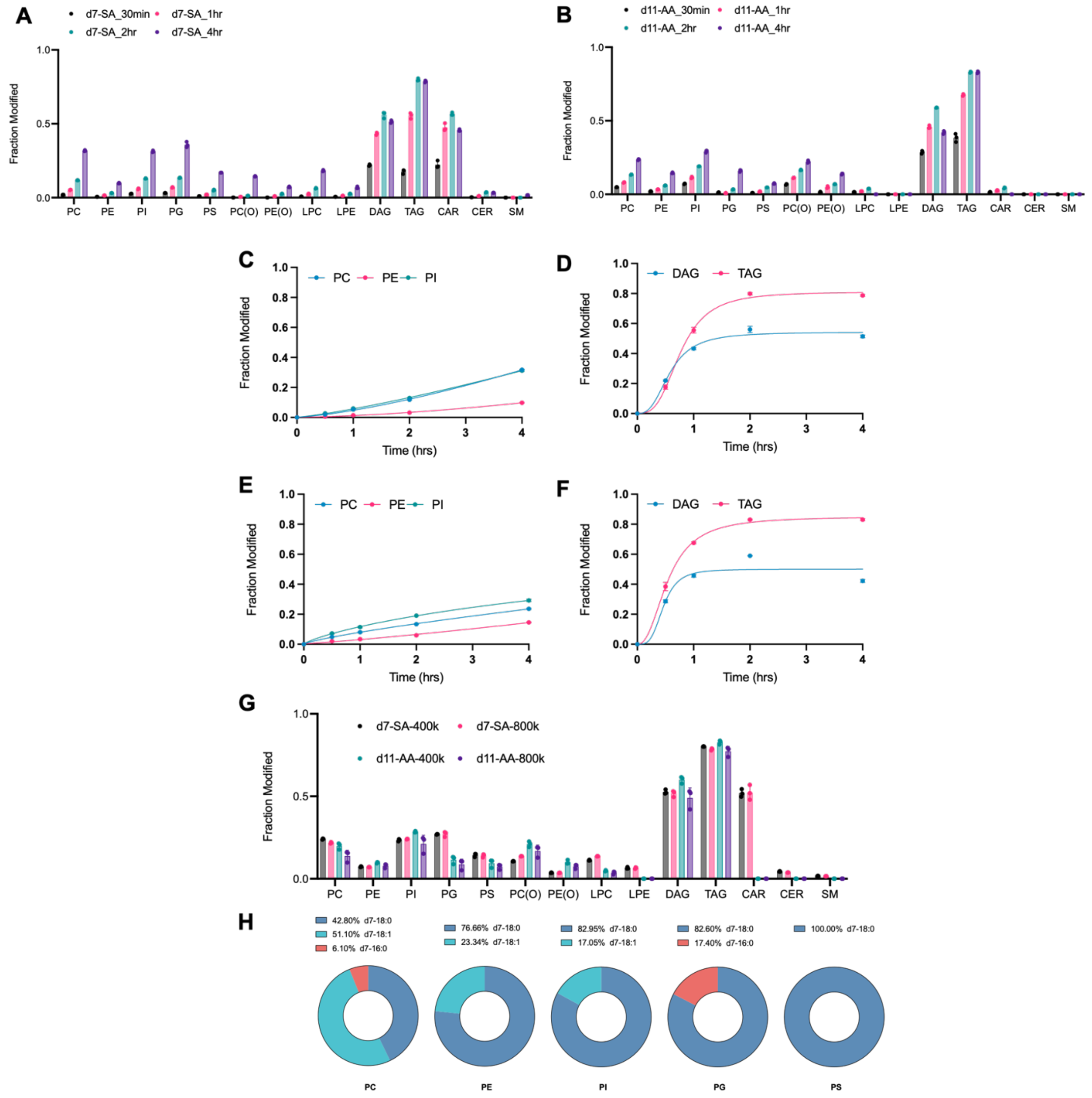

Extended Figure S1 | Expanded analysis of lipid tail-dependent metabolic routing.

(A) Lipidomics analysis of incorporation of d7-stearic acid (d7-SA; 50 μM) into major lipid classes in HeLa cells at different treatment times, shown as the fraction of modified lipids relative to endogenous levels (n=3; mean ± s.d.).**(B)** Lipidomics analysis of incorporation of d11-arachidonic acid (d11-AA; 50 μM) into major lipid classes at different treatment times, shown as the fraction of modified lipids relative to endogenous levels (n = 3; mean

bioRxiv preprint doi: https://doi.org/10.64898/2026.04.24.720406; this version posted April 27, 2026. The copyright holder for this preprint (which was not certified by peer review) is the author/funder, who has granted bioRxiv a license to display the preprint in perpetuity. It is made available under aCC-BY 4.0 International license.

± s.d.). **(C, D)** Nonlinear regression analysis of d7-SA incorporation kinetics into phospholipids (C) and neutral lipids (D). **(E, F)** Nonlinear regression analysis of d11-AA incorporation kinetics into phospholipids (E) and neutral lipids (F). **(G)** Lipidomics analysis of d7-SA and d11-AA incorporation at different HeLa cell culture confluencies (50 µM, 4 h), shown as the fraction of modified lipids relative to endogenous levels (mean ± s.d., n = 3). **(H)** Lipidomics analysis of d7-SA-derived tail-modified species incorporated into major lipid classes in HeLa cells following 4 h treatment at 50 µM (n = 3, mean ± s.d.). Analysis was based on MS/MS acyl chain composition.

bioRxiv preprint doi: https://doi.org/10.64898/2026.04.24.720406; this version posted April 27, 2026. The copyright holder for this preprint (which was not certified by peer review) is the author/funder, who has granted bioRxiv a license to display the preprint in perpetuity. It is made available under aCC-BY 4.0 International license.

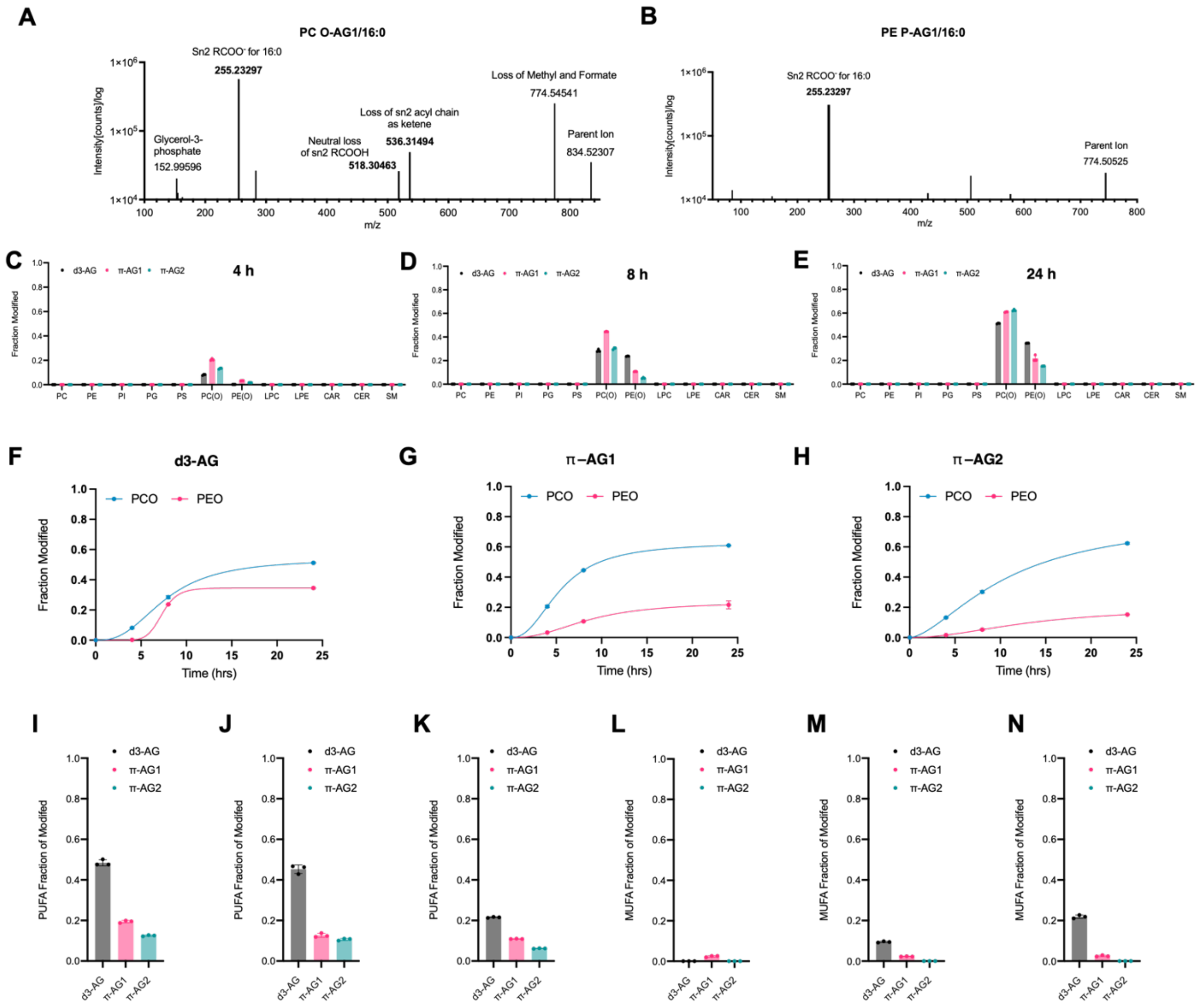

Extended Figure S2 | Expanded analysis of chemical ether lipid editing.

(A, B) Representative MS/MS spectra of modified alkylglycerol-incorporated ether phosphatidylcholine (PC) (**A**) and ether phosphatidylethanolamine (PE) (**B**). **(C–E)** Lipidomics analysis of incorporation of chemically modified alkylglycerols (50 µM) into major lipid classes over time, shown as the fraction of modified lipids relative to endogenous levels (*n* = 3; mean ± s.d.). **(F–H)** Nonlinear regression analysis of alkylglycerol incorporation kinetics. **(I–K)** Fraction of polyunsaturated fatty acyl chains (PUFAs) in modified ether lipids after 4h **(I)**, 8h **(J)**, and 24h **(K)**. **(L–N)** Fraction of

bioRxiv preprint doi: https://doi.org/10.64898/2026.04.24.720406; this version posted April 27, 2026. The copyright holder for this preprint (which was not certified by peer review) is the author/funder, who has granted bioRxiv a license to display the preprint in perpetuity. It is made available under aCC-BY 4.0 International license.

monounsaturated fatty acyl chains (MUFAs) in modified ether lipids after 4h **(L)**, 8h **(M)**, and 24h **(N)**.

bioRxiv preprint doi: https://doi.org/10.64898/2026.04.24.720406; this version posted April 27, 2026. The copyright holder for this preprint (which was not certified by peer review) is the author/funder, who has granted bioRxiv a license to display the preprint in perpetuity. It is made available under aCC-BY 4.0 International license.

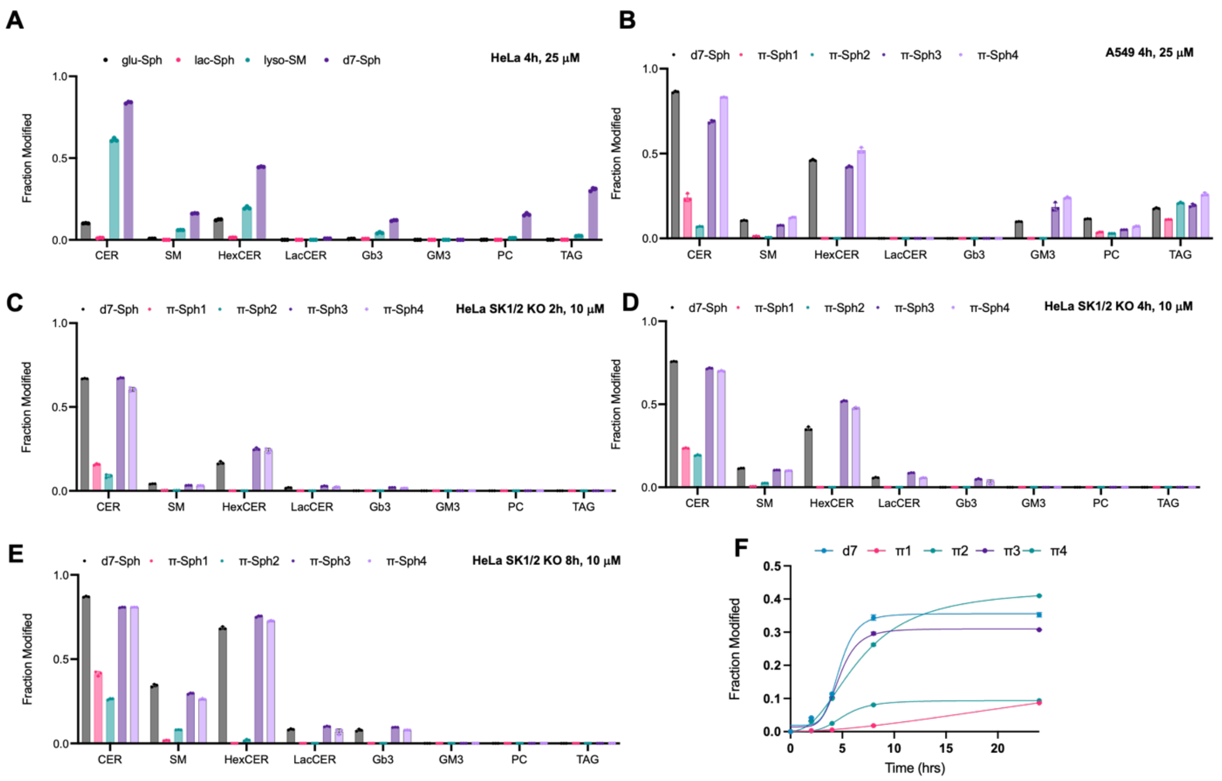

Extended Figure S3. Expanded analysis of chemical sphingolipid editing.

(A). Lipidomics analysis of incorporation of lyso-sphingolipids (25 μM, 4 h in HeLa cells) into major lipid classes, shown as the fraction of modified lipids relative to endogenous levels (n = 3; mean ± s.d.) **(B).** Lipidomics analysis of incorporation of aromatic sphingosines (25 μM, 4 h in A549 cells) into major lipid classes, shown as the fraction of modified lipids relative to endogenous levels (n = 3; mean ± s.d.). **(C-E).** Lipidomics analysis of incorporation of aromatic sphingosines (10 μM in HeLa cells) into major lipid classes across different treatment time, shown as the fraction of modified lipids relative to endogenous levels (n = 3; mean ± s.d.) **(F)** Nonlinear regression analysis of sphingomyelin (SM) incorporation kinetics.

bioRxiv preprint doi: https://doi.org/10.64898/2026.04.24.720406; this version posted April 27, 2026. The copyright holder for this preprint (which was not certified by peer review) is the author/funder, who has granted bioRxiv a license to display the preprint in perpetuity. It is made available under aCC-BY 4.0 International license.

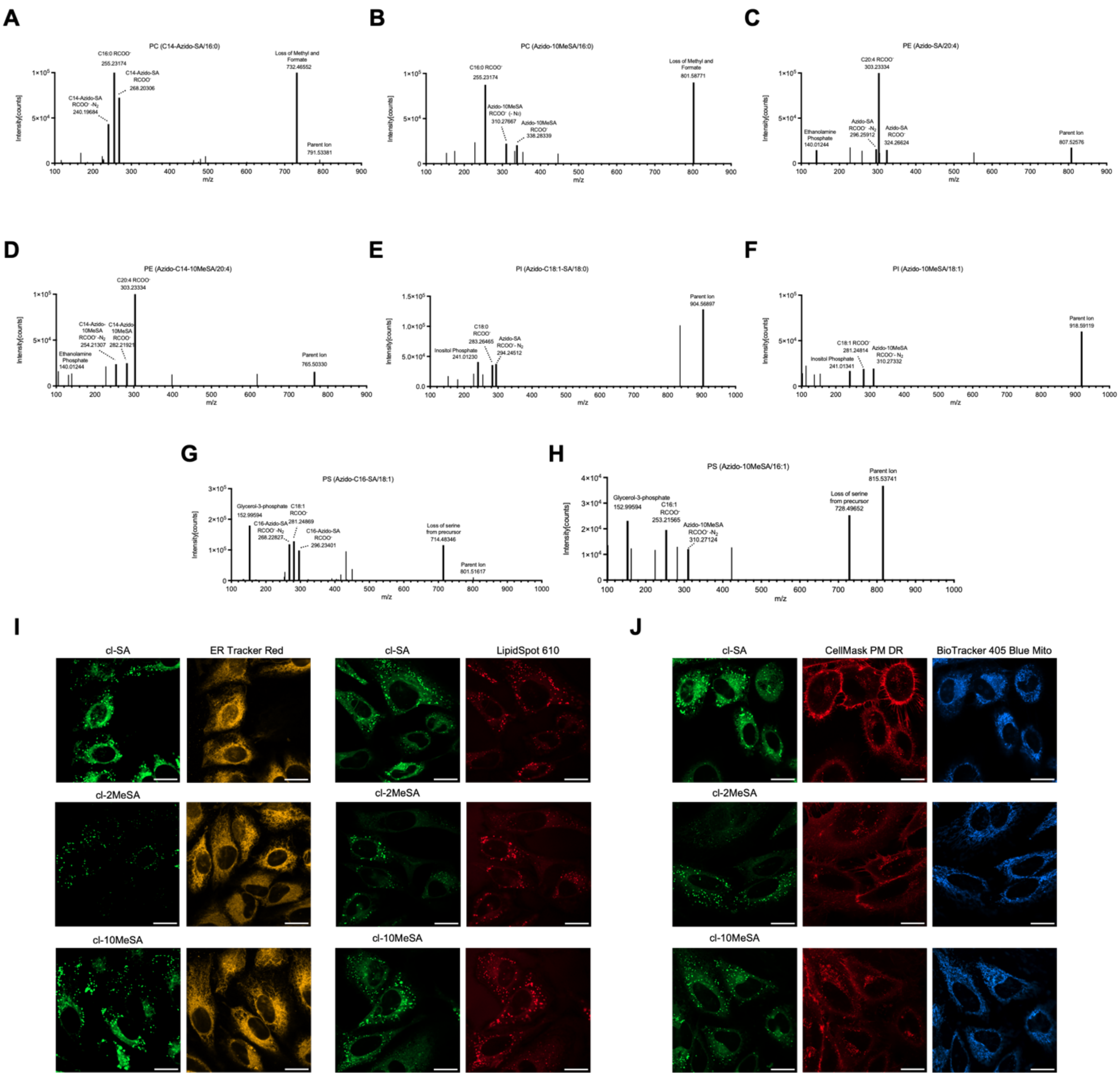

Extended Figure S4. Expanded analysis of in situ lipid functionalization.

(A, B) Confocal imaging of bifunctional fatty acid analogues (**cl-SA**, **cl-2MeSA**, and **cl-10MeSA**) in HeLa cells after 4 hour incubation and subsequent SPAAC labeling with BODIPY-BCN (CO-1[38]). Images were taken with co-treatment of oleic acid to induce lipid droplet formation. **(A)** Co-staining with LipidSpot 610 (1/1000 dilution) and ER-Tracker Red (1 μM). **(B)** Co-staining with CellMask Plasma Membrane Deep Red (2 μg/mL) and BioTracker 405 Blue Mitochondria (100 nM). **(C–J)** Representative MS/MS spectra of

bioRxiv preprint doi: https://doi.org/10.64898/2026.04.24.720406; this version posted April 27, 2026. The copyright holder for this preprint (which was not certified by peer review) is the author/funder, who has granted bioRxiv a license to display the preprint in perpetuity. It is made available under aCC-BY 4.0 International license.

modified glycerophospholipids incorporating azido-functionalized fatty acids and branched analogues. **(C, D)** MS/MS spectra of modified PC species. **(E, F)** MS/MS spectra of modified PE species. **(G, H)** MS/MS spectra of modified PI species. **(I, J)** MS/MS spectra of PS species. Diagnostic fragment ions are highlighted to support lipid class identification and incorporation of modified acyl chains.

bioRxiv preprint doi: https://doi.org/10.64898/2026.04.24.720406; this version posted April 27, 2026. The copyright holder for this preprint (which was not certified by peer review) is the author/funder, who has granted bioRxiv a license to display the preprint in perpetuity. It is made available under aCC-BY 4.0 International license.

Created by

Contents of Report https://lipidomicstandards.org, version v2.6.1

Separation Workflow 1
Overall study design . . . 1
Lipid extraction . . . 1
Analytical platform . . . 2
Quality control . . . 2
Method qualification and validation . . . 2
Reporting . . . 2

Sample Descriptions 2
epithelial cells / Human / Cells . . . 2

Lipid Class Descriptions 3
1) CAR[M+H]+ / Lipid identification . . . 3
1) CAR[M+H]+ / Lipid quantification . . . 3
2) DG[M+NH4]+ / Lipid identification . . . 3
2) DG[M+NH4]+ / Lipid quantification . . . 3
3) TG[M+NH4]+ / Lipid identification . . . 3
3) TG[M+NH4]+ / Lipid quantification . . . 4
4) LPC[M+H]+ / Lipid identification . . . 4
4) LPC[M+H]+ / Lipid quantification . . . 4
5) LPE[M-H]- / Lipid identification . . . 4
5) LPE[M-H]- / Lipid quantification . . . 5
6) PC[M+H]+ / Lipid identification . . . 5
6) PC[M+H]+ / Lipid quantification . . . 6
7) PE[M-H]- / Lipid identification . . . 6
7) PE[M-H]- / Lipid quantification . . . 6
8) PI[M-H]- / Lipid identification . . . 6
8) PI[M-H]- / Lipid quantification . . . 7
9) PS[M-H]- / Lipid identification . . . 7
9) PS[M-H]- / Lipid quantification . . . 7
10) PG[M-H]- / Lipid identification . . . 7
10) PG[M-H]- / Lipid quantification . . . 8
11) PC O[M+H]+ / Lipid identification . . . 8
11) PC O[M+H]+ / Lipid quantification . . . 9
12) PE O[M-H]- / Lipid identification . . . 9
12) PE O[M-H]- / Lipid quantification . . . 9
13) Cer[M+H]+ / Lipid identification . . . 9
13) Cer[M+H]+ / Lipid quantification . . . 10
14) SM[M+H]+ / Lipid identification . . . 10
14) SM[M+H]+ / Lipid quantification . . . 10
15) HexCer[M+H]+ / Lipid identification . . . 10
15) HexCer[M+H]+ / Lipid quantification . . . 10
16) LacCer[M+H]+ / Lipid identification . . . 11
16) LacCer[M+H]+ / Lipid quantification . . . 11
17) PC P[M+H]+ / Lipid identification . . . 11
17) PC P[M+H]+ / Lipid quantification . . . 11
18) PE P[M-H]- / Lipid identification . . . 11
18) PE P[M-H]- / Lipid quantification . . . 12

Separation Workflow

Overall study design

Title of the study	Selective Editing and Functionalization of Mamalian Lipidome		
Document creation date	04/23/2026	Principal investigator	Johannes Morstein
Institution	California Institute of Technology	Corresponding Email	morstein@caltech.edu
Is the workflow targeted or untargeted?	Untargeted	Clinical	No

Lipid extraction

bioRxiv preprint doi: https://doi.org/10.64898/2026.04.24.720406; this version posted April 27, 2026. The copyright holder for this preprint (which was not certified by peer review) is the author/funder, who has granted bioRxiv a license to display the preprint in perpetuity. It is made available under aCC-BY 4.0 International license.

Extraction method	2-phase system	pH adjustment	None
2-phase system	MTBE	Special conditions	None
Derivatization	None	Were internal standards added prior extraction?	Yes

Analytical platform

Ionizaton additives	Ammonium formate, Formic acid	Number of separation dimensions	One dimension
Separation type 1	LC	Separation mode 1 (liquid)	RP
Detector	Mass spectrometer	MS type	Orbitrap
MS vendor	Thermo	Ion source	ESI
MS Level	MS^1, MS^2	Mass resolution for detected ion at MS^1	High resolution
Resolution at m/z 200 at MS^1	120000	Mass accuracy in ppm at MS^1	5
Recording mode of raw data at MS^1	Profile mode	Mass window for precursor ion isolation (in Da total isolation window)	1
Mass resolution for detected ion at MS^2	High resolution	Resolution at m/z 200 at MS^2	15000
Mass accuracy in ppm at MS^2	5	Recording mode of raw data at MS^2	Centroid mode
Was/Were additional dimension/techniques used	No		

Quality control

Blanks	Yes	Type of Blanks	Solvent blank
Quality control	No		

Method qualification and validation

Method validation	No		

Reporting

Are reported raw data uploaded into repository?	Available on request	Are metadata available?	Available on request
Raw data upload	Available on request		

Sample Descriptions

epithelial cells / Human / Cells

Storage and collection conditions	Available	Provided preanalytical information	Time to separate plasma/serum (min), Storage time (month), Freeze-thaw cycles, Preservation method
Temperature handling original sample	4-8 °C	Instant sample preparation	Yes
Storage temperature	-80 °C	Storage time (month)	0
Freeze-thaw cycles	0	Additives	None
Were samples stored under inert gas?	No	Additional preservation methods	No
Biobank samples	No		

bioRxiv preprint doi: https://doi.org/10.64898/2026.04.24.720406; this version posted April 27, 2026. The copyright holder for this preprint (which was not certified by peer review) is the author/funder, who has granted bioRxiv a license to display the preprint in perpetuity. It is made available under aCC-BY 4.0 International license.

Lipid Class Descriptions

1) CAR[M+H]+ / Lipid identification

Lipid class	CAR	MS Level for identification	MS^1
Identification level	Species level	MS^1 adduct	[M+H]+
Isotope correction at MS^1	No	MS^1 verified by standard	Yes
Background check at MS^1	Yes	Did you presume assumptions for identification?	Yes
Which assumptions were presumed?	only common fatty acyl chains included	Check on:	Isobaric overlap
Limit of detection	Signal threshold	RT verified by standard	Yes
Separation of isobaric/isomeric interferece confirmed	No	Model for separation prediction	No
Lipid Identification Software	Compound Discoverer	Nomenclature for intact lipid molecule	Yes

1) CAR[M+H]+ / Lipid quantification

Quantitative	No	Normalization to reference	No
Batch correction	No		

2) DG[M+NH4]+ / Lipid identification

Lipid class	DG	MS Level for identification	MS^1
Identification level	Species level	MS^1 adduct	[M+NH4]+
Isotope correction at MS^1	No	MS^1 verified by standard	Yes
Background check at MS^1	Yes	Did you presume assumptions for identification?	Yes
Which assumptions were presumed?	only common fatty acyl chains included	Check on:	Isobaric overlap
Limit of detection	Signal threshold	RT verified by standard	Yes
Separation of isobaric/isomeric interferece confirmed	No	Model for separation prediction	No
Lipid Identification Software	Compound Discoverer	Nomenclature for intact lipid molecule	Yes

2) DG[M+NH4]+ / Lipid quantification

Quantitative	No	Normalization to reference	No
Batch correction	No		

3) TG[M+NH4]+ / Lipid identification

Lipid class	TG	MS Level for identification	MS^1

bioRxiv preprint doi: https://doi.org/10.64898/2026.04.24.720406; this version posted April 27, 2026. The copyright holder for this preprint (which was not certified by peer review) is the author/funder, who has granted bioRxiv a license to display the preprint in perpetuity. It is made available under aCC-BY 4.0 International license.

Identification level	Species level	MS^1 adduct	[M+NH4]+
Isotope correction at MS^1	No	MS^1 verified by standard	Yes
Background check at MS^1	Yes	Did you presume assumptions for identification?	Yes
Which assumptions were presumed?	only common fatty acyl chains included	Check on:	Isobaric overlap
Limit of detection	Signal threshold	RT verified by standard	Yes
Separation of isobaric/isomeric interferece confirmed	No	Model for separation prediction	No
Lipid Identification Software	Compound Discoverer	Nomenclature for intact lipid molecule	No

3) TG[M+NH4]+ / Lipid quantification

Quantitative	No	Normalization to reference	No
Batch correction	No		

4) LPC[M+H]+ / Lipid identification

Lipid class	LPC	MS Level for identification	MS^1, MS^2
Identification level	Molecular species level	MS^1 adduct	[M+H]+
Isotope correction at MS^1	No	MS^2 adduct	[M+H]+

Fragments for identification

Fragment name
-HG(PC,183)
HG(PC,184)
Loss of CH3 and formate from precursor ion

Isotope correction at MS^2	No	MS^1 verified by standard	Yes
MS^2 verified by standard	Yes	Background check at MS^1	Yes
Background check at MS^2	Yes	Did you presume assumptions for identification?	Yes
Which assumptions were presumed?	only common fatty acyl chains included	Check on:	Isobaric overlap
Limit of detection	Signal threshold	RT verified by standard	Yes
Separation of isobaric/isomeric interferece confirmed	No	Model for separation prediction	No
Lipid Identification Software	Compound Discoverer	Nomenclature for intact lipid molecule	Yes
Nomenclature for fragment ions	N/A		

4) LPC[M+H]+ / Lipid quantification

Quantitative	No	Normalization to reference	No
Batch correction	No		

5) LPE[M-H]- / Lipid identification

bioRxiv preprint doi: https://doi.org/10.64898/2026.04.24.720406; this version posted April 27, 2026. The copyright holder for this preprint (which was not certified by peer review) is the author/funder, who has granted bioRxiv a license to display the preprint in perpetuity. It is made available under aCC-BY 4.0 International license.

Lipid class	LPE	MS Level for identification	MS^1, MS^2
Identification level	Molecular species level	MS^1 adduct	[M-H]-
Isotope correction at MS^1	No	MS^2 adduct	[M-H]-

Fragments for identification

Fragment name
GP(153)
FA1(+O)

Isotope correction at MS^2	No	MS^1 verified by standard	Yes
MS^2 verified by standard	Yes	Background check at MS^1	Yes
Background check at MS^2	Yes	Did you presume assumptions for identification?	Yes
Which assumptions were presumed?	only common fatty acyl chains included	Limit of detection	Signal threshold
RT verified by standard	Yes	Separation of isobaric/isomeric interferece confirmed	No
Model for separation prediction	No	Lipid Identification Software	Compound Discoverer
Nomenclature for intact lipid molecule	Yes	Nomenclature for fragment ions	N/A

5) LPE[M-H]- / Lipid quantification

Quantitative	No	Normalization to reference	No
Batch correction	No		

6) PC[M+H]+ / Lipid identification

Lipid class	PC	MS Level for identification	MS^1, MS^2
Identification level	Molecular species level	MS^1 adduct	[M+H]+
Isotope correction at MS^1	No	MS^2 adduct	[M+H]+

Fragments for identification

Fragment name
-HG(PC,183)
HG(PC,184)
-FA1(+H)-(H2O)
-FA2(+H)

Isotope correction at MS^2	No	MS^1 verified by standard	Yes
MS^2 verified by standard	Yes	Background check at MS^1	Yes
Background check at MS^2	Yes	Did you presume assumptions for identification?	Yes
Which assumptions were presumed?	only common fatty acyl chains included	Check on:	Isobaric overlap
Limit of detection	Signal threshold	RT verified by standard	Yes
Separation of isobaric/isomeric interferece confirmed	No	Model for separation prediction	No
Lipid Identification Software	Compound Discoverer	Nomenclature for intact lipid molecule	Yes
Nomenclature for fragment ions	N/A		

bioRxiv preprint doi: https://doi.org/10.64898/2026.04.24.720406; this version posted April 27, 2026. The copyright holder for this preprint (which was not certified by peer review) is the author/funder, who has granted bioRxiv a license to display the preprint in perpetuity. It is made available under aCC-BY 4.0 International license.

6) PC[M+H]+ / Lipid quantification

Quantitative	No	Normalization to reference	No
Batch correction	No		

7) PE[M-H]- / Lipid identification

Lipid class	PE	MS Level for identification	MS^1, MS^2
Identification level	Molecular species level	MS^1 adduct	[M-H]-
Isotope correction at MS^1	No	MS^2 adduct	[M-H]-

Fragments for identification

Fragment name
GP(153)
-FA1(-H)
-FA2(-H)
HG(PE,140)

Isotope correction at MS^2	No	MS^1 verified by standard	Yes
MS^2 verified by standard	Yes	Background check at MS^1	Yes
Background check at MS^2	Yes	Did you presume assumptions for identification?	Yes
Which assumptions were presumed?	only common fatty acyl chains included	Check on:	Isobaric overlap
Limit of detection	Signal threshold	RT verified by standard	Yes
Separation of isobaric/isomeric interferece confirmed	No	Model for separation prediction	No
Lipid Identification Software	Compound Discoverer	Nomenclature for intact lipid molecule	Yes
Nomenclature for fragment ions	N/A		

7) PE[M-H]- / Lipid quantification

Quantitative	No	Normalization to reference	No
Batch correction	No		

8) PI[M-H]- / Lipid identification

Lipid class	PI	MS Level for identification	MS^1, MS^2
Identification level	Species level	MS^1 adduct	[M-H]-
Isotope correction at MS^1	No	MS^2 adduct	[M-H]-

Fragments for identification

Fragment name
-FA1(-H)
-FA2(-H)
HG(PI,241)

Isotope correction at MS^2	No	MS^1 verified by standard	Yes
MS^2 verified by standard	Yes	Background check at MS^1	Yes

bioRxiv preprint doi: https://doi.org/10.64898/2026.04.24.720406; this version posted April 27, 2026. The copyright holder for this preprint (which was not certified by peer review) is the author/funder, who has granted bioRxiv a license to display the preprint in perpetuity. It is made available under aCC-BY 4.0 International license.

Background check at MS^2	Yes	Did you presume assumptions for identification?	Yes
Which assumptions were presumed?	only common fatty acyl chains included	Check on:	Isobaric overlap
Limit of detection	Signal threshold	RT verified by standard	Yes
Separation of isobaric/isomeric interferece confirmed	No	Model for separation prediction	No
Lipid Identification Software	Compound Discoverer	Nomenclature for intact lipid molecule	Yes
Nomenclature for fragment ions	N/A		

8) PI[M-H]- / Lipid quantification

Quantitative	No	Normalization to reference	No
Batch correction	No		

9) PS[M-H]- / Lipid identification

Lipid class	PS	MS Level for identification	MS^1, MS^2
Identification level	Molecular species level	MS^1 adduct	[M-H]-
Isotope correction at MS^1	No	MS^2 adduct	[M-H]-

Fragments for identification

Fragment name
-(C3H5NO2,87)
FA1(+O)
FA2(+O)

Isotope correction at MS^2	No	MS^1 verified by standard	Yes
MS^2 verified by standard	Yes	Background check at MS^1	Yes
Background check at MS^2	Yes	Did you presume assumptions for identification?	Yes
Which assumptions were presumed?	only common fatty acyl chains included	Check on:	Isobaric overlap
Limit of detection	Signal threshold	RT verified by standard	Yes
Separation of isobaric/isomeric interferece confirmed	No	Model for separation prediction	No
Lipid Identification Software	Compound Discoverer	Nomenclature for intact lipid molecule	Yes
Nomenclature for fragment ions	N/A		

9) PS[M-H]- / Lipid quantification

Quantitative	No	Normalization to reference	No
Batch correction	No		

10) PG[M-H]- / Lipid identification

bioRxiv preprint doi: https://doi.org/10.64898/2026.04.24.720406; this version posted April 27, 2026. The copyright holder for this preprint (which was not certified by peer review) is the author/funder, who has granted bioRxiv a license to display the preprint in perpetuity. It is made available under aCC-BY 4.0 International license.

Lipid class	PG	MS Level for identification	MS^1, MS^2
Identification level	Molecular species level	MS^1 adduct	[M-H]-
Isotope correction at MS^1	No	MS^2 adduct	[M-H]-

Fragments for identification

Fragment name
FA1(+O)
FA2(+O)
HG(PG,171)
GP(153)

Isotope correction at MS^2	No	MS^1 verified by standard	Yes
MS^2 verified by standard	Yes	Background check at MS^1	Yes
Background check at MS^2	Yes	Did you presume assumptions for identification?	Yes
Which assumptions were presumed?	only common fatty acyl chains included	Check on:	Isobaric overlap
Limit of detection	Signal threshold	RT verified by standard	Yes
Separation of isobaric/isomeric interferece confirmed	No	Model for separation prediction	No
Lipid Identification Software	Compound Discoverer	Nomenclature for intact lipid molecule	Yes
Nomenclature for fragment ions	N/A		

10) PG[M-H]- / Lipid quantification

Quantitative	No	Normalization to reference	No
Batch correction	No		

11) PC O[M+H]+ / Lipid identification

Lipid class	PC O	MS Level for identification	MS^1, MS^2
Identification level	Molecular species level	MS^1 adduct	[M+H]+
Isotope correction at MS^1	No	MS^2 adduct	[M+H]+

Fragments for identification

Fragment name
HG(PC,184)

Isotope correction at MS^2	No	MS^1 verified by standard	No
MS^2 verified by standard	No	Background check at MS^1	Yes
Background check at MS^2	Yes	Did you presume assumptions for identification?	Yes
Which assumptions were presumed?	only common fatty acyl chains included	Check on:	Isobaric overlap
Limit of detection	Signal threshold	RT verified by standard	No
Separation of isobaric/isomeric interferece confirmed	No	Model for separation prediction	No
Lipid Identification Software	Compound Discoverer	Nomenclature for intact lipid molecule	Yes
Nomenclature for fragment ions	N/A		

bioRxiv preprint doi: https://doi.org/10.64898/2026.04.24.720406; this version posted April 27, 2026. The copyright holder for this preprint (which was not certified by peer review) is the author/funder, who has granted bioRxiv a license to display the preprint in perpetuity. It is made available under aCC-BY 4.0 International license.

11) PC O[M+H]+ / Lipid quantification

Quantitative	No	Normalization to reference	No
Batch correction	No		

12) PE O[M-H]- / Lipid identification

Lipid class	PE O	MS Level for identification	MS^1, MS^2
Identification level	Molecular species level	MS^1 adduct	[M-H]-
Isotope correction at MS^1	No	MS^2 adduct	[M-H]-
Fragments for identification			

Fragment name
FA2(+O)
GP(153)

Isotope correction at MS^2	No	MS^1 verified by standard	No
MS^2 verified by standard	No	Background check at MS^1	Yes
Background check at MS^2	Yes	Did you presume assumptions for identification?	Yes
Which assumptions were presumed?	only common fatty acyl chains included	Check on:	Isobaric overlap
Limit of detection	Signal threshold	RT verified by standard	No
Separation of isobaric/isomeric interferece confirmed	No	Model for separation prediction	No
Lipid Identification Software	Compound Discoverer	Nomenclature for intact lipid molecule	Yes
Nomenclature for fragment ions	N/A		

12) PE O[M-H]- / Lipid quantification

Quantitative	No	Normalization to reference	No
Batch correction	No		

13) Cer[M+H]+ / Lipid identification

Lipid class	Cer	MS Level for identification	MS^1
Identification level	Species level	MS^1 adduct	[M+H]+
Isotope correction at MS^1	No	MS^1 verified by standard	Yes
Background check at MS^1	Yes	Did you presume assumptions for identification?	Yes
Which assumptions were presumed?	only common fatty acyl chains included	Check on:	Isobaric overlap
Limit of detection	Signal threshold	RT verified by standard	Yes
Separation of isobaric/isomeric interferece confirmed	No	Model for separation prediction	No
Lipid Identification Software	Compound Discoverer	Nomenclature for intact lipid molecule	Yes

bioRxiv preprint doi: https://doi.org/10.64898/2026.04.24.720406; this version posted April 27, 2026. The copyright holder for this preprint (which was not certified by peer review) is the author/funder, who has granted bioRxiv a license to display the preprint in perpetuity. It is made available under aCC-BY 4.0 International license.

13) Cer[M+H]+ / Lipid quantification

Quantitative	No	Normalization to reference	No
Batch correction	No		

14) SM[M+H]+ / Lipid identification

Lipid class	SM	MS Level for identification	MS^1
Identification level	Species level	MS^1 adduct	[M+H]+
Isotope correction at MS^1	No	MS^1 verified by standard	Yes
Background check at MS^1	Yes	Did you presume assumptions for identification?	Yes
Which assumptions were presumed?	only common fatty acyl chains included	Check on:	Isobaric overlap
Limit of detection	Signal threshold	RT verified by standard	Yes
Separation of isobaric/isomeric interferece confirmed	No	Model for separation prediction	No
Lipid Identification Software	Compound Discoverer	Nomenclature for intact lipid molecule	Yes

14) SM[M+H]+ / Lipid quantification

Quantitative	No	Normalization to reference	No
Batch correction	No		

15) HexCer[M+H]+ / Lipid identification

Lipid class	HexCer	MS Level for identification	MS^1
Identification level	Species level	MS^1 adduct	[M+H]+
Isotope correction at MS^1	No	MS^1 verified by standard	No
Background check at MS^1	Yes	Did you presume assumptions for identification?	Yes
Which assumptions were presumed?	only common fatty acyl chains included	Check on:	Isobaric overlap
Limit of detection	Signal threshold	RT verified by standard	No
Separation of isobaric/isomeric interferece confirmed	No	Model for separation prediction	No
Lipid Identification Software	Compound Discoverer	Nomenclature for intact lipid molecule	Yes

15) HexCer[M+H]+ / Lipid quantification

Quantitative	No	Normalization to reference	No
Batch correction	No		

bioRxiv preprint doi: https://doi.org/10.64898/2026.04.24.720406; this version posted April 27, 2026. The copyright holder for this preprint (which was not certified by peer review) is the author/funder, who has granted bioRxiv a license to display the preprint in perpetuity. It is made available under aCC-BY 4.0 International license.

16) LacCer[M+H]+ / Lipid identification

Lipid class	LacCer	MS Level for identification	MS^1
Identification level	Species level	MS^1 adduct	[M+H]+
Isotope correction at MS^1	No	MS^1 verified by standard	Yes
Background check at MS^1	Yes	Did you presume assumptions for identification?	Yes
Which assumptions were presumed?	only common fatty acyl chains included	Check on:	Isobaric overlap
Limit of detection	Signal threshold	RT verified by standard	No
Separation of isobaric/isomeric interferece confirmed	No	Model for separation prediction	No
Lipid Identification Software	Compound Discoverer	Nomenclature for intact lipid molecule	Yes

16) LacCer[M+H]+ / Lipid quantification

Quantitative	No	Normalization to reference	No
Batch correction	No		

17) PC P[M+H]+ / Lipid identification

Lipid class	PC P	MS Level for identification	MS^1
Identification level	Species level	MS^1 adduct	[M+H]+
Isotope correction at MS^1	No	MS^1 verified by standard	No
Background check at MS^1	No	Did you presume assumptions for identification?	Yes
Which assumptions were presumed?	only common fatty acyl chains included	Check on:	Isobaric overlap
Limit of detection	Signal threshold	RT verified by standard	No
Separation of isobaric/isomeric interferece confirmed	No	Model for separation prediction	No
Lipid Identification Software	Compound Discoverer	Nomenclature for intact lipid molecule	Yes

17) PC P[M+H]+ / Lipid quantification

Quantitative	No	Normalization to reference	No
Batch correction	No		

18) PE P[M-H]- / Lipid identification

Lipid class	PE P	MS Level for identification	MS^1
Identification level	Species level	MS^1 adduct	[M-H]-
Isotope correction at MS^1	No	MS^1 verified by standard	No
Background check at MS^1	No	Did you presume assumptions for identification?	Yes

bioRxiv preprint doi: https://doi.org/10.64898/2026.04.24.720406; this version posted April 27, 2026. The copyright holder for this preprint (which was not certified by peer review) is the author/funder, who has granted bioRxiv a license to display the preprint in perpetuity. It is made available under aCC-BY 4.0 International license.

Which assumptions were presumed?	only common fatty acyl chains included	Check on:	Isobaric overlap
Limit of detection	Signal threshold	RT verified by standard	No
Separation of isobaric/isomeric interferece confirmed	No	Model for separation prediction	No
Lipid Identification Software	Compound Discoverer	Nomenclature for intact lipid molecule	Yes

18) PE P[M-H]- / Lipid quantification

Quantitative	No	Normalization to reference	No
Batch correction	No		

bioRxiv preprint doi: https://doi.org/10.64898/2026.04.24.720406; this version posted April 27, 2026. The copyright holder for this preprint (which was not certified by peer review) is the author/funder, who has granted bioRxiv a license to display the preprint in perpetuity. It is made available under aCC-BY 4.0 International license.

Chemical Synthesis Materials and Methods

Contents

π-Fatty Acids 2

Alkyl Glycerol Probes 18

π-Sphingosines 27

Azido-Stearic Acids 39

NMR Data 56

bioRxiv preprint doi: https://doi.org/10.64898/2026.04.24.720406; this version posted April 27, 2026. The copyright holder for this preprint (which was not certified by peer review) is the author/funder, who has granted bioRxiv a license to display the preprint in perpetuity. It is made available under aCC-BY 4.0 International license.

π-Fatty Acids

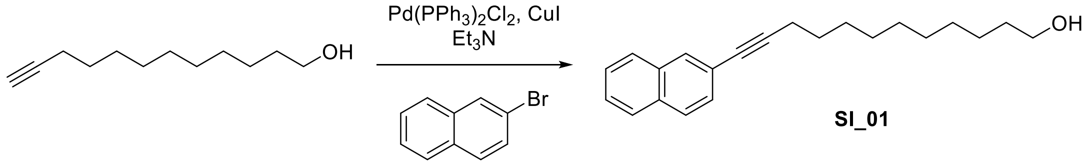

SI_01

12-(naphthalen-2-yl)dodec-11-yn-1-ol (SI_01): 2-Bromonaphthalene (5.01 g, 24.2 mmol, 1.00 equiv) was dissolved in 30.0 mL triethylamine, followed by the addition of dodec-11-yn-1-ol (6.60 g, 36.2 mmol, 1.50 equiv), $Pd(PPh_3)_2Cl_2$ (850 mg, 1.21 mmol, 0.05 equiv) and CuI (460 mg, 2.42 mmol, 0.10 equiv) under nitrogen atmosphere. The reaction mixture was heated to 80 °C and stirred for 2 h, then allowed to cool to ambient temperature. The reaction mixture was diluted with water and extracted three times with ethyl acetate. The combined organic layers were washed with brine, dried over Na_2SO_4, filtered and concentrated under reduced pressure. The residue was purified by silica gel chromatography (hexanes/ethyl acetate, 0-5%) to afford the title compound as a clear oil (7.38 g, 24.2 mmol, 99% yield).

LCMS *m/z* calculated for $C_{22}H_{28}O^+$ ($[M+H]^+$): 309.2 found: 309.2.

bioRxiv preprint doi: https://doi.org/10.64898/2026.04.24.720406; this version posted April 27, 2026. The copyright holder for this preprint (which was not certified by peer review) is the author/funder, who has granted bioRxiv a license to display the preprint in perpetuity. It is made available under aCC-BY 4.0 International license.

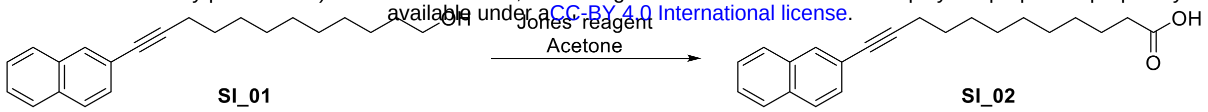

12-(naphthalen-2-yl)dodec-11-ynoic acid (SI_02): 12-(Naphthalen-2-yl)dodec-11-yn-1-ol (**SI_01**, 1.00 g, 3.24 mmol, 1.00 equiv) was dissolved in 10.0 mL acetone and cooled to 0 °C. Jones' reagent (3.24 mL, 6.48 mmol, 2.00 equiv) was added, and the cooling bath was removed and the mixture allowed to warm to ambient temperature and stirred for 2 h. The reaction mixture was diluted with water and extracted with three times with ethyl acetate. The combined organic layers were washed with brine, dried over Na_2SO_4, filtered and concentrated under reduced pressure. The residue was purified by silica gel chromatography (hexanes/EtOAc, 5-20%) to afford the title compound **SI_02** (400 mg, 1.24 mmol, 38% yield).

LCMS *m/z* calculated for $C_{22}H_{32}O^+$ ($[M+H]^+$): 323.2 found: 323.2.

bioRxiv preprint doi: https://doi.org/10.64898/2026.04.24.720406; this version posted April 27, 2026. The copyright holder for this preprint (which was not certified by peer review) is the author/funder, who has granted bioRxiv a license to display the preprint in perpetuity. It is made available under aCC-BY 4.0 International license.

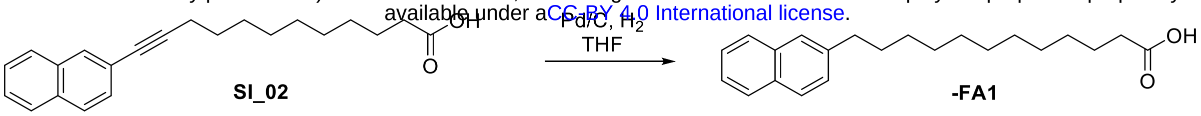

π-FA1: A mixture of 12-(naphthalen-2-yl)dodec-11-ynoic acid (**SI_02**, 330 mg, 1.02 mmol, 1.00 equiv) and 10% palladium on carbon (218 mg, 0.24 mmol, 0.24 equiv) in 5.0 mL tetrahydrofuran was stirred under hydrogen atmosphere (50 psi) overnight. The reaction mixture was filtered through a celite pad and the filtrate was evaporated to dryness. The residue was purified by C18 column chromatography (MeCN/water, 0-100%, with 0.1% formic acid) to afford **π-FA1** as a white solid (220 mg, 0.67 mmol, 66% yield).

^{1}H NMR (400 MHz, $CDCl_3$) δ 10.33 (bs, 1H) 7.81 – 7.75 (m, 3H), 7.60 (s, 1H), 7.48 – 7.37 (m, 2H), 7.33 (dd, *J* = 8.4, 1.6 Hz, 1H), 2.82 – 2.71 (t, *J* = 7.6 Hz, 2H), 2.34 (t, *J* = 7.6 Hz, 2H), 1.73-1.58 (m, 4H), 1.40 – 1.26 (m, 14H).

^{13}C NMR (101 MHz, $CDCl_3$) δ 179.6, 140.6, 133.8, 132.1, 127.8, 127.7, 127.6, 127.5, 126.4, 125.9, 125.1, 36.3, 34.1, 31.5, 29.70, 29.68, 29.65, 29.55, 29.47, 29.4, 29.2, 24.8.

LCMS *m/z* calculated for $C_{22}H29O_2^-$ ([M-H]$^-$): 327.2 found: 327.2.

bioRxiv preprint doi: https://doi.org/10.64898/2026.04.24.720406; this version posted April 27, 2026. The copyright holder for this preprint (which was not certified by peer review) is the author/funder, who has granted bioRxiv a license to display the preprint in perpetuity. It is made available under aCC-BY 4.0 International license.

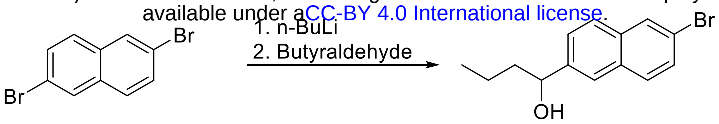

SI_03

1-(6-bromonaphthalen-2-yl)butan-1-ol (SI_03): To a solution of 2,6-dibromonaphthalene (5.00 g, 17.5 mmol, 1.00 equiv) in 20.0 mL THF was added dropwise n-butyllithium (7.6 mL, 19.2 mmol, 1.10 equiv) at –78 °C. The reaction mixture was stirred at –78 °C for 30 min, then butyraldehyde (1.26 g, 17.5 mmol, 1.00 equiv) was added and the solution was allowed to warm up to room temperature and stirred for another 16 h. The reaction was quenched with saturated NH_4Cl and extracted three times with EtOAc. The combined organic layers were washed with brine, dried over Na_2SO_4, filtered and concentrated under reduced pressure. The residue was purified by silica gel chromatography (hexanes/EtOAc, 0 - 20%) to afford the title compound **SI_03** as a yellow solid (2.4 g, 8.6 mmol, 49%).

bioRxiv preprint doi: https://doi.org/10.64898/2026.04.24.720406; this version posted April 27, 2026. The copyright holder for this preprint (which was not certified by peer review) is the author/funder, who has granted bioRxiv a license to display the preprint in perpetuity. It is made available under aCC-BY 4.0 International license.

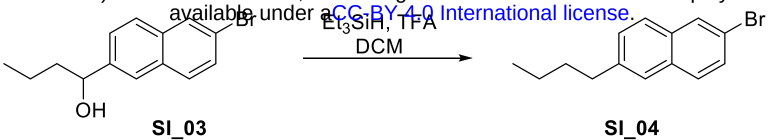

SI_03 **SI_04**

2-Bromo-6-butylnaphthalene (SI_04): To a solution of 1-(6-bromonaphthalen-2-yl)butan-1-ol (**SI_03**, 4.93 g, 17.7 mmol) in 20.0 mL dichloromethane, was added triethylsilane (10.3 g, 88.3 mmol, 5.00 equiv) and trifluoroacetic acid (10.1 g, 88.3 mmol, 5.00 equiv). The mixture was stirred at room temperature for 4 h. The reaction quenched by the addition of water and the resulting mixture extracted three times with ethyl acetate. The combined organic layers were washed with brine, dried over Na_2SO_4, filtered and concentrated under reduced pressure. The residue was purified by silica gel chromatography (hexanes/EtOAc, 0-5%) to afford the title compound **SI_04** as a white solid (2.4 g, 9.1 mmol, 52% yield).

bioRxiv preprint doi: https://doi.org/10.64898/2026.04.24.720406; this version posted April 27, 2026. The copyright holder for this preprint (which was not certified by peer review) is the author/funder, who has granted bioRxiv a license to display the preprint in perpetuity. It is made available under aCC-BY 4.0 International license.

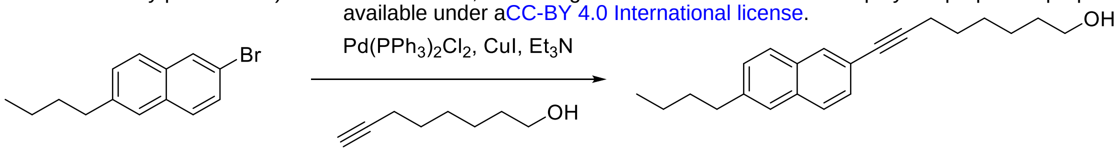

SI_04 SI_05

8-(6-Butylnaphthalen-2-yl)oct-7-yn-1-ol (SI_05): To a mixture of 2-bromo-6-butylnaphthalene (**SI_04**, 1.7 g, 6.46 mmol, 1.00 equiv) in 17.0 mL triethylamine were added oct-7-yn-1-ol (1.22 g, 9.69 mmol, 1.50 equiv), $Pd(PPh_3)_2Cl_2$ (0.23 g, 0.32 mmol, 0.05 equiv) and CuI (0.12 g, 0.65 mmol, 0.10 equiv). The reaction mixture was stirred for 16 h at 80 °C under nitrogen atmosphere. The reaction quenched by the addition of water and the resulting mixture extracted three times with ethyl acetate. The combined organic layers were washed with brine, dried over Na_2SO_4, filtered and concentrated under reduced pressure. The residue was purified by silica gel chromatography (hexanes/EtOAc, 0-15%) to afford the title compound **SI_05** as a yellow solid (1.75 g, 5.67 mmol, 88% yield).

LCMS *m/z* calculated for $C_{22}H_{29}O^+$ ($[M+H]^+$): 309.21 found: 309.2.

bioRxiv preprint doi: https://doi.org/10.64898/2026.04.24.720406; this version posted April 27, 2026. The copyright holder for this preprint (which was not certified by peer review) is the author/funder, who has granted bioRxiv a license to display the preprint in perpetuity. It is made available under aCC-BY 4.0 International license.

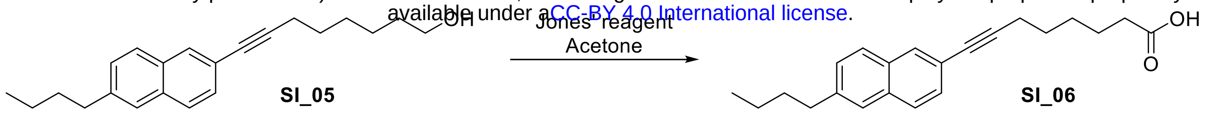

8-(6-Butylnaphthalen-2-yl)oct-7-ynoic acid (SI_06): To a mixture of 8-(6-butylnaphthalen-2-yl)oct-7-yn-1-ol (**SI_05**, 1.30 g, 4.21 mmol, 1.00 equiv) in 3.0 mL acetone at 0 °C was added Jones' reagent (1.00 g, 5.05 mmol, 1.20 equiv) at 0°C. The mixture was stirred at 25 °C for 2 h. The reaction quenched by the addition of water and the ensuing mixture extracted twice with ethyl acetate. The combined organic layers were washed with brine, dried over Na_2SO_4, filtered and concentrated under reduced pressure. The residue was purified by silica gel chromatography (hexanes/EtOAc, 0-35%) to afford the title compound **SI_06** as a yellow solid (1.20, 3.72 mmol, 88%).

LCMS *m/z* calculated for $C_{22}H_{25}O_2{}^-$ ($[M-H]^-$): 321.19 found: 321.0.

bioRxiv preprint doi: https://doi.org/10.64898/2026.04.24.720406; this version posted April 27, 2026. The copyright holder for this preprint (which was not certified by peer review) is the author/funder, who has granted bioRxiv a license to display the preprint in perpetuity. It is made available under aCC-BY 4.0 International license.

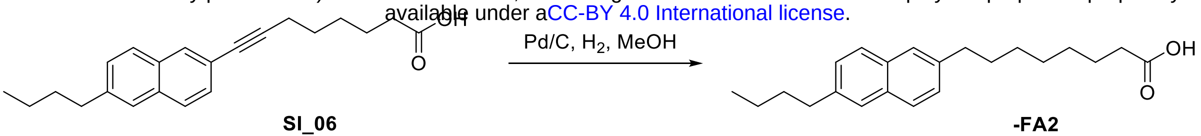

π-FA2: To a solution of 8-(6-butylnaphthalen-2-yl)oct-7-ynoic acid (**SI_06**, 400 mg, 1.24 mmol) in 10.0 mL methanol was added 10% palladium on carbon (264 mg, 0.248 mmol) and the reaction stirred under H_2 atmosphere (50 psi) overnight. The reaction mixture was then filtered through a celite pad and the filtrate was evaporated to dryness. The residue was purified by C18 column chromatography (MeCN/water, 0-75%, with 0.1% formic acid) to afford **π-FA2** as a white solid (230 mg, 0.703 mmol, 57% yield).

^{1}H NMR (400 MHz, DMSO-d_6) δ 11.95 (s, 1H), 7.74 (d, J = 8.8 Hz, 2H), 7.61 (s, 2H), 7.33 (d, J = 8.4 Hz, 2H), 2.72-2.67 (m, 4H), 2.18 (t, J = 7.2 Hz, 2H), 1.69 – 1.58 (m, 4H), 1.53 – 1.43 (m, 2H), 1.40 – 1.21 (m, 8H), 0.91 (t, J = 7.2 Hz, 3H).

^{13}C NMR (101 MHz, DMSO-d_6) δ 174.5, 139.02, 139.00, 131.7, 127.3, 127.1, 125.7, 35.2, 34.9, 33.7, 33.0, 30.8, 28.58, 28.56, 28.51, 24.48, 21.8, 13.8

Note: Four resonances corresponding to the naphthalene fragment are not visible in the ^{13}C NMR due to overlap.

LCMS *m/z* calculated for $C_{22}H_{29}O_2^-$ ([M-H]$^-$): 325.22 found: 325.1.

bioRxiv preprint doi: https://doi.org/10.64898/2026.04.24.720406; this version posted April 27, 2026. The copyright holder for this preprint (which was not certified by peer review) is the author/funder, who has granted bioRxiv a license to display the preprint in perpetuity. It is made available under aCC-BY 4.0 International license.

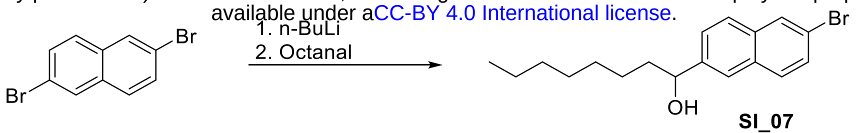

1-(6-Bromonaphthalen-2-yl)octan-1-ol (SI_07): To a solution of 2,6-dibromonaphthalene (10.0 g, 35.0 mmol, 1.00 equiv) in 100.0 mL THF was added dropwise n-butyllithium (15.3 mL, 38.4 mmol, 1.10 equiv, 2.50 M) at –78 °C. The reaction mixture was stirred at –78 °C for 50 min, then octanal (4.48 g, 35.0 mmol, 1.00 equiv) was added and the solution was allowed to warm up to room temperature and stirred for another 16 h. The reaction was quenched with saturated NH_4Cl and extracted three times with EtOAc. The combined organic layers were washed with brine, dried over Na_2SO_4, filtered and concentrated under reduced pressure. The residue was purified by silica gel chromatography (hexanes/EtOAc, 0 - 20%) to afford the title compound **SI_07** as a yellow solid (5.29 g, 15.8 mmol, 49%).

bioRxiv preprint doi: https://doi.org/10.64898/2026.04.24.720406; this version posted April 27, 2026. The copyright holder for this preprint (which was not certified by peer review) is the author/funder, who has granted bioRxiv a license to display the preprint in perpetuity. It is made available under aCC-BY 4.0 International license.

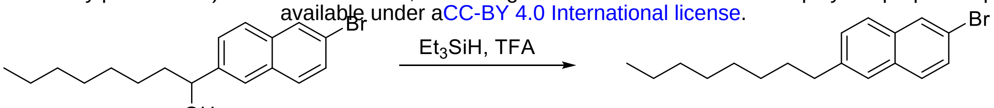

2-Bromo-6-octylnaphthalene (SI_08): To a solution of 1-(6-bromonaphthalen-2-yl)octan-1-ol (**SI_07**, 5.29 g, 15.8 mmol, 1.00 equiv) in 53.0 mL dichloromethane was added triethylsilane (9.20 g, 79.1 mmol, 5.00 equiv) and trifluoroacetic acid (9.01 g, 79.1 mmol, 5.00 equiv). The mixture was stirred at room temperature for 16 h. The reaction quenched by the addition of water and the resulting mixture extracted three times with ethyl acetate. The combined organic layers were washed with brine, dried over Na_2SO_4, filtered and concentrated under reduced pressure. The residue was purified by silica gel chromatography (hexanes/EtOAc, 0-5%) to afford the title compound **SI_08** as a white solid (4.58 g, 14.4 mmol, 91% yield).

bioRxiv preprint doi: https://doi.org/10.64898/2026.04.24.720406; this version posted April 27, 2026. The copyright holder for this preprint (which was not certified by peer review) is the author/funder, who has granted bioRxiv a license to display the preprint in perpetuity. It is made available under aCC-BY 4.0 International license.

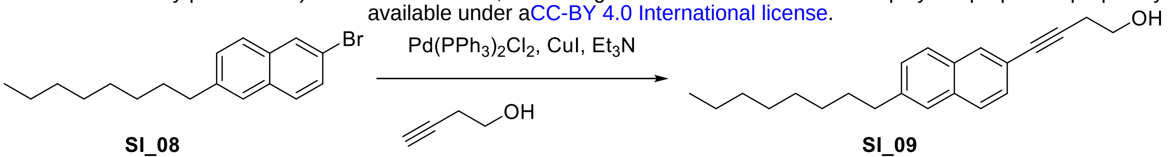

4-(6-Octylnaphthalen-2-yl)but-3-yn-1-ol (SI_09): To a mixture of 2-bromo-6-octylnaphthalene (**SI_08**, 1.9 g, 5.95 mmol, 1.00 equiv) in 10.0 mL triethylamine were added but-3-yn-1-ol (0.63 g, 8.93 mmol, 1.50 equiv), $Pd(PPh_3)_2Cl_2$ (0.21 g, 0.30 mmol, 0.05 equiv) and CuI (0.11 g, 0.65 mmol, 0.10 equiv). The reaction mixture was stirred for 2 h at 80 °C under nitrogen atmosphere. The reaction quenched by the addition of water and the resulting mixture extracted three times with ethyl acetate. The combined organic layers were washed with brine, dried over Na_2SO_4, filtered and concentrated under reduced pressure. The residue was purified by silica gel chromatography (hexanes/EtOAc, 0-15%) to afford the title compound **SI_09** as a clear oil (1.1 g, 3.56 mmol, 60% yield).

LCMS *m/z* calculated. for $C_{22}H_{28}O^+$ ($[M+H]^+$): 309.2 found: 309.2.

bioRxiv preprint doi: https://doi.org/10.64898/2026.04.24.720406; this version posted April 27, 2026. The copyright holder for this preprint (which was not certified by peer review) is the author/funder, who has granted bioRxiv a license to display the preprint in perpetuity. It is made available under aCC-BY 4.0 International license.

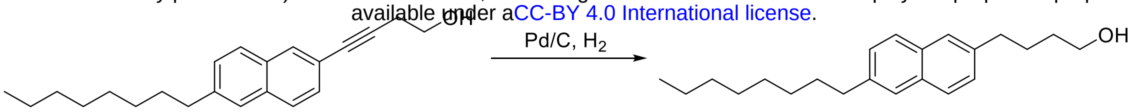

SI_09 **SI_10**

4-(6-Octylnaphthalen-2-yl)butan-1-ol SI_10: To a solution of 4-(6-octylnaphthalen-2-yl)but-3-yn-1-ol (**SI_09**, 1.00 g, 3.24 mmol, 1.00 equiv) in 20.0 mL THF was added 10% palladium on carbon (345 mg, 3.24 mmol, 1.00 equiv) and the reaction stirred under H_2 atmosphere (50 psi) overnight. The reaction mixture was then filtered through a celite pad and the filtrate was evaporated to dryness. The residue was used in the next step without further purification.

LCMS *m/z* calculated. for $C_{22}H_{32}O^+$ ($[M+H]^+$): 313.2 found: 313.3.

bioRxiv preprint doi: https://doi.org/10.64898/2026.04.24.720406; this version posted April 27, 2026. The copyright holder for this preprint (which was not certified by peer review) is the author/funder, who has granted bioRxiv a license to display the preprint in perpetuity. It is made available under aCC-BY 4.0 International license.

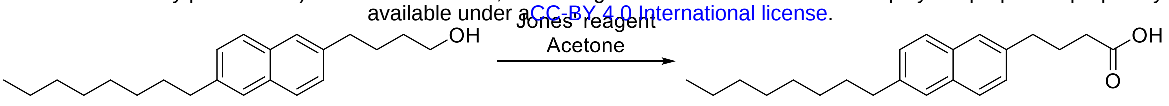

SI_10 **-FA3**

π-FA3: To a mixture of 4-(6-octylnaphthalen-2-yl)butan-1-ol (**SI_10**, 600 mg, 1.92 mmol, 1.00 equiv) in 10.0 mL acetone at 0 °C was added Jones' reagent (1.2 mL, 2.50 mmol, 1.30 equiv) at 0℃. The mixture was stirred at 25 °C for 2 h. The reaction quenched by the addition of water and the ensuing mixture extracted twice with ethyl acetate. The combined organic layers were washed with brine, dried over Na_2SO_4, filtered and concentrated under reduced pressure. The residue was purified by silica gel chromatography (hexanes/EtOAc, 0-55%) to afford the title compound **π-FA3** as a yellow solid (1.20, 3.72 mmol, 88%).

^{1}H NMR (400 MHz, $CDCl_3$) δ 7.70 (m, 2H), 7.57 (s, 2H), 7.32–7.28 (m, 2H), 2.82 (m, 2H), 2.74(t, J = 7.6 Hz, 2H), 2.40 (t, J = 7.4 Hz, 2H), 2.12 – 1.99 (m, 2H), 1.72– 1.65 (m, 2H), 1.35 – 1.27 (m, 10H), 0.87 (t, J = 6.4 Hz, 3H).

^{13}C NMR (101 MHz, $CDCl_3$) δ 179.7, 140.0, 137.9, 132.4, 132.2, 127.8, 127.7, 127.4, 127.3, 126.5, 126.2, 36.2, 35.2, 33.4, 32.0, 31.6, 29.7, 29.5, 29.4, 26.3, 22.8, 14.3.

LCMS *m/z* calculated for $C_{22}H_{29}O_2^-$ ($[M-H]^-$): 325.2 found: 325.2.

bioRxiv preprint doi: https://doi.org/10.64898/2026.04.24.720406; this version posted April 27, 2026. The copyright holder for this preprint (which was not certified by peer review) is the author/funder, who has granted bioRxiv a license to display the preprint in perpetuity. It is made available under aCC-BY 4.0 International license.

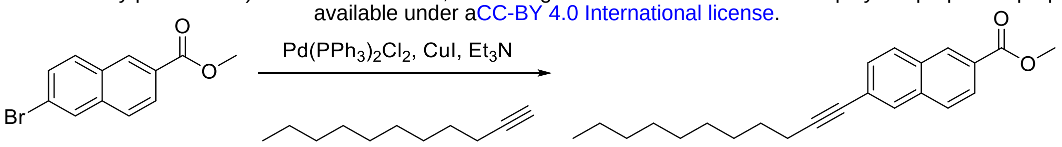

SI_11

Methyl 6-(undec-1-yn-1-yl)-2-naphthoate (SI_11): To a mixture of methyl 6-bromo-2-naphthoate (2.0 g, 7.54 mmol, 1.00 equiv) in 15.0 mL triethylamine were added undec-1-yne (1.72 g, 11.3 mmol, 1.50 equiv), $Pd(PPh_3)_2Cl_2$ (0.26 g, 0.38 mmol, 0.05 equiv) and CuI (0.14 g, 0.75 mmol, 0.10 equiv) under N_2. The mixture was stirred for 12 h at 80 °C under nitrogen atmosphere. The reaction quenched by water and extracted three times with ethyl acetate. The combined organic layers were washed with brine, dried over anhydrous sodium sulfate and concentrated under reduced pressure. The residue was purified by silica gel chromatography (hexanes/EtOAc, 0 - 5%) to afford the title compound **SI_11** as a yellow solid (1.60 g, 4.75 mmol, 63% yield).

LCMS *m/z* calculated for $C_{23}H_{29}O_2^+$ ($[M+H]^+$): 337.2 found: 337.1.

bioRxiv preprint doi: https://doi.org/10.64898/2026.04.24.720406; this version posted April 27, 2026. The copyright holder for this preprint (which was not certified by peer review) is the author/funder, who has granted bioRxiv a license to display the preprint in perpetuity. It is made available under aCC-BY 4.0 International license.

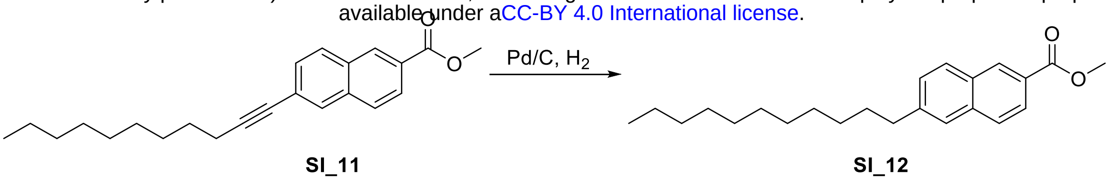

Methyl 6-undecyl-2-naphthoate (SI_12): To a mixture of methyl 6-(undec-1-yn-1-yl)-2-naphthoate (**SI_11**, 1.6 g, 4.76 mmol, 1.00 equiv) in 8.0 mL methanol 10% palladium on carbon (0.54 g, 0.51 mmol, 0.11 equiv) was added. The reaction mixture was stirred overnight under hydrogen atmosphere (50 psi). The reaction mixture was filtered through a celite pad and the filtrate was evaporated to dryness. The residue was used in the next step directly without further purification.

bioRxiv preprint doi: https://doi.org/10.64898/2026.04.24.720406; this version posted April 27, 2026. The copyright holder for this preprint (which was not certified by peer review) is the author/funder, who has granted bioRxiv a license to display the preprint in perpetuity. It is made available under aCC-BY 4.0 International license.

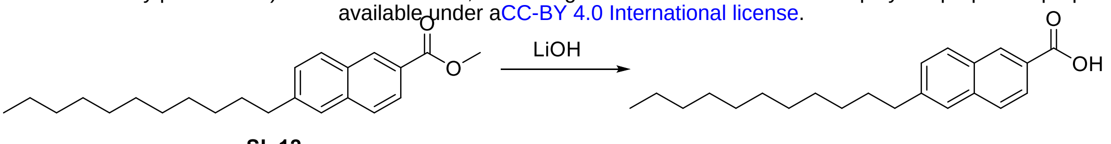

π-FA4: To a solution of methyl 6-undecyl-2-naphthoate (**SI_12**, 500 mg, 1.47 mmol, 1.00 equiv) in a mixture of methanol and water (1:1, 4.0 mL total) was added $LiOH{\cdot}H_2O$ (123.3 mg, 2.94 mmol, 2.00 equiv) and the reaction stirred for 12 h at room temperature. The reaction mixture was diluted with water, the residue was adjusted to pH = 1, which was extracted three times with ethyl acetate. The combined organic layers were washed with brine, dried over Na_2SO_4, filtered and concentrated under reduced pressure. The residue was purified by silica gel chromatography (hexanes/EtOAc, 10 - 55%) to afford the title product **π-FA4** as a white solid (223 mg, 0.66 mmol, 45% yield).

^{1}H NMR (400 MHz, DMSO-d_6) δ 12.96 (s, 1H), 8.54 (s, 1H), 8.01 (d, J = 8.4 Hz, 1H), 7.97 – 7.87 (m, 2H), 7.75 (s, 1H), 7.46 (d, J = 8.4 Hz, 1H), 2.75 (t, J = 7.5 Hz, 3H), 1.71 – 1.60 (m, 3H), 1.21 (s, 14H), 0.83 (t, J = 6.8 Hz, 3H).

^{13}C NMR (101 MHz, DMSO-d_6) δ 167.5, 142.8, 135.2, 130.6, 130.3, 129.2, 128.2, 127.6, 127.3, 125.9, 125.2, 35.4, 31.3, 30.6, 29.01, 28.97, 28.96, 28.8, 28.70, 28.66, 22.1, 13.9.

LCMS *m/z* calculated for $C_{22}H_{31}O_2^+$ ($[M+H]^+$): 327.2 found:327.1.

bioRxiv preprint doi: https://doi.org/10.64898/2026.04.24.720406; this version posted April 27, 2026. The copyright holder for this preprint (which was not certified by peer review) is the author/funder, who has granted bioRxiv a license to display the preprint in perpetuity. It is made available under aCC-BY 4.0 International license.

Alkyl Glycerol Probes

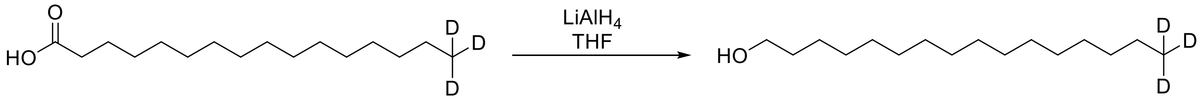

Palmitic acid-d_3 **SI_13**

Palmytol-d$_3$ (SI-13): Palmitic acid-d$_3$ (50.0 mg, 0.192 mmol, 1.00 equiv) was dissolved in 0.40 mL THF and added to a cooled (0 °C) solution of $LiAlH_4$ (11.0 mg, 0.290 mmol, 1.50 equiv) in 0.6 mL THF. The resulting solution slowly warmed to ambient temperature over two hours. The reaction was quenched by the addition of sat. NH_4Cl, and the mixture extracted with MBTE (3x). The combined organic solvents were dried over Na_2SO_4, filtered and evaporated to a crude which was immediately used in the next reaction

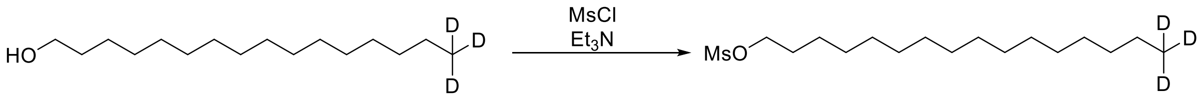

SI_13 **SI_14**

Palmytol-d$_3$ mesylate (SI_14): Unpurified alcohol (assumed quant., 0.192 mmol, 1.00 equiv) was dissolved in 3.0 mL CH_2Cl_2 and cooled to 0°C using an ice bath. Sequentially a solution of Et_3N (53.5 µL in 540 µL CH_2Cl_2, 0.384 mmol, 2.00 equiv) and MsCl (22.3 µL in 450 µL CH_2Cl_2, 0.288 mmol, 1.50 equiv) were added. Upon stirring for 30 min, the reaction was quenched by the addition of sat. $NaHCO_3$ and the resulting mixture extracted 3x with CH_2Cl_2. The combined organic phases were dried over Na_2SO_4, filtered and evaporated to give the crude mesylate **SI-14**, which was immediately employed in the subsequent reaction.

bioRxiv preprint doi: https://doi.org/10.64898/2026.04.24.720406; this version posted April 27, 2026. The copyright holder for this preprint (which was not certified by peer review) is the author/funder, who has granted bioRxiv a license to display the preprint in perpetuity. It is made available under aCC-BY 4.0 International license.

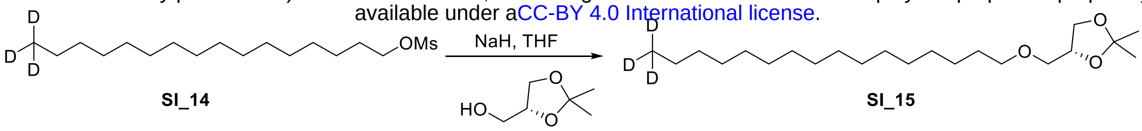

SI_14 **SI_15**

D_3-Pa-Alkylated (SI_15): (R)-(2,2-dimethyl-1,3-dioxolan-4-yl)methanol (75 mg, 0.576 mmol, 3.00 equiv) was dissolved in 1.0 mL THF and stirred. NaH (24 mg, 0576 mmol, 3.00 equiv, 60% in oil) was added at once and the resulting mixture stirred for 10 minutes. Then, a solution of Mesylate **SI_14** (unpurified, assumed quant., 0.192 mmol, 1.00 equiv) in 0.4 mL THF (flask rinsed 4x with 0.05 mL THF) was added and the resulting mixture heated to 50 °C overnight. The reaction was then cooled to ambient temperature and quenched by the addition of sat. NH_4Cl. The mixture was extracted 3x with MTBE, the combined organic phases were dried over Na_2SO_4, filtered and evaporated to a crude. Purification by column chromatography gave desired product **SI_15** as a clear oil (13 mg, 36.2 µmol, 19% yield over three steps).

^{1}H NMR (400 MHz, $CDCl_3$) δ 4.26 (p, J = 6.0 Hz, 1H), 4.06 (dd, J = 8.2, 6.4 Hz, 1H), 3.73 (dd, J = 8.2, 6.4 Hz, 1H), 3.56 – 3.37 (m, 4H), 1.65 – 1.51 (m, 2H), 1.42 (s, 3H), 1.36 (s, 3H), 1.25 (s, 26H).

^{13}C NMR (101 MHz, $CDCl_3$) δ 109.5, 74.9, 72.1, 72.0, 67.1, 32.0, 29.85, 29.83, 29.81, 29.76, 29.75, 29.70, 29.6, 29.5, 26.9, 26.2, 25.6, 22.6.

Note: The resonance corresponding to the CD_3 is not visible in the ^{13}C NMR, also 3 resonances corresponding to carbon atoms within the fatty acid chain are not visible due to overlap.

HRMS *m/z* calculated for $C_{22}H_{42}D_3O_3$ $[M+H]^+$: 360.3552, found: 360.3552.

bioRxiv preprint doi: https://doi.org/10.64898/2026.04.24.720406; this version posted April 27, 2026. The copyright holder for this preprint (which was not certified by peer review) is the author/funder, who has granted bioRxiv a license to display the preprint in perpetuity. It is made available under aCC-BY 4.0 International license.

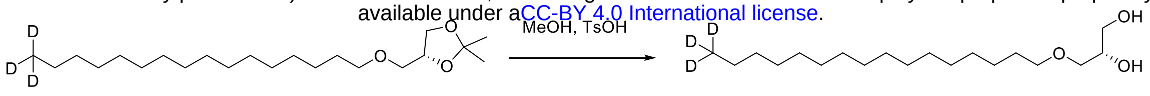

d_3-Alkyl glycerol: Acetonide **SI_15** (13 mg, 36.2 µmol, 1.00 equiv) was dissolved in 0.9 mL MeOH and stirred. TsOH (7.0 mg, 36.2 µmol, 1.00 equiv) was added at once and the reaction mixture warmed to 35°C. After two hours the reaction was cooled to ambient temperature and quenched by the addition of sat. NaHCO3. The mixture was extracted 3x with EtOAc, the combined organic phases filtered and evaporated to a crude. Purification by column chromatography gave desired product **d_3-alkyl glycerol** as a white solid (10.2 mg, 36.2 µmol, 88%).

^{1}H NMR (400 MHz, $CDCl_3$) δ 3.86 (tt, *J* = 5.6, 4.0 Hz, 1H), 3.72 (dd, *J* = 11.4, 3.9 Hz, 1H), 3.64 (dd, *J* = 11.4, 5.2 Hz, 1H), 3.57 – 3.39 (m, 4H), 1.57 (p, *J* = 6.7 Hz, 2H), 1.25 (s, 26H).

^{13}C NMR (101 MHz, $CDCl_3$) δ 72.7, 72.0, 70.6, 64.5, 32.0, 29.84, 29.82, 29.81, 29.76, 29.73, 29.6, 29.5, 26.2, 22.6.

Note: The resonance corresponding to the CD_3 is not visible in the ^{13}C NMR, also 4 resonances corresponding to carbon atoms within the fatty acid chain are not visible due to overlap.

HRMS *m/z* calculated for $C_{19}H_{38}D_3O_3$ $[M+H]^+$: 320.3239, found: 320.3239.

bioRxiv preprint doi: https://doi.org/10.64898/2026.04.24.720406; this version posted April 27, 2026. The copyright holder for this preprint (which was not certified by peer review) is the author/funder, who has granted bioRxiv a license to display the preprint in perpetuity. It is made available under aCC-BY 4.0 International license.

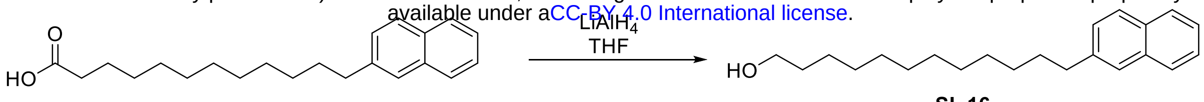

-FA1 SI_16

π-FA1-alcohol (SI_16): π-FA1 (10.0 mg, 0.031 mmol, 1.00 equiv) was dissolved in 0.60 mL THF and cooled to 0 °C using an ice-water bath. $LiAlH_4$ (3.5 mg, 0.092 mmol, 3.00 equiv) was added and the resulting solution slowly warmed to ambient temperature over 2 hours. The reaction was quenched by the addition of sat. NH_4Cl, and the mixture extracted with MBTE (3x). The combined organic solvents were dried over Na_2SO_4, filtered and evaporated to a crude which was immediately used in the next reaction:

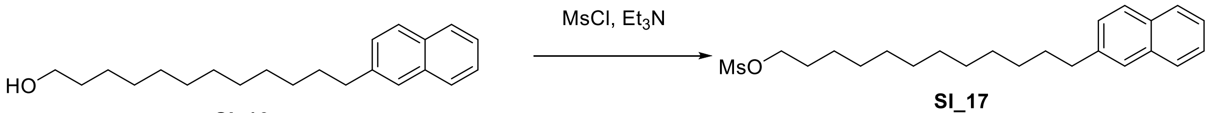

SI_16 SI_17

π -FA1-mesylate (SI_17): Unpurified alcohol **SI_16** (assumed quant., 0.031 mmol, 1.00 equiv) was dissolved in 0.6 mL CH_2Cl_2 and cooled to 0°C using an ice bath. Sequentially a solution of Et_3N (8.5 µL in 85 µL CH_2Cl_2, 0.061 mmol, 2.00 equiv) and MsCl (3.6 µL in 72 µL CH_2Cl_2, 0.046 mmol, 1.50 equiv) were added. Upon stirring for 30 min, the reaction was quenched by the addition of sat. $NaHCO_3$ and the resulting mixture extracted 3x with CH_2Cl_2. The combined organic phases were dried over Na_2SO_4 and purified by preparative TLC (6:2:2 Hexanes/CH_2Cl_2/MTBE) to give the desired compound **SI_17** as a colorless solid (7.4 mg, 0.019 mmol, 61%).

^{1}H NMR (400 MHz, $CDCl_3$) δ 7.71 (ddd, *J* = 13.9, 8.0, 3.0 Hz, 3H), 7.53 (s, 1H), 7.41 – 7.29 (m, 2H), 7.26 (dd, *J* = 8.4, 1.7 Hz, 1H), 4.14 (t, *J* = 6.6 Hz, 2H), 2.92 (d, *J* = 0.8 Hz, 3H), 2.73 – 2.65 (m, 2H), 1.72 – 1.57 (m, 4H), 1.40 – 1.05 (m, 16H).

^{13}C NMR (101 MHz, $CDCl_3$) δ 140.5, 133.6, 131.9, 127.7, 127.6, 127.5, 127.4, 126.3, 125.8, 125.0, 70.2, 37.4, 36.1, 31.4, 29.59, 29.56, 29.54, 29.51, 29.42, 29.35, 29.1, 29.0, 25.4.

Note: HMRS data for the parent compound could not be detected, only an ion consistent with loss of MsO$^-$:

HRMS *m/z* calculated for $C_{22}H_{31}$ [M-MsO$^-$]$^+$: 295.2420, found: 295.2418.

bioRxiv preprint doi: https://doi.org/10.64898/2026.04.24.720406; this version posted April 27, 2026. The copyright holder for this preprint (which was not certified by peer review) is the author/funder, who has granted bioRxiv a license to display the preprint in perpetuity. It is made available under aCC-BY 4.0 International license.

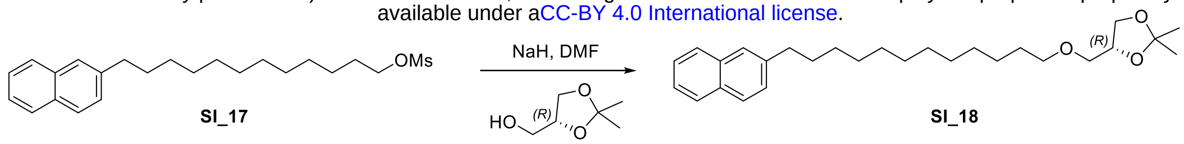

π-FA1-acetonide (SI_18): (R)-(2,2-dimethyl-1,3-dioxolan-4-yl)methanol (12.5 mg, 0.095 mmol, 5.00 equiv) was dissolved in 0.30 mL THF and cooled to 0 °C using an ice-water bath. NaH (3.9 mg mg, 0.095 mmol, 5.00 equiv, 60%) was added and the resulting mixture stirred until a clear solution was obtained (10 min). Then, Mesylate **SI_17** (7.4 mg, 0.019 mmol, 1.00 equiv) was added as a solution in THF (0.2 mL for dissolution, rinsed 4x with 0.05 mL each). The resulting solution was then concentrated to ca. A third of its volume under a stream of N_2, sealed and heated to 45 °C overnight. TLC analysis showed incomplete conversion, so the reaction was further heated to 55 °C for 2 hours. The mixture was then quenched by the addition of sat. $NaHCO_3$ and the resulting mixture extracted 3x with MTBE. The combined organic phases were dried over Na_2SO_4, filtered and evaporated to a crude. Purification by pTLC gave desired product **SI_18** as a colorless solid (4.6 mg, 10.8 µmol, 57%).

^{1}H NMR (400 MHz, $CDCl_3$) δ 7.78 (ddd, J = 14.2, 7.9, 3.2 Hz, 3H), 7.61 (d, J = 1.7 Hz, 1H), 7.48 – 7.38 (m, 2H), 7.33 (dd, J = 8.4, 1.8 Hz, 1H), 4.26 (p, J = 6.0 Hz, 1H), 4.06 (dd, J = 8.3, 6.4 Hz, 1H), 3.73 (dd, J = 8.2, 6.4 Hz, 1H), 3.56 – 3.36 (m, 4H), 2.81 – 2.72 (m, 2H), 1.70 (p, J = 7.5 Hz, 2H), 1.56 (m, 2H), 1.42 (s, 3H), 1.36 (s, 3H), 1.32 – 1.20 (m, 16H).

^{13}C NMR (101 MHz, $CDCl_3$) δ 140.6, 133.8, 132.0, 127.8, 127.7, 127.6, 127.5, 126.4, 125.9, 125.1, 109.5, 74.9, 72.04, 71.96, 67.1, 36.3, 31.5, 29.8, 29.73, 29.73, 29.70, 29.69, 29.6, 29.5, 26.9, 26.2, 25.6.

HRMS *m/z* calculated for $C_{28}H_{43}O_3$ $[M+H]^+$: 427.3207, found: 427.3210.

bioRxiv preprint doi: https://doi.org/10.64898/2026.04.24.720406; this version posted April 27, 2026. The copyright holder for this preprint (which was not certified by peer review) is the author/funder, who has granted bioRxiv a license to display the preprint in perpetuity. It is made available under aCC-BY 4.0 International license.

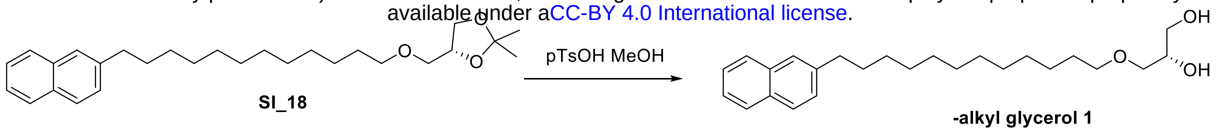

π-Alkyl glycerol 1: Acetonide **SI_18** (4.6 mg, 10.8 umol, 1.00 equiv) was dissolved in 0.3 mL MeOH and stirred. TsOH (2.1 mg, 10.8 umol, 1.00 equiv) was added at once, and the reaction mixture warmed to 35 °C. Upon stirring for 3 hours, the reaction was quenched by the addition of sat. $NaHCO_3$ and the resulting mixture extracted 3x with EtOAc. The combined organic phases were dried over Na_2SO_4, filtered and evaporated to a crude. Purification by pTLC gave desired product **π-alkyl glycerol 1** as a white solid (2.5 mg, 6.5 µmol, 60%).

^{1}H NMR (400 MHz, $CDCl_3$) δ 7.78 (ddd, J = 14.1, 7.9, 3.1 Hz, 3H), 7.61 (d, J = 1.7 Hz, 1H), 7.50 – 7.37 (m, 2H), 7.33 (dd, J = 8.3, 1.7 Hz, 1H), 3.85 (d, J = 7.2 Hz, 1H), 3.72 (d, J = 11.6 Hz, 1H), 3.65 (dd, J = 11.3, 5.0 Hz, 1H), 3.58 – 3.39 (m, 4H), 2.81 – 2.72 (m, 2H), 2.57 (s, 1H), 2.14 (s, 1H), 1.70 (p, J = 7.5 Hz, 2H), 1.57 (t, J = 6.7 Hz, 4H), 1.39 – 1.17 (m, 14H).

^{13}C NMR (101 MHz, $CDCl_3$) δ 140.6, 133.8, 132.0, 127.8, 127.7, 127.6, 127.5, 126.4, 125.9, 125.1, 72.7, 72.0, 70.5, 64.5, 36.3, 31.5, 29.9, 29.8, 29.72 (2C, overlap), 29.71, 29.68, 29.6, 29.5, 26.2.

HRMS *m/z* calculated for $C_{25}H_{39}O_3$ $[M+H]^+$: 387.2894, found: 387.2871.

bioRxiv preprint doi: https://doi.org/10.64898/2026.04.24.720406; this version posted April 27, 2026. The copyright holder for this preprint (which was not certified by peer review) is the author/funder, who has granted bioRxiv a license to display the preprint in perpetuity. It is made available under aCC-BY 4.0 International license.

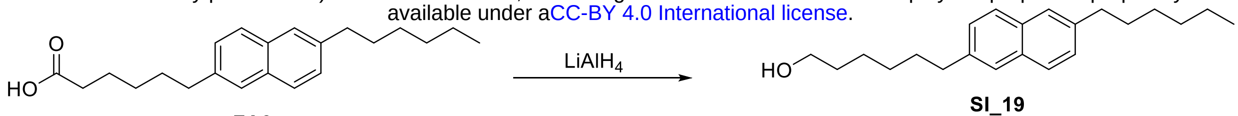

π-FA3-alcohol (SI_19): **π-FA3** (20.0 mg, 0.062 mmol, 1.00 equiv) was dissolved in 1.20 mL THF and cooled to 0 °C using an ice-water bath. $LiAlH_4$ (7.00 mg, 0.186 mmol, 3.00 equiv) was added and the resulting solution slowly warmed to ambient temperature over 3 hours. The reaction was quenched by the addition of sat. NH_4Cl, and the mixture extracted with MBTE (3x). The combined organic solvents were dried over Na_2SO_4, filtered and evaporated to give crude **SI_19** which was immediately used in the next reaction:

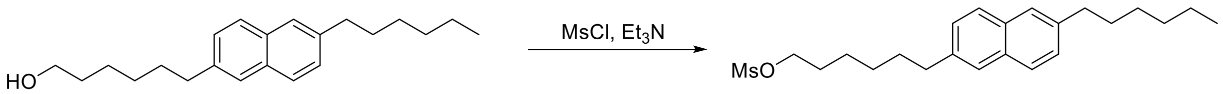

π-FA1-mesylate (SI_20): Unpurified alcohol **SI_19** (assumed quant., 0.062 mmol, 1.00 equiv) was dissolved in 1.20 mL CH_2Cl_2 and cooled to 0°C using an ice bath. Sequentially, Et_3N (19 µL 0.124 mmol, 2.00 equiv) and MsCl (7.2 µL 0.093 mmol, 1.50 equiv) were added. Upon stirring for 30 min, the reaction was quenched by the addition of sat. $NaHCO_3$ and the resulting mixture extracted 3x with CH_2Cl_2. The combined organic phases were dried over Na_2SO_4 and purified by preparative TLC (6:2:2 Hexanes/CH_2Cl_2/MTBE) to give the desired compound **SI_20** as a colorless solid (20.6 mg, 52.7 µmol, 85% over two steps).

^{1}H NMR (400 MHz, $CDCl_3$) δ 7.70 (dd, *J* = 8.3, 5.6 Hz, 2H), 7.57 (d, *J* = 2.1 Hz, 2H), 7.30 (ddd, *J* = 12.7, 8.4, 1.7 Hz, 2H), 4.28 – 4.21 (m, 2H), 2.97 (s, 3H), 2.86 – 2.79 (m, 2H), 2.79 – 2.70 (m, 2H), 1.82 (ttd, *J* = 7.7, 4.6, 1.9 Hz, 4H), 1.71 – 1.65 (m, 2H), 1.41 – 1.22 (m, 10H), 0.96 – 0.84 (m, 3H).

^{13}C NMR (101 MHz, $CDCl_3$) δ 140.1, 138.2, 132.4, 132.1, 127.8, 127.7, 127.4, 127.2, 126.4, 126.2, 70.0, 37.5, 36.2, 35.4, 32.0, 31.6, 29.7, 29.5, 29.4, 28.7, 27.2, 22.8, 14.3.

Note: HMRS data for the parent compound could not be detected, only an ion consistent with loss of MsO^-:

HRMS *m/z* calculated for $C_{22}H_{31}$ $[M-MsO^-]^+$: 295.2420, found: 295.2421.

bioRxiv preprint doi: https://doi.org/10.64898/2026.04.24.720406; this version posted April 27, 2026. The copyright holder for this preprint (which was not certified by peer review) is the author/funder, who has granted bioRxiv a license to display the preprint in perpetuity. It is made available under aCC-BY 4.0 International license.

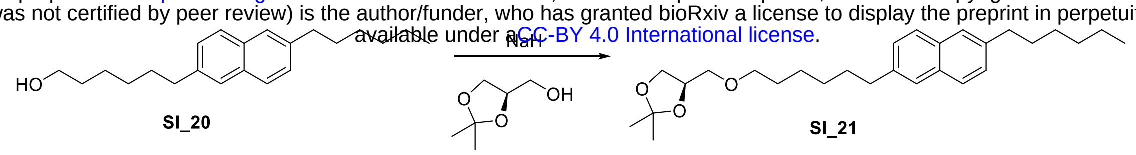

π-FA3-acetonide (SI_21): (R)-(2,2-dimethyl-1,3-dioxolan-4-yl)methanol (34.9 mg, 0.264 mmol, 5.00 equiv) was dissolved in 0.80 mL THF and cooled to 0 °C using an ice-water bath. NaH (6.3 mg mg, 0.264 mmol, 5.00 equiv, 60%) was added and the resulting mixture stirred until a clear solution was obtained (10 min). Then, mesylate **SI_20** (20.6 mg, 0.053 mmol, 1.00 equiv) was added as a solution in THF (0.4 mL for dissolution, rinsed 4x with 0.05 mL each). The resulting solution was then sealed and heated to 50 °C overnight. The mixture was then quenched by the addition of sat. $NaHCO_3$ and the resulting mixture extracted 3x with MTBE. The combined organic phases were dried over Na_2SO_4, filtered and evaporated to a crude. Purification by pTLC gave desired product **SI_21** as a colorless solid (12.6 mg, 29.5 µmol, 56%).

^{1}H NMR (400 MHz, $CDCl_3$) δ 7.69 (dd, *J* = 8.5, 2.2 Hz, 2H), 7.56 (Bs, 2H), 7.29 (ddd, *J* = 8.4, 4.3, 1.6 Hz, 2H), 4.31 – 4.20 (m, 1H), 4.05 (dd, *J* = 8.3, 6.4 Hz, 1H), 3.72 (dd, *J* = 8.3, 6.4 Hz, 1H), 3.56 – 3.44 (m, 3H), 3.41 (dd, *J* = 9.9, 5.5 Hz, 1H), 2.76 (dt, *J* = 11.4, 7.6 Hz, 6H), 1.82 – 1.59 (m, 6H), 1.42 (s, 3H), 1.36 (s, 3H), 1.35 – 1.22 (m, 8H), 0.93 – 0.85 (m, 3H).

^{13}C NMR (101 MHz, $CDCl_3$) δ 139.8, 139.1, 132.3, 132.2, 127.6, 127.5, 127.43, 127.36, 126.3, 126.2, 109.5, 74.9, 72.0, 71.8, 67.1, 36.2, 35.9, 32.0, 31.6, 29.7, 29.5, 29.4, 29.3, 28.0, 26.9, 25.6, 22.8, 14.3.

HRMS *m/z* calculated for $C_{28}H_{43}O_3$ $[M+H]^+$: 427.3207, found: 427.3210.

bioRxiv preprint doi: https://doi.org/10.64898/2026.04.24.720406; this version posted April 27, 2026. The copyright holder for this preprint (which was not certified by peer review) is the author/funder, who has granted bioRxiv a license to display the preprint in perpetuity. It is made available under aCC-BY 4.0 International license.

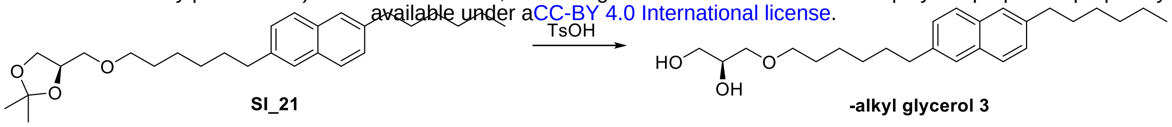

SI_21 -alkyl glycerol 3

π-Alkyl glycerol 3: Acetonide **SI_21** (12.6 mg, 29.5 umol, 1.00 equiv) was dissolved in 1.20 mL MeOH and stirred. TsOH (5.6 mg, 29.5 umol, 1.00 equiv) was added at once, and the reaction mixture warmed to 35 °C. Upon stirring for 3 hours, the reaction was quenched by the addition of sat. $NaHCO_3$ and the resulting mixture extracted 3x with EtOAc. The combined organic phases were dried over Na_2SO_4, filtered and evaporated to a crude. Purification by pTLC gave desired product **π-alkyl glycerol 3** as a white solid (8.7 mg, 23 µmol, 76%).

^{1}H NMR (400 MHz, $CDCl_3$) δ 7.39 (dd, J = 8.3, 3.1 Hz, 2H), 7.26 (s, 2H), 6.99 (ddd, J = 8.4, 6.5, 1.7 Hz, 2H), 3.54 (ddd, J = 7.8, 5.7, 3.9 Hz, 1H), 3.40 (dd, J = 11.4, 3.9 Hz, 1H), 3.33 (dd, J = 11.4, 5.2 Hz, 1H), 3.26 – 3.13 (m, 4H), 2.53 – 2.40 (m, 4H), 2.27 (s, 1H), 1.84 (s, 1H), 1.49 – 1.31 (m, 6H), 1.13 – 0.92 (m, 10H), 0.69 – 0.53 (m, 3H).

^{13}C NMR (101 MHz, $CDCl_3$) δ 139.9, 138.9, 132.3, 132.2, 127.7, 127.5, 127.4, 126.3, 126.2, 72.6, 71.7, 70.6, 64.4, 36.2, 35.9, 32.0, 31.6, 29.9, 29.7, 29.5, 29.4, 29.3, 27.9, 22.8, 14.3.

HRMS m/z calculated for $C_{25}H_{39}O_3$ $[M+H]^+$: 387.2894, found: 387.2891.

bioRxiv preprint doi: https://doi.org/10.64898/2026.04.24.720406; this version posted April 27, 2026. The copyright holder for this preprint (which was not certified by peer review) is the author/funder, who has granted bioRxiv a license to display the preprint in perpetuity. It is made available under aCC-BY 4.0 International license.

π-Sphingosines

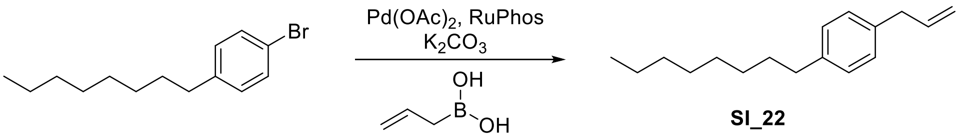

1-Allyl-4-octylbenzene (SI_22): 1-Bromo-4-octylbenzene (2.00 g, 7.43 mmol, 1.00 equiv) was dissolved in a mixture of PhMe and H_2O (22 mL, 10:1). Allylboronic acid (0.77 g, 8.91 mmol, 1.20 equiv), K_2CO_3 (3.08 g, 22.3 mmol, 3.00 equiv), $Pd(OAc)_2$ (197 mg, 0.88 mmol, 0.12 equiv), and Ruphos (819 mg, 1.76 mmol, 0.24 equiv) were added and the reaction mixture was stirred for 5 h at 80°C. EtOAc and water were added, the phases separated and the aqueous phase twice extracted with EtOAc. The combined organic phases were dried over Na_2SO_4, filtered, and concentrated. The mixture was purified by flash column chromatography (hexanes) to yield the title compound **SI_22** as colorless oil (1.20 g, 5.20 mmol, 70%).

^{1}H NMR (400 MHz, $CDCl_3$) δ 7.11 (s, 4H), 5.97 (ddt, *J* = 16.9, 10.0, 6.7 Hz, 1H), 5.13 – 5.01 (m, 2H), 3.36 (d, *J* = 6.7 Hz, 2H), 2.63 – 2.50 (m, 2H), 1.65 – 1.56 (m, 2H), 1.32 – 1.23 (m, 10H), 0.87 (d, *J* = 7.0 Hz, 3H).

bioRxiv preprint doi: https://doi.org/10.64898/2026.04.24.720406; this version posted April 27, 2026. The copyright holder for this preprint (which was not certified by peer review) is the author/funder, who has granted bioRxiv a license to display the preprint in perpetuity. It is made available under aCC-BY 4.0 International license.

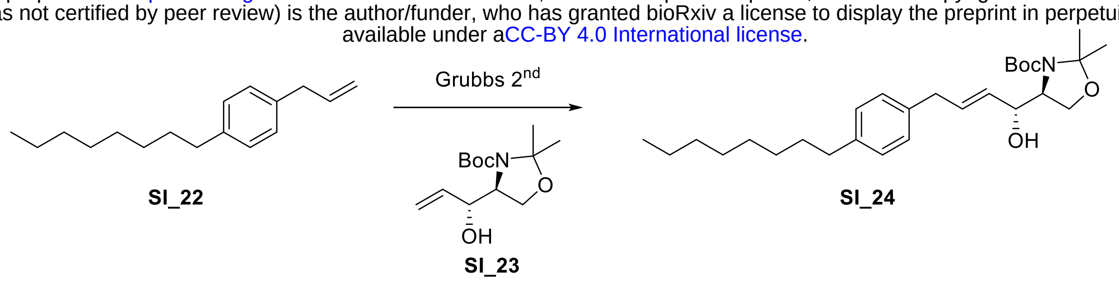

tert-butyl (S)-4-((R,E)-1-hydroxy-4-(4-octylphenyl)but-2-en-1-yl)-2,2-dimethyloxazolidine-3-carboxylate (SI_24): 1-Allyl-4-octylbenzene (100 mg, 0.43 mmol, 1.00 equiv) was dissolved in CH_2Cl_2 (5 mL). The vinyl adduct of Garner's aldehyde (**SI_23**, 223 mg, 0.866 mmol, 2.00 equiv) and HG-II (5 mg, 5.89 µmol, 1.37 %) were added and the reaction mixture was stirred for 16h at 40°C. The reaction mixture was concentrated and purified by flash column chromatography (PE/EA: 0% ~ 10%) to yield **SI_24** as colorless oil (80 mg, 0.174 mmol 40%).

LCMS: *m/z* calculated for $C_{27}H_{46}NO_4Na^+$ ($[M+Na]^+$): 482.3 found: 482.4.

Note: ***SI_23*** *was prepared according to the procedure outlined in J. Org. Chem.* ***1998****, 22, 7999.*

bioRxiv preprint doi: https://doi.org/10.64898/2026.04.24.720406; this version posted April 27, 2026. The copyright holder for this preprint (which was not certified by peer review) is the author/funder, who has granted bioRxiv a license to display the preprint in perpetuity. It is made available under aCC-BY 4.0 International license.

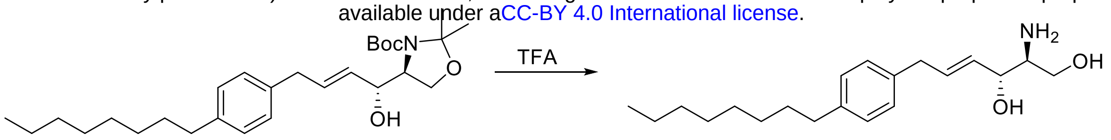

SI_24 **-SPh1**

π-Sph1: SI_24 (80.0 mg, 0.17 mmol, 1.00 equiv) was dissolved in 15.0 mL DCM. 3.00 mL TFA were added and the reaction mixture stirred for 1 hour at ambient temperature, then it was concentrated and the residue directly purified by HPLC (C18, MeCN/H_2O 40-50% over 16 min, R_t = 8.3-9.5 min) to give the title compound **π-Sph1** as a colorless solid (26.5 mg, 0.082 mmol, 48%).

^{1}H NMR (400 MHz, MeOD) δ 7.10 (s, 4H), 5.99 (dt, *J* = 14.1, 6.8 Hz, 1H), 5.54 (dd, *J* = 15.3, 6.7 Hz, 1H), 4.36 – 4.28 (m, 1H), 3.79 (dd, *J* = 11.6, 4.0 Hz, 1H), 3.66 (dd, *J* = 11.6, 8.4 Hz, 1H), 3.39 (d, *J* = 6.8 Hz, 2H), 3.22 (dt, *J* = 8.5, 4.3 Hz, 1H), 2.61 – 2.52 (m, 2H), 1.59 (d, *J* = 7.1 Hz, 2H), 1.30 (m, 10H), 0.89 (t, *J* = 6.8 Hz, 3H).

LCMS *m/z* calculated for $C_{20}H_{34}NO_2^+$ ($[M+H]^+$): 320.3 found: 320.2.

bioRxiv preprint doi: https://doi.org/10.64898/2026.04.24.720406; this version posted April 27, 2026. The copyright holder for this preprint (which was not certified by peer review) is the author/funder, who has granted bioRxiv a license to display the preprint in perpetuity. It is made available under aCC-BY 4.0 International license.

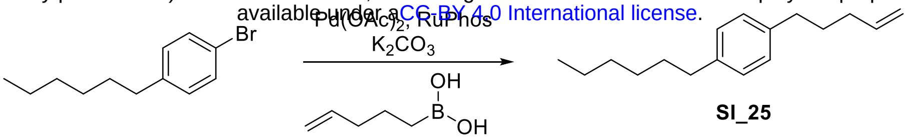

SI_25

1-Hexyl-4-(pent-4-en-1-yl)benzene (SI_25): 1-Bromo-4-hexylbenzene (3.10 g, 21.1 mmol, 1.00 equiv) was dissolved in a mixture of PhMe and H_2O (22 mL, 10:1). 4-Pentenylboronic acid (2.00 g, 17.6 mmol, 0.83 equiv), K_2CO_3 (7.30 g, 52.7 mmol, 2.50 equiv), $Pd(OAc)_2$ (197 mg, 0.88 mmol, 0.042 equiv), and Ruphos (819 mg, 1.76 mmol, 0.083 equiv) were added and the reaction mixture was stirred for 5 h at 80°C. EtOAc and water were added, the phases separated and the aqueous phase twice extracted with EtOAc. The combined organic phases were dried over Na_2SO_4, filtered, and concentrated. The mixture was purified by flash column chromatography (hexanes) to yield the title compound as colorless oil (1.50 g, 7.81 mmol, 37%).

^{1}H NMR (400 MHz, $CDCl_3$) δ 7.02 (s, 4H), 5.77 (ddt, J = 17.0, 10.1, 6.6 Hz, 1H), 5.00 – 4.86 (m, 2H), 2.51 (q, J = 8.4 Hz, 4H), 2.02 (q, J = 7.1 Hz, 2H), 1.62 (q, J = 7.5 Hz, 2H), 1.55 – 1.49 (m, 2H), 1.25 (d, J = 9.4 Hz, 6H), 0.81 (t, J = 6.5 Hz, 3H).

bioRxiv preprint doi: https://doi.org/10.64898/2026.04.24.720406; this version posted April 27, 2026. The copyright holder for this preprint (which was not certified by peer review) is the author/funder, who has granted bioRxiv a license to display the preprint in perpetuity. It is made available under aCC-BY 4.0 International license.

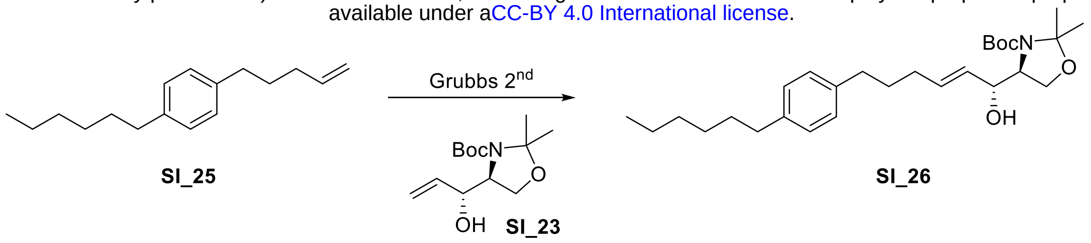

SI_25

SI_26

tert-butyl (S)-4-((R,E)-6-(4-hexylphenyl)-1-hydroxyhex-2-en-1-yl)-2,2-dimethyloxazolidine-3-carboxylate (SI_26): SI_25 (1.50 g, 6.51 mmol, 1.00 equiv) was dissolved in CH_2Cl_2 (15 mL). The vinyl adduct of Garner's aldehyde **SI_23** (2.50 g, 9.77 mmol, 1.50 equiv) and HG-II (50 mg, 0.0589 mmol, 0.90 mol%) were added and the reaction mixture was stirred for 16 h at 40°C. The reaction mixture was concentrated and purified by flash column chromatography (PE/EA: 0% ~ 10%) to yield **SI_26** as colorless oil (1.90 g, 2.60 mmol, 40%).

LCMS *m/z* calculated for $C_{27}H_{46}NO_4Na^+$ ($[M+Na]^+$): 482.3 found: 482.4.

*Note: **SI_23** was prepared according to the procedure outlined in J. Org. Chem. **1998**, 22, 7999.*

bioRxiv preprint doi: https://doi.org/10.64898/2026.04.24.720406; this version posted April 27, 2026. The copyright holder for this preprint (which was not certified by peer review) is the author/funder, who has granted bioRxiv a license to display the preprint in perpetuity. It is made available under aCC-BY 4.0 International license.

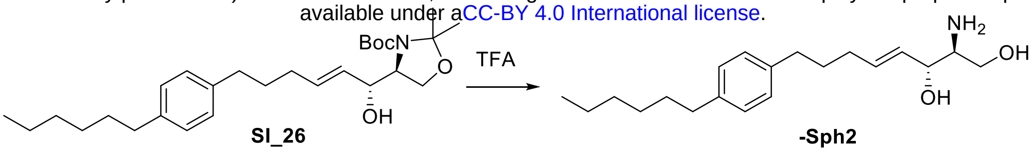

SI_26 **-Sph2**

π-Sph2: SI_26 (200 mg, 0.43 mmol, 1.00 equiv) was dissolved in 15.0 mL DCM. 3.0 mL TFA was added and the resulting solution stirred for 1 hour at ambient temperature, then it was concentrated and directly purified by HPLC (C18, MeCN/H_2O 42-52% over 16 min, R_t = 7.5-8.0 min) to give the title compound **π-Sph2** as a colorless solid (34.5 mg, 0.11 mmol, 25%).

^{1}H NMR (400 MHz, DMSO-d_6) δ 7.75 (s, 2H), 7.08 (s, 4H), 5.73 (dt, J = 13.9, 6.5 Hz, 1H), 5.45 (dd, J = 17.6, 5.5 Hz, 2H), 5.14 (t, J = 4.7 Hz, 1H), 4.18 (d, J = 5.5 Hz, 1H), 3.60 (dt, J = 8.1, 4.1 Hz, 1H), 3.52 – 3.41 (m, 1H), 3.03 (s, 1H), 2.55 (d, J = 7.6 Hz, 2H), 2.03 (q, J = 6.7 Hz, 2H), 1.63 (p, J = 7.5 Hz, 2H), 1.52 (d, J = 7.3 Hz, 2H), 1.27 (s, 6H), 0.89 – 0.81 (m, 3H).

LCMS *m/z* calculated for $C_{20}H_{34}NO_2^+$ $[M+H]^+$: 320.3 found: 320.2.

bioRxiv preprint doi: https://doi.org/10.64898/2026.04.24.720406; this version posted April 27, 2026. The copyright holder for this preprint (which was not certified by peer review) is the author/funder, who has granted bioRxiv a license to display the preprint in perpetuity. It is made available under aCC-BY 4.0 International license.

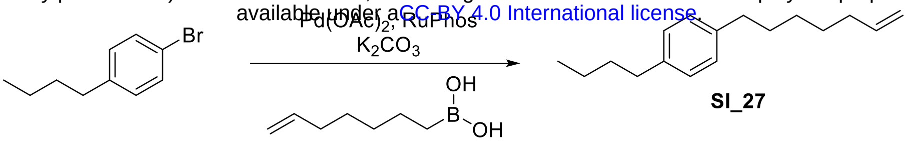

SI_27

1-Butyl-4-(hept-6-en-1-yl)benzene (SI_27): 1-Bromo-4-butylbenzene (2.40 g, 16.9 mmol, 1.00 equiv) was dissolved in a mixture of PhMe and H2O (22 mL, 10:1). 6-heptenylboronic acid (3.00 g, 14.1 mmol, 0.83 equiv), K_2CO_3 (5.80 g, 42.2 mmol, 2.50 equiv), $Pd(OAc)_2$ (159 mg, 0.70 mmol, 0.042 equiv), and Ruphos (657 mg, 1.76 mmol, 0.083 equiv) were added and the reaction mixture was stirred for 5 h at 80°C. EtOAc and water were added, the phases separated and the aqueous phase twice extracted with EtOAc. The combined organic phases were dried over Na_2SO_4, filtered, and concentrated. The mixture was purified by flash column chromatography (hexanes) to yield the title compound **SI_27** as colorless oil (1.20 g, 6.25 mmol, 37%).

^{1}H NMR (400 MHz, $CDCl_3$) δ 7.09 (s, 4H), 5.81 (ddt, *J* = 17.0, 10.1, 6.7 Hz, 1H), 5.04 – 4.90 (m, 1H), 2.58 (td, *J* = 8.0, 2.4 Hz, 4H), 2.05 (q, *J* = 6.9 Hz, 2H), 1.65 – 1.57 (m, 4H), 1.38 (ddq, *J* = 22.2, 14.6, 7.5 Hz, 6H), 0.93 (t, *J* = 7.3 Hz, 3H).

bioRxiv preprint doi: https://doi.org/10.64898/2026.04.24.720406; this version posted April 27, 2026. The copyright holder for this preprint (which was not certified by peer review) is the author/funder, who has granted bioRxiv a license to display the preprint in perpetuity. It is made available under aCC-BY 4.0 International license.

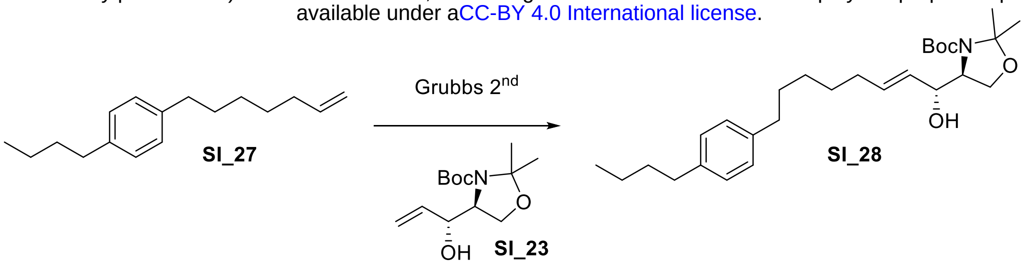

tert-tert-butyl (S)-4-((R,E)-8-(4-butylphenyl)-1-hydroxyoct-2-en-1-yl)-2,2-dimethyloxazolidine-3-carboxylate (SI_28): SI_27 (1.00 g, 4.30 mmol, 1.00 equiv) was dissolved in 10.0 mL CH_2Cl_2. The vinyl adduct of Garner's aldehyde (1.60 g, 6.51 mmol, 1.51 equiv) and HG-II (60 mg, 0.071 mmol, 1.64 mol%) were added and the reaction mixture was stirred for 16 h at 40°C. The reaction mixture was concentrated and purified by flash column chromatography (PE/EA: 0% ~ 10%) to yield **SI_28** as colorless oil (1.10 g, 2.37 mmol, 40%).

LCMS *m/z* calculated for $C_{27}H_{46}NO_4Na^+$ ($[M+Na]^+$): 482.3 found: 482.4.

*Note: **SI_23** was prepared according to the procedure outlined in J. Org. Chem. **1998**, 22, 7999.*

bioRxiv preprint doi: https://doi.org/10.64898/2026.04.24.720406; this version posted April 27, 2026. The copyright holder for this preprint (which was not certified by peer review) is the author/funder, who has granted bioRxiv a license to display the preprint in perpetuity. It is made available under aCC-BY 4.0 International license.

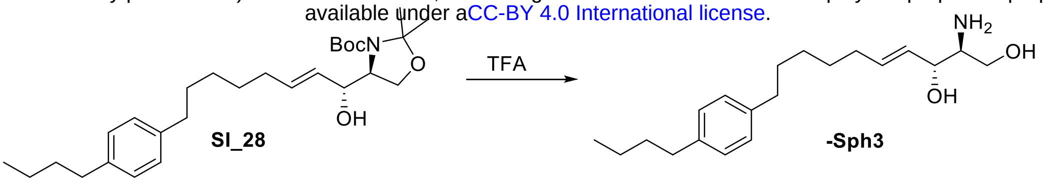

π-Sph3: SI_28 (200 mg, 0.43 mmol, 1.00 equiv) was dissolved in 15.0 mL DCM. 3.0 mL TFA was added and the resulting solution stirred for 1 hour at ambient temperature, then it was concentrated and directly purified by HPLC (C18, MeCN/H_2O 46-56% over 16 min, R_t = 7.0-8.7 min) to give the title compound **π-Sph3** as a colorless solid (37.2 mg, 0.12 mmol, 27%).

^{1}H NMR (400 MHz, DMSO-d_6) δ 7.72 (s, 2H), 7.08 (s, 4H), 5.69 (dt, *J* = 14.0, 6.8 Hz, 1H), 5.43 (dd, *J* = 14.5, 5.5 Hz, 2H), 5.16 – 5.09 (m, 1H), 4.17 (d, *J* = 5.4 Hz, 1H), 3.64 – 3.56 (m, 1H), 3.49 – 3.42 (m, 1H), 3.05 – 2.99 (m, 1H), 2.54 (s, 2H), 2.01 (q, *J* = 6.7 Hz, 2H), 1.52 (dt, *J* = 15.3, 7.4 Hz, 4H), 1.31 (ddt, *J* = 21.8, 14.5, 7.1 Hz, 6H), 0.89 (t, *J* = 7.3 Hz, 3H).

LCMS *m/z* calculated for $C_{20}H_{33}NO_2^+$ ([M+H]$^+$): 319.5 found: 320.2.

bioRxiv preprint doi: https://doi.org/10.64898/2026.04.24.720406; this version posted April 27, 2026. The copyright holder for this preprint (which was not certified by peer review) is the author/funder, who has granted bioRxiv a license to display the preprint in perpetuity. It is made available under aCC-BY 4.0 International license.

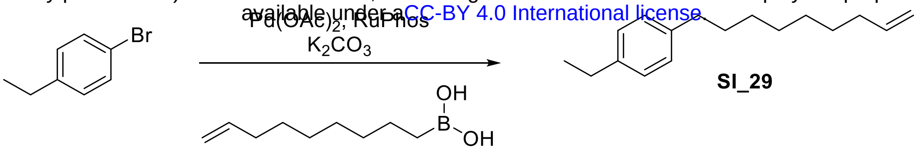

1-Ethyl-4-(non-8-en-1-yl)benzene (SI_29): 1-Bromo-4-ethylbenzene (15.7 g, 84.7 mmol, 1.00 equiv) was dissolved in a mixture of PhMe and H_2O (143 mL, 10:1). 8-nonenylboronic acid (12.0 g, 70.6 mmol, 0.83 equiv), K_2CO_3 (29.2 g, 211 mmol, 2.50 equiv), $Pd(OAc)_2$ (792 mg, 3.52 mmol, 0.50 equiv), and Ruphos (3.28 g, 8.82 mmol, 0.10 equiv) were added and the reaction mixture was stirred for 5 h at 80°C. EtOAc and water were added, the phases separated and the aqueous phase twice extracted with EtOAc. The combined organic phases were dried over Na_2SO_4, filtered, and concentrated. The mixture was purified by flash column chromatography (hexanes) to yield the title compound **SI_29** as colorless oil (13.0 g, 73.7 mmol, 37%).

^{1}H NMR (400 MHz, $CDCl_3$) δ 7.15 – 7.04 (m, 4H), 5.81 (ddt, J = 16.9, 10.2, 6.7 Hz, 1H), 5.04 – 4.89 (m, 2H), 2.67 – 2.53 (m, 4H), 2.04 (q, J = 6.9 Hz, 2H), 1.59 (q, J = 7.3 Hz, 2H), 1.35 (d, J = 24.2 Hz, 8H), 1.23 (t, J = 7.6 Hz, 3H).

bioRxiv preprint doi: https://doi.org/10.64898/2026.04.24.720406; this version posted April 27, 2026. The copyright holder for this preprint (which was not certified by peer review) is the author/funder, who has granted bioRxiv a license to display the preprint in perpetuity. It is made available under aCC-BY 4.0 International license.

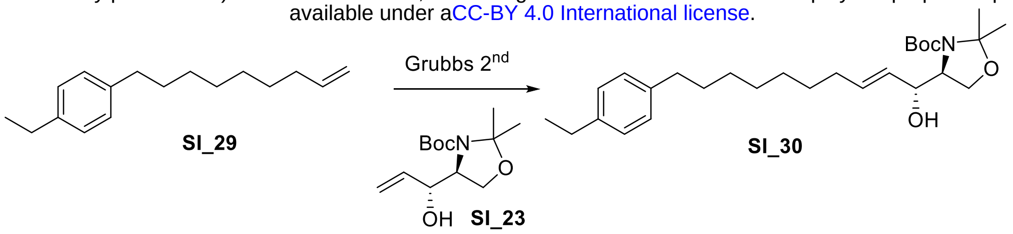

Tert-butyl (S)-4-((R,E)-10-(4-ethylphenyl)-1-hydroxydec-2-en-1-yl)-2,2-dimethyloxazolidine-3-carboxylate (SI_30): SI_29 (2.00 g, 8.70 mmol, 1.00 equiv) was dissolved in 20.0 mL DCM. The vinyl adduct of Garner's aldehyde **SI_23** (2.00 g, 7.90 mmol, 0.88 equiv) and HG-II (120 mg, catalytic) were added and the reaction mixture stirred at 40 °C for 16 hours. The mixture was concentrated and purified by column chromatography (hexanes/EtOAc 0-10%) to give the title compound **SI_29** as a colorless oil (1.45 g, 3.13 mmol, 36%).

LCMS *m/z* calculated for $C_{27}H_{46}NO_4Na^+$ ($[M+Na]^+$): 482.3 found: 482.4.

*Note: **SI_23** was prepared according to the procedure outlined in J. Org. Chem. **1998**, 22, 7999.*

bioRxiv preprint doi: https://doi.org/10.64898/2026.04.24.720406; this version posted April 27, 2026. The copyright holder for this preprint (which was not certified by peer review) is the author/funder, who has granted bioRxiv a license to display the preprint in perpetuity. It is made available under aCC-BY 4.0 International license.

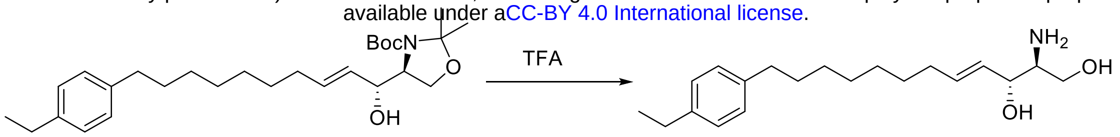

SI_30 -Sph4

π-Sph4: SI_30 (300 mg, 0.65 mmol, 1.00 equiv) was dissolved in 10.0 mL DCM. 2.0 mL TFA was added and the resulting solution stirred for 1 hour at ambient temperature, then it was concentrated and directly purified by HPLC (C18, MeCN/H_2O 43-53% over 16 min, R_t = 7.8-10.0 min) to give the title compound **π-Sph4** as a colorless solid (34.3 mg, 0.11 mmol, 17%).

^{1}H NMR (400 MHz, MeOD) δ 7.11 – 7.02 (m, 4H), 5.90 – 5.78 (m, 1H), 5.47 (dd, J =15.4, 6.9 Hz, 1H), 4.30 – 4.23 (m, 1H), 3.79 (dd, J = 11.6, 4.1 Hz, 1H), 3.65 (dd, J =11.6, 8.3 Hz, 1H), 3.18 (dt, J = 8.6, 4.2 Hz, 1H), 2.64 – 2.51 (m, 4H), 2.10 (q, J = 6.9Hz, 2H), 1.59 (t, J = 6.8 Hz, 2H), 1.45 – 1.38 (m, 2H), 1.34 (d, J = 4.5 Hz, 6H), 1.20 (t,J = 7.6 Hz, 3H).

LCMS *m/z* calculated for $C_{20}H_{33}NO_2^+$ ($[M+H]^+$): 319.5 found: 320.2.

bioRxiv preprint doi: https://doi.org/10.64898/2026.04.24.720406; this version posted April 27, 2026. The copyright holder for this preprint (which was not certified by peer review) is the author/funder, who has granted bioRxiv a license to display the preprint in perpetuity. It is made available under aCC-BY 4.0 International license.

Azido-Stearic Acids

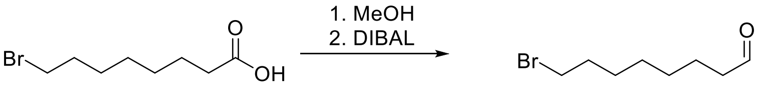

SI_31

8-Bromooctanal (SI_31): 8-Bromooctanoic acid (1.88 g, 8.43 mmol, 1.00 equiv) was dissolved in 25.6 mL MeOH. Dropwise, sulfuric acid (0.23 mL, 4.22 mmol, 0.50 equiv) was added and the resulting solution stirred at ambient temperature for 1 hour. The mixture was then poured into sat. $NaHCO_3$ and the resulting mixture extracted 3x with EtOAc. The combined organic phases were dried over Na_2SO_4, filtered and evaporated to a crude, which was directly subjected to the next reaction.

Methyl-8-bromooctanoate (assumed quant. 8.43 mmol, 1.00 equiv) was dissolved in 40.0 mL DCM and cooled to – 78 °C. Dropwise, DIBAL (9.0 mL, 9.02 mmol, 1.07 equiv, 1 M solution in PhMe) was added and the resulting mixture stirred for 1 hour at -78 C. The reaction was quenched by the addition of 1.7 mL Methanol to the reaction, then warmed to ambient temperature and poured into 1 M HCl. The reaction mixture was extracted 3x with DCM, the combined organic phases were dried over Na_2SO_4, filtered and evaporated to a crude. Purification by column chromatography gave the desired product **SI_31** as a colorless oil (910 mg, 4.39 mmol, 52% over two steps.)

^{1}H NMR (400 MHz, $CDCl_3$) δ 9.76 (t, J = 1.8 Hz, 1H), 3.40 (t, J = 6.8 Hz, 2H), 2.43 (td, J = 7.3, 1.8 Hz, 2H), 1.85 (p, J = 6.9 Hz, 2H), 1.69 – 1.55 (m, 2H), 1.50 – 1.40 (m, 2H), 1.34 (p, J = 3.6 Hz, 4H).

^{13}C NMR (101 MHz, $CDCl_3$) δ 202.9, 44.0, 34.0, 32.8, 29.1, 28.6, 28.1, 22.1.

bioRxiv preprint doi: https://doi.org/10.64898/2026.04.24.720406; this version posted April 27, 2026. The copyright holder for this preprint (which was not certified by peer review) is the author/funder, who has granted bioRxiv a license to display the preprint in perpetuity. It is made available under aCC-BY 4.0 International license.

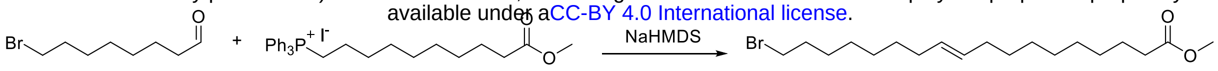

SI_31 SI_32 SI_33

Methyl (E)-18-bromooctadec-10-enoate (SI_33): Wittig reagent **SI_32** (505 mg, 0.878 mmol, 1.30 equiv) was azeotroped 3x with PhMe, then dissolved in 5.00 mL THF and cooled to 0 °C. Dropwise, NaHMDS (0.88 mL, 0.878 mmol, 1.30 equiv) was added and the resulting bright orange solution stirred for 10 minutes. The solution containing the ylide was then cooled to -78 °C and a solution of 8-Bromooctanal (**SI_31**, 140.0 mg, 0.676 mmol, 1.00 equiv) in THF (0.7 mL for dissolution, 3x 0.1 mL rinse) was added to the mixture. The reaction was then slowly warmed to ambient temperature over 3 hours, then quenched by pouring the reaction mixture into sat. NH_4Cl. The resulting mixture was extracted 3x with EtOAc, the combined organic phases were dried over Na_2SO_4, filtered and evaporated to a crude. Purification by column chromatography gave desired product as a colorless oil (170 mg, 0.453 mmol, 67%, mixture of E/Z isomers).

^{1}H NMR (400 MHz, $CDCl_3$) δ 5.39 – 5.25 (m, 2H), 3.66 (s, 3H), 3.40 (t, J = 6.9 Hz, 2H), 2.30 (t, J = 7.5 Hz, 2H), 2.07 – 1.95 (m, J = 3.0 Hz, 4H), 1.85 (dt, J = 14.3, 6.9 Hz, 2H), 1.60 (dt, J = 9.9, 6.9 Hz, 2H), 1.49 – 1.39 (m, 2H), 1.37 – 1.20 (m, 16H).

^{13}C NMR (101 MHz, $CDCl_3$) δ 174.5, 130.1, 129.9, 51.6, 34.24, 34.15, 33.0, 29.9, 29.7, 29.5, 29.4 (2C), 29.3, 29.2, 28.8, 28.3, 27.33, 27.26, 25.1.

*Note: Wittig reagent **SI_32** was prepared according to Helvetica Chimica Acta, **1974**, 57, 434.*

bioRxiv preprint doi: https://doi.org/10.64898/2026.04.24.720406; this version posted April 27, 2026. The copyright holder for this preprint (which was not certified by peer review) is the author/funder, who has granted bioRxiv a license to display the preprint in perpetuity. It is made available under aCC-BY 4.0 International license.

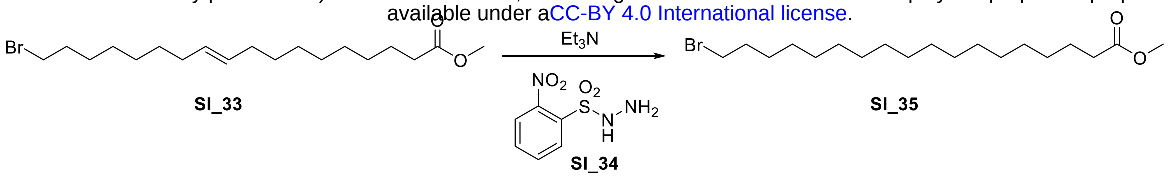

Methyl 18-bromooctadecanoate (SI_35): SI_33 (100.0 mg, 0.266 mmol, 1.00 equiv) was dissolved in 2.50 mL DCM. Sequentially, Et_3N (0.37 mL, 2.66 mmol, 10.0 equiv) and Hydrazide **SI_34** (289 mg, 1.33 mmol, 5.00 equiv) were added and the resulting suspension stirred overnight. The mixture was then poured into sat. $NaHCO_3$ and the phases separated. The aqueous phase was further extracted 3x with DCM, the combined organic phases were dried over Na_2SO_4, filtered and evaporated to a crude. Purification by column chromatography (hexanes/EtOAc) gave desired product **SI_35** as a colorless oil (69.7 mg, 0.19 mmol, 69%).

^{1}H NMR (400 MHz, $CDCl_3$) δ 3.66 (s, 3H), 3.41 (t, J = 6.9 Hz, 2H), 2.30 (t, J = 7.5 Hz, 2H), 1.85 (p, J = 7.0 Hz, 2H), 1.62 (p, J = 7.2 Hz, 2H), 1.41 (q, J = 7.1 Hz, 2H), 1.26 (d, J = 10.3 Hz, 24H).

^{13}C NMR (101 MHz, $CDCl_3$) δ 174.5, 51.6, 34.3, 34.2, 33.0, 29.81, 29.81, 29.80, 29.79, 29.76, 29.74, 29.69, 29.60, 29.59, 29.4, 29.3, 28.9, 28.3, 25.1.

HRMS *m/z* calculated for $C_{19}H_{38}BrO_2$ $[M+H]^+$: 377.2050, found: 377.2050.

*Note: Hydrazide **SI_34** was prepared according to J. Am. Chem. Soc. **2017**, 139, 15636.*

bioRxiv preprint doi: https://doi.org/10.64898/2026.04.24.720406; this version posted April 27, 2026. The copyright holder for this preprint (which was not certified by peer review) is the author/funder, who has granted bioRxiv a license to display the preprint in perpetuity. It is made available under aCC-BY 4.0 International license.

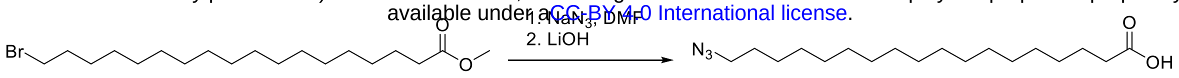

SI_35 **cl-SA**

18-Azido-stearic acid (cl-SA): **SI_35** (68 mg, 0.18 mmol, 1.00 equiv) was dissolved in 0.75 mL DMF, followed by the addition of NaN_3 (59 mg, 0.90 mmol, 5.00 equiv). The resulting mixture was stirred for 24 hours, then poured into sat. $NaHCO_3$. The mixture was extracted 3x with EtOAc, the combined organic phases were dried over Na_2SO_4, filtered and evaporated to a crude. The crude was azeotroped 3x with heptanes, then directly subjected to the next reaction.

Unpurified 18-azido-stearic acid methyl ester (assumed quant. 0.18 mmol, 1.00 equiv) was dissolved in a mixture of 1.00 mL THF, 0.40 mL MeOH and 0.20 mL water and cooled to 0°C. $LiOH{\cdot}H_2O$ (23.0 mg, 0.54 mmol, 3.00 equiv) was added and the mixture slowly allowed to warm to ambient temperature. After stirring for four hours, the reaction mixture was poured into 1 M HCl and the ensuing mixture extracted with DCM 3x. The combined organic phases were dried over Na_2SO_4, filtered and evaporated to a solid, which was recrystallized from hexanes to give the desired product **cl-SA** as a white solid (29 mg, 0.089 mmol, 49% over two steps).

^{1}H NMR (400 MHz, $CDCl_3$) δ 10.30 (bs, 1H), 3.25 (t, J = 7.0 Hz, 2H), 2.35 (t, J = 7.5 Hz, 2H), 1.62 (dp, J = 14.4, 7.3 Hz, 4H), 1.27 (d, J = 8.2 Hz, 26H).

^{13}C NMR (101 MHz, $CDCl_3$) δ 178.7, 51.7, 33.9, 29.80, 29.78, 29.77, 29.73, 29.69, 29.63, 29.58, 29.4, 29.3, 29.2, 29.0, 26.9, 24.8.

Note: 2 Resonances corresponding to the alkyl chain are not visible in the ^{13}C NMR due to overlap.

HRMS *m/z* calculated for $C_{19}H_{34}N_3O_2$ [M-H]$^-$: 324.2657, found: 324.2657.

bioRxiv preprint doi: https://doi.org/10.64898/2026.04.24.720406; this version posted April 27, 2026. The copyright holder for this preprint (which was not certified by peer review) is the author/funder, who has granted bioRxiv a license to display the preprint in perpetuity. It is made available under aCC-BY 4.0 International license.

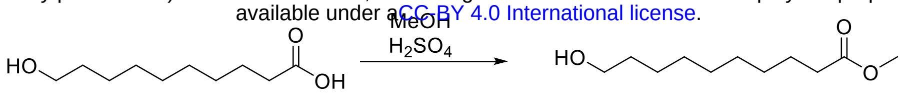

SI_36

10-Hydroxydecanoic acid methyl ester (SI_36): 10-hydroxydecanoic acid (6.00 g, 31.9 mmol, 1.00 equiv) was suspended in 130 mL MeOH and stirred vigorously. Dropwise, H_2SO_4 (0.85 mL, 15.9 mmol, 0.50 equiv) was added. The resulting mixture was then heated to reflux for 30 min, cooled to ambient temperature and poured into sat. $NaHCO_3$. The mixture was extracted 3x with EtOAc, the combined organic phases were washed with water and brine, dried over Na_2SO_4, filtered and evaporated to give **SI_36** (6.27 g, 31.0 mmol, 97%) as a pale yellow oil, which was immediately used in subsequent reactions.

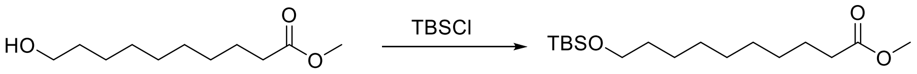

SI_36 SI_37

Methyl 10-((tert-butyldimethylsilyl)oxy)decanoate (SI_37): SI_36 (5.27 g, 26.1 mmol, 1.00 equiv) was dissolved in 60.0 mL DCM and cooled to 0 °C. Sequentially, Imidazole (4.61 g, 67.7 mmol, 2.60 equiv) and TBSCl (5.10 g, 33.9 mmol, 1.30 equiv) was added and the resulting solution stirred for 2 hours, slowly warming to ambient temperature. The reaction was then poured onto sat. NH_4Cl, the resulting mixture extracted 3x with DCM and the combined organic phases dried over Na_2SO_4, filtered and evaporated to a crude. Purification by column chromatography gave desired product **SI_37** as colorless oil (6.80 g, 21.5 mmol, 83%).

^{1}H NMR (400 MHz, $CDCl_3$) δ 3.66 (d, J = 0.7 Hz, 3H), 3.59 (t, J = 6.6 Hz, 2H), 2.30 (t, J = 7.6 Hz, 2H), 1.67 – 1.56 (m, 3H), 1.50 (p, J = 7.0 Hz, 2H), 1.29 (d, J = 5.5 Hz, 9H), 0.89 (d, J = 0.7 Hz, 9H), 0.04 (s, 6H).

^{13}C NMR (101 MHz, $CDCl_3$) δ 174.5, 63.5, 51.6, 34.3, 33.0, 29.57, 29.51, 29.34, 29.28, 26.1, 25.9, 25.1, 18.5, -5.1.

Identical characterization as reported in: *Chemistry and Physics of Lipids*, **1999**, *97*, 87-91.

bioRxiv preprint doi: https://doi.org/10.64898/2026.04.24.720406; this version posted April 27, 2026. The copyright holder for this preprint (which was not certified by peer review) is the author/funder, who has granted bioRxiv a license to display the preprint in perpetuity. It is made available under aCC-BY 4.0 International license.

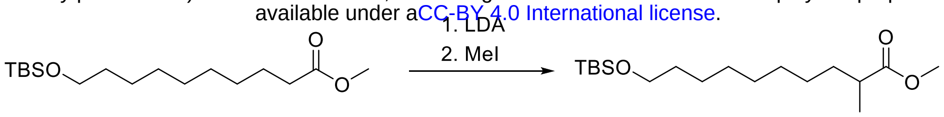

SI_37 SI_38

Methyl 10-((tert-butyldimethylsilyl)oxy)-2-methyldecanoate (SI_38): A LDA solution was prepared by dissolving *i*-Pr_2NH (0.98 mL, 6.95 mmol, 1.10 equiv) in 13.0 mL THF and cooling to 0 °C, followed by the dropwise addition of n-BuLi (4.3 mL, 6.95 mmol, 1.10 equiv). After stirring for 20 min, a solution of **SI_37** (2.00 g, 6.32 mmol, 1.00 equiv) in 12 mL THF was added and the resulting mixture stirred at 0 °C for 30 min. Then, MeI (0.43 mL, 6.95 mmol, 1.00 equiv) was added and the reaction stirred for 2 hours, slowly warming to ambient temperature. The mixture was then poured onto sat. NH_4Cl and the mixture extracted three times with EtOAc. The combined organic phases were dried over Na_2SO_4, filtered and evaporated to a crude. Purification by column chromatography (hexanes/EtOAc) gave desired product as a colorless oil (1.35 g, 4.08 mmol, 65%).

^{1}H NMR (400 MHz, $CDCl_3$) δ 3.67 (s, 3H), 3.59 (t, J = 6.6 Hz, 2H), 2.43 (h, J = 7.0 Hz, 1H), 1.71 – 1.60 (m, 2H), 1.56 – 1.44 (m, 2H), 1.28 (d, J = 4.7 Hz, 10H), 1.14 (d, J = 7.0 Hz, 3H), 0.89 (s, 9H), 0.04 (s, 6H).

^{13}C NMR (101 MHz, $CDCl_3$) δ 177.6, 63.5, 51.6, 39.6, 34.0, 33.0, 29.6, 29.5, 27.4, 26.1, 25.9, 18.5, 17.2, -5.1.

Identical characterization as reported in: *Chemistry and Physics of Lipids*, **1999**, *97*, 87-91.

bioRxiv preprint doi: https://doi.org/10.64898/2026.04.24.720406; this version posted April 27, 2026. The copyright holder for this preprint (which was not certified by peer review) is the author/funder, who has granted bioRxiv a license to display the preprint in perpetuity. It is made available under aCC-BY 4.0 International license.

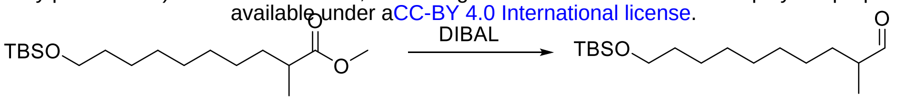

SI_38 SI_39

10-((Tert-butyldimethylsilyl)oxy)-2-methyldecanal (SI_39): SI_38 (260 mg, 0.786 mmol, 1.00 equiv) was dissolved in 7.0 mL DCM and cooled to -78 °C. Dropwise, a solution of DIBAL-H (1.02 mL, 1.02 mmol, 1.30 equiv, 1 M in DCM) was added and the resulting solution stirred for 1 hour. The raction was then quenched by the rapid addition of MeOH at -78 °C. The mixture was then poured into sat. NH_4Cl, and the phases separated. The aqueous phase was further extracted 3x with DCM, the combined organic phases were dried over Na_2SO_4, filtered and evaporated to a crude. Purification by column chromatography (hexanes/EtOAc) gave desired product **SI_39** as a colorless oil (150 mg, 0.499 mmol, 64%).

^{1}H NMR (400 MHz, $CDCl_3$) δ 9.61 (d, *J* = 2.0 Hz, 1H), 3.59 (t, *J* = 6.6 Hz, 2H), 2.33 (hd, *J* = 6.8, 2.0 Hz, 1H), 1.76 – 1.65 (m, 1H), 1.50 (p, *J* = 6.8 Hz, 2H), 1.38 – 1.22 (m, 11H), 1.08 (d, *J* = 7.0 Hz, 3H), 0.89 (s, 9H), 0.04 (s, 6H).

^{13}C NMR (101 MHz, $CDCl_3$) δ 205.6, 63.4, 46.5, 33.0, 30.7, 29.7, 29.6, 29.5, 27.1, 26.1, 25.9, 18.5, 13.5, -5.1.

bioRxiv preprint doi: https://doi.org/10.64898/2026.04.24.720406; this version posted April 27, 2026. The copyright holder for this preprint (which was not certified by peer review) is the author/funder, who has granted bioRxiv a license to display the preprint in perpetuity. It is made available under aCC-BY 4.0 International license.

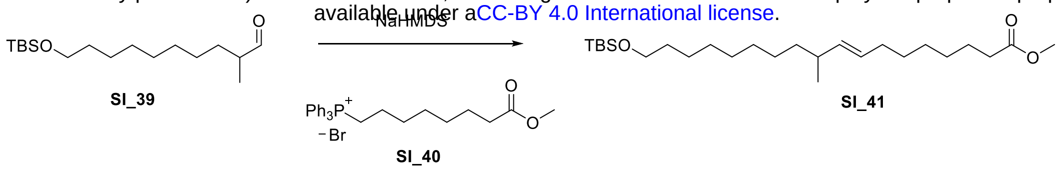

Methyl (E)-18-((tert-butyldimethylsilyl)oxy)-12-methyloctadec-10-enoate (SI_41): Wittig reagent **SI_40** (324 mg, 0.649 mmol, 1.30 equiv) was azeotroped 3x with PhMe, then dissolved in 3.00 mL THF and cooled to 0 °C. Dropwise, NaHMDS (0.65 mL, 0.649 mmol, 1.30 equiv) was added and the resulting bright orange solution stirred for 10 minutes. The solution containing the ylide was then cooled to -78 °C and a solution of **SI_39** (150.0 mg, 0.499 mmol, 1.00 equiv) in THF (0.7 mL for dissolution, 3x 0.1 mL rinse) was added to the mixture. The reaction was then slowly warmed to ambient temperature over 3 hours, then quenched by pouring the reaction mixture into sat. NH_4Cl. The resulting mixture was extracted 3x with EtOAc, the combined organic phases were dried over Na_2SO_4, filtered and evaporated to a crude. Purification by column chromatography gave desired product **SI_41** as a colorless oil (165 mg, 0.374 mmol, 75%, mixture of E/Z isomers).

^{1}H NMR (400 MHz, $CDCl_3$) δ 5.32 – 5.21 (m, 1H), 5.10 (ddt, J = 11.0, 9.7, 1.5 Hz, 1H), 3.66 (s, 3H), 3.59 (t, J = 6.6 Hz, 2H), 2.38 (d, J = 9.3 Hz, 1H), 2.30 (t, J = 7.6 Hz, 2H), 2.06 – 1.96 (m, 2H), 1.72 – 1.57 (m, 2H), 1.49 (q, J = 6.8 Hz, 2H), 1.41 – 1.18 (m, 18H), 0.91 (d, J = 6.9 Hz, 3H), 0.89 (s, 9H), 0.04 (s, 6H).

^{13}C NMR (101 MHz, $CDCl_3$) δ 174.5, 136.7, 128.2, 63.5, 51.6, 37.7, 34.3, 33.0, 31.8, 29.94, 29.87, 29.8, 29.6, 29.2, 29.1, 27.7, 27.5, 26.1, 26.0, 25.1, 21.6, 18.5, -5.1.

HRMS *m/z* calculated for $C_{26}H_{53}O_3Si$ $[M+H]^+$: 441.3759, found: 441.3758.

*Note: Wittig reagent **SI_40** was prepared according to: J. Org. Chem. **2001**, 66, 7765.*

bioRxiv preprint doi: https://doi.org/10.64898/2026.04.24.720406; this version posted April 27, 2026. The copyright holder for this preprint (which was not certified by peer review) is the author/funder, who has granted bioRxiv a license to display the preprint in perpetuity. It is made available under aCC-BY 4.0 International license.

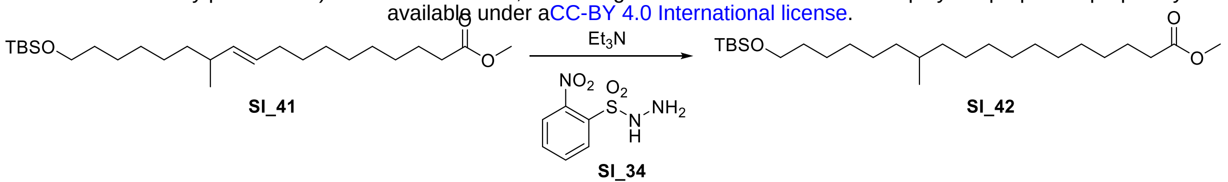

Methyl 18-((tert-butyldimethylsilyl)oxy)-10-methyloctadecanoate (SI_42): SI_41 (90.0 mg, 0.204 mmol, 1.00 equiv) was dissolved in 1.60 mL DCM. Sequentially, Et_3N (0.29 mL, 2.04 mmol, 10.0 equiv) and Hydrazide **SI_34** (222 mg, 1.02 mmol, 5.00 equiv) were added and the resulting suspension stirred overnight. The mixture was then poured into sat. $NaHCO_3$ and the phases separated. The aqueous phase was further extracted 3x with DCM, the combined organic phases were dried over Na_2SO_4, filtered and evaporated to a crude. Purification by column chromatography (hexanes/EtOAc) gave desired product **SI_42** as a colorless oil (75 mg, 0.17 mmol, 83%).

^{1}H NMR (400 MHz, $CDCl_3$) δ 3.66 (s, 3H), 3.59 (t, *J* = 6.6 Hz, 2H), 2.30 (t, *J* = 7.6 Hz, 2H), 1.61 (q, *J* = 7.1 Hz, 2H), 1.51 (h, *J* = 6.5 Hz, 2H), 1.38 – 1.18 (m, 23H), 1.16 – 0.97 (m, 2H), 0.89 (s, 9H), 0.83 (d, *J* = 6.5 Hz, 3H), 0.04 (s, 6H).

^{13}C NMR (101 MHz, $CDCl_3$) δ 174.4, 63.4, 51.5, 37.1, 34.1, 32.9, 32.8, 29.98, 29.96, 29.7, 29.51, 29.48, 29.3, 29.2, 27.09, 27.07, 26.0, 25.8, 25.0, 19.7, 18.4, -5.2.

HRMS *m/z* calculated for $C_{26}H_{55}O_3Si$ $[M+H]^+$: 443.3915, found: 443.3922.

*Note: Hydrazide **SI_34** was prepared according to J. Am. Chem. Soc. **2017**, 139, 15636.*

bioRxiv preprint doi: https://doi.org/10.64898/2026.04.24.720406; this version posted April 27, 2026. The copyright holder for this preprint (which was not certified by peer review) is the author/funder, who has granted bioRxiv a license to display the preprint in perpetuity. It is made available under aCC-BY 4.0 International license.

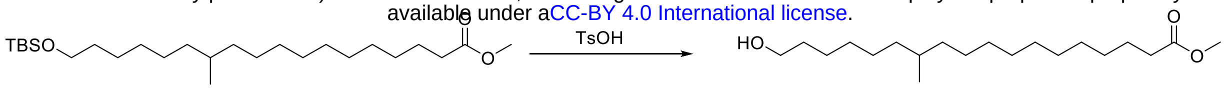

SI_42 **SI_43**

Methyl 18-hydroxy-10-methyloctadecanoate (SI_43): SI_42 (75 mg, 0.17 mmol,1.00 equiv) was dissolved in 2.00 mL MeOH. Subsequently, pTsOH (32 mg, 0.17 mmol, 1.00 equiv) was added and the resulting mixture stirred at ambient temperature for 2 hours. The reaction was then poured into sat. $NaHCO_3$, and the mixture extracted 3x with EtOAc. The combined organic phases were dried over Na_2SO_4, filtered and evaporated to a crude. Purification by column chromatography (hexanes/EtOAc) gave the desired product **SI_43** as a colorless oil (51 mg, 0.16 mmol, 91%).

^{1}H NMR (400 MHz, $CDCl_3$) δ 3.64 (s, 3H), 3.61 (t, J = 6.7 Hz, 2H), 2.28 (t, J = 7.5 Hz, 2H), 1.65 – 1.50 (m, 4H), 1.25 (m, 24H), 1.05 (dt, J = 11.3, 8.1 Hz, 2H), 0.81 (d, J = 6.5 Hz, 3H).

^{13}C NMR (101 MHz, $CDCl_3$) δ 174.5, 63.1, 51.6, 37.2, 34.2, 32.9, 32.8, 30.0, 29.8, 29.59, 29.56, 29.4, 29.3, 27.2, 27.1, 25.9, 25.1, 19.8.

HRMS *m/z* calculated for $C_{20}H_{41}O_3$ $[M+H]^+$: 329.3050, found: 329.3050.

bioRxiv preprint doi: https://doi.org/10.64898/2026.04.24.720406; this version posted April 27, 2026. The copyright holder for this preprint (which was not certified by peer review) is the author/funder, who has granted bioRxiv a license to display the preprint in perpetuity. It is made available under aCC-BY 4.0 International license.

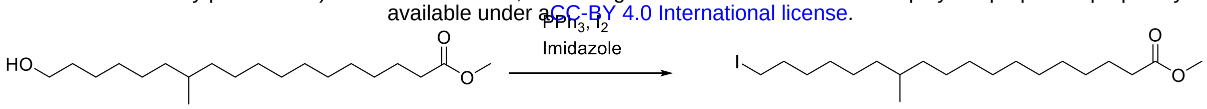

SI_43 SI_44

Methyl 18-iodo-10-methyloctadecanoate (SI_44): SI_43 (51 mg, 0.16 mmol, 1.00 equiv) was dissolved in 0.40 mL DCM and cooled to 0 °C. Sequentially, imidazole (32 mg, 0.47 mmol, 3.00 equiv), PPh_3 (61 mg, 0.23 mmol, 1.50 equiv) and I_2 (59 mg, 0.23 mmol, 1.50 equiv) were added. After stirring at 0 °C for two hours, the reaction was quenched by the simultaneous addition of sat. $NaHCO_3$ and sat. $Na_2S_3O_3$. The ensuing mixture was extracted 3x with DCM, the combined organic phases were dried over Na_2SO_4, filtered and evaporated to a crude. Purification by column chromatography gave desired product **SI_44** as a colorless oil (65 mg, 0.16 mmol, 96%).

^{1}H NMR (400 MHz, $CDCl_3$) δ 3.65 (s, 3H), 3.18 (t, J = 7.1 Hz, 2H), 2.29 (t, J = 7.6 Hz, 2H), 1.81 (p, J = 7.1 Hz, 2H), 1.61 (p, J = 7.3 Hz, 2H), 1.43 – 1.16 (m, 24H), 1.06 (dt, J = 11.3, 8.1 Hz, 2H), 0.82 (d, J = 6.5 Hz, 3H).

^{13}C NMR (101 MHz, $CDCl_3$) δ 174.4, 51.6, 37.18, 37.17, 34.2, 33.7, 32.9, 30.6, 30.1, 30.0, 29.62, 29.58, 29.4, 29.3, 28.7, 27.2, 27.1, 25.1, 19.8, 7.4.

HRMS *m/z* calculated for $C_{20}H_{41}IO_2$ $[M+H]^+$: 439.2068, found: 439.2057.

bioRxiv preprint doi: https://doi.org/10.64898/2026.04.24.720406; this version posted April 27, 2026. The copyright holder for this preprint (which was not certified by peer review) is the author/funder, who has granted bioRxiv a license to display the preprint in perpetuity. It is made available under aCC-BY 4.0 International license.

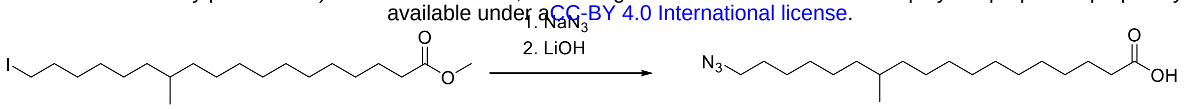

SI_44 cl-10-MeSA

18-azido-10-methyloctadecanoic acid (cl-10-MeSA): SI_44 (65 mg, 0.15 mmol, 1.00 equiv) was dissolved in 0.80 mL DMF. NaN_3 (48 mg, 0.74 mmol, 5.00 equiv) was added and the resulting solution stirred for 16 hours. The reaction was then poured into sat. $NaHCO_3$, and the ensuing mixture extracted 3x with EtOAc. The combined organic phases were dried over Na_2SO_4, filtered and evaporated to a crude, which immediately subjected to the next reaction:

Crude Methyl 18-azido-10-methyloctadecanoate (0.15 mmol, 1.00 equiv) was dissolved in a mixture of 1.0 mL THF, 0.4 mL MeOH and 0.2 mL water and cooled to 0 °C. $LiOH{\cdot}H_2O$ (19 mg, 0.44 mmol, 3.00 equiv) was then added and the reaction mixture warmed to ambient temperature. After stirring for 16 hours the reaction was poured into 1 M HCl and the mixture extracted 5x with DCM. The combined organic phases were dried over Na_2SO_4, filtered and evaporated to a crude. Purification by column chromatography followed by preparative TLC (both hexanes/EtOAc with 1% AcOH) gave desired product **cl-10-MeSA** as a colorless oil (25 mg, 0.074 mmol, 50% over two steps).

^{1}H NMR (400 MHz, $CDCl_3$) δ 9.90 (s, 1H), 3.25 (t, J = 7.0 Hz, 2H), 2.35 (t, J = 7.5 Hz, 2H), 1.77 – 1.49 (m, 4H), 1.39 – 1.20 (m, 23H), 1.07 (dt, J = 11.5, 8.1 Hz, 2H), 0.83 (d, J = 6.5 Hz, 3H).

^{13}C NMR (101 MHz, $CDCl_3$) δ 180.1, 51.6, 37.2, 32.9, 30.1, 30.0, 29.7, 29.6, 29.4, 29.3, 29.2, 29.0, 27.19, 27.17, 26.9, 24.8, 19.9.

HRMS *m/z* calculated for $C_{19}H_{36}N_3O_2$ $[M-H]^-$: 338.2813, found: 338.2821.

bioRxiv preprint doi: https://doi.org/10.64898/2026.04.24.720406; this version posted April 27, 2026. The copyright holder for this preprint (which was not certified by peer review) is the author/funder, who has granted bioRxiv a license to display the preprint in perpetuity. It is made available under aCC-BY 4.0 International license.

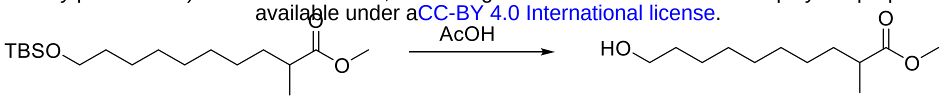

SI_38 **SI_45**

Methyl 10-hydroxy-2-methyldecanoate (SI_45): SI_38 (1.04 g, 3.15 mmol, 1.00 equiv) was dissolved in a mixture of 10.0 mL AcOH, 3.30 mL THF and 3.30 mL Water and stirred at ambient temperature for 4 hours. Then, the reaction was poured into sat. $NaHCO_3$, and the ensuing mixture extracted 3x with DCM. The combined organic phases were dried over Na_2SO_4, filtered and evaporated to a crude. Purification by column chromatography (hexanes/EtOAc) gave desired product **SI_45** as a clear oil (0.107 g, 0.495 mmol, 16%).

^{1}H NMR (400 MHz, $CDCl_3$) δ 3.66 (s, 3H), 3.63 (t, *J* = 6.6 Hz, 2H), 2.49 – 2.36 (m, 1H), 1.67 – 1.50 (m, 4H), 1.41 – 1.22 (m, 10H), 1.13 (d, *J* = 7.0 Hz, 3H).

^{13}C NMR (101 MHz, $CDCl_3$) δ 177.6, 63.2, 51.6, 39.6, 33.9, 32.9, 29.5, 29.50, 29.45, 27.3, 25.8, 17.2.

HRMS *m/z* calculated for $C_{12}H_{24}NaO_3$ $[M+Na]^+$: 239.1618, found: 239.1614.

bioRxiv preprint doi: https://doi.org/10.64898/2026.04.24.720406; this version posted April 27, 2026. The copyright holder for this preprint (which was not certified by peer review) is the author/funder, who has granted bioRxiv a license to display the preprint in perpetuity. It is made available under aCC-BY 4.0 International license.

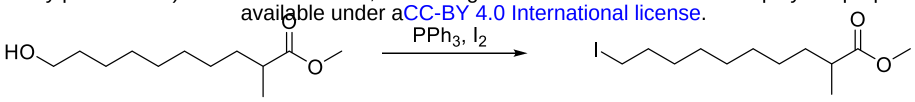

SI_45 **SI_46**

Methyl 10-iodo-2-methyldecanoate SI_46: Methyl 10-hydroxy-2-methyldecanoate (0.107 g, 0.495 mmol, 1.00 equiv) was dissolved in 5.0 mL DCM and cooled to 0 °C. Sequentially, imidazole (101 mg, 1.48 mmol, 3.00 equiv), PPh_3 (143 mg, 0.544 mmol, 1.10 equiv) and iodine (138 mg, 0.544 mmol, 1.10 equiv) were added and the resulting solution stirred for 2 hours at 0 °C. The reaction was then quenched by the simultaneous addition of sat. $Na_2S_2O_3$ and $NaHCO_3$. The phases were separated and the aqueous phase further extracted 3x with DCM. The combined organic phases were dried over Na_2SO_4, filtered and evaporated to a crude. Purification by column chromatography (hexanes/EtOAc) gave desired product **SI_46** as a colorless oil (160 mg, 0.490 mmol, 99%).

^{1}H NMR (400 MHz, $CDCl_3$) δ 3.62 (s, 3H), 3.14 (t, J = 7.0 Hz, 2H), 2.38 (h, J = 7.0 Hz, 1H), 1.77 (p, J = 7.1 Hz, 2H), 1.65 – 1.52 (m, 1H), 1.34 (td, J = 8.3, 4.5 Hz, 3H), 1.27 (s, 8H), 1.09 (d, J = 7.0 Hz, 3H).

^{13}C NMR (101 MHz, $CDCl_3$) δ 177.3, 51.5, 39.5, 33.8, 33.5, 30.5, 29.4, 29.3, 28.5, 27.2, 17.1, 7.3.

HRMS *m/z* calculated for $C_{12}H_{24}IO_2$ $[M+H]^+$: 327.0816, found: 327.0811.

bioRxiv preprint doi: https://doi.org/10.64898/2026.04.24.720406; this version posted April 27, 2026. The copyright holder for this preprint (which was not certified by peer review) is the author/funder, who has granted bioRxiv a license to display the preprint in perpetuity. It is made available under aCC-BY 4.0 International license.

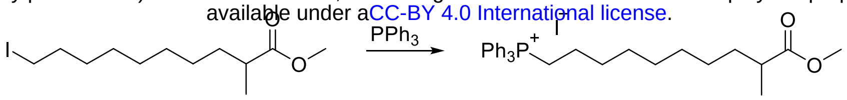

SI_46 SI_47

Methyl 10-(iodotriphenyl-l5-phosphaneyl)-2-methyldecanoate SI_47: SI_46 (160 mg, 0.49 mmol, 1.00 equiv) was dissolved in 2.00 mL PhMe. PPh_3 (180 mg, 0.687 mmol, 1.40 equiv) was added and the solution heated to reflux for 24 hours. The mixture was then cooled to ambient temperature and volatiles were removed under vacuo. The residue was dissolved in MeCN, and extracted 10x with hexanes, discarding the hexane layer. The MeCN solution was then evaporated, giving the wittig reagent **SI_47** as a colorless syrup (207 mg, 0.352 mmol, 72%) which was immediately employed in the subsequent reaction:

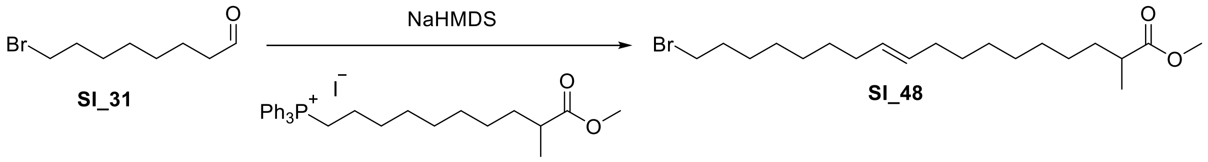

Methyl (E)-18-bromo-2-methyloctadec-10-enoate (SI_48): SI_47 (207 mg, 0.352 mmol, 1.30 equiv) was azeotroped with PhMe (3x), then dissolved in 2.00 mL THF and cooled to 0 °C. Dropwise, NaHMDS (0.35 mL, 0.35 mmol, 1.30 equiv, 1M in THF) was added and the resulting bright orange solution stirred for 10 minutes, then cooled to -78 °C. Dropwise, a solution of **SI_31** (56 mg, 0.27 mmol, 1.00 equiv) in 1.0 mL THF (0.7 mL for dissolution, 3x 0.1 mL rinse) was added and the resulting solution stirred for 1 hour at -78 °C, then the cooling bath was removed and the solution slowly warmed to ambient temperature and further stirred for 1 hour. The reaction was then quenched by pouring into sat. NH_4Cl and the ensuing mixture was extracted 3x with EtOAc. The combined organic phases were dried over Na_2SO_4, filtered and evaporated to a crude. Purification by column chromatography (hexanes/EtOAc) gave desired product **SI_48** as a colorless oil (66 mg, 0.17 mmol, 63%).

^{1}H NMR (400 MHz, $CDCl_3$) δ 5.45 – 5.27 (m, 2H), 3.66 (s, 3H), 3.40 (t, J = 6.9 Hz, 2H), 2.43 (h, J = 7.0 Hz, 1H), 2.01 (th, J = 5.6, 2.9 Hz, 4H), 1.91 – 1.81 (m, 2H), 1.71 – 1.55 (m, 1H), 1.42 (qd, J = 6.2, 3.1 Hz, 2H), 1.38 – 1.23 (m, 17H), 1.14 (d, J = 7.0 Hz, 3H).

^{13}C NMR (101 MHz, $CDCl_3$) δ 177.6, 130.1, 129.9, 51.6, 39.6, 34.2, 34.0, 33.0, 29.9, 29.8, 29.6, 29.5, 29.4, 29.2, 28.8, 28.3, 27.38, 27.35, 27.27, 17.2.

HRMS *m/z* calculated for $C_{20}H_{38}BrO_2$ $[M+H]^+$: 389.2950, found: 389.2050.

bioRxiv preprint doi: https://doi.org/10.64898/2026.04.24.720406; this version posted April 27, 2026. The copyright holder for this preprint (which was not certified by peer review) is the author/funder, who has granted bioRxiv a license to display the preprint in perpetuity. It is made available under aCC-BY 4.0 International license.

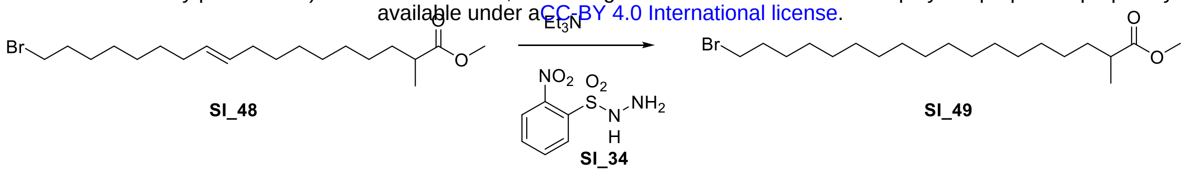

Methyl 18-bromo-2-methyloctadecanoate (SI_49): SI_48 (66 mg, 0.17 mmol, 1.00 equiv) was dissolved in 1.50 mL DCM. Et_3N (0.24 mL, 1.70 mmol, 10.0 equiv) and Hydrazide **SI_34** (184 mg, 0.85 mmol, 5.00 equiv) were added and the reaction left stirring overnight. The reaction was poured into sat $NaHCO_3$ and the ensuing mixture extracted 3x with DCM. The combined organic phases were dried over Na_2SO_4, filtered and evaporated to a crude. Purification by column chromatography gave the desired product **SI_49** as a colorless oil (52 mg, 0.13 mmol, 78%).

^{1}H NMR (400 MHz, $CDCl_3$) δ 3.65 (s, 3H), 3.39 (t, *J* = 6.9 Hz, 2H), 2.42 (h, *J* = 7.0 Hz, 1H), 1.84 (dt, *J* = 14.6, 7.0 Hz, 2H), 1.72 – 1.56 (m, 1H), 1.39 (tq, *J* = 13.0, 6.6 Hz, 3H), 1.25 (d, *J* = 3.5 Hz, 24H), 1.13 (d, *J* = 6.9 Hz, 3H).

^{13}C NMR (101 MHz, $CDCl_3$) δ 177.5, 51.6, 39.6, 34.2, 34.0, 33.0, 29.79, 29.77, 29.75, 29.72, 29.68, 29.65, 29.62, 29.57, 28.9, 28.3, 27.4, 17.2.

HRMS *m/z* calculated for $C_{20}H_{40}BrO_2$ $[M+H]^+$: 391.2206, found: 391.2204.

*Note: Hydrazide **SI_34** was prepared according to J. Am. Chem. Soc. **2017**, 139, 15636.*

bioRxiv preprint doi: https://doi.org/10.64898/2026.04.24.720406; this version posted April 27, 2026. The copyright holder for this preprint (which was not certified by peer review) is the author/funder, who has granted bioRxiv a license to display the preprint in perpetuity. It is made available under aCC-BY 4.0 International license.

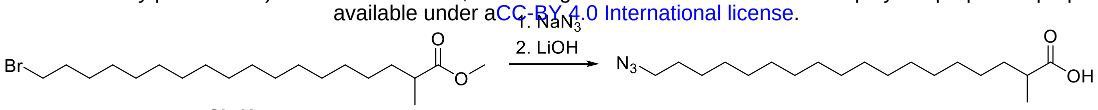

SI_48 cl-2-MeSA

18-Azido-2-methyloctadecanoic acid (cl-2-MeSA): SI_48 (52 mg, 0.133 mmol, 1.00 equiv) was dissolved in 0.65 mL DMF, followed by the addition of NaN_3 (43.2 mg, 0.66 mmol, 5.00 equiv). The reaction was stirred for 24 hours, then poured into sat. $NaHCO_3$ and the mixture extracted 3x with EtOAc. The combined organic phases were dried over Na_2SO_4, filtered and evaporated to a crude, which was directly dissolved in a mixture of 1.0 mL THF, 0.4 mL MeOH, 0.2 mL water and cooled to 0 °C. $LiOH·H_2O$ (16.7 mg, 0.40 mmol, 3.00 equiv) was added and the reaction allowed to warm to ambient temperature and stirred for 4 hours. The reaction was then poured into 1 M HCl and the mixture extracted 5x with DCM. The combined organic phases were dried over Na_2SO_4, filtered and evaporated to a crude, which was purified by crystallization from hexanes to give **cl-2-MeSA** as a white solid (19 mg, 0.056 mmol, 42% over two steps).

^{1}H NMR (400 MHz, $CDCl_3$) δ 3.25 (t, J = 7.0 Hz, 2H), 2.46 (h, J = 6.9 Hz, 1H), 1.73 – 1.53 (m, 3H), 1.45 – 1.22 (m, 28H), 1.17 (d, J = 6.9 Hz, 3H).

^{13}C NMR (101 MHz, $CDCl_3$) δ 182.6, 51.7, 33.7, 29.81, 29.77, 29.75, 29.70, 29.67, 29.63, 29.3, 29.0, 27.3, 26.9, 17.0.

HRMS *m/z* calculated for $C_{19}H_{36}N_3O_2$ $[M-H]^-$: 338.2813, found: 338.2830.

bioRxiv preprint doi: https://doi.org/10.64898/2026.04.24.720406; this version posted April 27, 2026. The copyright holder for this preprint (which was not certified by peer review) is the author/funder, who has granted bioRxiv a license to display the preprint in perpetuity. It is made available under aCC-BY 4.0 International license.

NMR Data

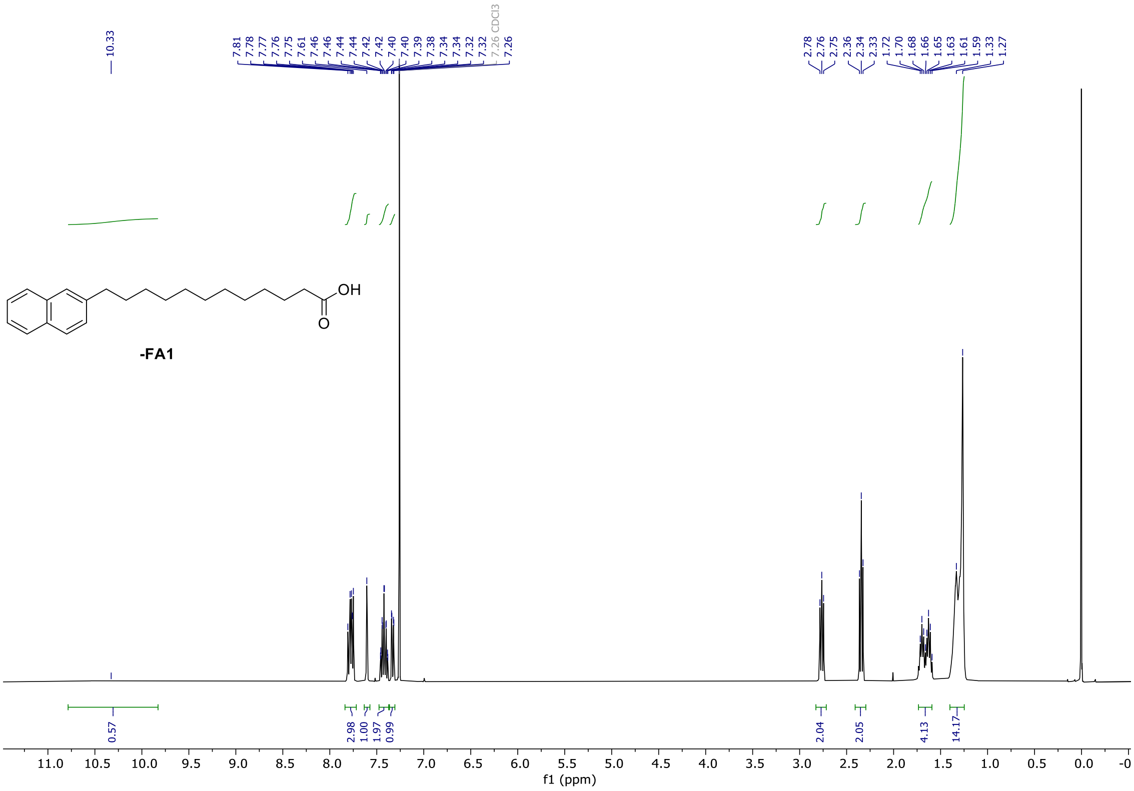

^{1}H NMR (400 MHz, $CDCl_3$) of **π-FA1**.

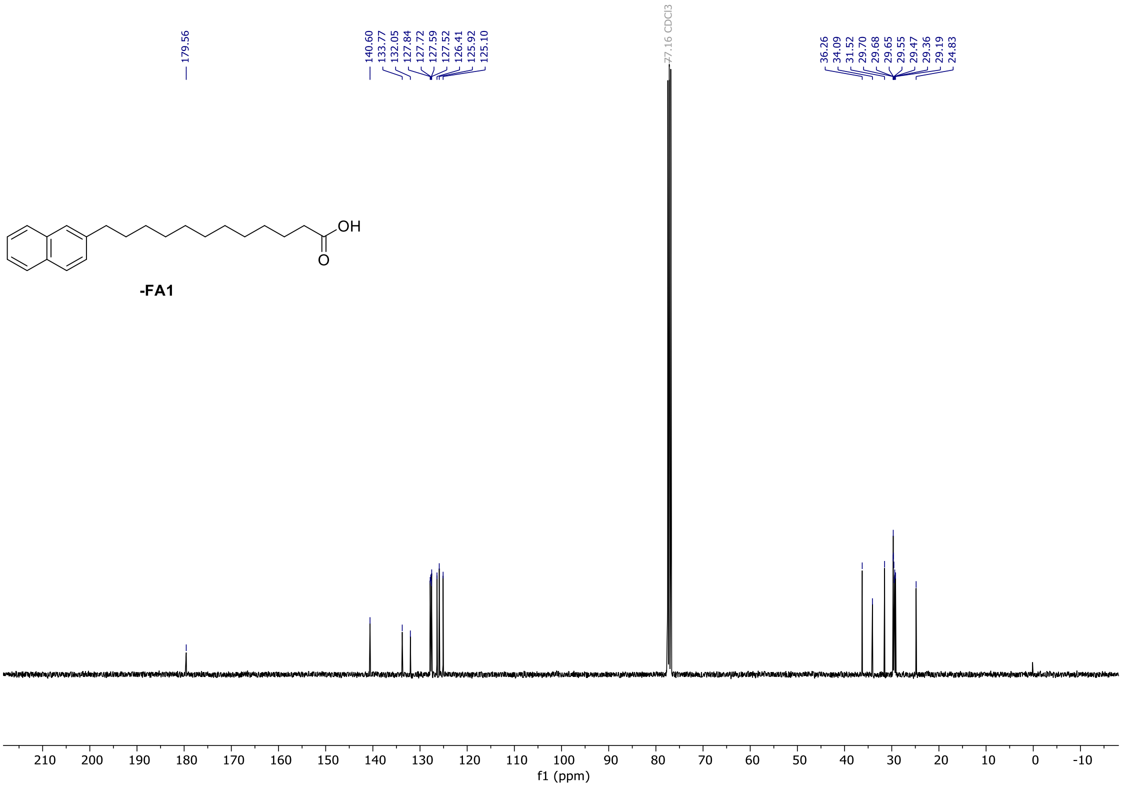

bioRxiv preprint doi: https://doi.org/10.64898/2026.04.24.720406; this version posted April 27, 2026. The copyright holder for this preprint (which was not certified by peer review) is the author/funder, who has granted bioRxiv a license to display the preprint in perpetuity. It is made available under aCC-BY 4.0 International license.

^{13}C NMR (101 MHz, $CDCl_3$) of ***π*-FAT**.

bioRxiv preprint doi: https://doi.org/10.64898/2026.04.24.720406; this version posted April 27, 2026. The copyright holder for this preprint (which was not certified by peer review) is the author/funder, who has granted bioRxiv a license to display the preprint in perpetuity. It is made available under aCC-BY 4.0 International license.

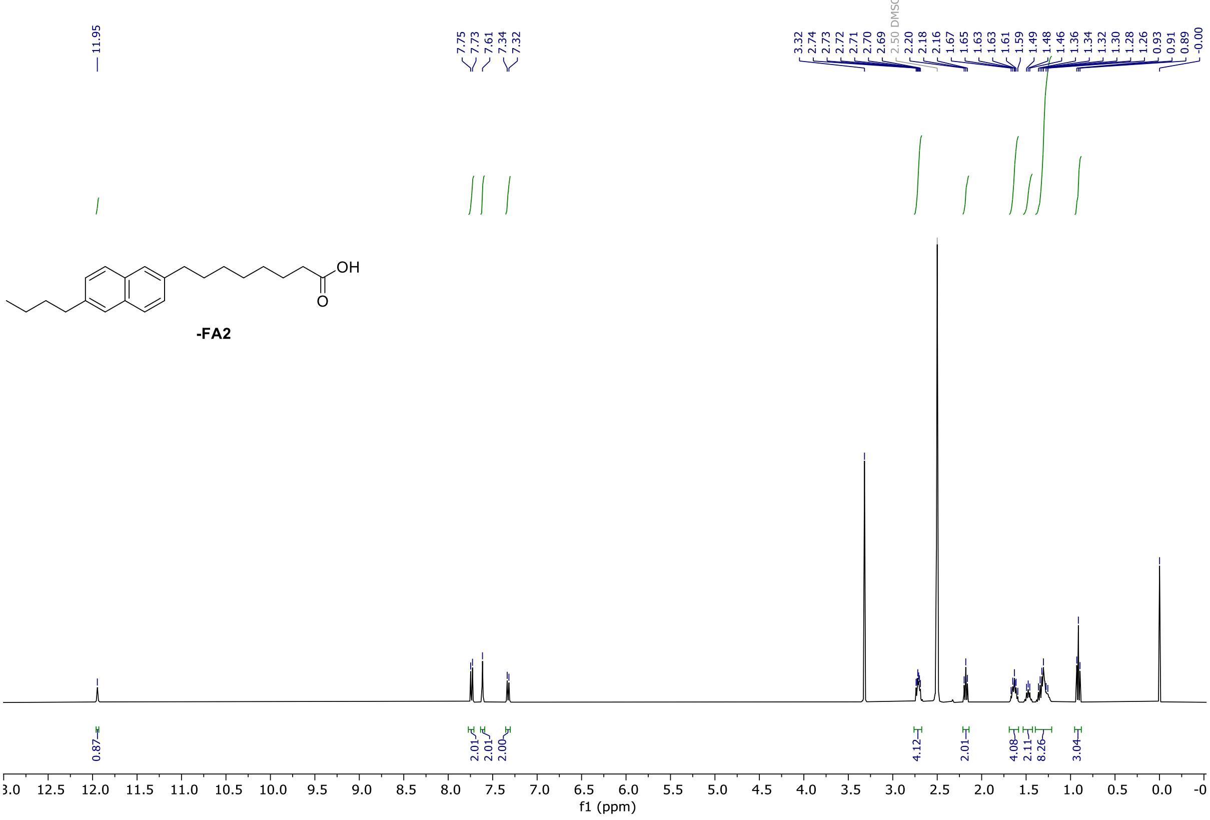

^{1}**H NMR** (400 MHz, $CDCl_3$) of **π-FA2**.

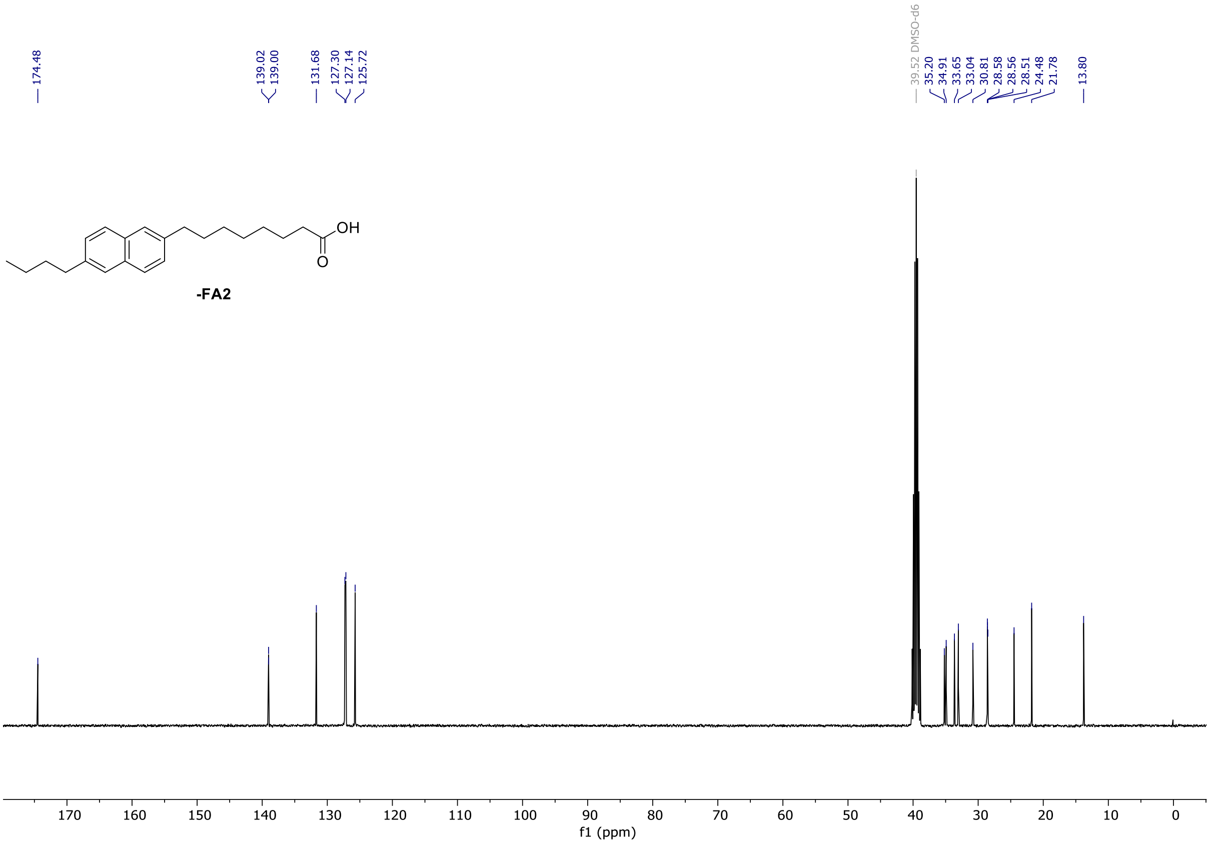

^{13}**C NMR** (101 MHz, $CDCl_3$) of **π-FA2**.

bioRxiv preprint doi: https://doi.org/10.64898/2026.04.24.720406; this version posted April 27, 2026. The copyright holder for this preprint (which was not certified by peer review) is the author/funder, who has granted bioRxiv a license to display the preprint in perpetuity. It is made available under aCC-BY 4.0 International license.

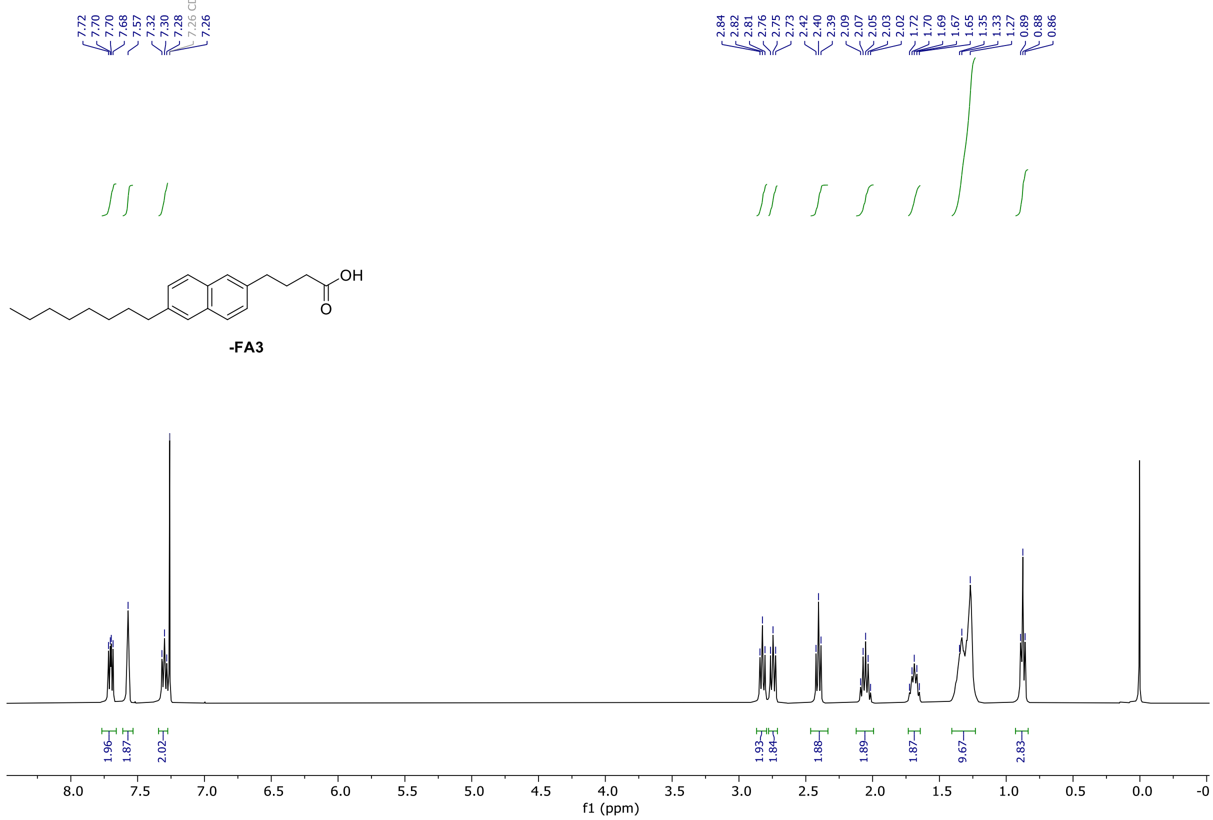

^{1}H NMR (400 MHz, $CDCl_3$) of **π-FA3**.

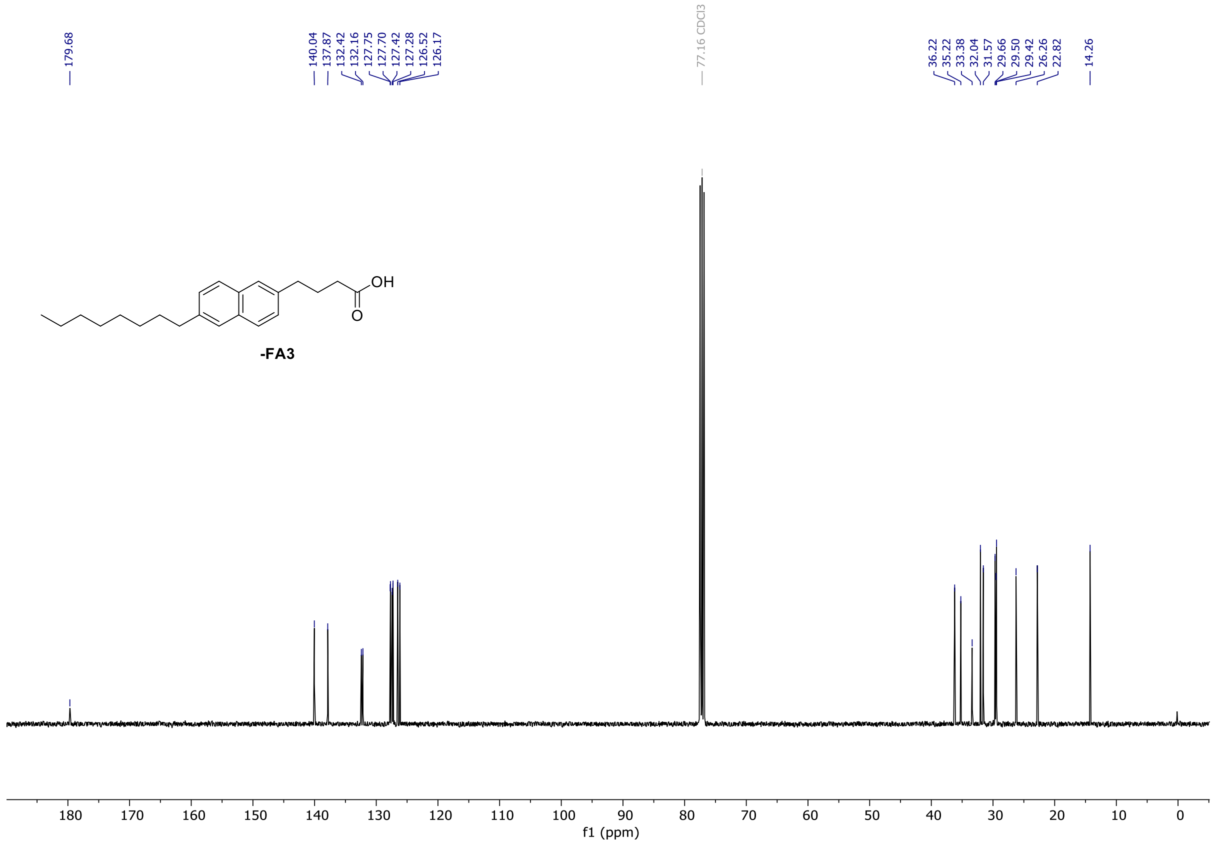

^{13}C NMR (101 MHz, $CDCl_3$) of **π-FA3**.

bioRxiv preprint doi: https://doi.org/10.64898/2026.04.24.720406; this version posted April 27, 2026. The copyright holder for this preprint (which was not certified by peer review) is the author/funder, who has granted bioRxiv a license to display the preprint in perpetuity. It is made available under aCC-BY 4.0 International license.

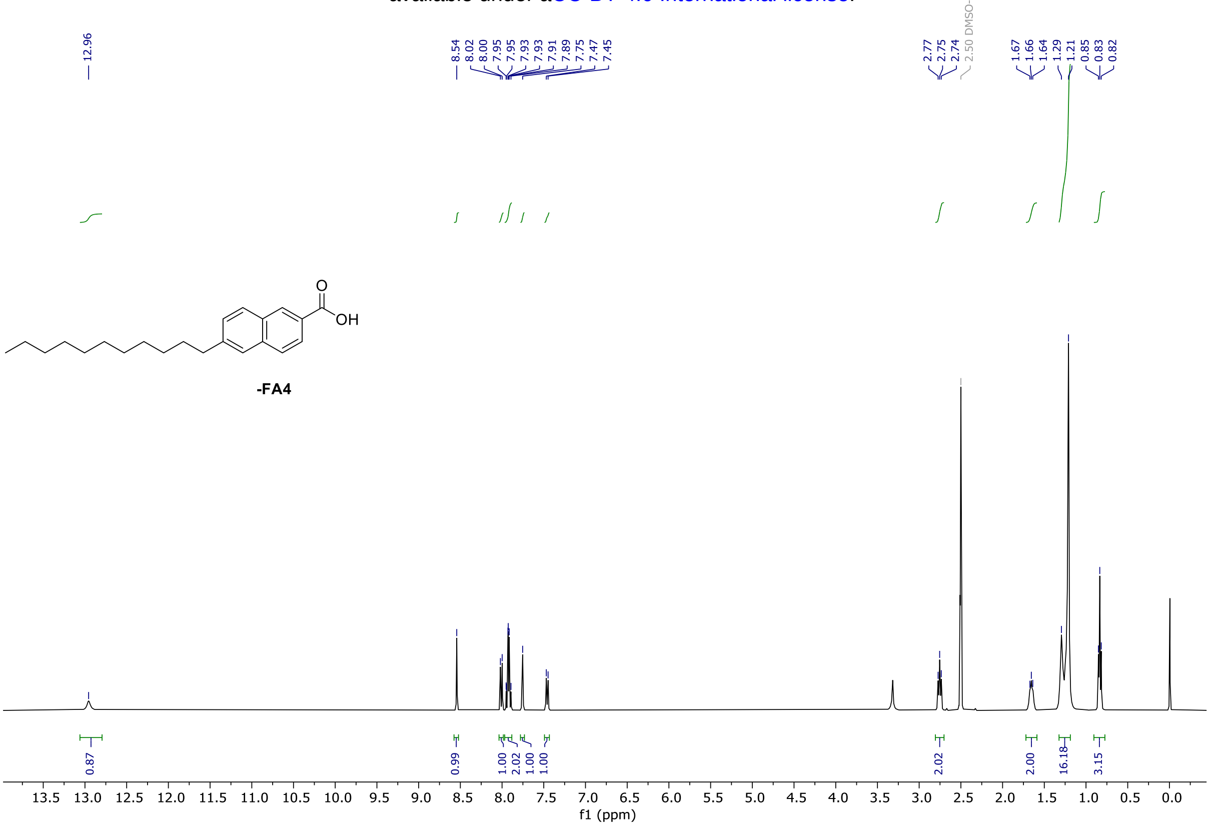

^{1}H NMR (400 MHz, $CDCl_3$) of **π-FA4**.

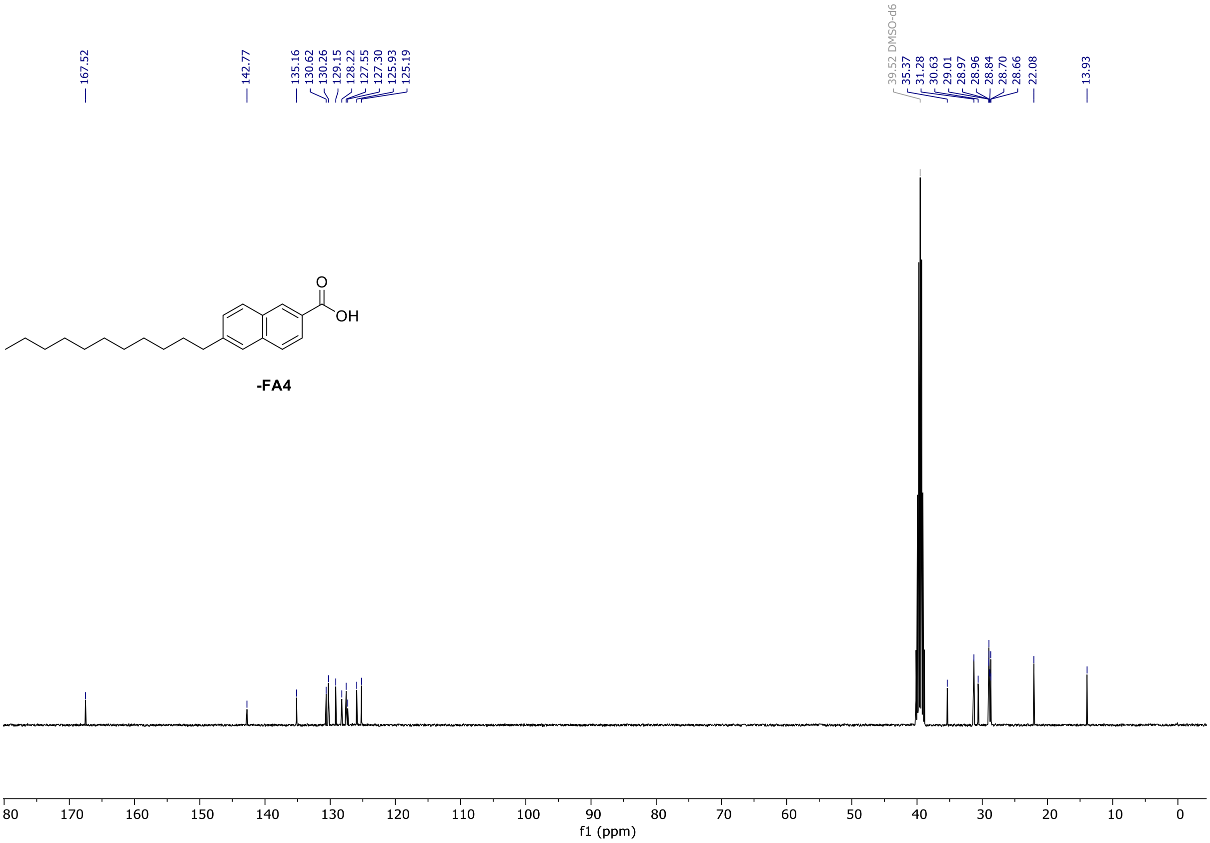

^{13}C NMR (101 MHz, $CDCl_3$) of **π-FA4**.

bioRxiv preprint doi: https://doi.org/10.64898/2026.04.24.720406; this version posted April 27, 2026. The copyright holder for this preprint (which was not certified by peer review) is the author/funder, who has granted bioRxiv a license to display the preprint in perpetuity. It is made available under aCC-BY 4.0 International license.

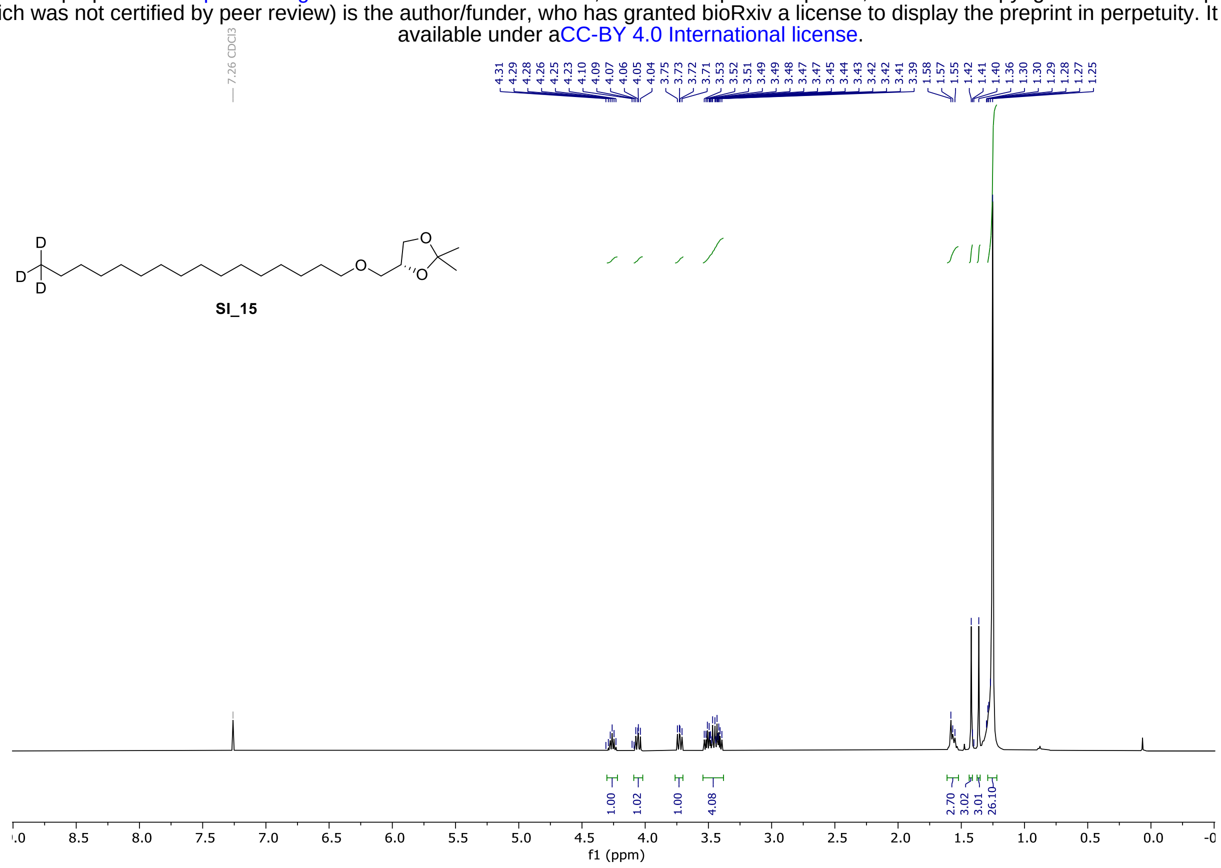

^{1}H NMR (400 MHz, CDCl$_3$) of **SI_15**.

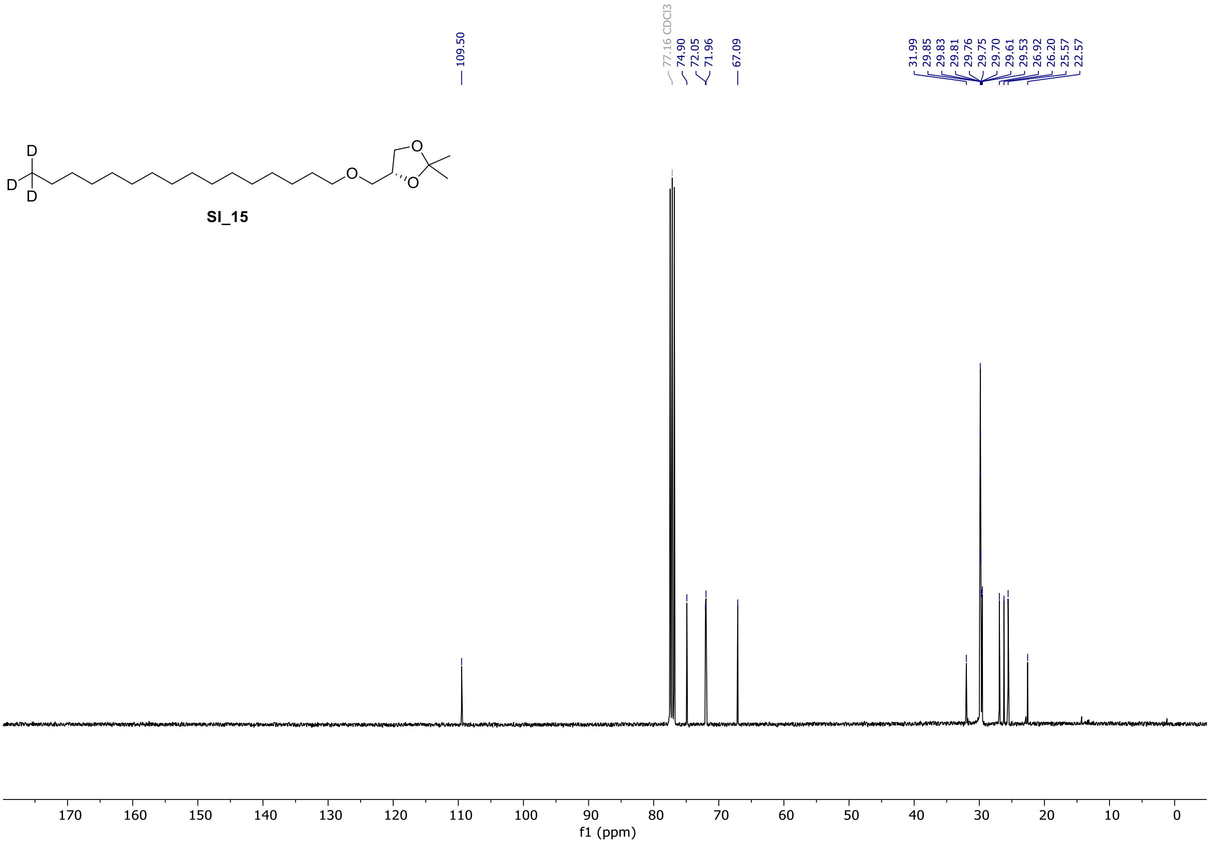

^{13}C NMR (101 MHz, CDCl$_3$) of **SI_15**.

bioRxiv preprint doi: https://doi.org/10.64898/2026.04.24.720406; this version posted April 27, 2026. The copyright holder for this preprint (which was not certified by peer review) is the author/funder, who has granted bioRxiv a license to display the preprint in perpetuity. It is made available under aCC-BY 4.0 International license.

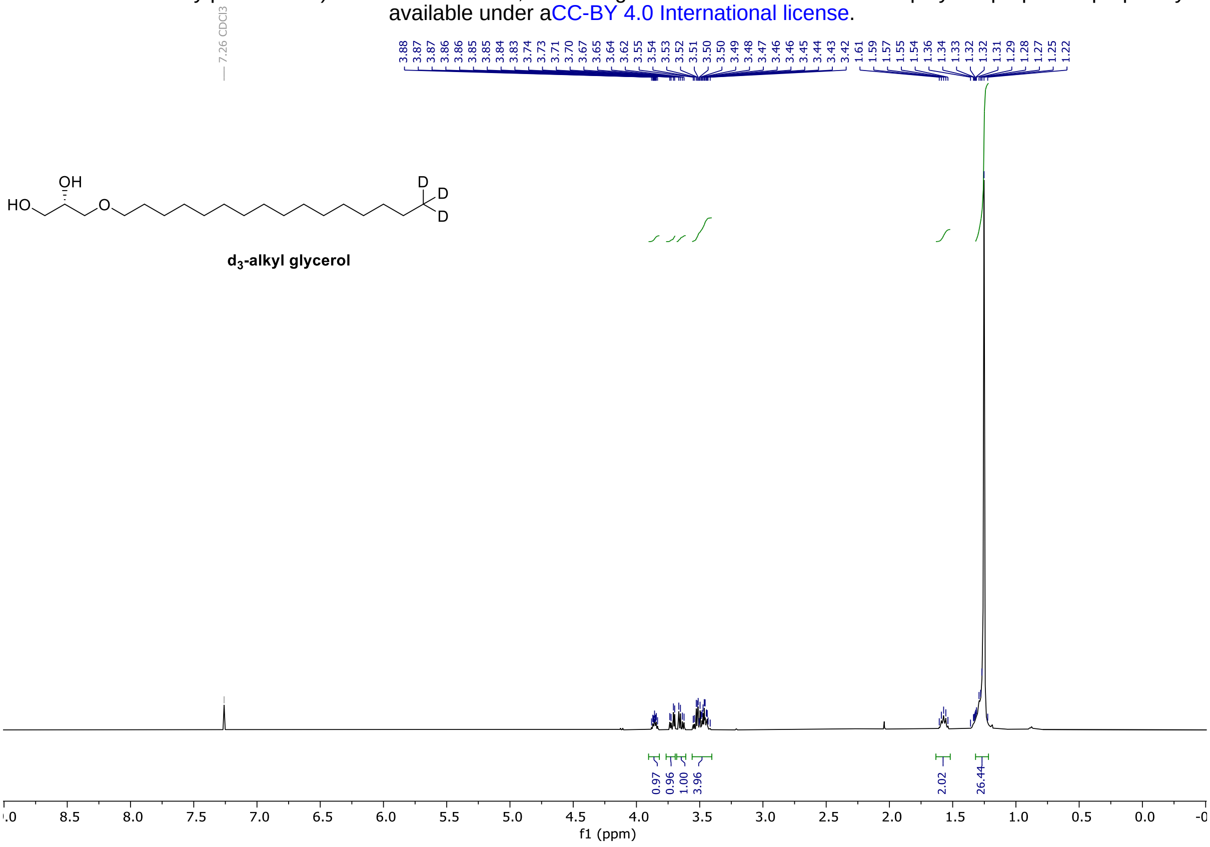

^{1}H NMR (400 MHz, $CDCl_3$) of **d_3-alkyl glycerol**.

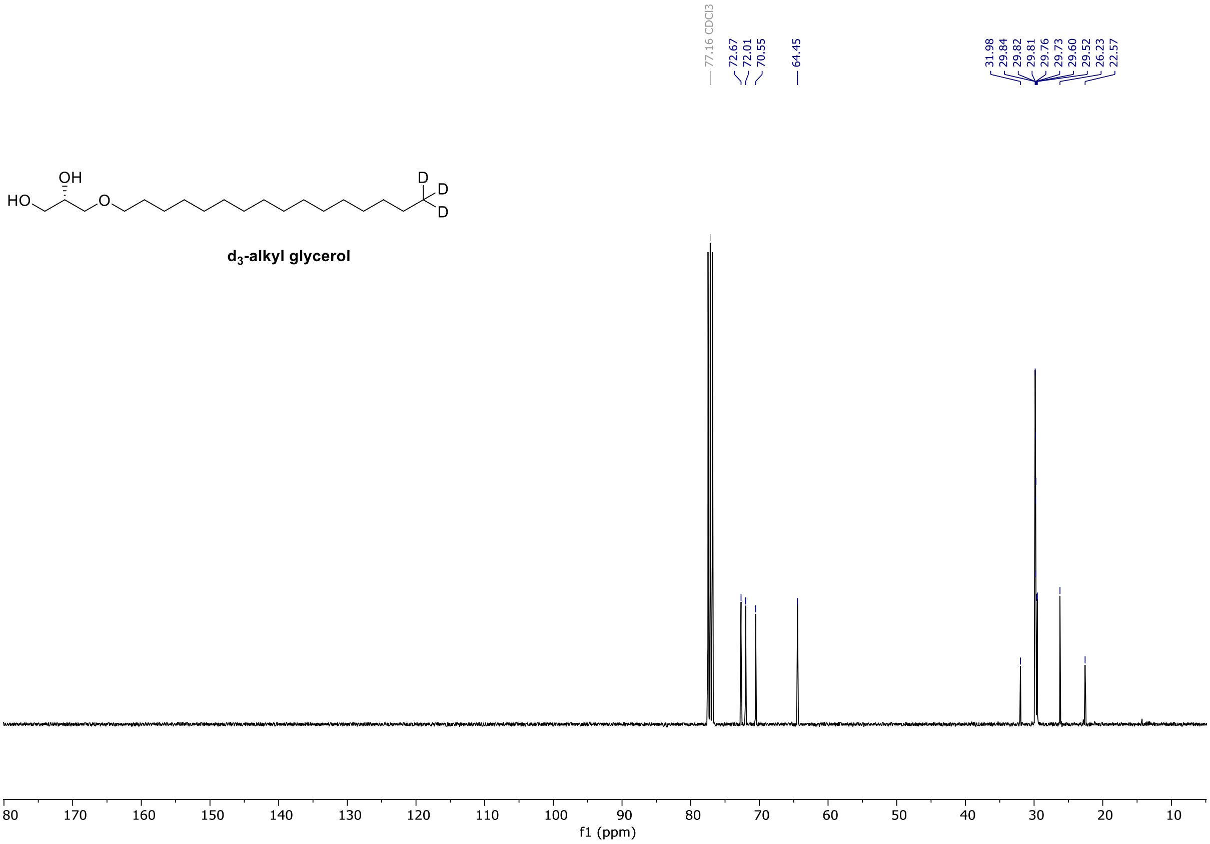

^{13}C NMR (101 MHz, $CDCl_3$) of **d_3-alkyl glycerol**.

bioRxiv preprint doi: https://doi.org/10.64898/2026.04.24.720406; this version posted April 27, 2026. The copyright holder for this preprint (which was not certified by peer review) is the author/funder, who has granted bioRxiv a license to display the preprint in perpetuity. It is made available under aCC-BY 4.0 International license.

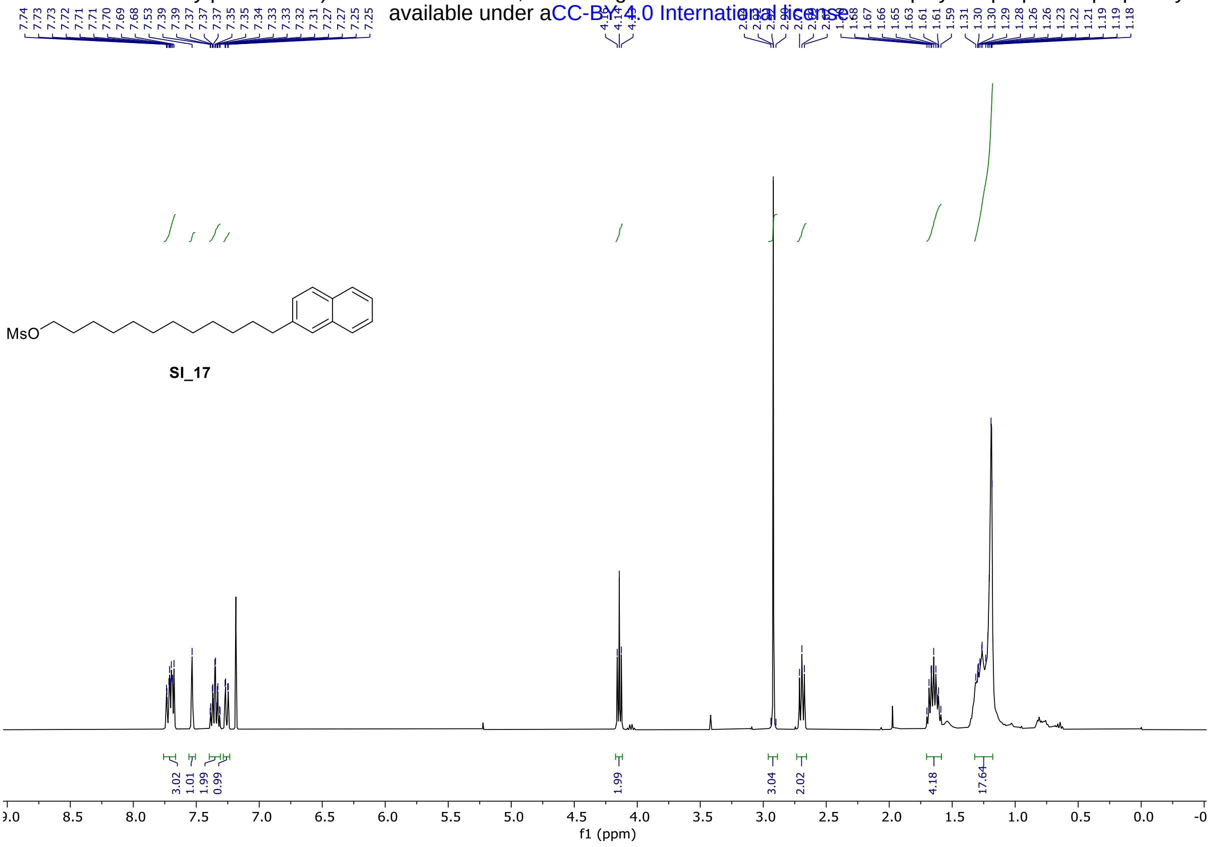

^{1}H NMR (400 MHz, $CDCl_3$) of **SI_17**.

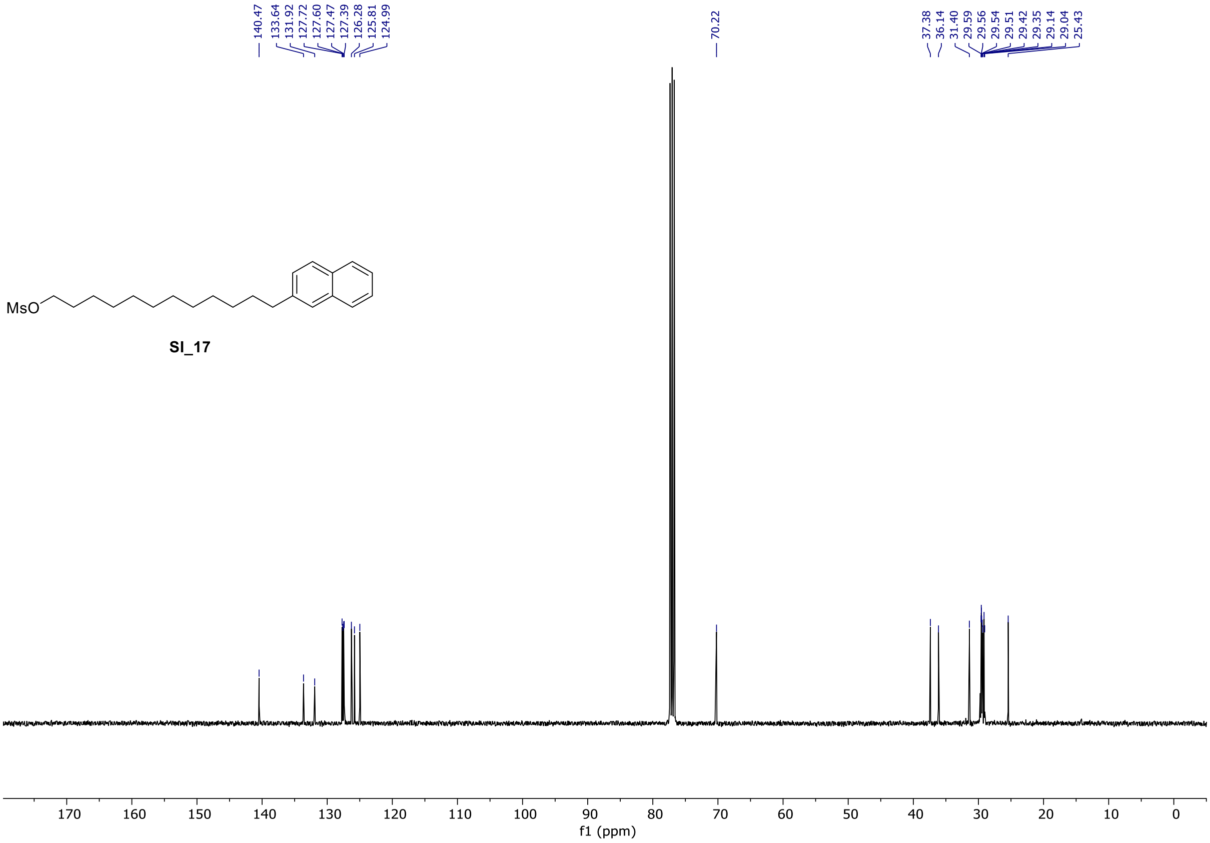

^{13}C NMR (101 MHz, $CDCl_3$) of **SI_17**.

bioRxiv preprint doi: https://doi.org/10.64898/2026.04.24.720406; this version posted April 27, 2026. The copyright holder for this preprint (which was not certified by peer review) is the author/funder, who has granted bioRxiv a license to display the preprint in perpetuity. It is made available under aCC-BY 4.0 International license.

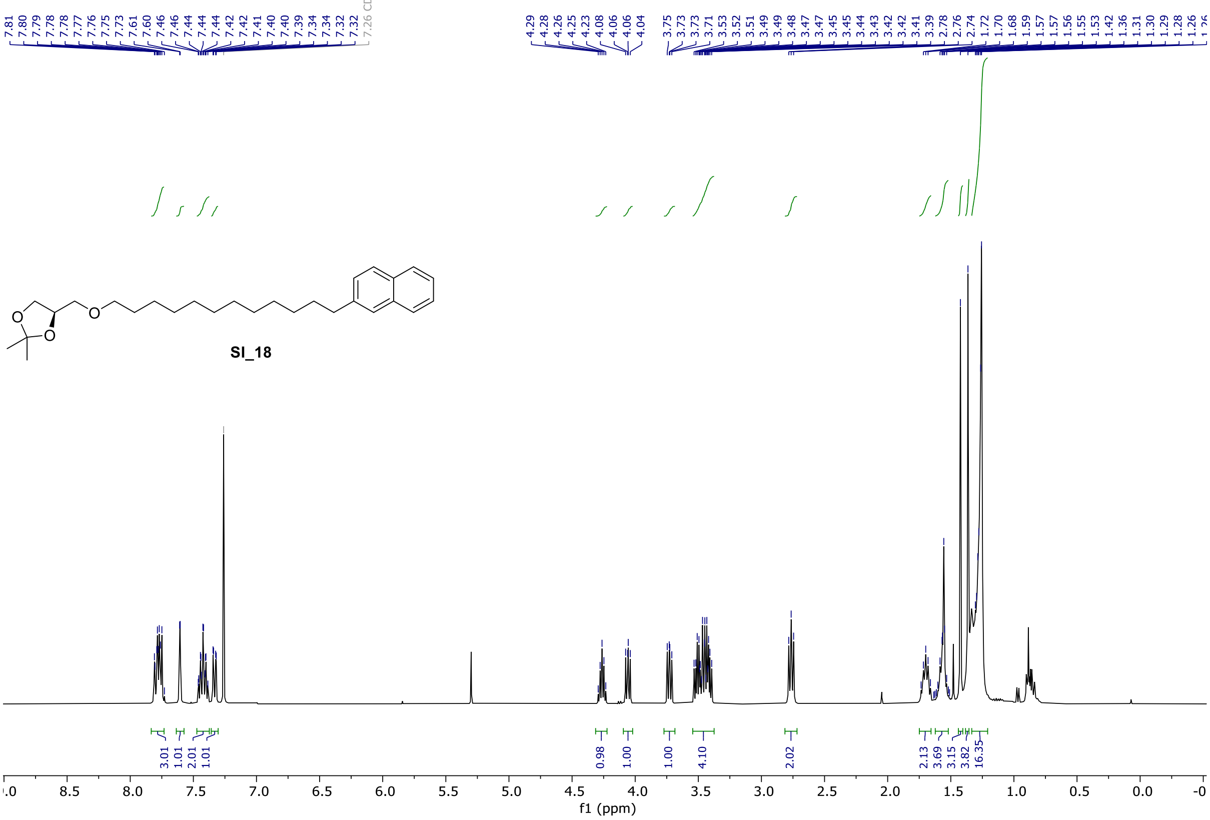

^{1}H NMR (400 MHz, $CDCl_3$) of **SI_18**.

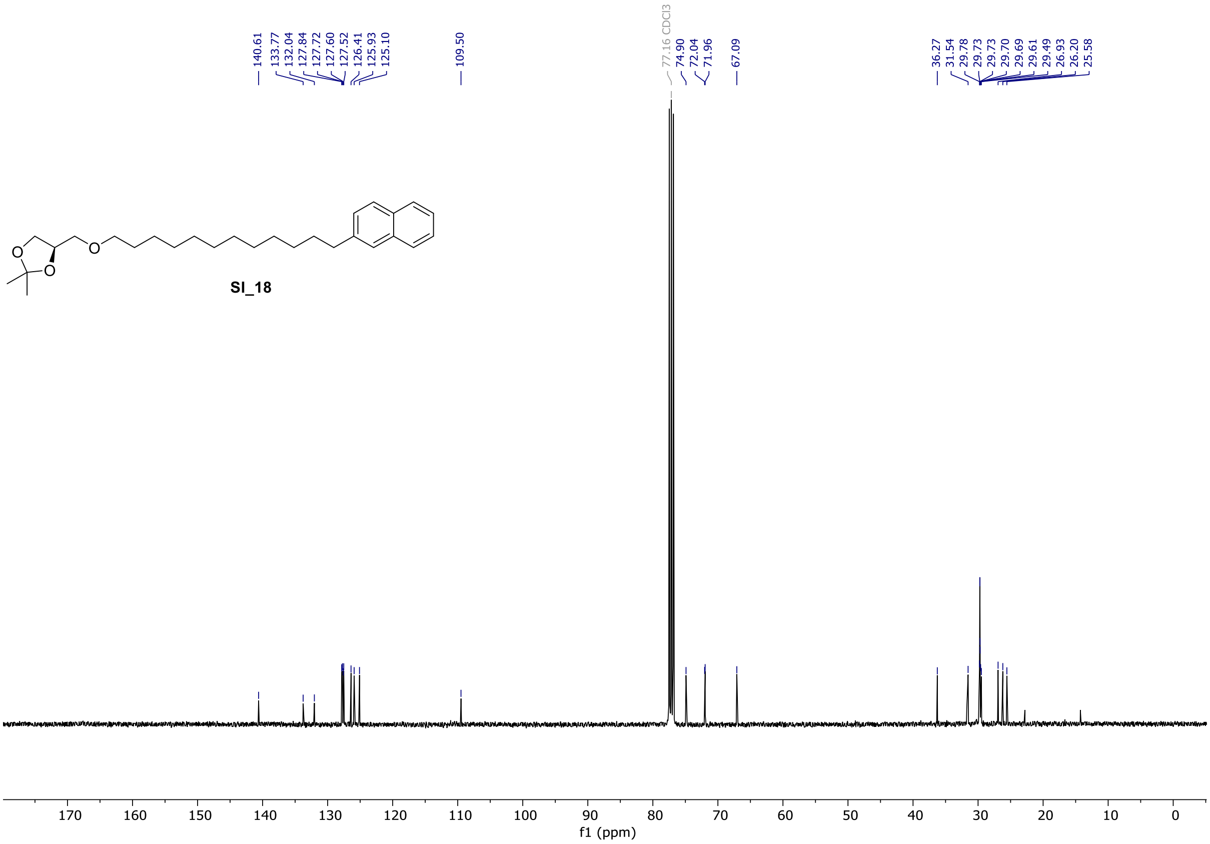

^{13}C NMR (101 MHz, $CDCl_3$) of **SI_18**.

bioRxiv preprint doi: https://doi.org/10.64898/2026.04.24.720406; this version posted April 27, 2026. The copyright holder for this preprint (which was not certified by peer review) is the author/funder, who has granted bioRxiv a license to display the preprint in perpetuity. It is made available under aCC-BY 4.0 International license.

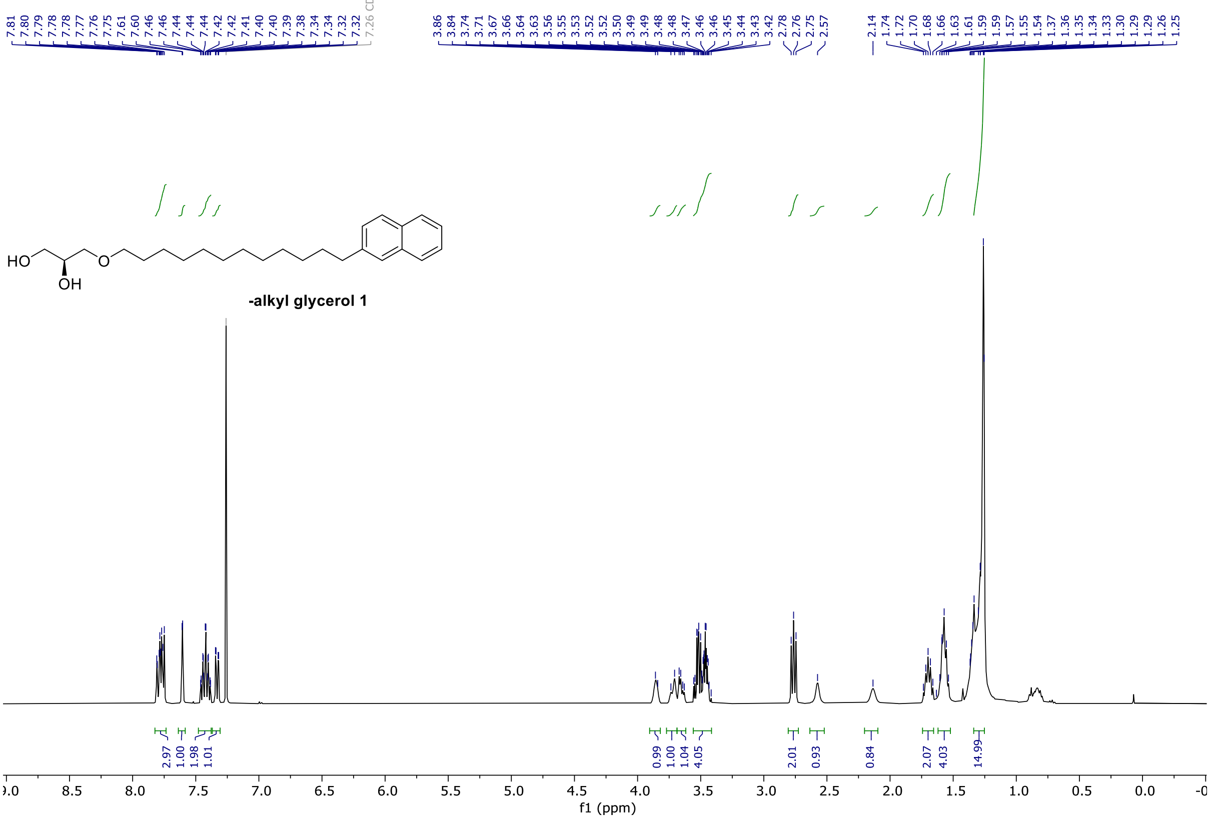

^{1}H NMR (400 MHz, $CDCl_3$) of **π-alkyl glycerol 1**.

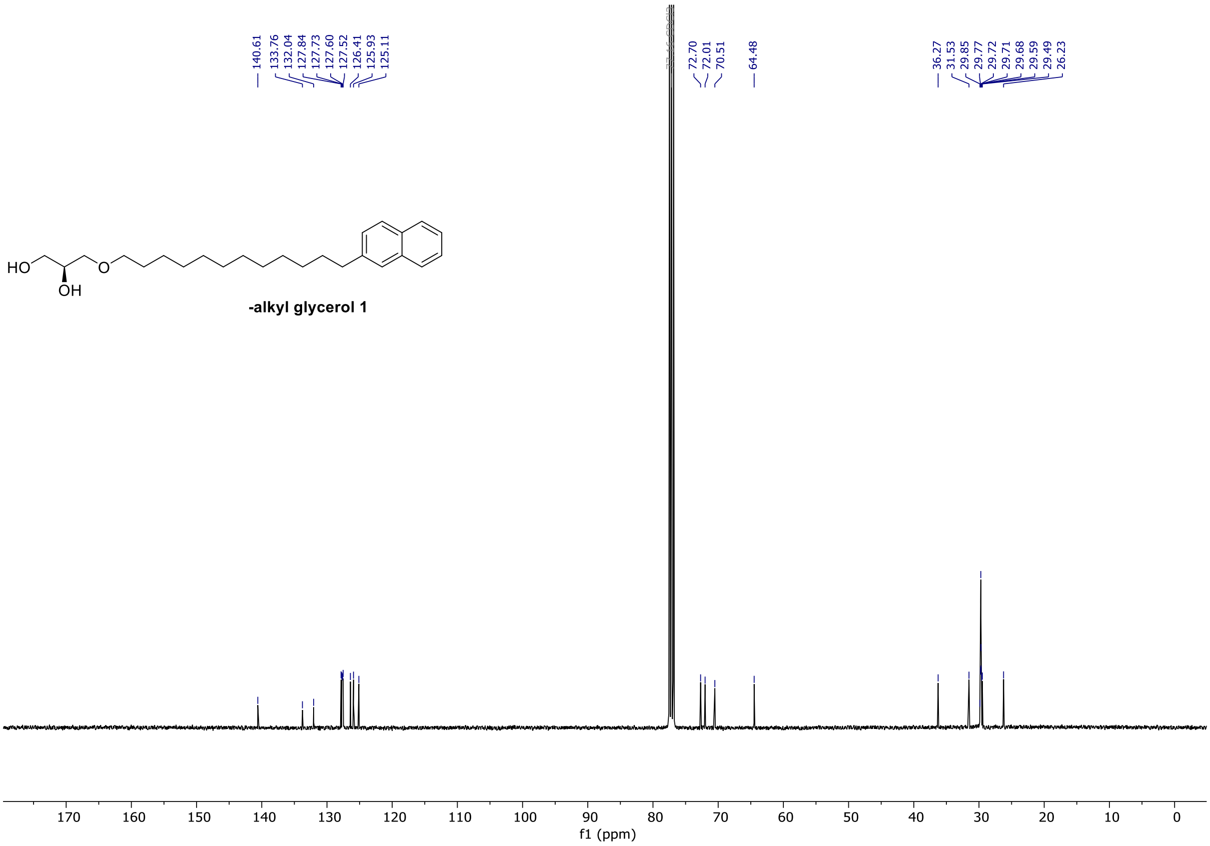

^{13}C NMR (101 MHz, $CDCl_3$) of **π-alkyl glycerol 1**.

bioRxiv preprint doi: https://doi.org/10.64898/2026.04.24.720406; this version posted April 27, 2026. The copyright holder for this preprint (which was not certified by peer review) is the author/funder, who has granted bioRxiv a license to display the preprint in perpetuity. It is made available under aCC-BY 4.0 International license.

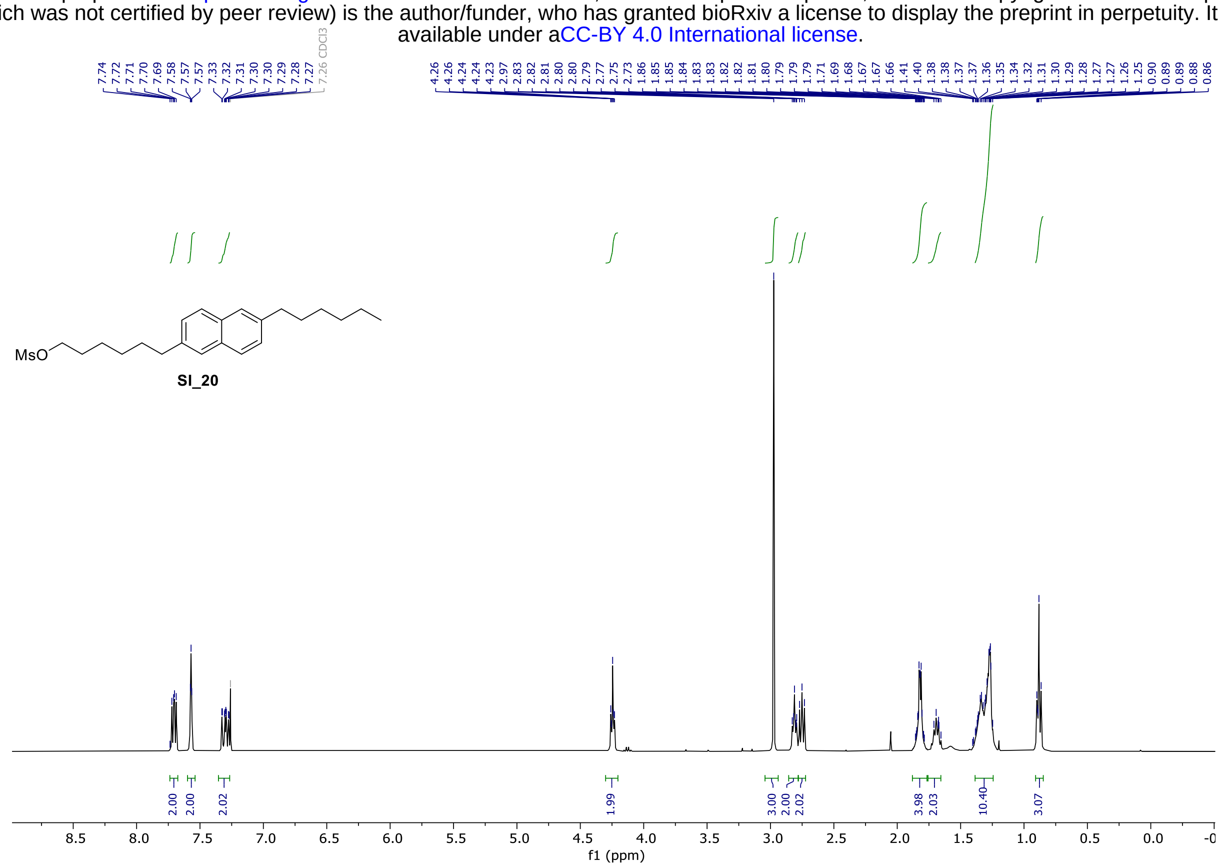

^{1}H NMR (400 MHz, $CDCl_3$) of **SI_20**.

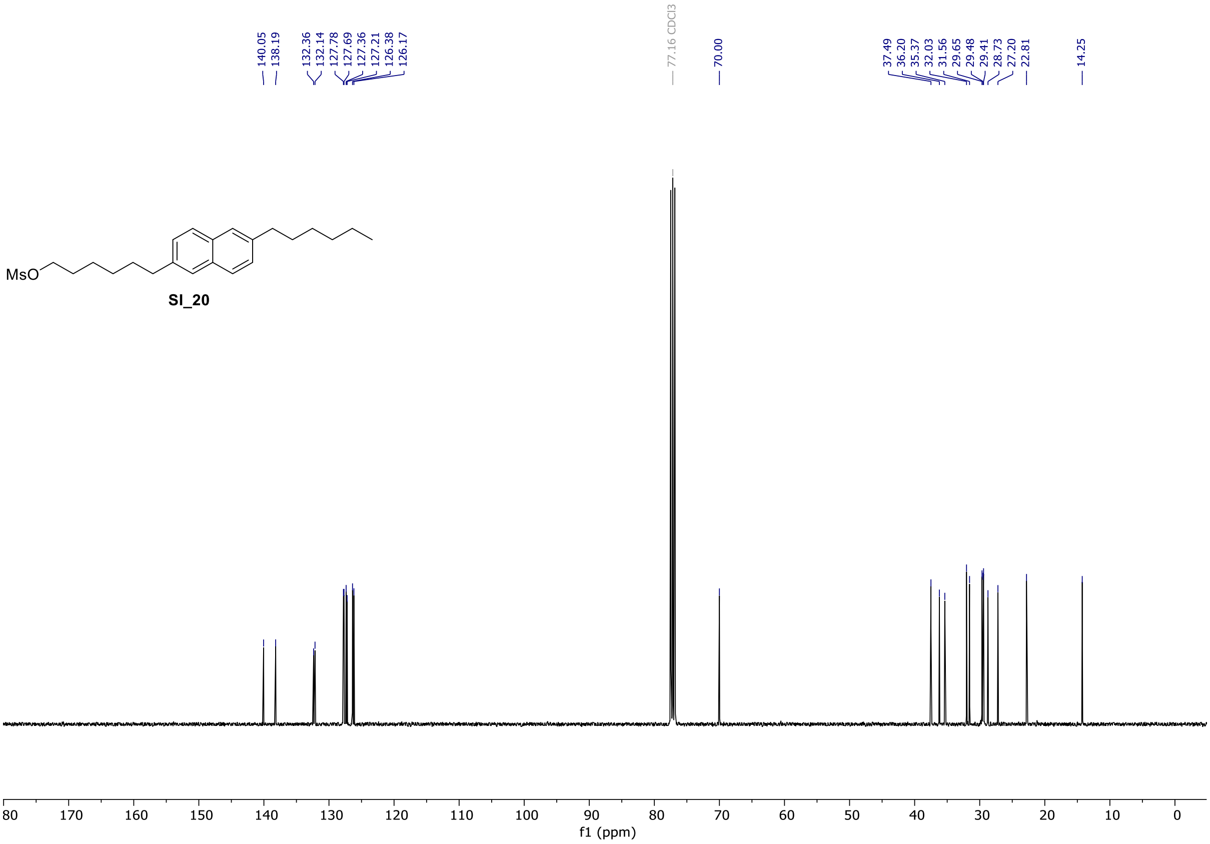

^{13}C NMR (101 MHz, $CDCl_3$) of **SI_20**.

bioRxiv preprint doi: https://doi.org/10.64898/2026.04.24.720406; this version posted April 27, 2026. The copyright holder for this preprint (which was not certified by peer review) is the author/funder, who has granted bioRxiv a license to display the preprint in perpetuity. It is made available under aCC-BY 4.0 International license.

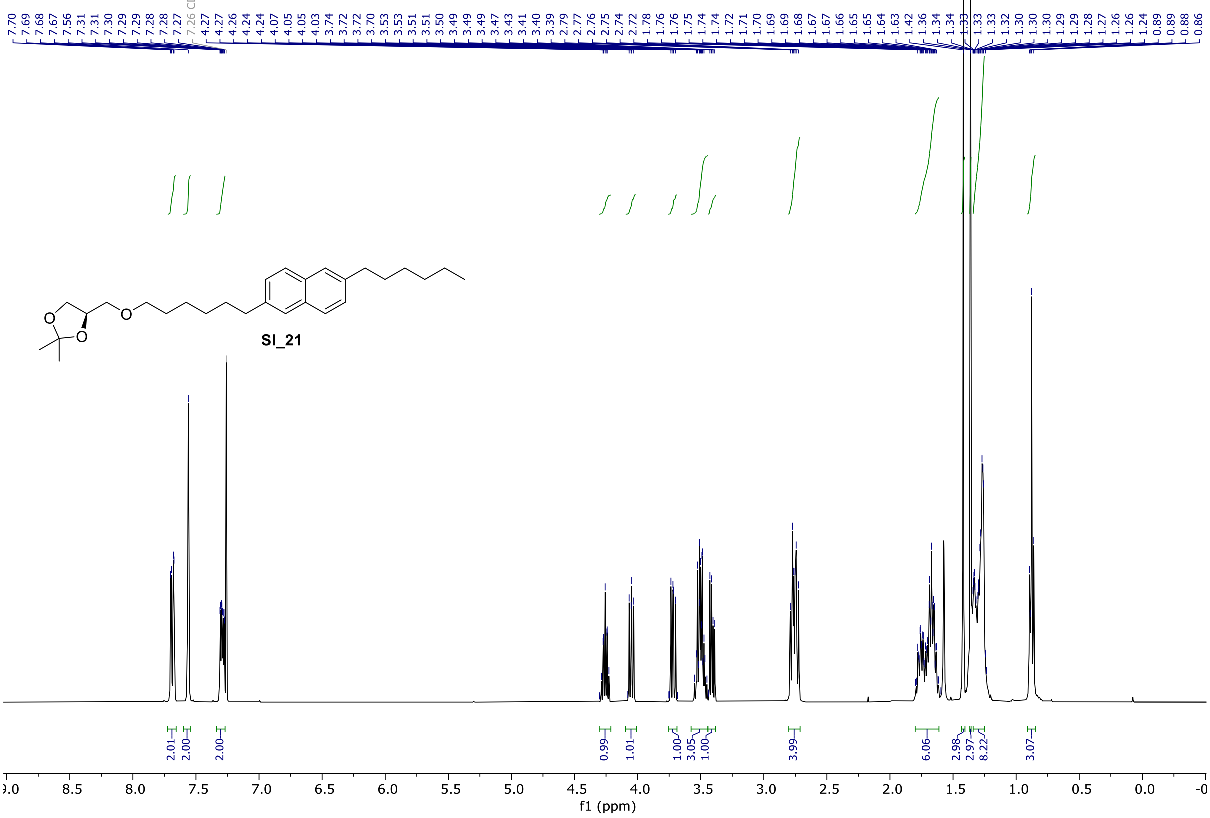

^{1}H NMR (400 MHz, $CDCl_3$) of **SI_21**.

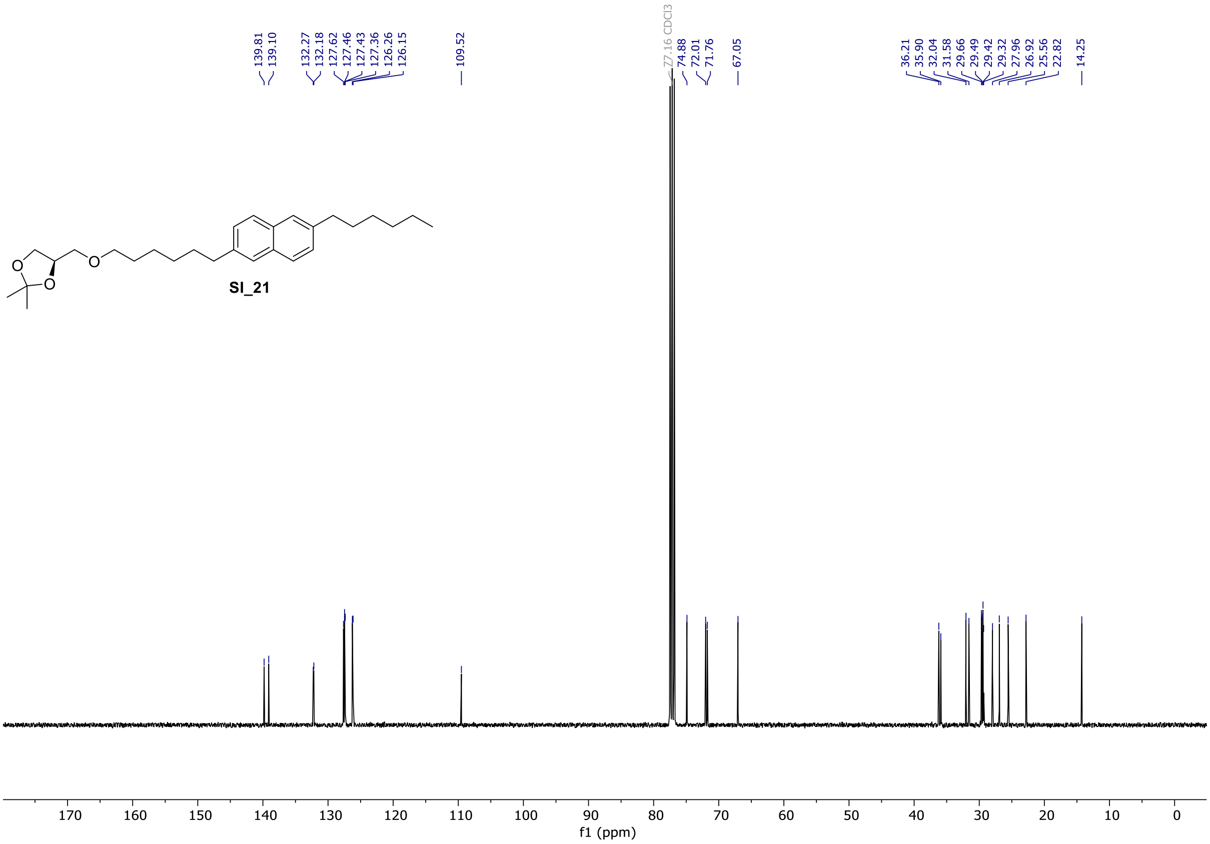

^{13}C NMR (101 MHz, $CDCl_3$) of **SI_21**.

bioRxiv preprint doi: https://doi.org/10.64898/2026.04.24.720406; this version posted April 27, 2026. The copyright holder for this preprint (which was not certified by peer review) is the author/funder, who has granted bioRxiv a license to display the preprint in perpetuity. It is made available under aCC-BY 4.0 International license.

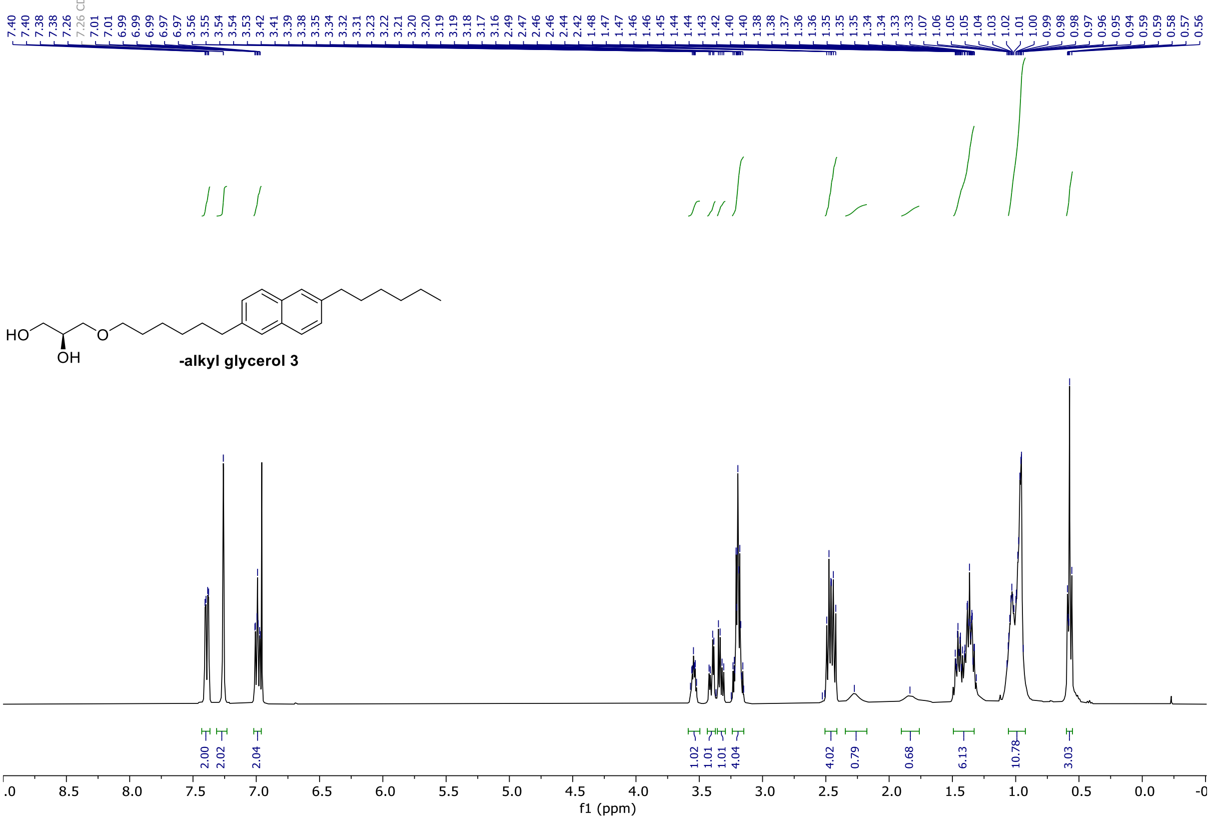

^{1}**H NMR** (400 MHz, $CDCl_3$) of **π-alkyl glycerol 3**.

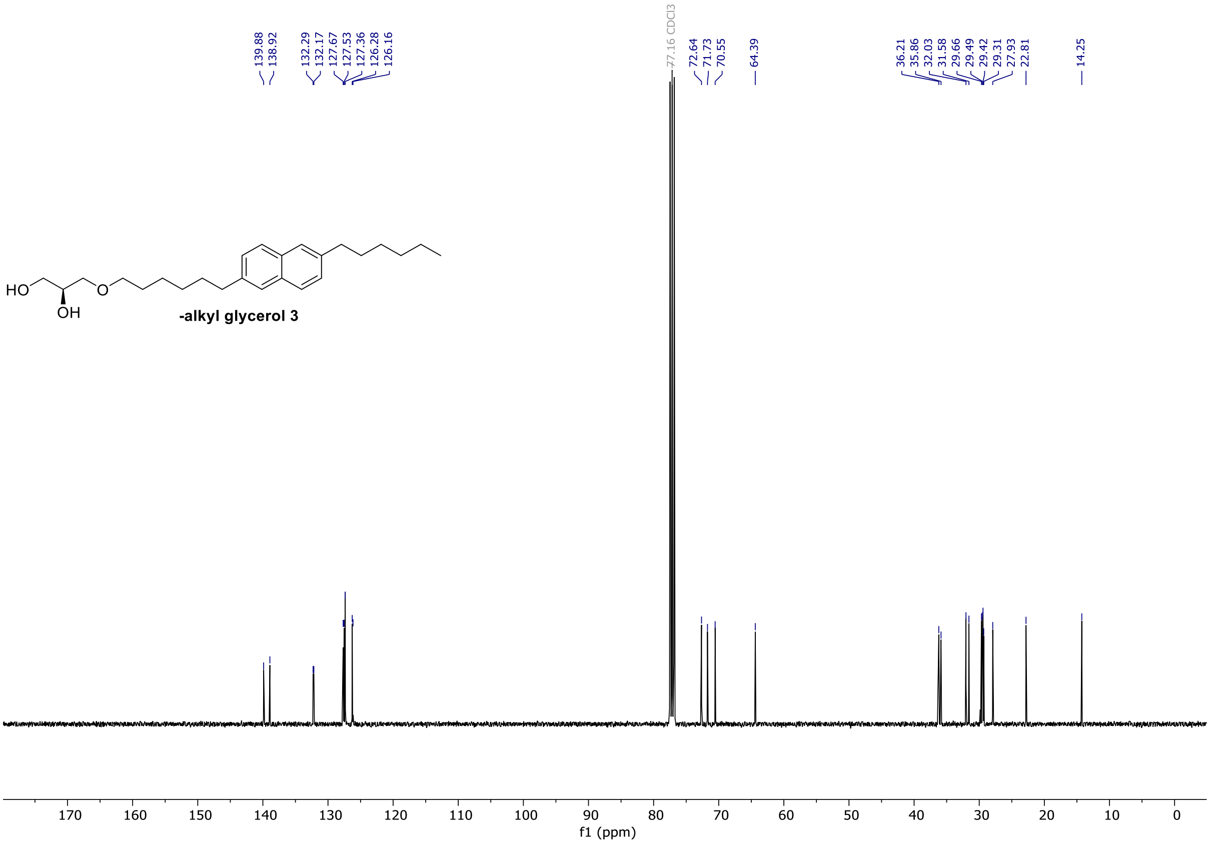

^{13}**C NMR** (101 MHz, $CDCl_3$) of **π-alkyl glycerol 3**.

bioRxiv preprint doi: https://doi.org/10.64898/2026.04.24.720406; this version posted April 27, 2026. The copyright holder for this preprint (which was not certified by peer review) is the author/funder, who has granted bioRxiv a license to display the preprint in perpetuity. It is made available under aCC-BY 4.0 International license.

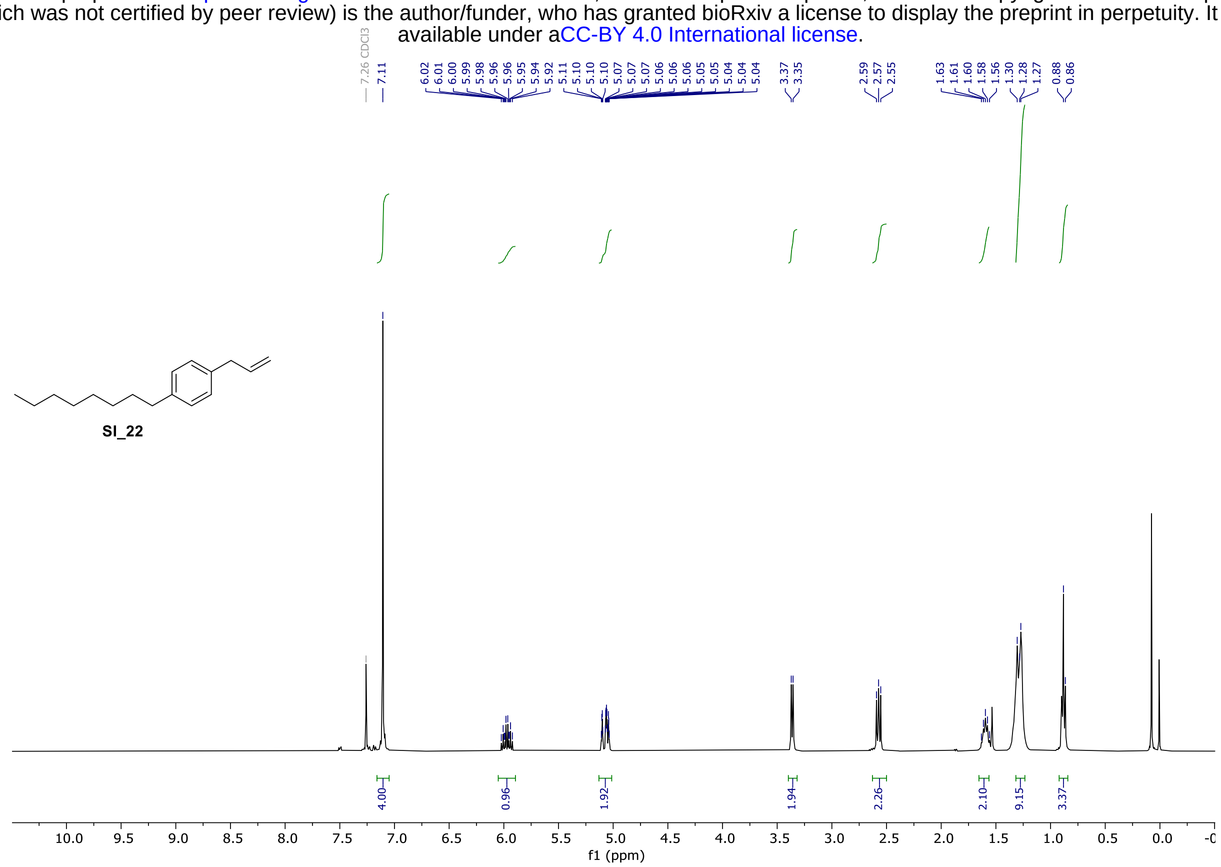

^{1}H NMR (400 MHz, $CDCl_3$) of **SI_22**.

bioRxiv preprint doi: https://doi.org/10.64898/2026.04.24.720406; this version posted April 27, 2026. The copyright holder for this preprint (which was not certified by peer review) is the author/funder, who has granted bioRxiv a license to display the preprint in perpetuity. It is made available under aCC-BY 4.0 International license.

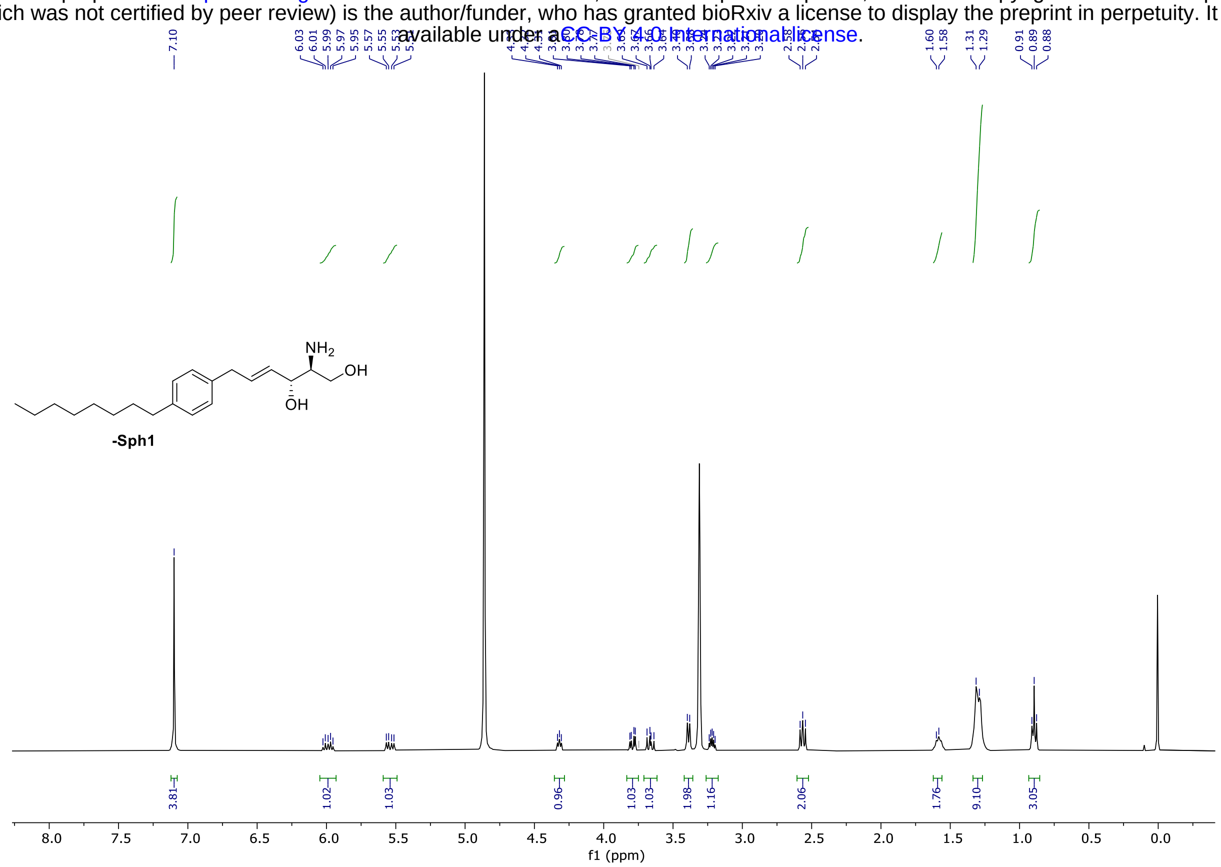

^{1}H NMR (400 MHz, MeOH-d$_4$) of **π-Sph-1**.

bioRxiv preprint doi: https://doi.org/10.64898/2026.04.24.720406; this version posted April 27, 2026. The copyright holder for this preprint (which was not certified by peer review) is the author/funder, who has granted bioRxiv a license to display the preprint in perpetuity. It is made available under aCC-BY 4.0 International license.

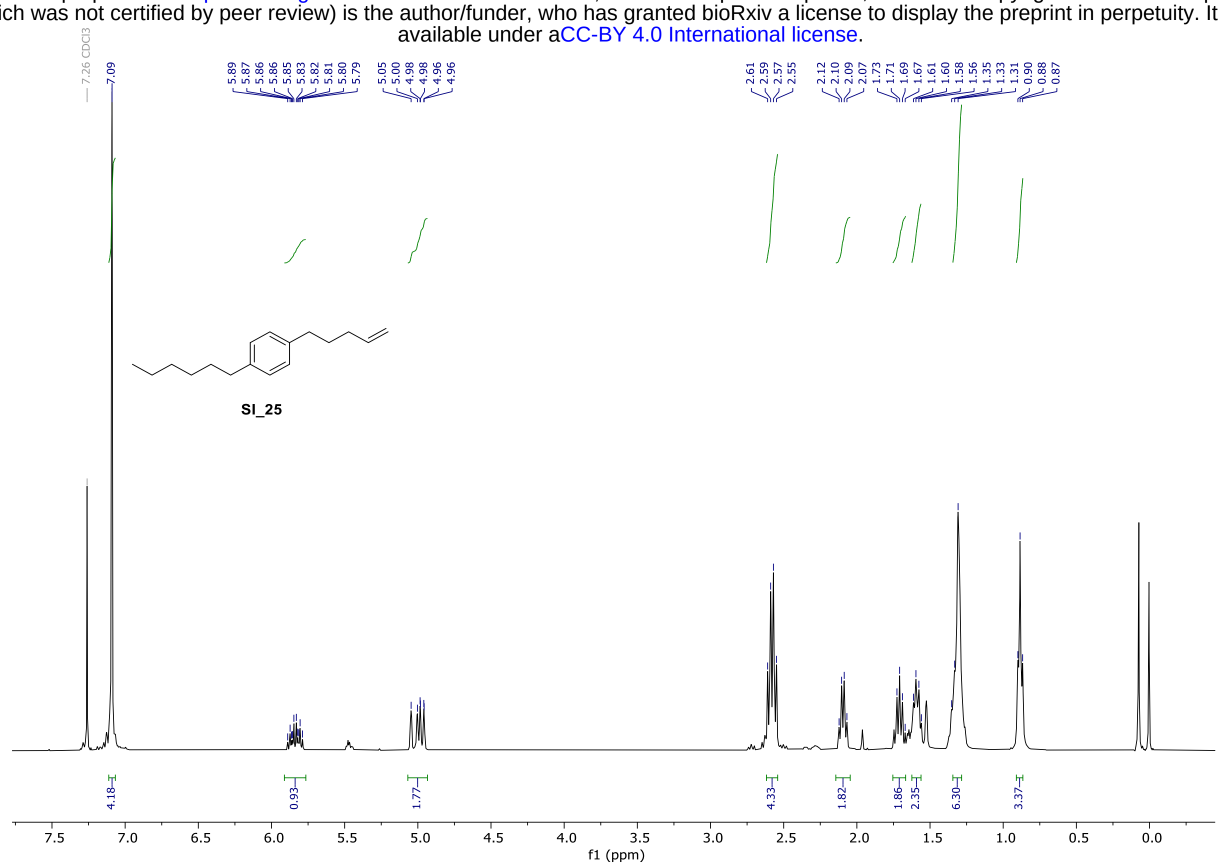

^{1}H NMR (400 MHz, $CDCl_3$) of **SI_25**.

bioRxiv preprint doi: https://doi.org/10.64898/2026.04.24.720406; this version posted April 27, 2026. The copyright holder for this preprint (which was not certified by peer review) is the author/funder, who has granted bioRxiv a license to display the preprint in perpetuity. It is made available under aCC-BY 4.0 International license.

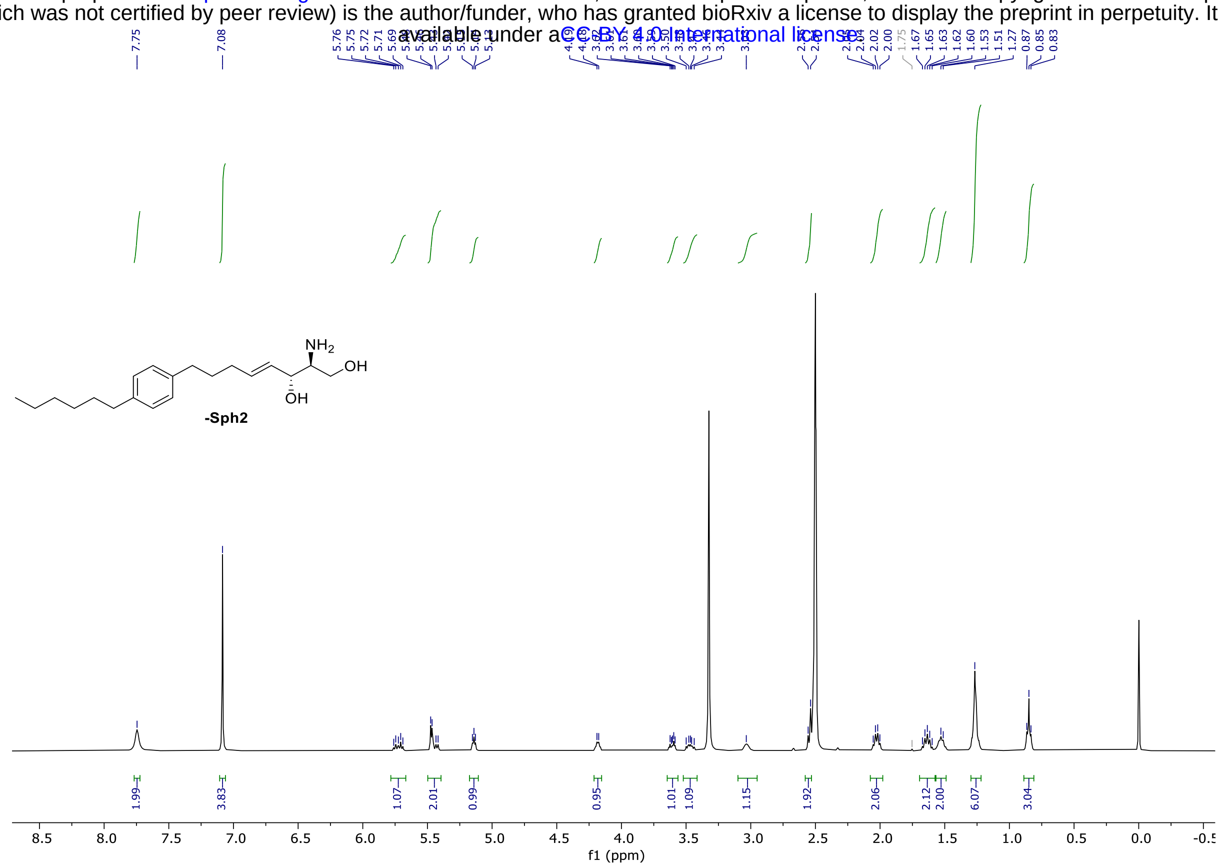

^{1}H NMR (400 MHz, DMSO-d$_6$) of **π-Sph2**.

bioRxiv preprint doi: https://doi.org/10.64898/2026.04.24.720406; this version posted April 27, 2026. The copyright holder for this preprint (which was not certified by peer review) is the author/funder, who has granted bioRxiv a license to display the preprint in perpetuity. It is made available under aCC-BY 4.0 International license.

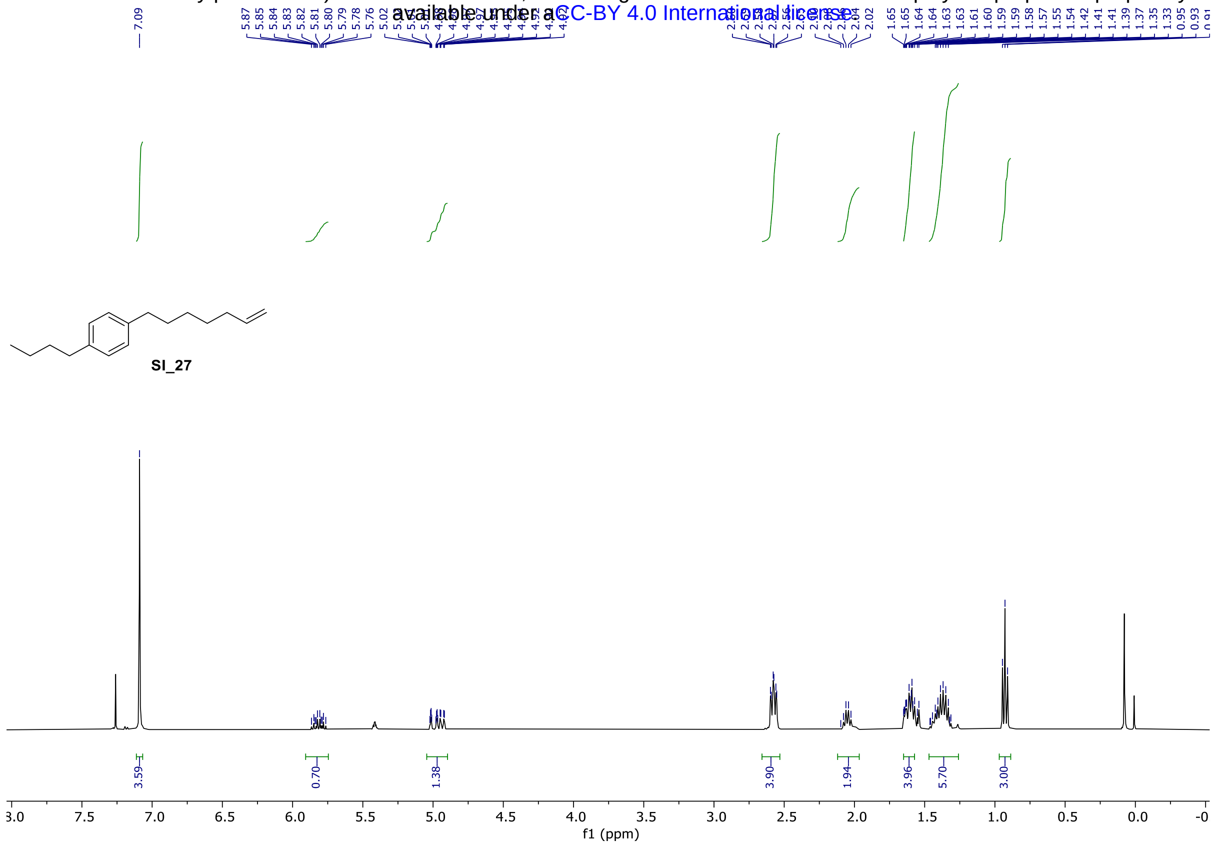

^{1}H NMR (400 MHz, $CDCl_3$) of **SI_27**.

bioRxiv preprint doi: https://doi.org/10.64898/2026.04.24.720406; this version posted April 27, 2026. The copyright holder for this preprint (which was not certified by peer review) is the author/funder, who has granted bioRxiv a license to display the preprint in perpetuity. It is made available under aCC-BY 4.0 International license.

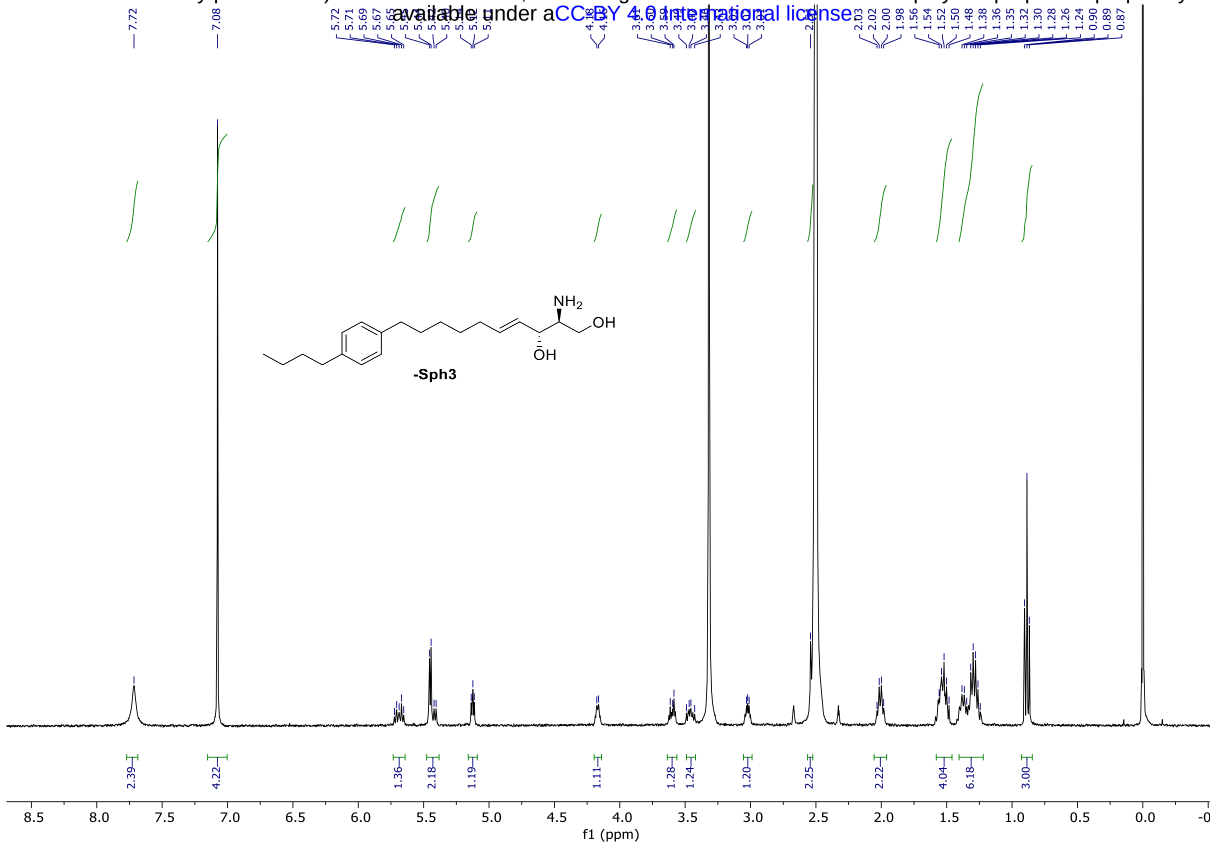

^{1}H NMR (400 MHz, DMSO-d_6) of **π-Sph3**.

bioRxiv preprint doi: https://doi.org/10.64898/2026.04.24.720406; this version posted April 27, 2026. The copyright holder for this preprint (which was not certified by peer review) is the author/funder, who has granted bioRxiv a license to display the preprint in perpetuity. It is made available under aCC-BY 4.0 International license.

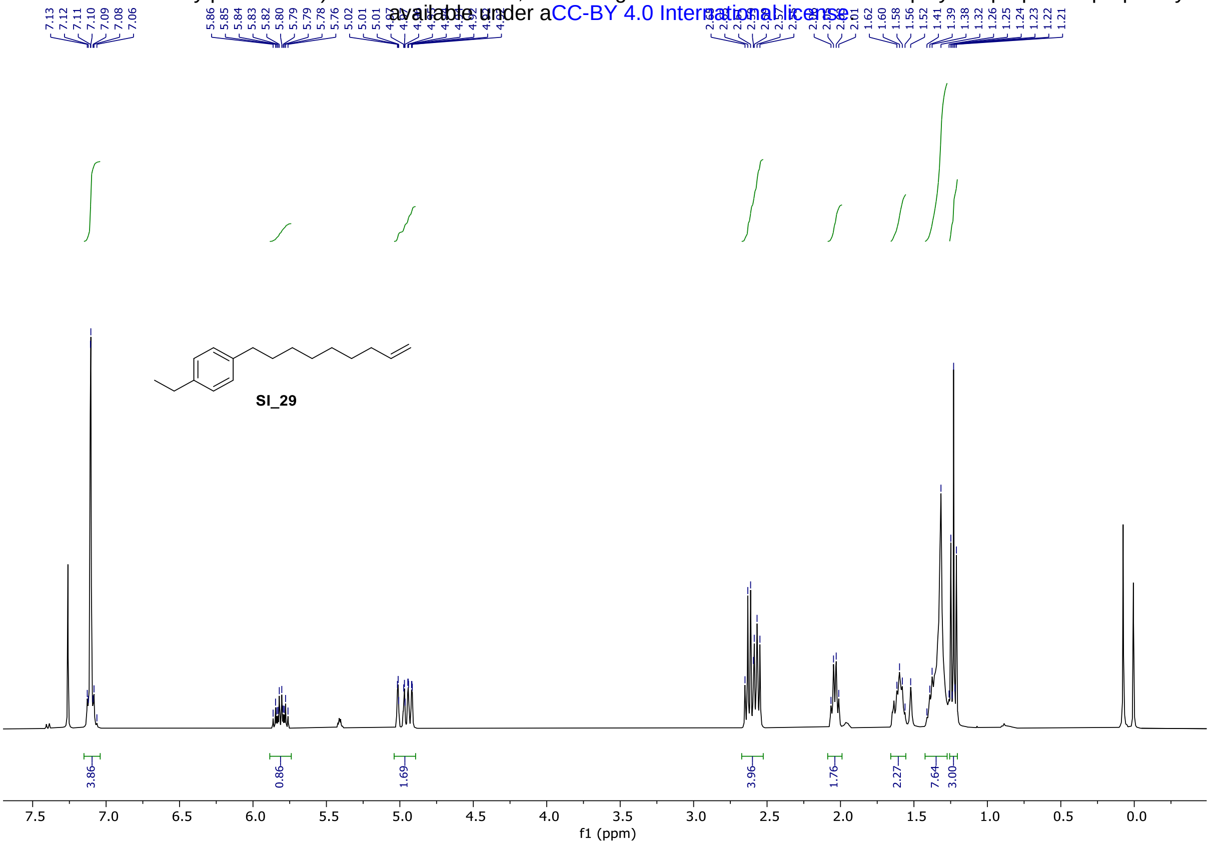

^{1}H NMR (400 MHz, $CDCl_3$) of **SI_29**.

bioRxiv preprint doi: https://doi.org/10.64898/2026.04.24.720406; this version posted April 27, 2026. The copyright holder for this preprint (which was not certified by peer review) is the author/funder, who has granted bioRxiv a license to display the preprint in perpetuity. It is made available under aCC-BY 4.0 International license.

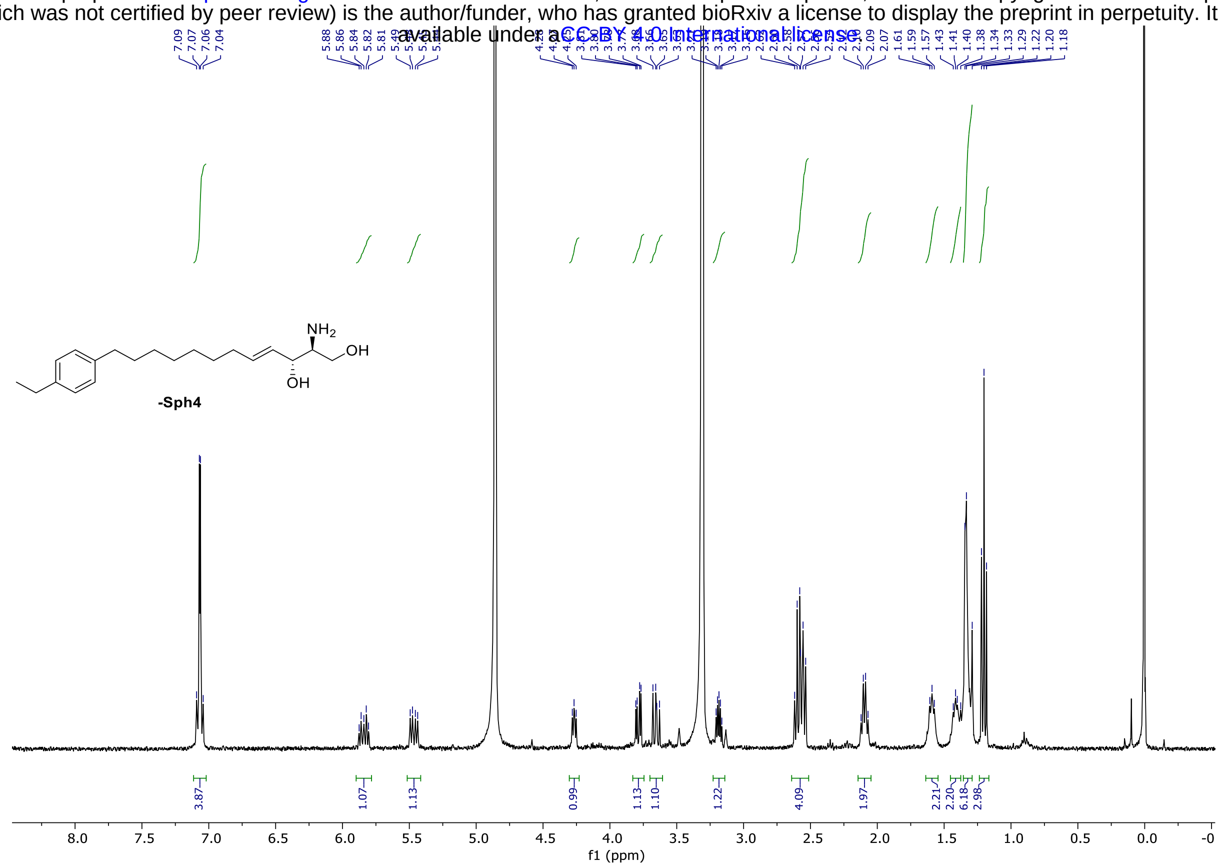

^{1}H NMR (400 MHz, MeOH-d$_4$) of **π-Sph4**.

bioRxiv preprint doi: https://doi.org/10.64898/2026.04.24.720406; this version posted April 27, 2026. The copyright holder for this preprint (which was not certified by peer review) is the author/funder, who has granted bioRxiv a license to display the preprint in perpetuity. It is made available under aCC-BY 4.0 International license.

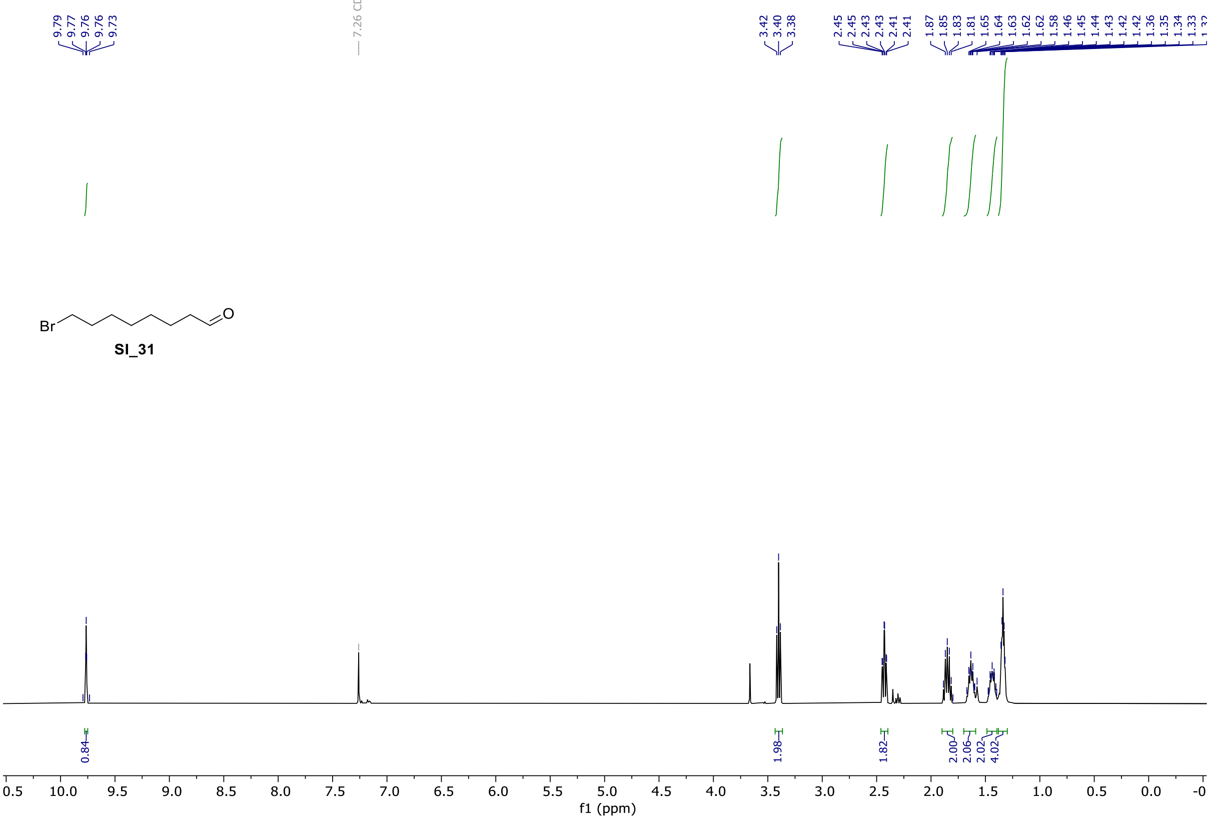

^{1}H NMR (400 MHz, $CDCl_3$) of **SI_31.**

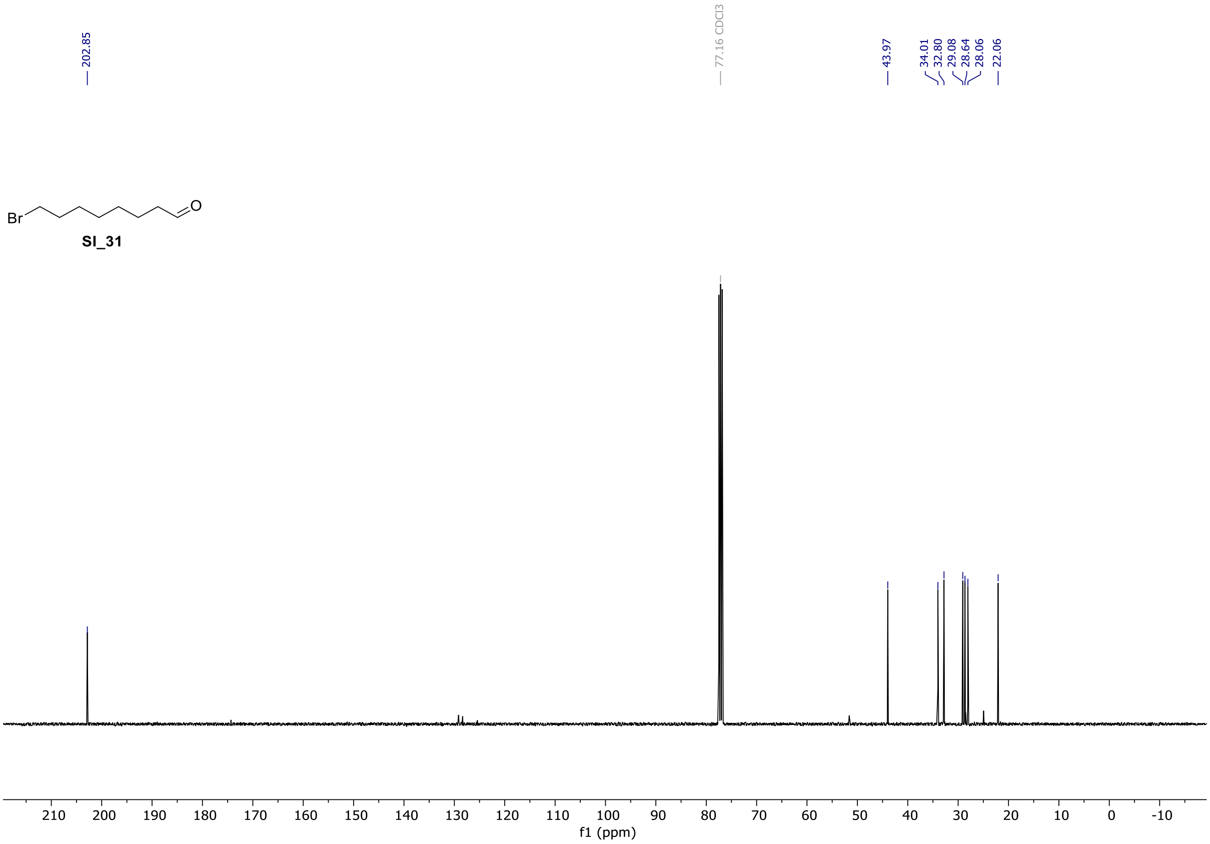

^{13}C NMR (101 MHz, $CDCl_3$) of **SI_31**.

bioRxiv preprint doi: https://doi.org/10.64898/2026.04.24.720406; this version posted April 27, 2026. The copyright holder for this preprint (which was not certified by peer review) is the author/funder, who has granted bioRxiv a license to display the preprint in perpetuity. It is made available under aCC-BY 4.0 International license.

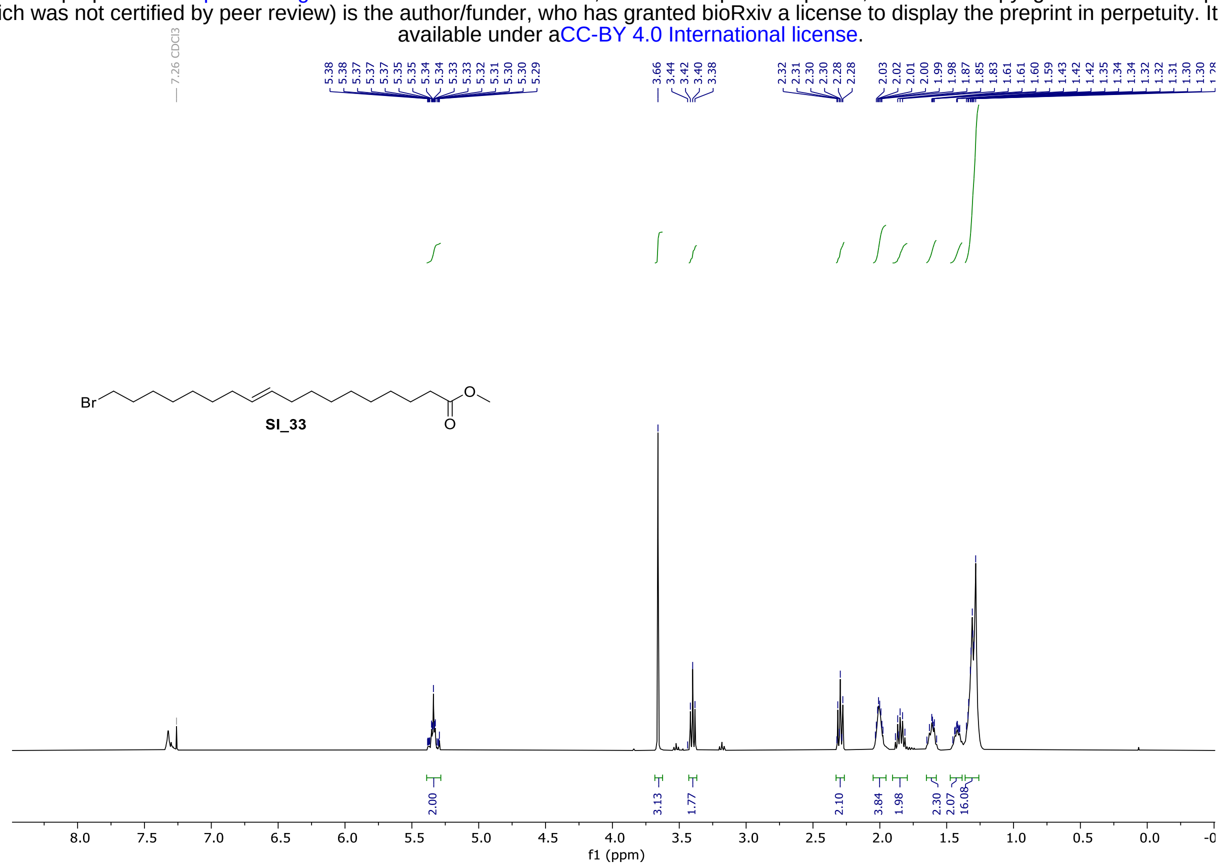

^{1}H NMR (400 MHz, $CDCl_3$) of **SI_33**.

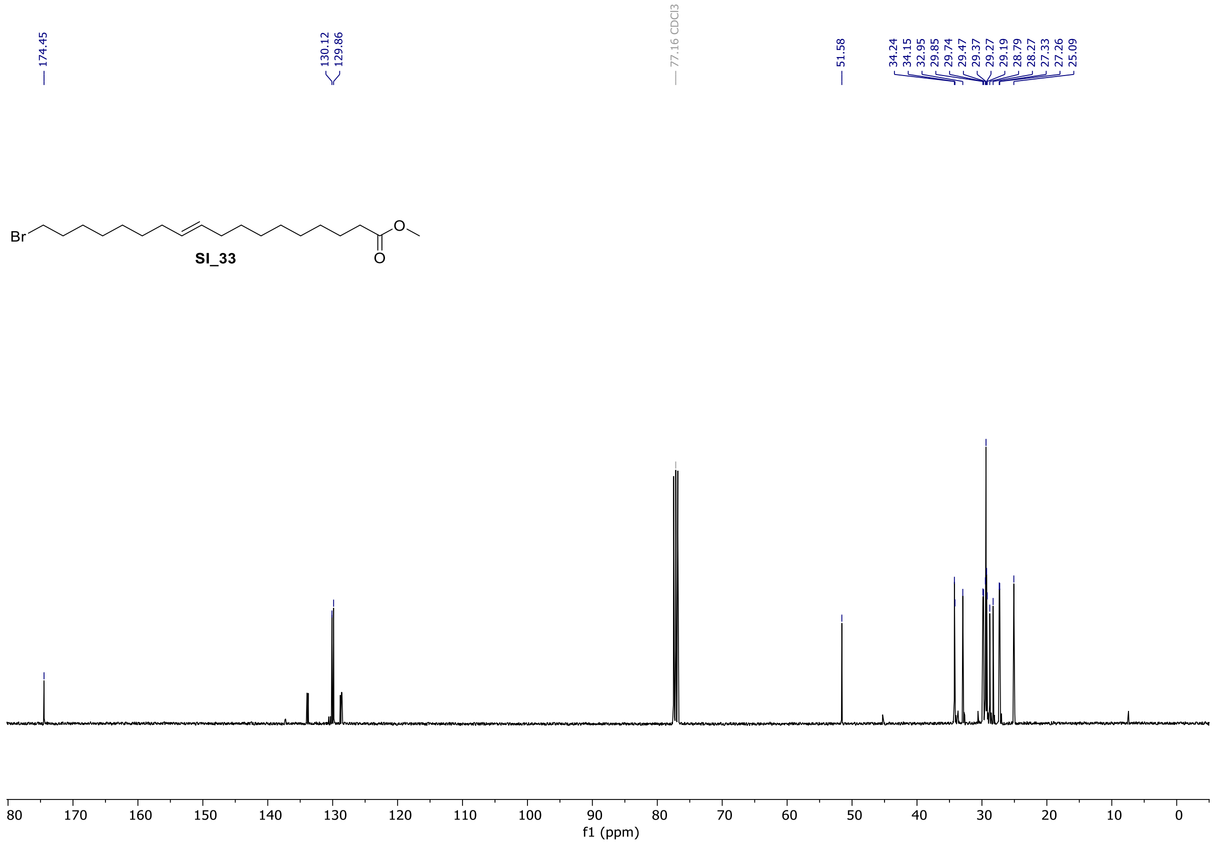

^{13}C NMR (101 MHz, $CDCl_3$) of **SI_33**.

bioRxiv preprint doi: https://doi.org/10.64898/2026.04.24.720406; this version posted April 27, 2026. The copyright holder for this preprint (which was not certified by peer review) is the author/funder, who has granted bioRxiv a license to display the preprint in perpetuity. It is made available under aCC-BY 4.0 International license.

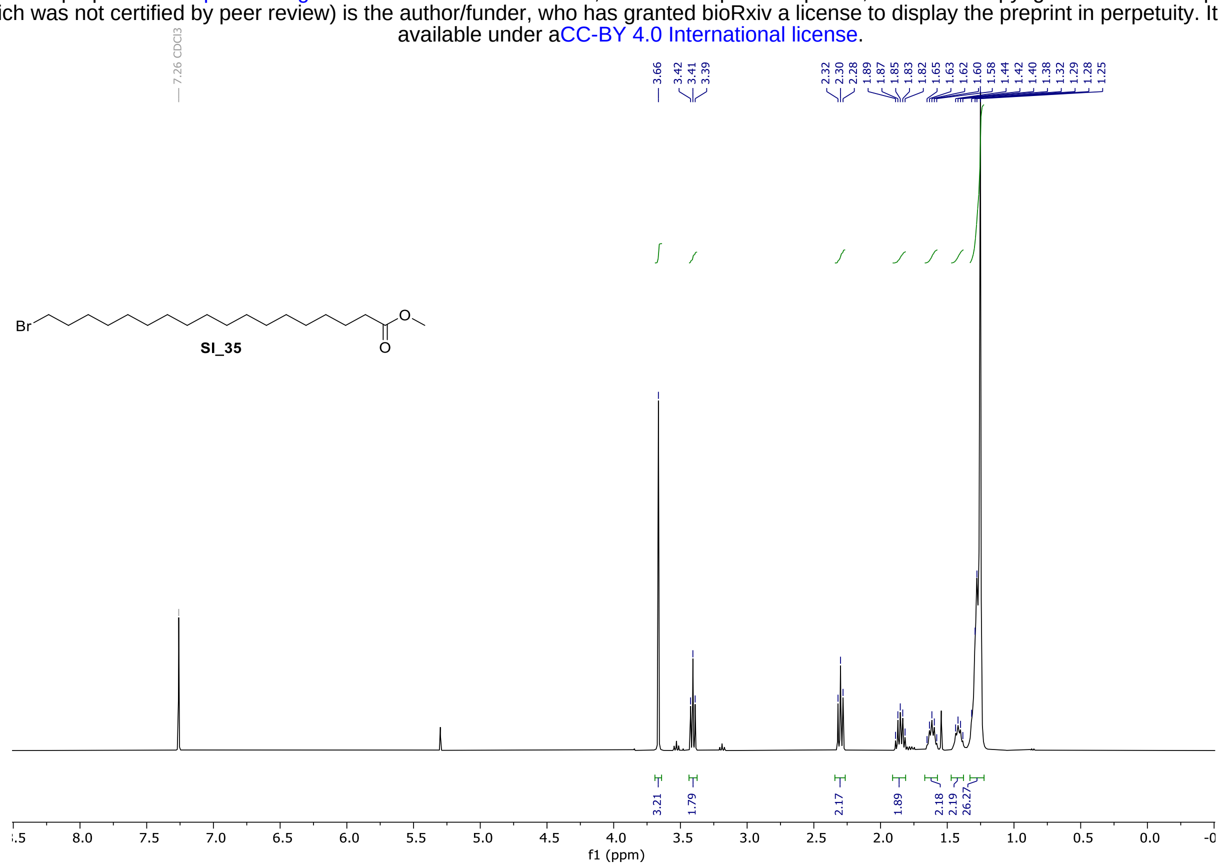

^{1}**H NMR** (400 MHz, $CDCl_3$) of **SI_35**.

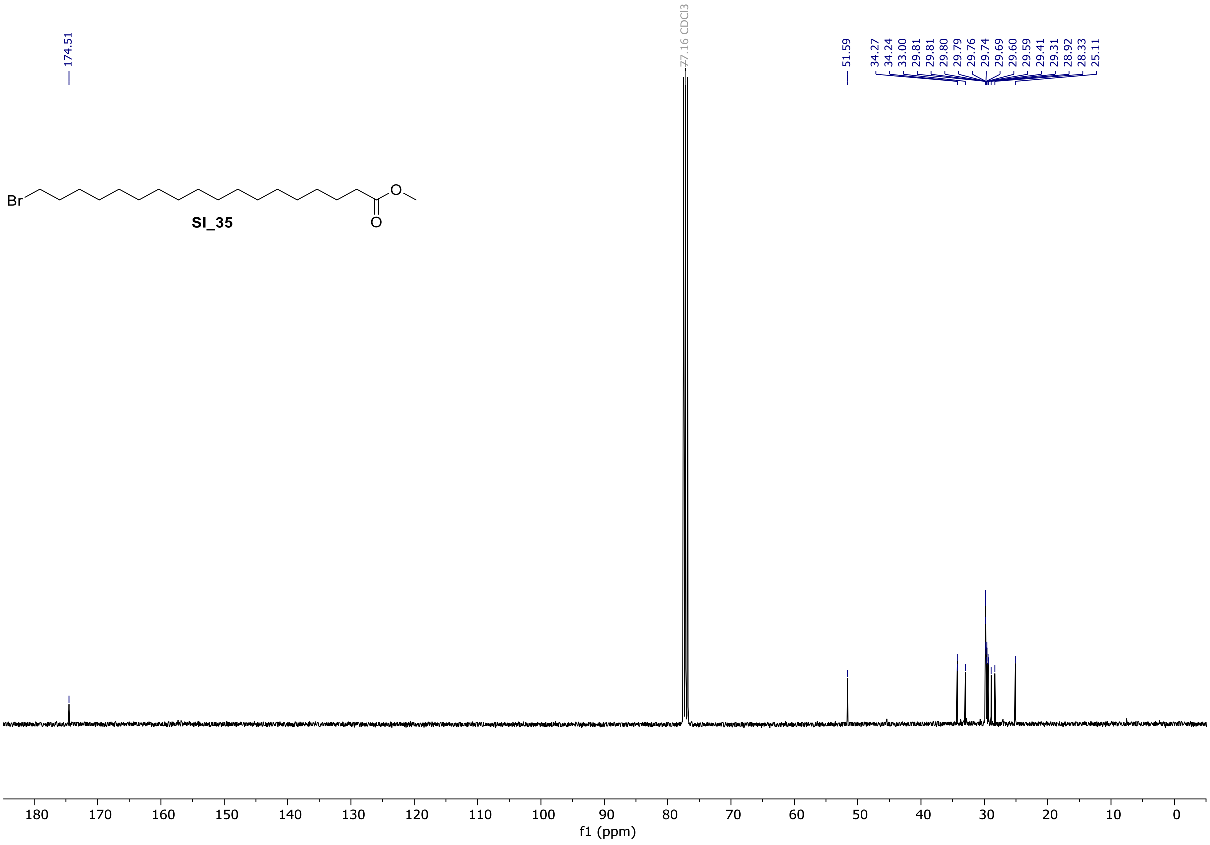

^{13}**C NMR** (101 MHz, $CDCl_3$) of **SI_35**.

bioRxiv preprint doi: https://doi.org/10.64898/2026.04.24.720406; this version posted April 27, 2026. The copyright holder for this preprint (which was not certified by peer review) is the author/funder, who has granted bioRxiv a license to display the preprint in perpetuity. It is made available under aCC-BY 4.0 International license.

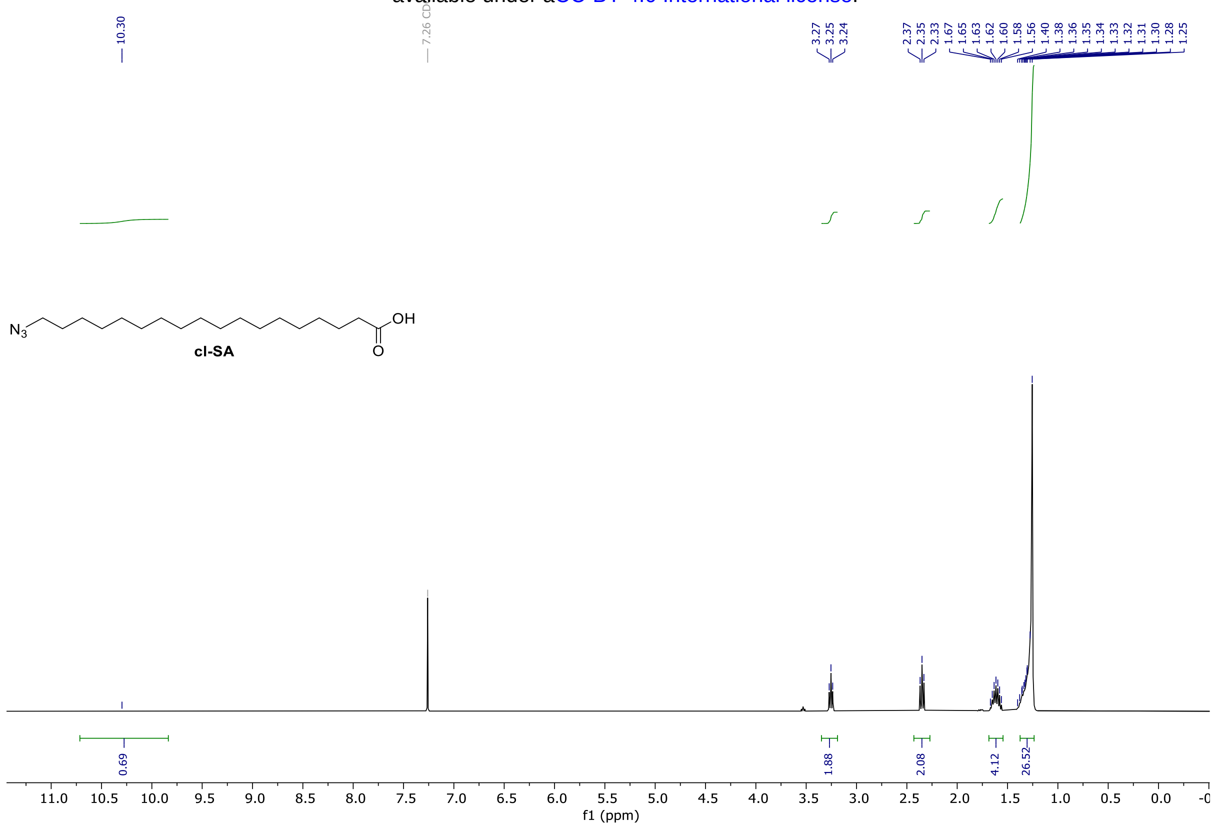

^{1}H NMR (400 MHz, $CDCl_3$) of **cl-SA**.

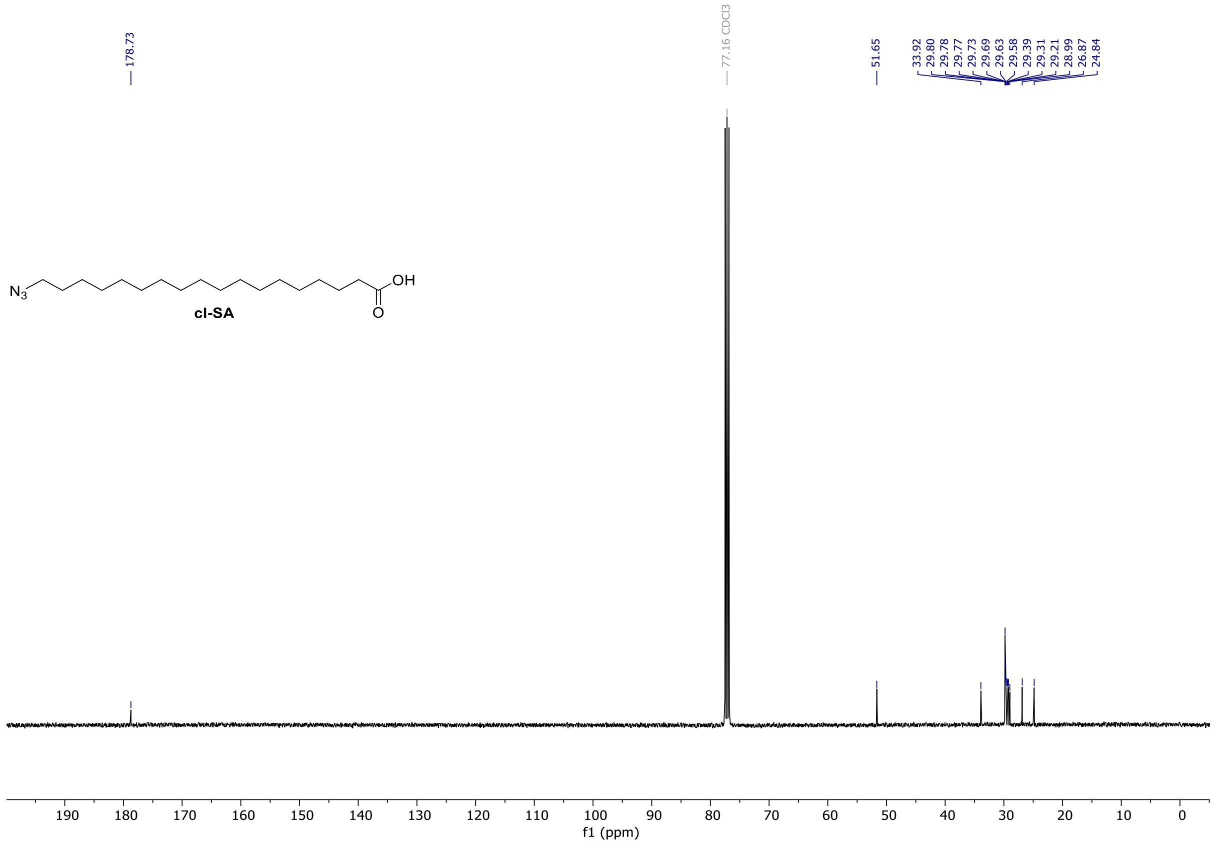

^{13}C NMR (101 MHz, $CDCl_3$) of **cl-SA**.

bioRxiv preprint doi: https://doi.org/10.64898/2026.04.24.720406; this version posted April 27, 2026. The copyright holder for this preprint (which was not certified by peer review) is the author/funder, who has granted bioRxiv a license to display the preprint in perpetuity. It is made available under aCC-BY 4.0 International license.

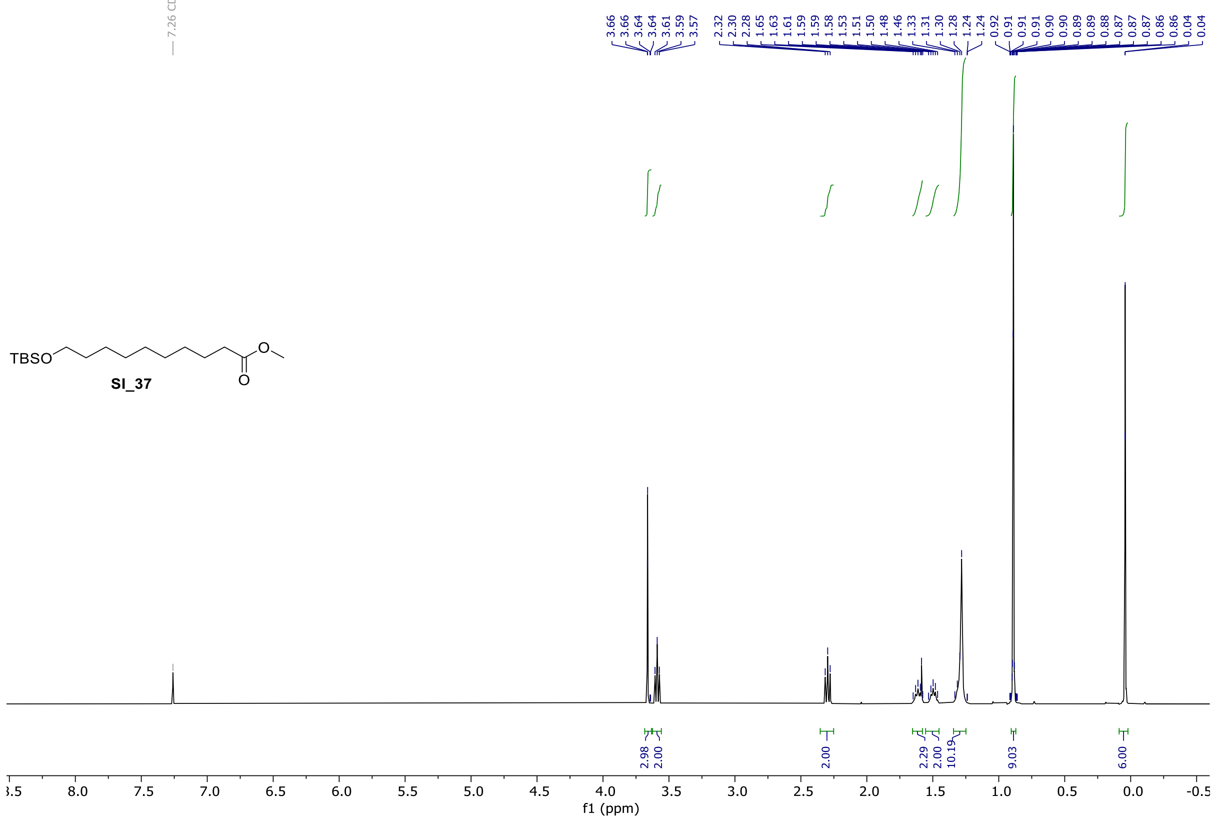

^{1}H NMR (400 MHz, $CDCl_3$) of **SI_37**.

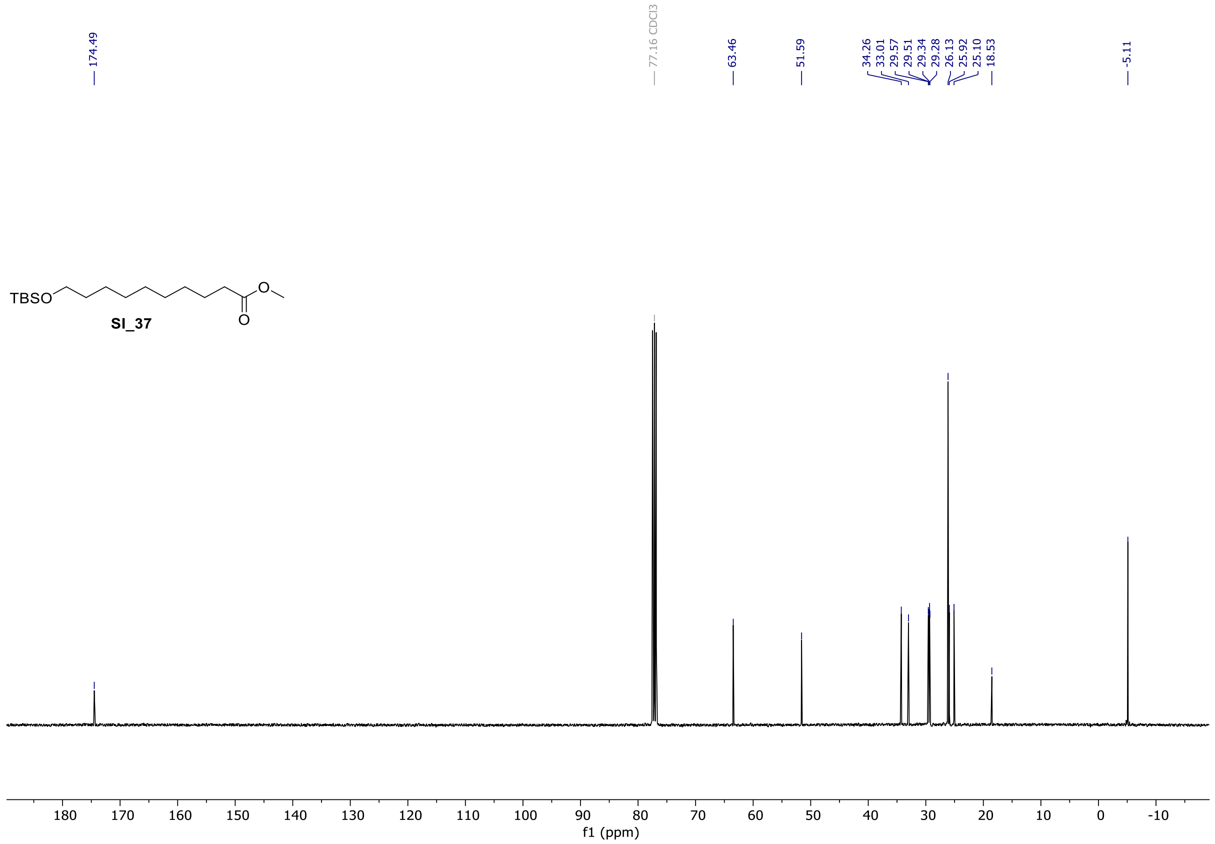

^{13}C NMR (101 MHz, $CDCl_3$) of **SI_37**.

bioRxiv preprint doi: https://doi.org/10.64898/2026.04.24.720406; this version posted April 27, 2026. The copyright holder for this preprint (which was not certified by peer review) is the author/funder, who has granted bioRxiv a license to display the preprint in perpetuity. It is made available under aCC-BY 4.0 International license.

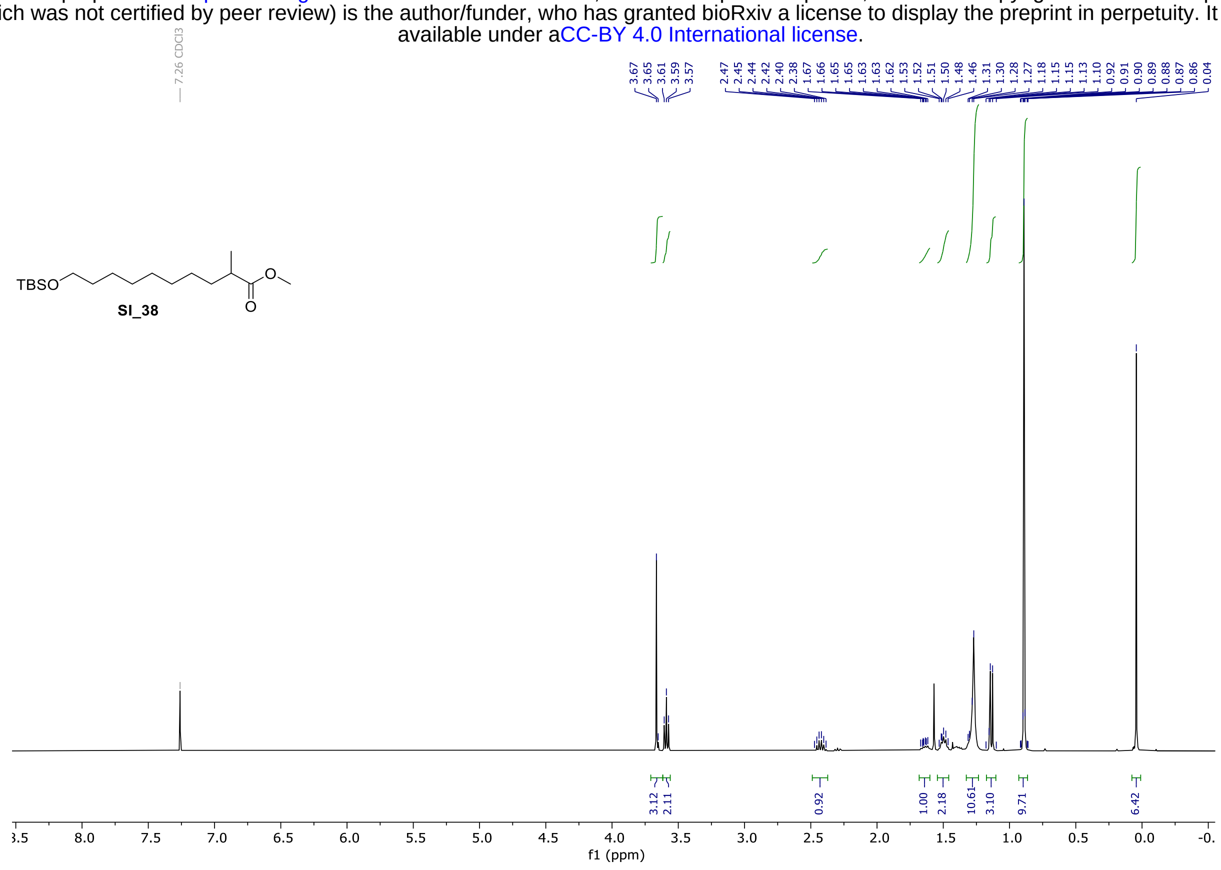

^{1}H NMR (400 MHz, $CDCl_3$) of **SI_38**.

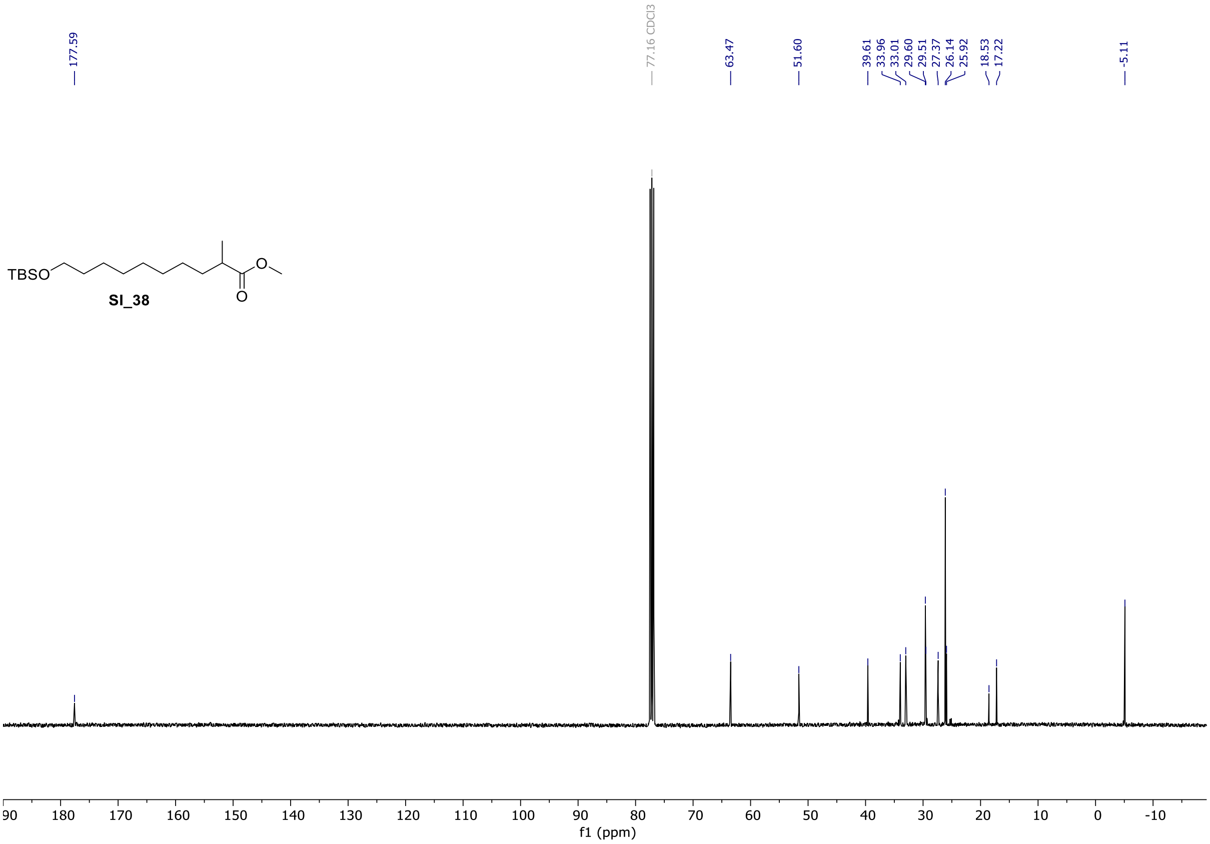

^{13}C NMR (101 MHz, $CDCl_3$) of **SI_38**.

bioRxiv preprint doi: https://doi.org/10.64898/2026.04.24.720406; this version posted April 27, 2026. The copyright holder for this preprint (which was not certified by peer review) is the author/funder, who has granted bioRxiv a license to display the preprint in perpetuity. It is made available under aCC-BY 4.0 International license.

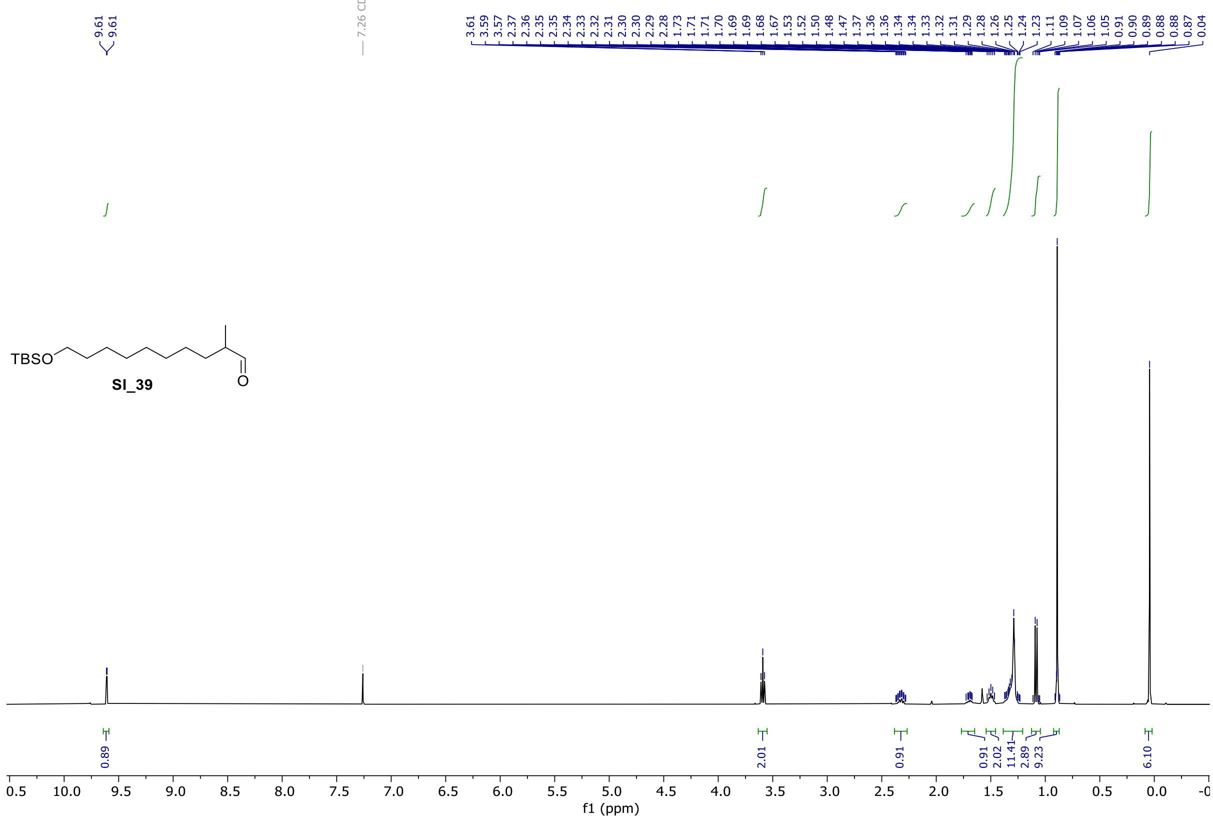

^{1}H NMR (400 MHz, $CDCl_3$) of **SI_39**.

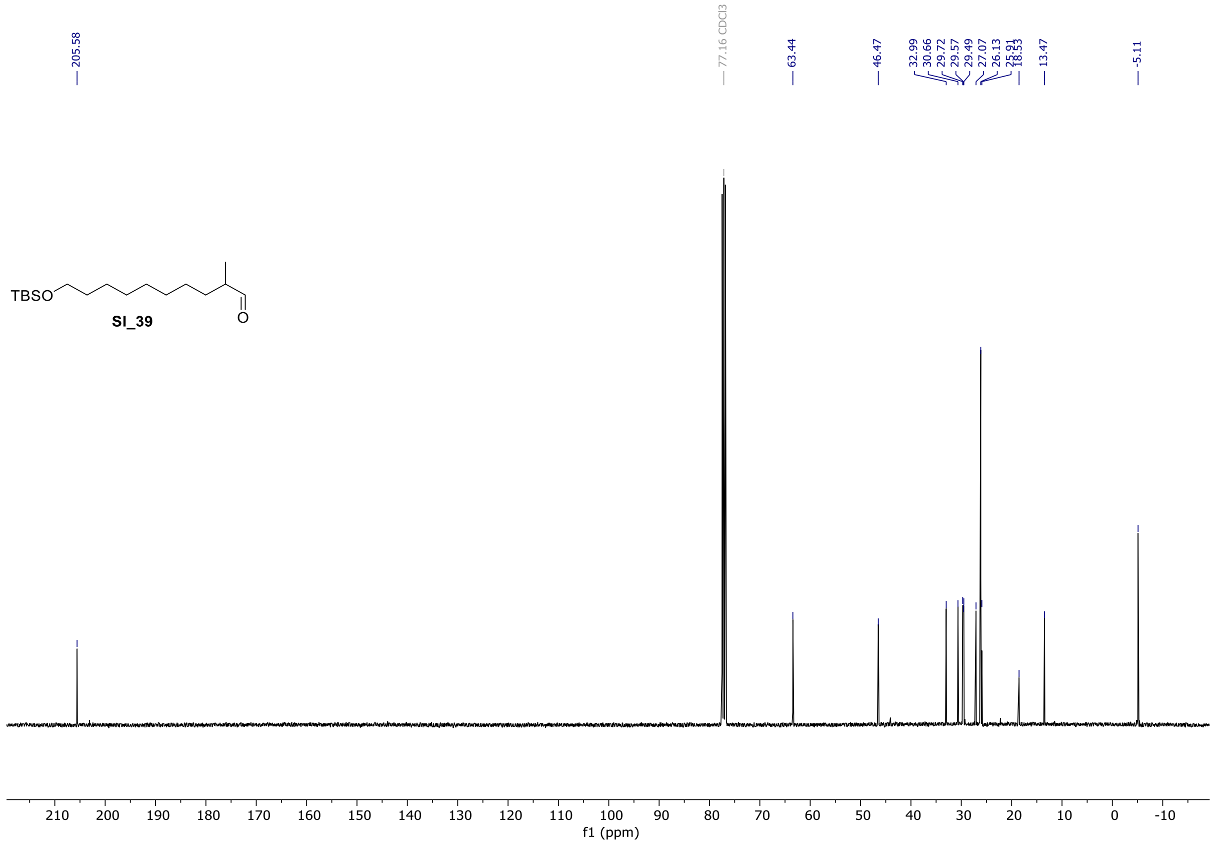

^{13}C NMR (101 MHz, $CDCl_3$) of **SI_39**.

bioRxiv preprint doi: https://doi.org/10.64898/2026.04.24.720406; this version posted April 27, 2026. The copyright holder for this preprint (which was not certified by peer review) is the author/funder, who has granted bioRxiv a license to display the preprint in perpetuity. It is made available under aCC-BY 4.0 International license.

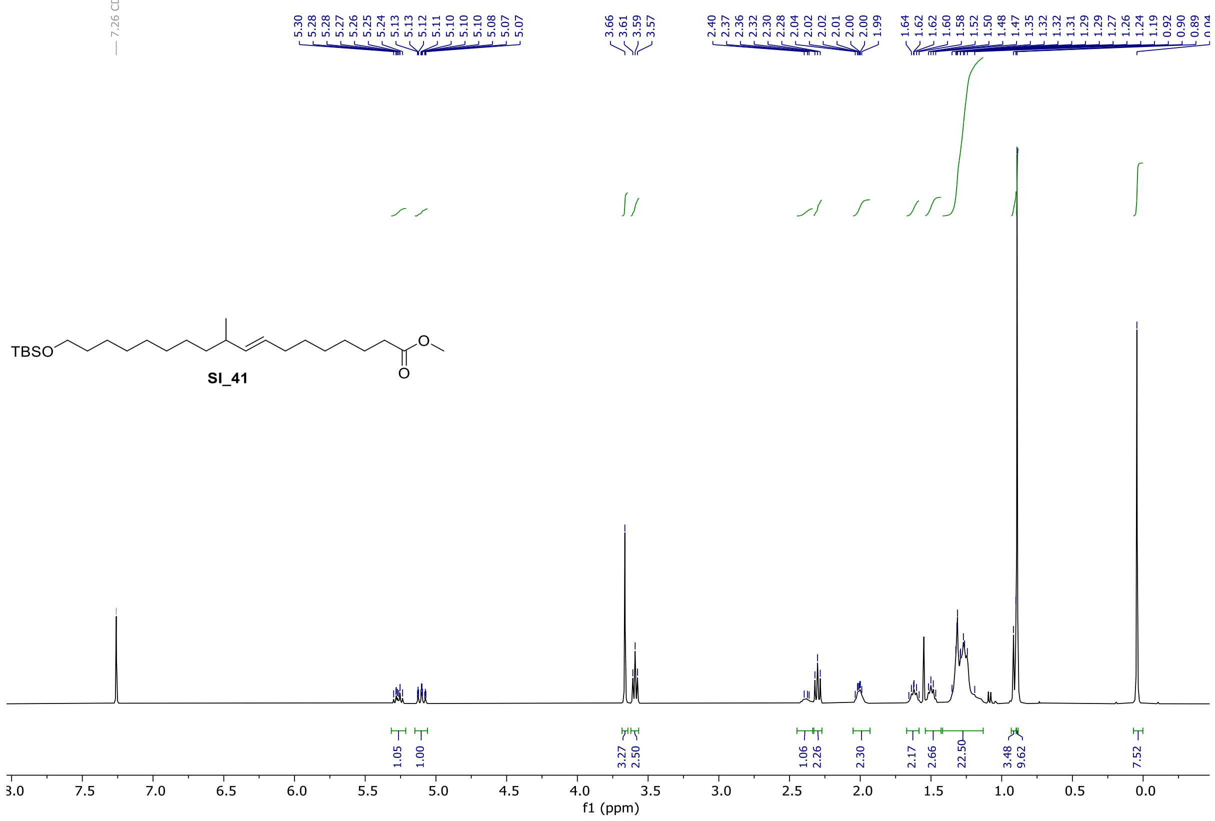

^{1}H NMR (400 MHz, $CDCl_3$) of **SI_41**.

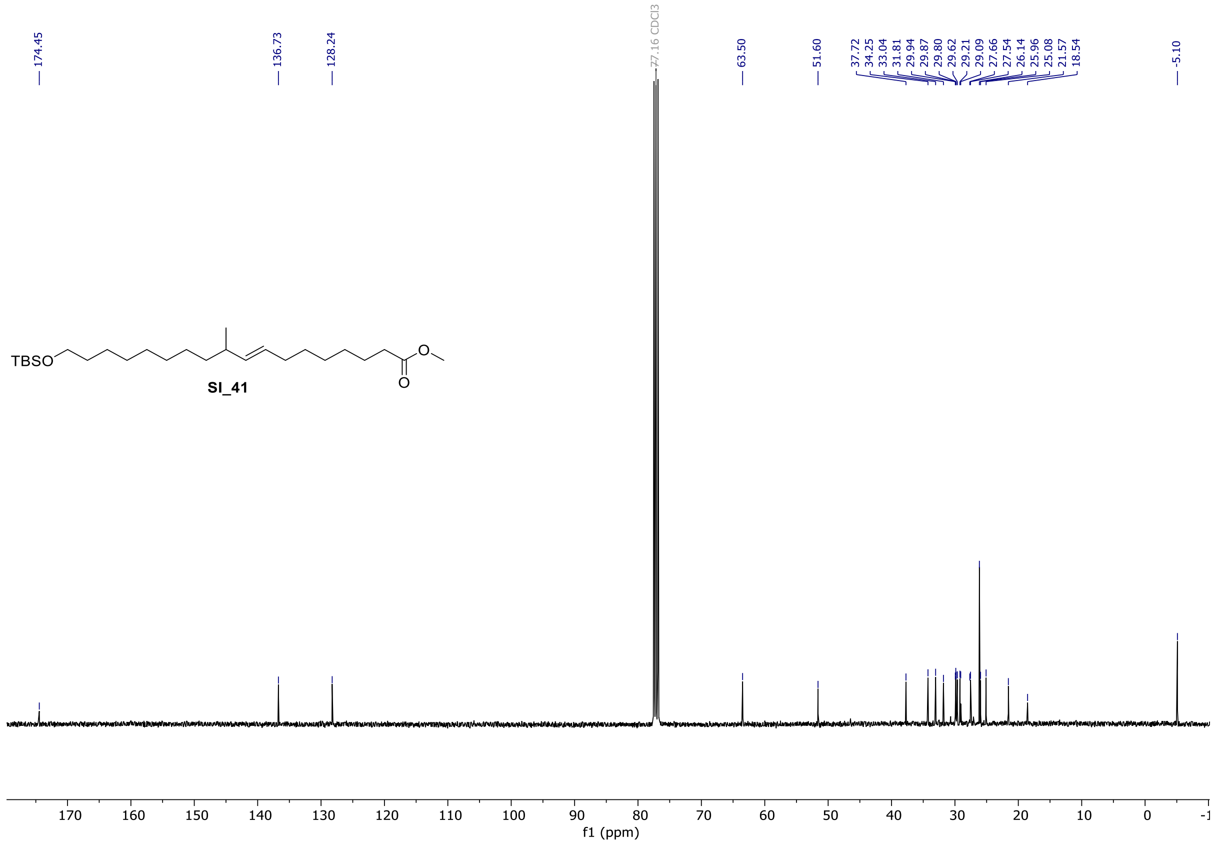

^{13}C NMR (101 MHz, $CDCl_3$) of **SI_41**.

bioRxiv preprint doi: https://doi.org/10.64898/2026.04.24.720406; this version posted April 27, 2026. The copyright holder for this preprint (which was not certified by peer review) is the author/funder, who has granted bioRxiv a license to display the preprint in perpetuity. It is made available under aCC-BY 4.0 International license.

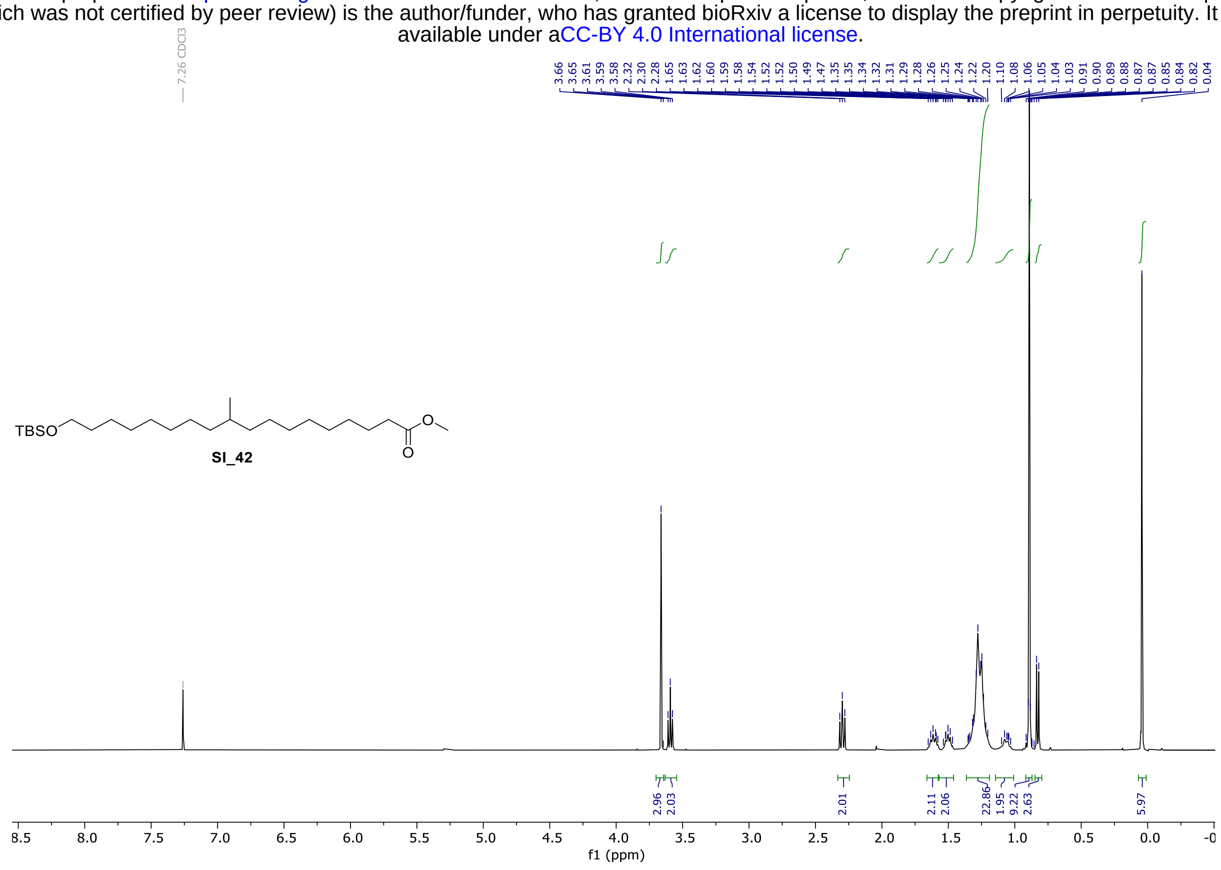

^{1}**H NMR** (400 MHz, $CDCl_3$) of **SI_42**.

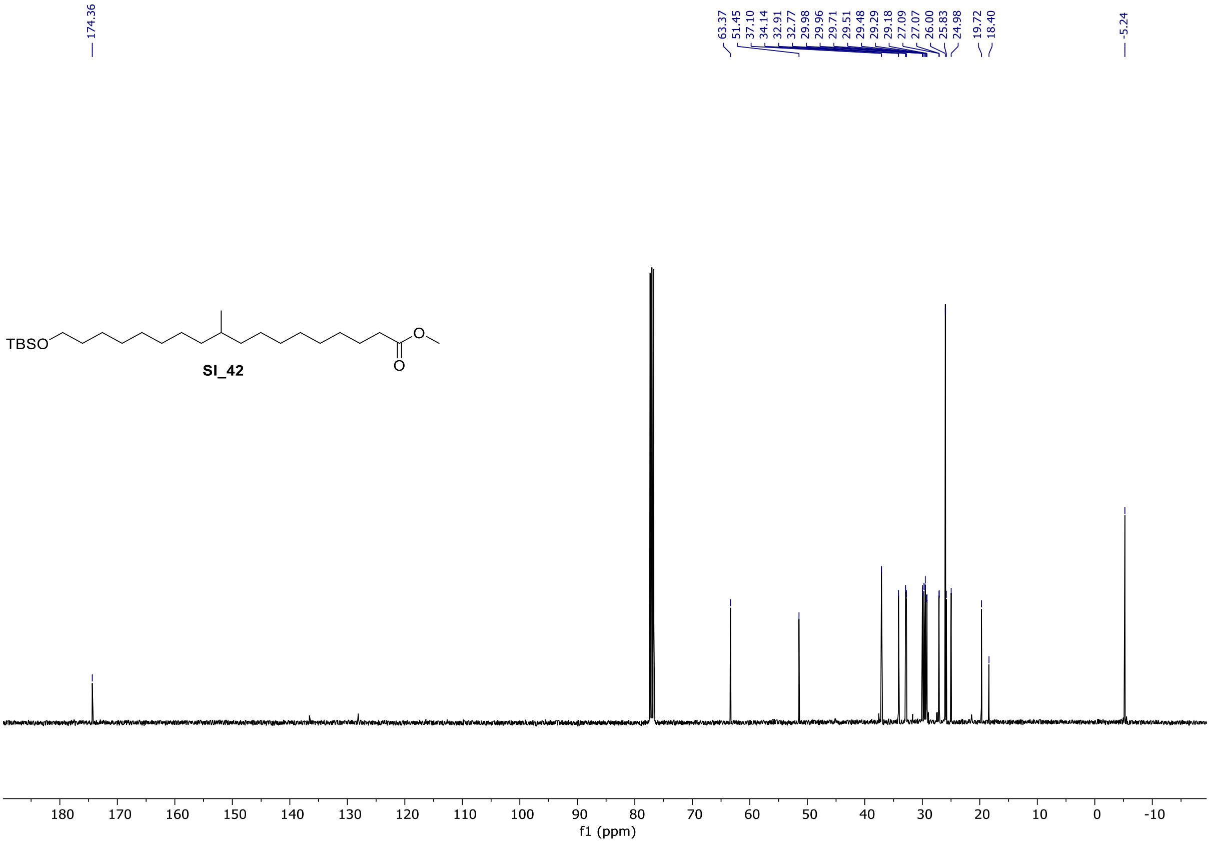

^{13}**C NMR** (101 MHz, $CDCl_3$) of **SI_42**.

bioRxiv preprint doi: https://doi.org/10.64898/2026.04.24.720406; this version posted April 27, 2026. The copyright holder for this preprint (which was not certified by peer review) is the author/funder, who has granted bioRxiv a license to display the preprint in perpetuity. It is made available under aCC-BY 4.0 International license.

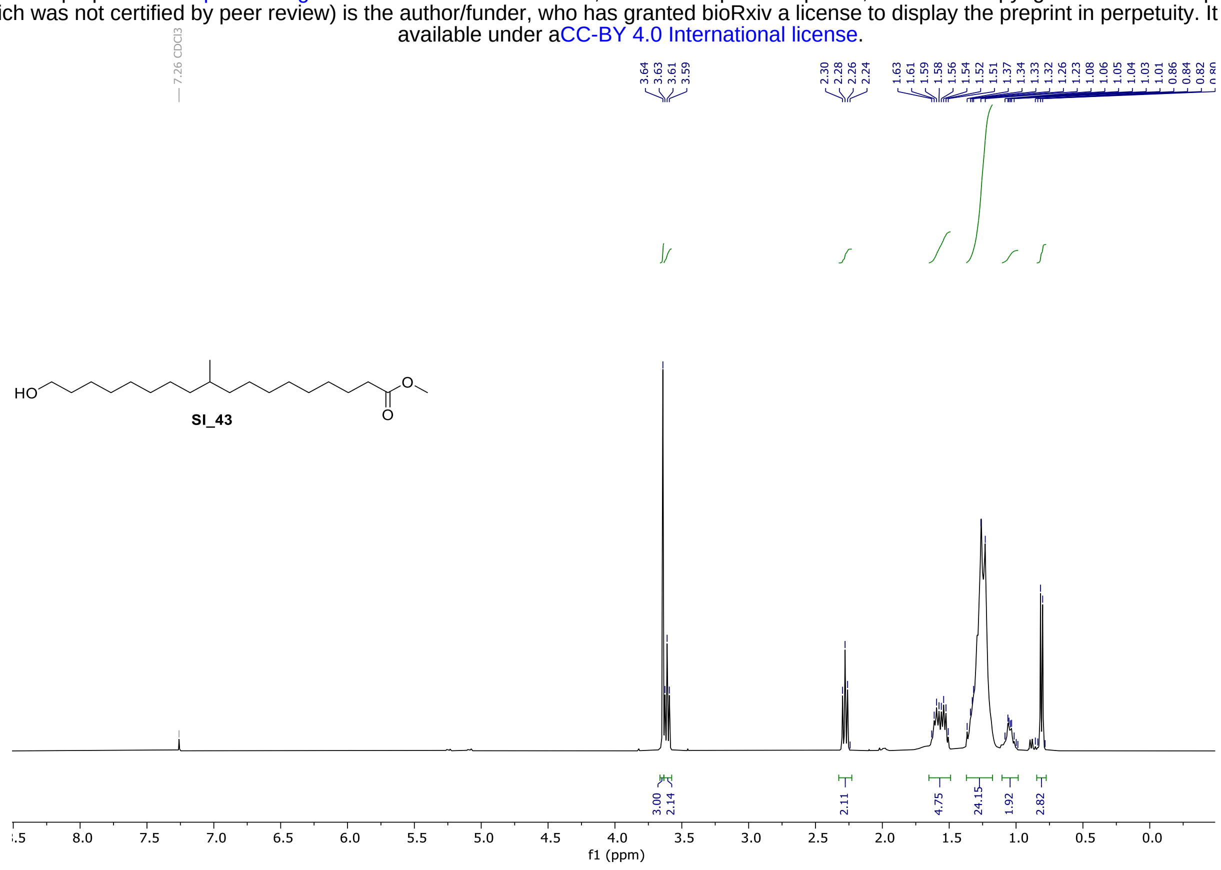

^{1}H NMR (400 MHz, $CDCl_3$) of **SI_43**.

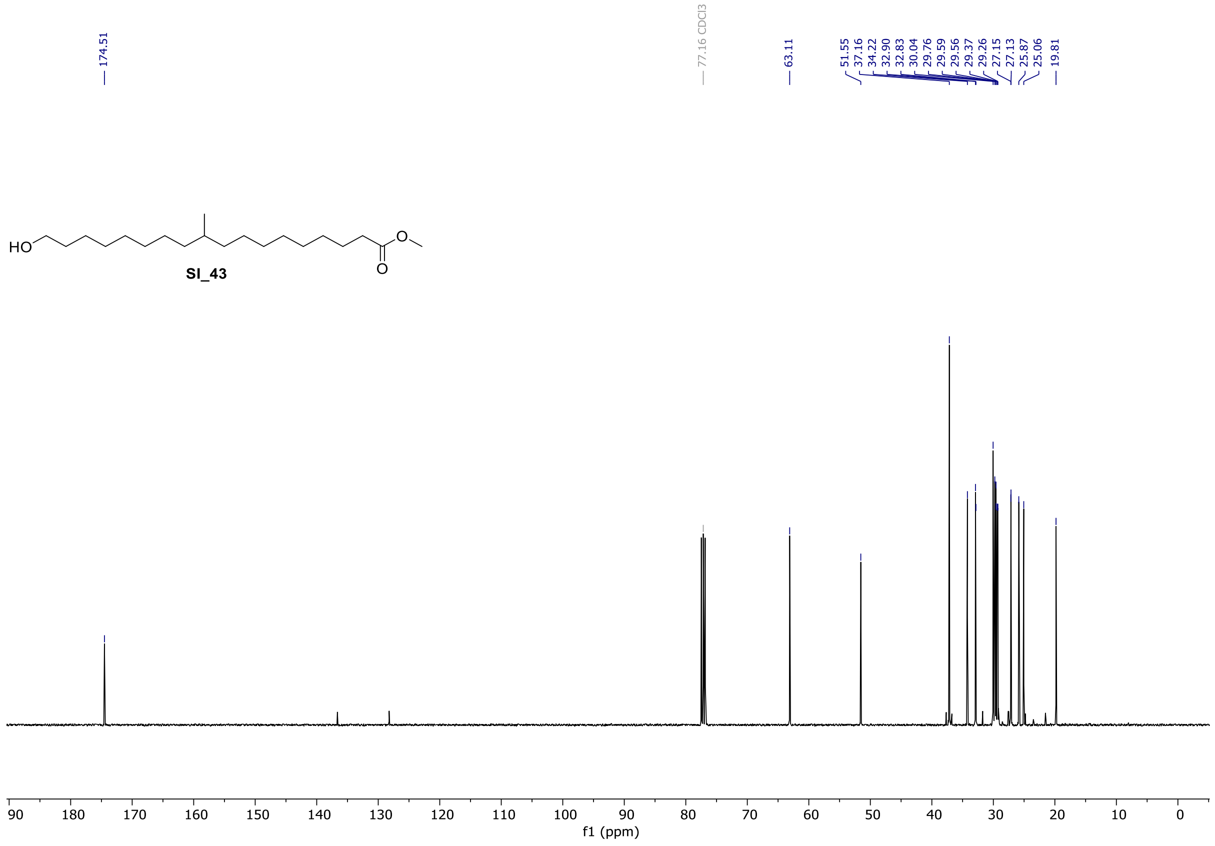

^{13}C NMR (101 MHz, $CDCl_3$) of **SI_43**.

bioRxiv preprint doi: https://doi.org/10.64898/2026.04.24.720406; this version posted April 27, 2026. The copyright holder for this preprint (which was not certified by peer review) is the author/funder, who has granted bioRxiv a license to display the preprint in perpetuity. It is made available under aCC-BY 4.0 International license.

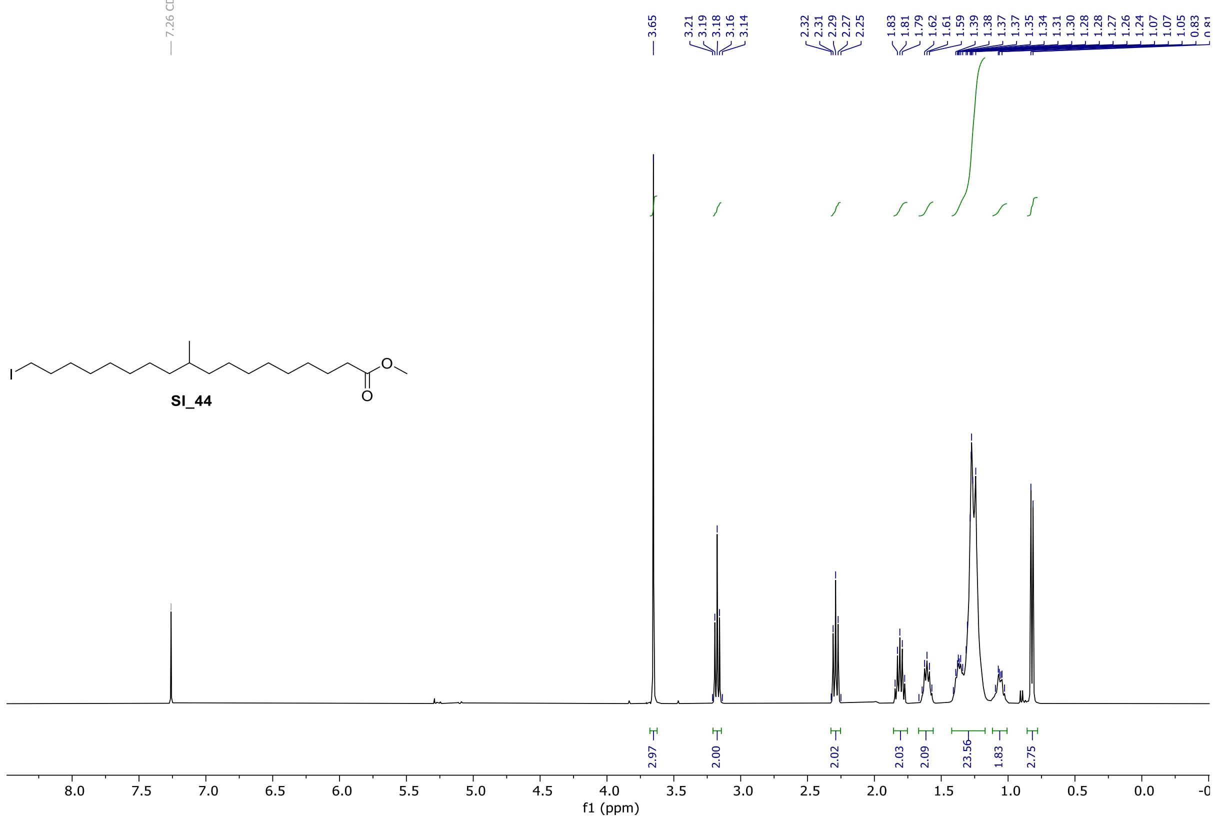

^{1}H NMR (400 MHz, $CDCl_3$) of **SI_44**.

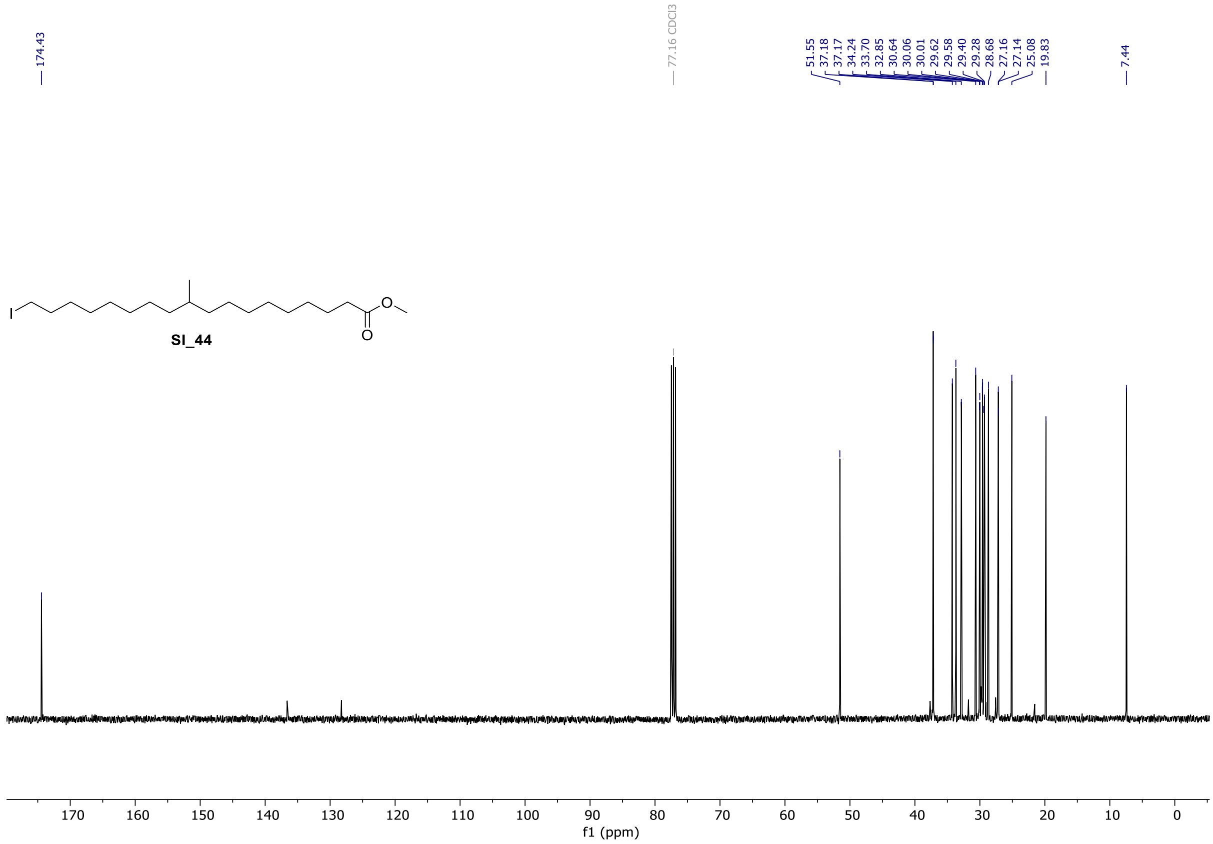

^{13}C NMR (101 MHz, $CDCl_3$) of **SI_44**.

bioRxiv preprint doi: https://doi.org/10.64898/2026.04.24.720406; this version posted April 27, 2026. The copyright holder for this preprint (which was not certified by peer review) is the author/funder, who has granted bioRxiv a license to display the preprint in perpetuity. It is made available under aCC-BY 4.0 International license.

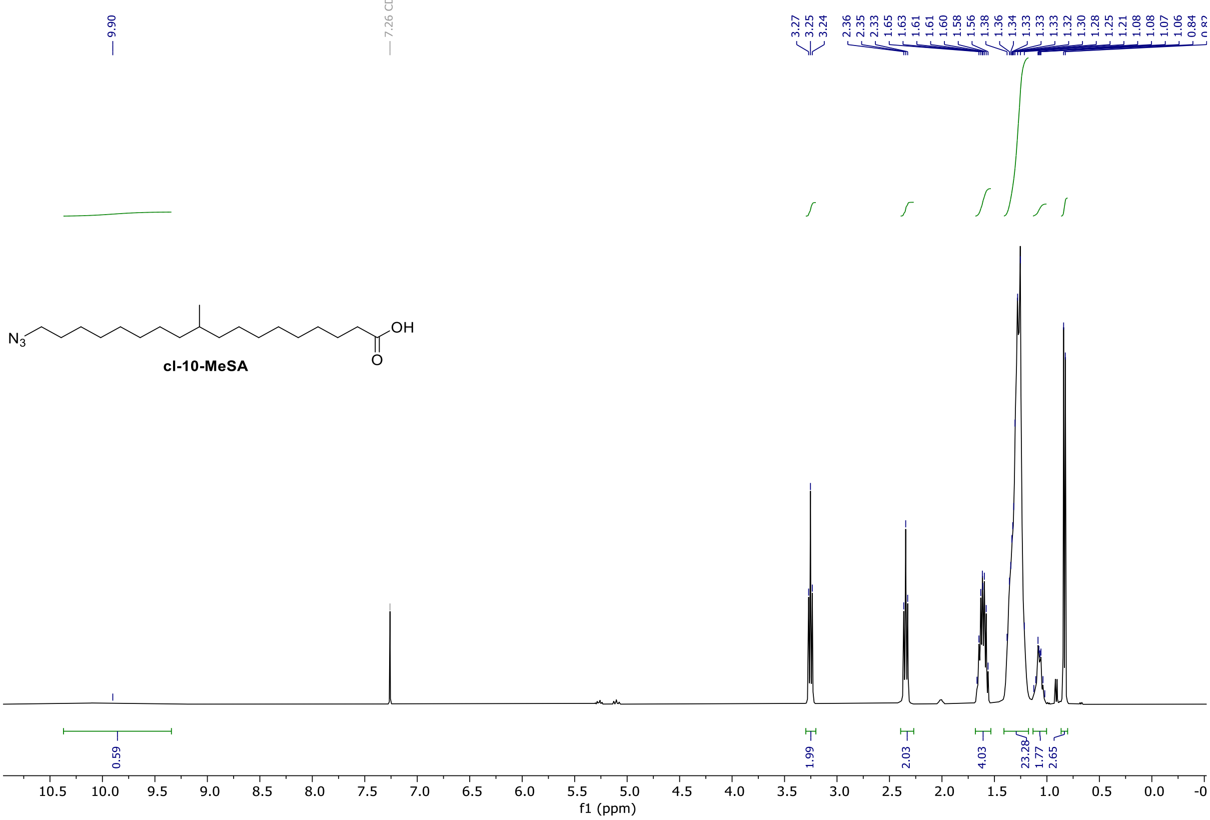

^{1}H NMR (400 MHz, $CDCl_3$) of **cl-10-MeSA**.

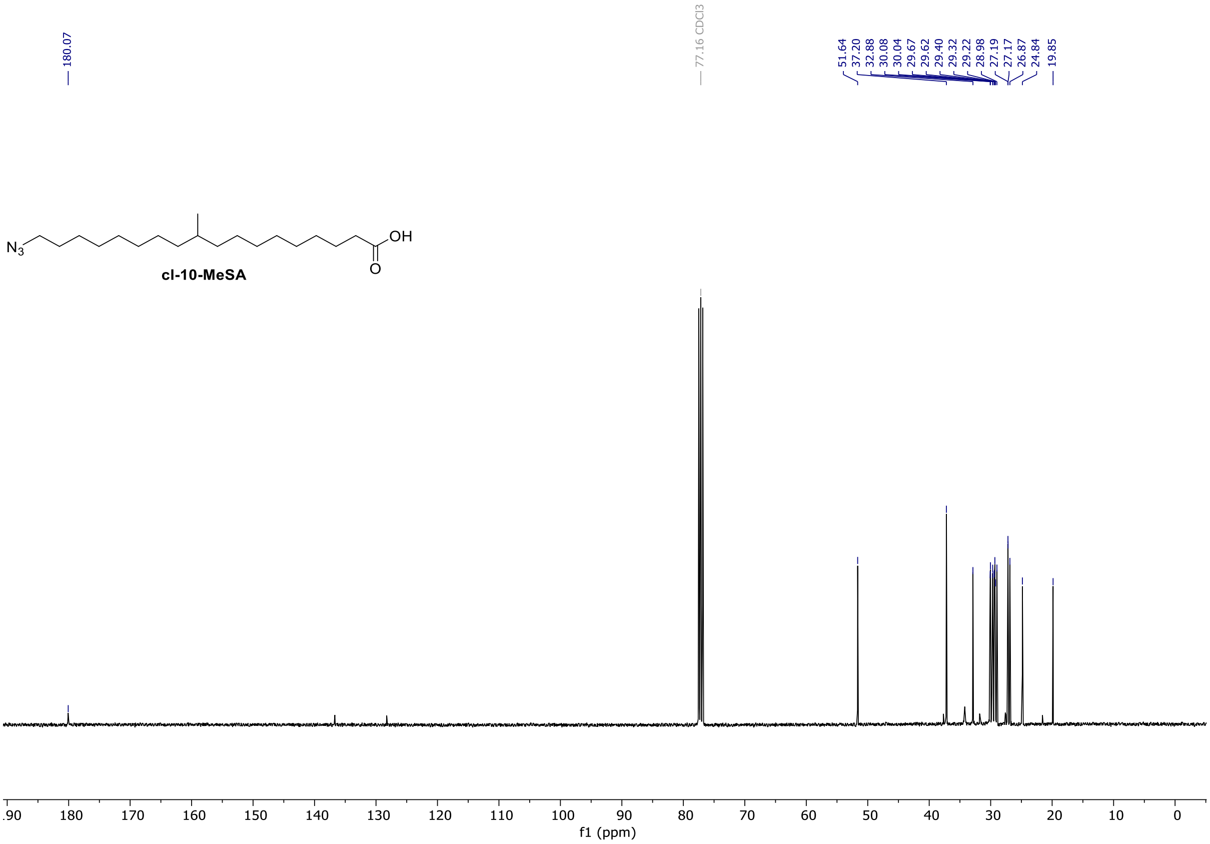

^{13}C NMR (101 MHz, $CDCl_3$) of **cl-10-MeSA**.

bioRxiv preprint doi: https://doi.org/10.64898/2026.04.24.720406; this version posted April 27, 2026. The copyright holder for this preprint (which was not certified by peer review) is the author/funder, who has granted bioRxiv a license to display the preprint in perpetuity. It is made available under aCC-BY 4.0 International license.

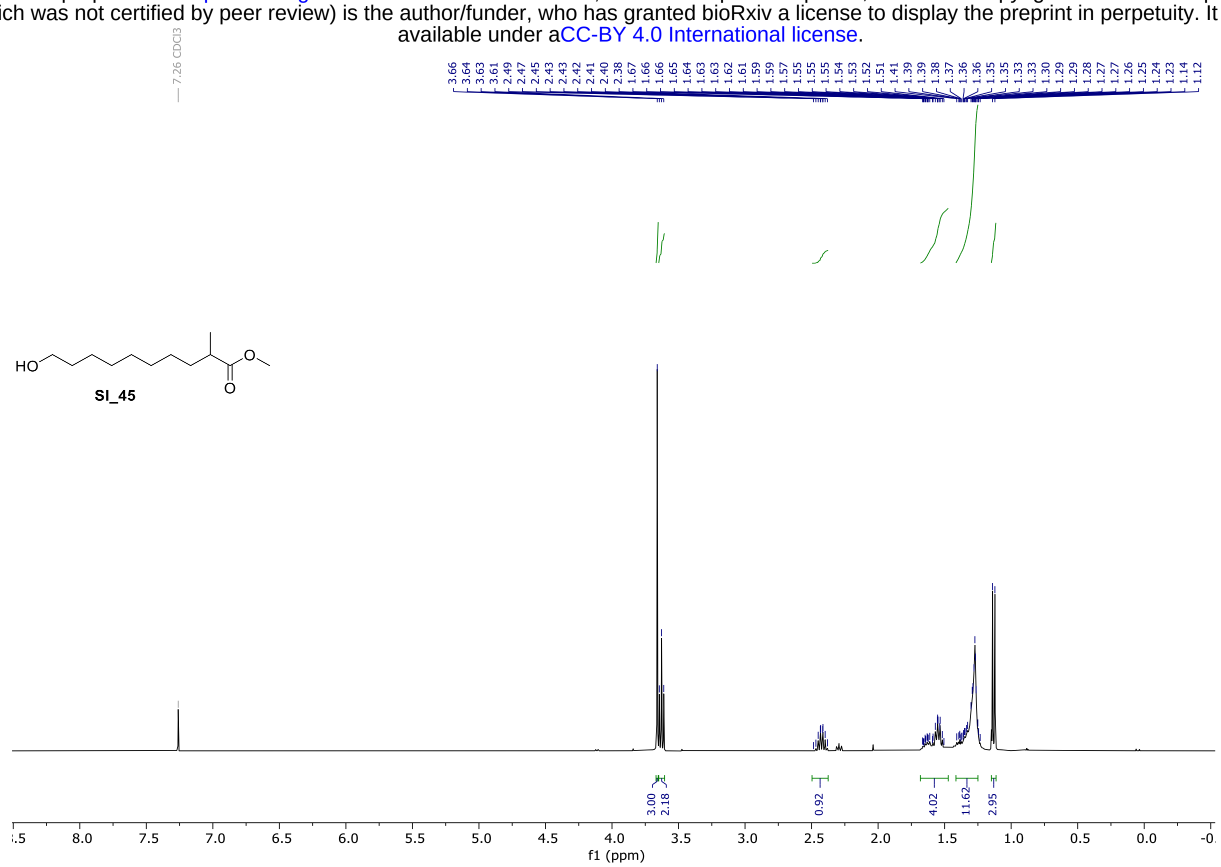

^{1}H NMR (400 MHz, $CDCl_3$) of **SI_45**.

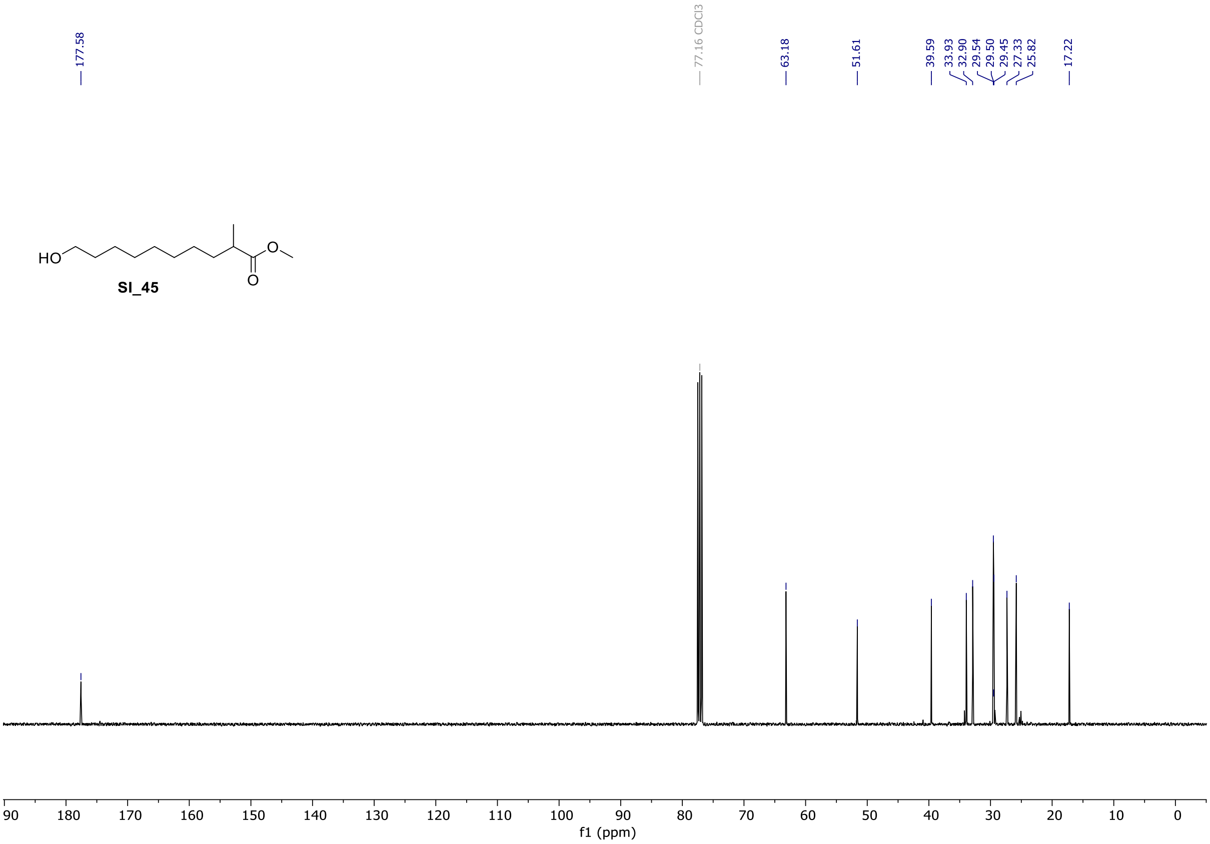

^{13}C NMR (101 MHz, $CDCl_3$) of **SI_45**.

bioRxiv preprint doi: https://doi.org/10.64898/2026.04.24.720406; this version posted April 27, 2026. The copyright holder for this preprint (which was not certified by peer review) is the author/funder, who has granted bioRxiv a license to display the preprint in perpetuity. It is made available under aCC-BY 4.0 International license.

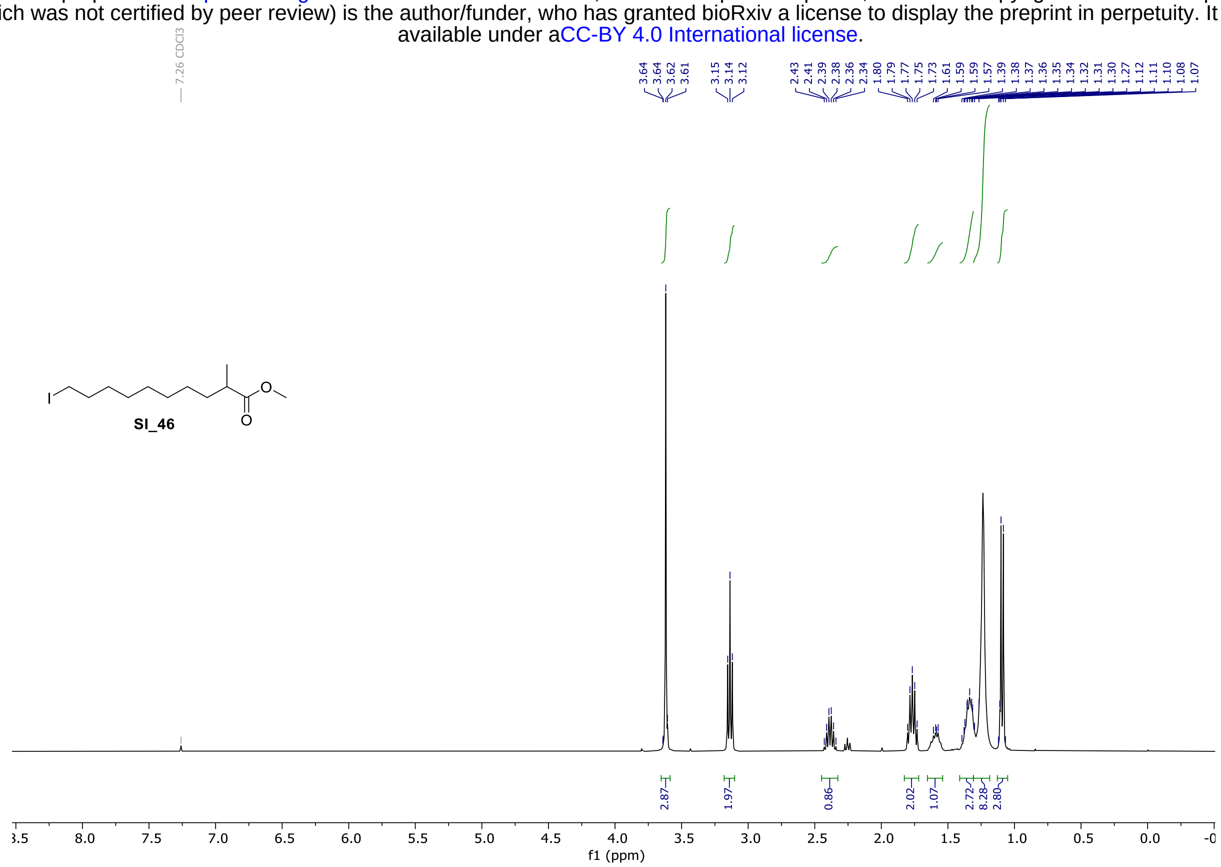

^{1}H NMR (400 MHz, $CDCl_3$) of **SI_46**.

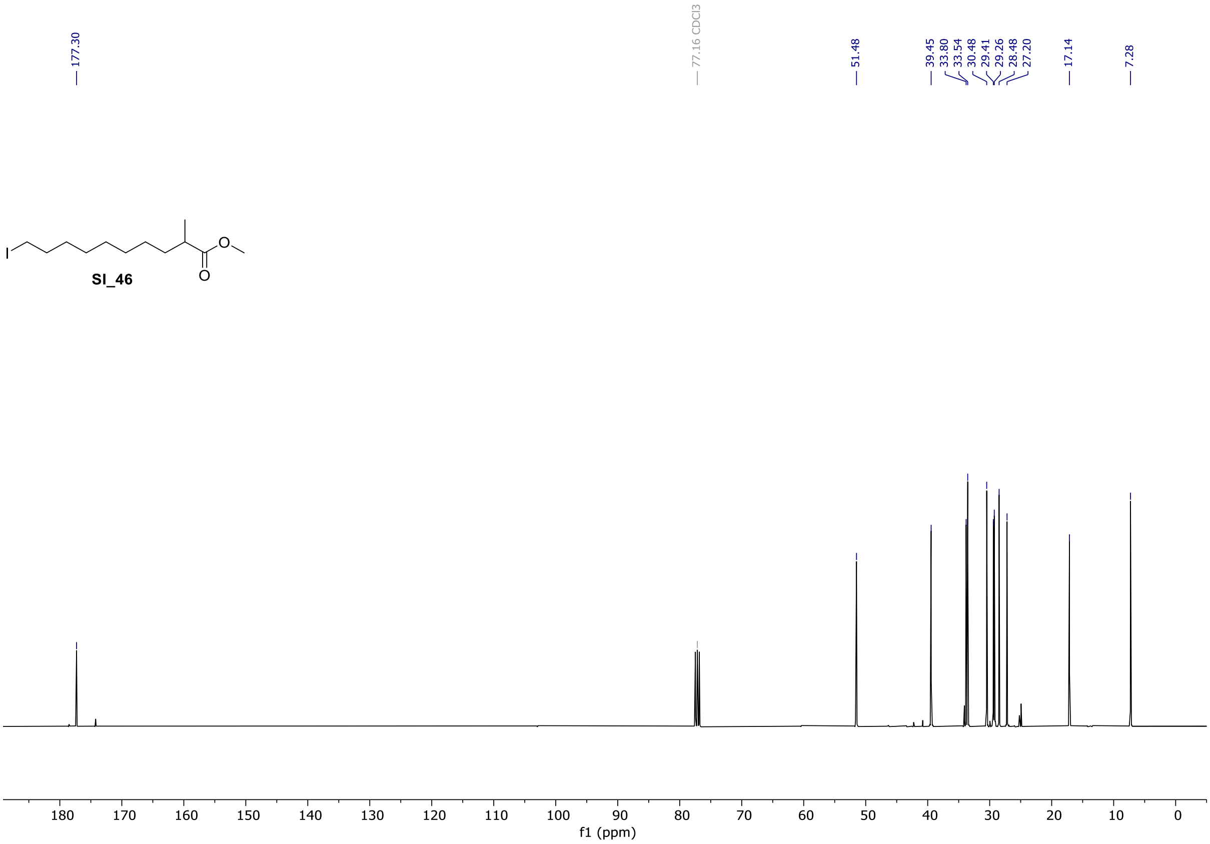

^{13}C NMR (101 MHz, $CDCl_3$) of **SI_46**.

bioRxiv preprint doi: https://doi.org/10.64898/2026.04.24.720406; this version posted April 27, 2026. The copyright holder for this preprint (which was not certified by peer review) is the author/funder, who has granted bioRxiv a license to display the preprint in perpetuity. It is made available under aCC-BY 4.0 International license.

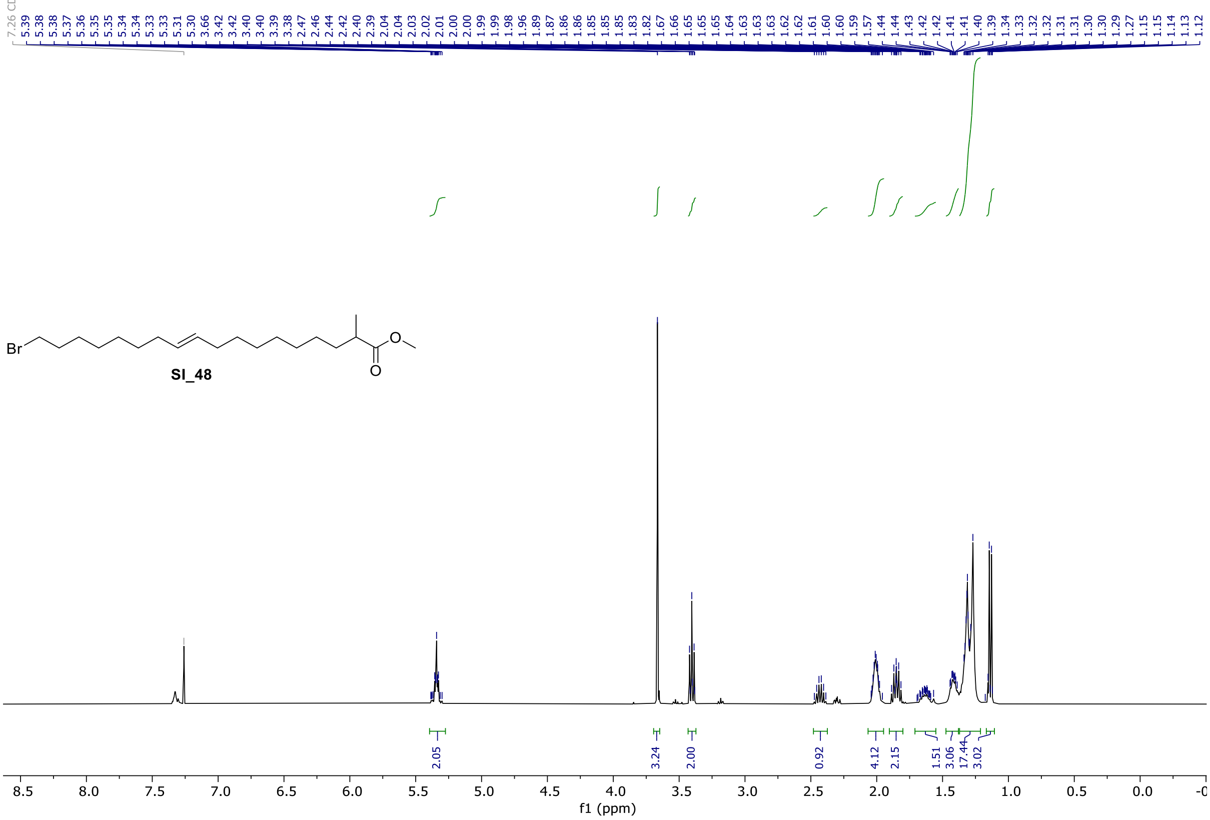

^{1}H NMR (400 MHz, $CDCl_3$) of **SI_48**.

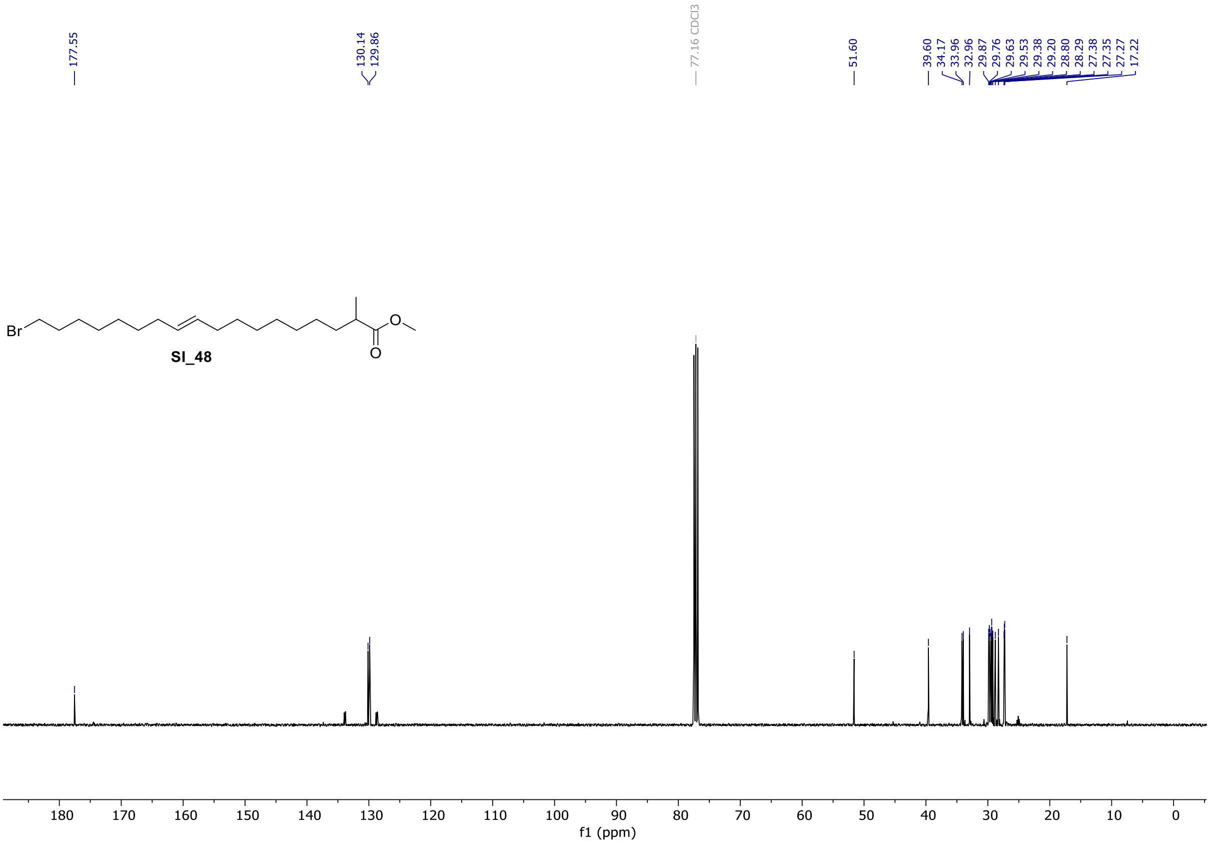

^{13}C NMR (101 MHz, $CDCl_3$) of **SI_48**.

bioRxiv preprint doi: https://doi.org/10.64898/2026.04.24.720406; this version posted April 27, 2026. The copyright holder for this preprint (which was not certified by peer review) is the author/funder, who has granted bioRxiv a license to display the preprint in perpetuity. It is made available under aCC-BY 4.0 International license.

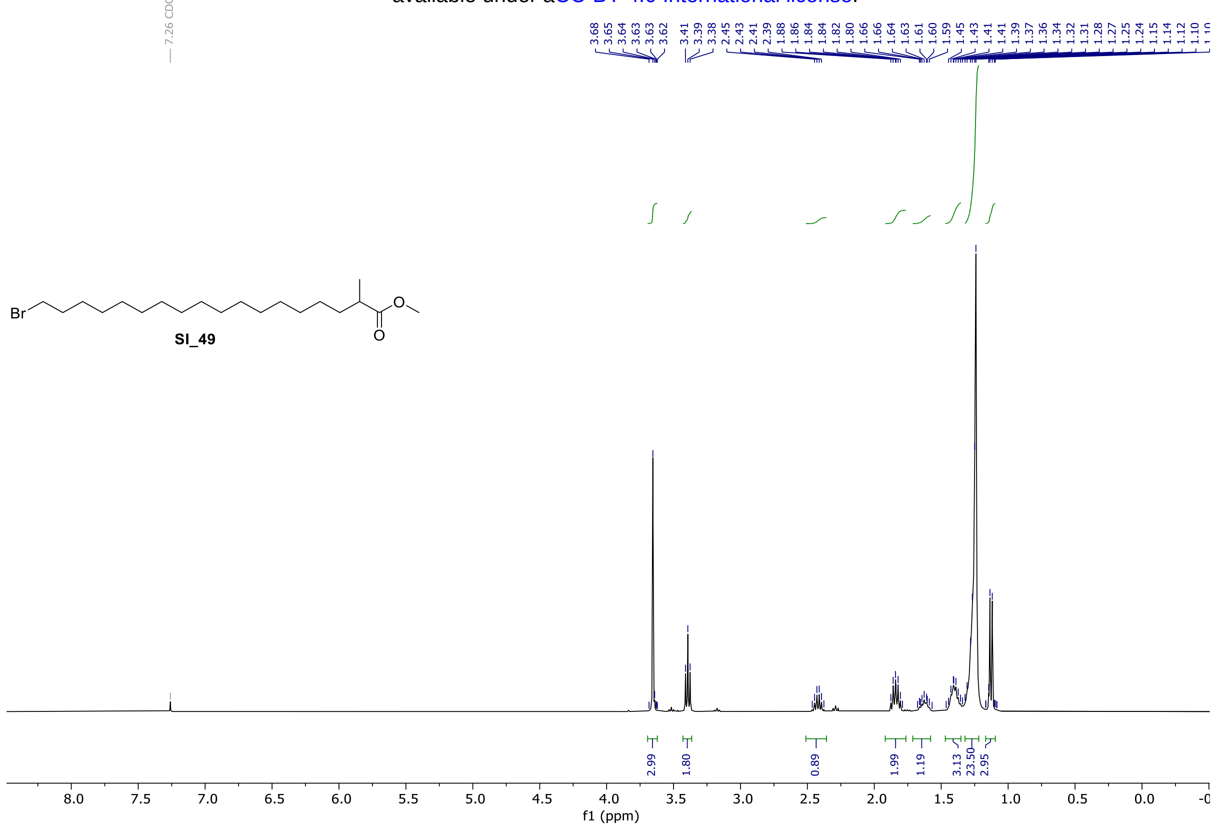

^{1}H NMR (400 MHz, $CDCl_3$) of **SI_49**.

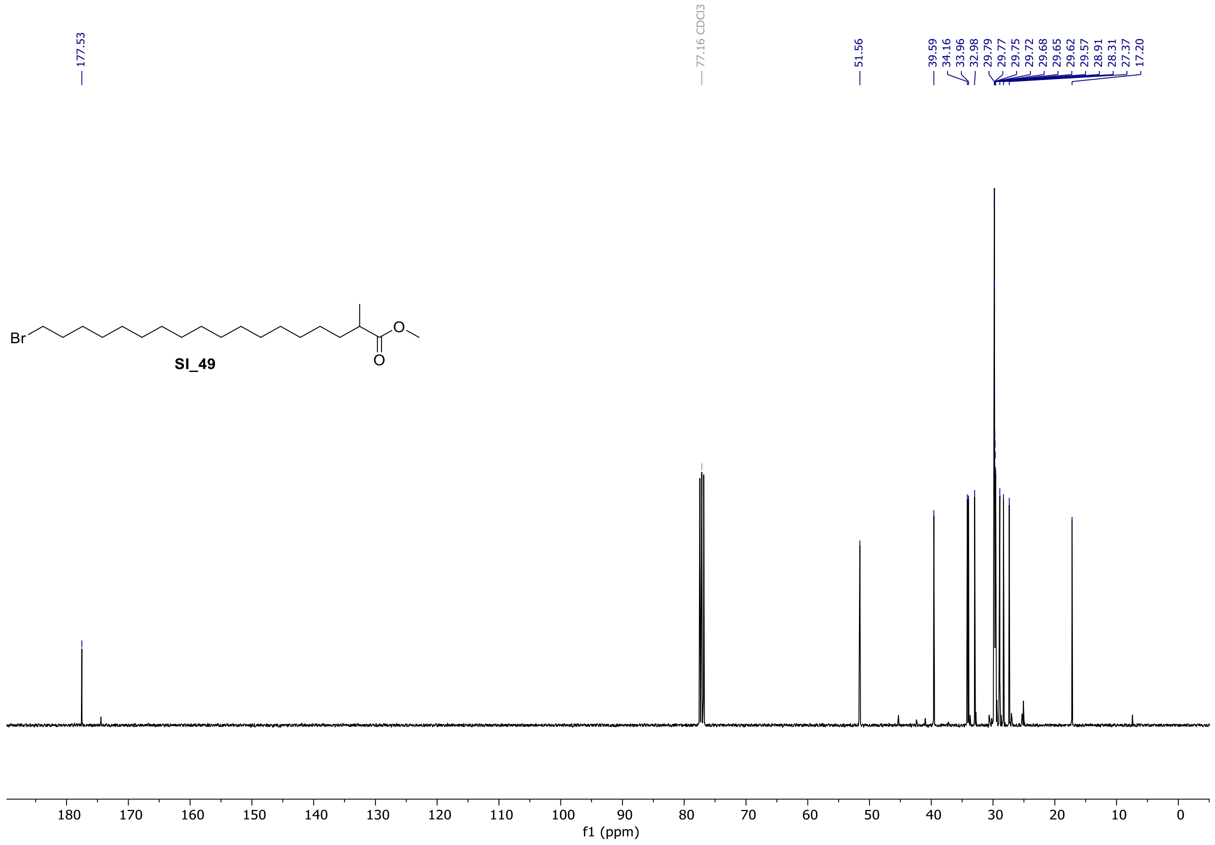

^{13}C NMR (101 MHz, $CDCl_3$) of **SI_49**.

bioRxiv preprint doi: https://doi.org/10.64898/2026.04.24.720406; this version posted April 27, 2026. The copyright holder for this preprint (which was not certified by peer review) is the author/funder, who has granted bioRxiv a license to display the preprint in perpetuity. It is made available under aCC-BY 4.0 International license.

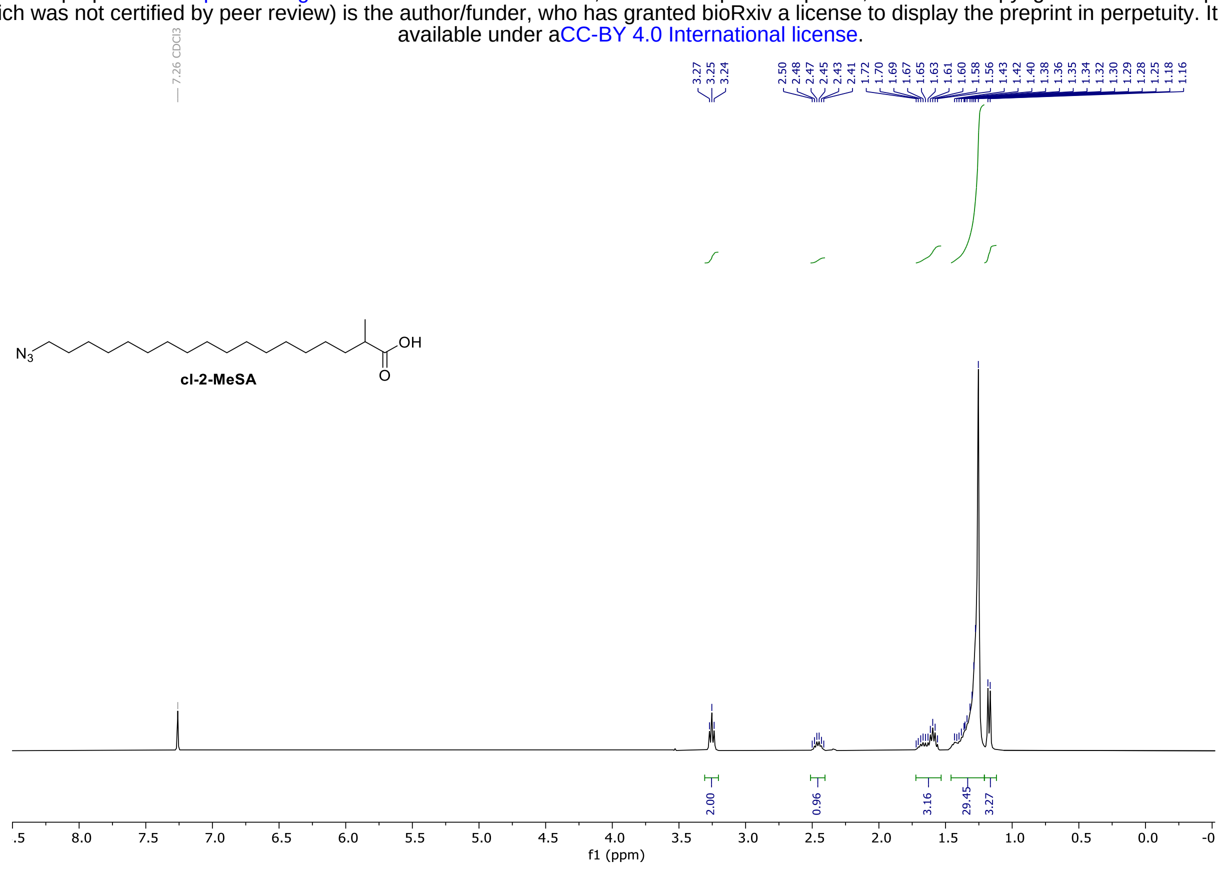

¹H NMR (400 MHz, $CDCl_3$) of **cl-2-MeSA**.

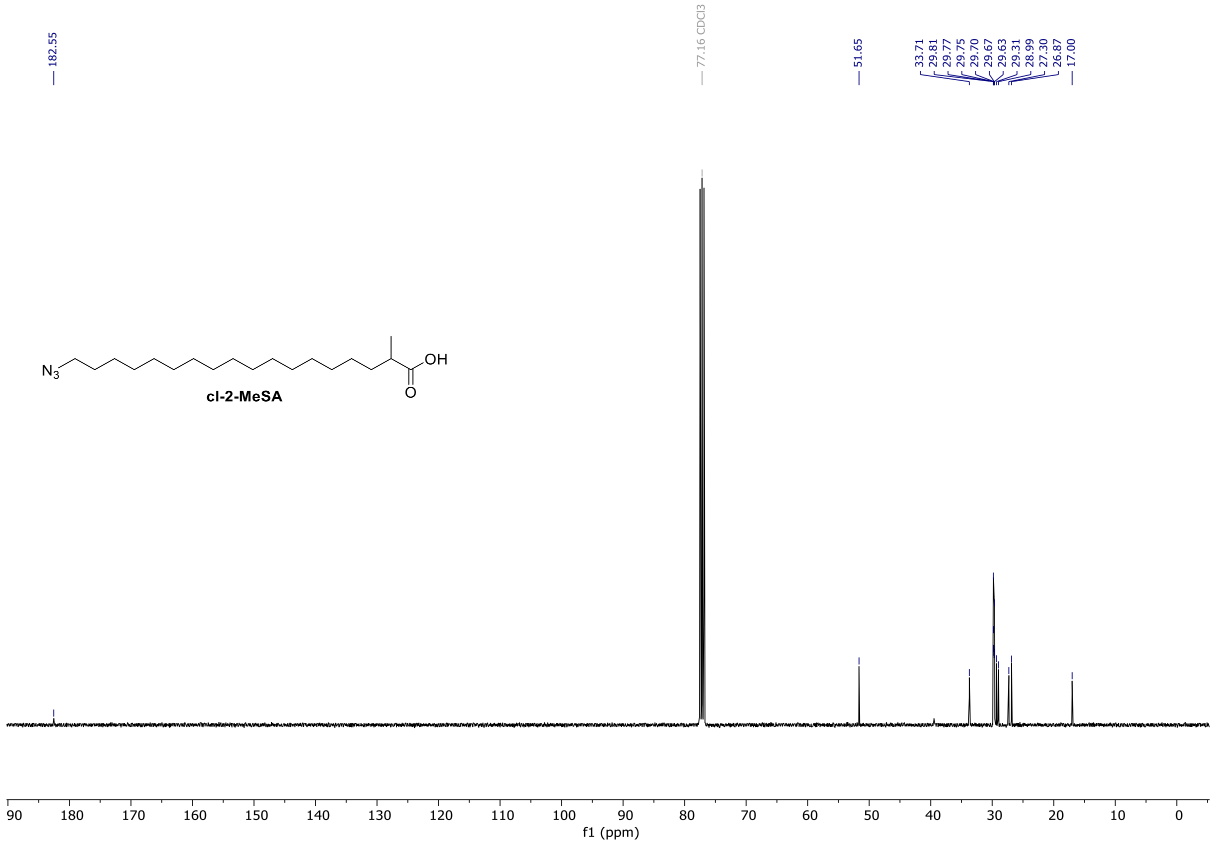

¹³C NMR (101 MHz, $CDCl_3$) of **cl-2-MeSA**.
